# Genome-Wide Computational Identification of Biologically Significant Cis-Regulatory Elements and Associated Transcription Factors from Rice

**DOI:** 10.3390/plants8110441

**Published:** 2019-10-23

**Authors:** Chai-Ling Ho, Matt Geisler

**Affiliations:** 1 Faculty of Biotechnology and Biomolecular Sciences, Universiti Putra Malaysia, 43400 UPM-Serdang, Selangor, Malaysia; 2 Division of Plant Biology, School of Biological Science, Southern Illinois University Carbondale, 1125 Lincoln Ave., Life Science II, Carbondale, IL 62901-6509, USA

**Keywords:** bioinformatic prediction, co-expressed genes, in silico, cDNA microarray, correlation

## Abstract

The interactions between transcription factors (TFs) and cis-acting regulatory elements (CREs) provide crucial information on the regulation of gene expression. The determination of TF-binding sites and CREs experimentally is costly and time intensive. An in silico identification and annotation of TFs, and the prediction of CREs from rice are made possible by the availability of whole genome sequence and transcriptome data. In this study, we tested the applicability of two algorithms developed for other model systems for the identification of biologically significant CREs of co-expressed genes from rice. CREs were identified from the DNA sequences located upstream from the transcription start sites, untranslated regions (UTRs), and introns, and downstream from the translational stop codons of co-expressed genes. The biologically significance of each CRE was determined by correlating their absence and presence in each gene with that gene’s expression profile using a meta-database constructed from 50 rice microarray data sets. The reliability of these methods in the predictions of CREs and their corresponding TFs was supported by previous wet lab experimental data and a literature review. New CREs corresponding to abiotic stresses, biotic stresses, specific tissues, and developmental stages were identified from rice, revealing new pieces of information for future experimental testing. The effectiveness of some—but not all—CREs was found to be affected by copy number, position, and orientation. The corresponding TFs that were most likely correlated with each CRE were also identified. These findings not only contribute to the prioritization of candidates for further analysis, the information also contributes to the understanding of the gene regulatory network.

## 1. Introduction

Cis-acting regulatory elements (CREs) are DNA sequences that reside in the neighboring region of structural genes that are necessary to regulate differential gene expression. CREs can be transcription factor binding sites (TFBSs), targets for miRNA suppression, and recognition sites for nucleosome positioning, chromatin remodeling, methylation, and other non-coding sequence-specific regulatory mechanisms. Transcription factors (TFs) bind TFBSs in the promoter sequence, to switch on and off or adjust the rate of transcription of their target genes. This results in patterns of gene expression temporarily and spatially in response to developmental or environmental signals. The immunoprecipitation of TF-bound chromatin, chromatin immunoprecipitation (ChIP)-chip, ChIP-seq, DNase-seq, SELEX-seq, and protein-binding microarrays (PBMs) are some of the techniques that facilitate the determination of TFBSs experimentally [1,2,3,4,5], albeit detailed characterization of TFBSs could be cost and time intensive. Direct binding assays often do not predict the type or weight of the regulatory influence.

The in silico identification and annotation of TFs, and the prediction of plant CREs are made possible by recent advances in genome sequencing and transcriptome analysis [6,7,8,9]. There are many databases and bioinformatics tools for plant TF and CRE analyses (as reviewed by Garg and Jaiswal [10]) such as the Plant cis-acting regulatory DNA elements (PLACE) database [11], Plant cis-acting regulatory element (PlantCARE) database [12], PlantTFDB 4.0 [6], and many others. PLACE and PlantCARE are public depositories of plant CREs, while PlantTFDB 4.0 is a comprehensive database for plant TFs, and information on the TF binding motifs (TFBMs) was derived from experiments, regulatory elements, and regulatory interactions. Meanwhile, PlantCARE and BAR [13] also provide tools for the in silico analysis of promoter sequences. Most of the well-characterized plant promoter sequences in these databases are for model organisms such as *Arabidopsis thaliana*. While several well-known CREs (such as the abscisic acid-responsive element, or ABRE, and the drought-responsive element, or DRE) have been shown to be somewhat conserved among flowering plants, the degree of functional conservation of other plant CREs is unknown. It is reasonable to assume that there would be some lineage-specific as well as deeply conserved CREs among plant promoters.

Rice is an important food crop, and a model for monocotyledonous plants. The completion of whole genome sequence (by the International Rice Genome Sequencing Project in 2005) has advanced basic and applied research in rice. Several rice microarray platforms have been developed for global gene expression studies of rice, including the National Science Foundation (NSF) 22K and 45K Rice Oligonucleotide Arrays, Agilent 44K arrays, Affymetrix arrays, and the Yale/BGI oligonucleotide array [14]. An analysis of promoters from co-expressed genes from microarray data has facilitated the prediction of rice CREs [7,9,15,16,17]. However, some of these predicted CREs could be short motifs that occur randomly throughout the genome without regulatory function. To examine the biological significance of these predicted CREs and demonstrate their contribution to the promoter activity, Geisler et al. [18] have developed an algorithm that statistically correlates the presence or absence of the CRE in genes with their expression profile on multiple DNA microarrays. Meanwhile, gene regulatory network reconstruction was shown to be able to improve the predictive power by using a combination of co-expression and CRE-TF data for the accurate identification of CREs [19].

In this study, we tested the applicability of the algorithm developed by Geisler et al. [18] for the identification of predicted CREs along with their potential regulatory function from co-expressed genes in rice, using a meta-database constructed from the rice microarray data. The predicted biologically function of rice CREs was determined by correlating their absence and presence, orientation, and position with the gene expression profiles across multiple experimental treatments. In addition, we also identified potential TFs associated with rice CREs by correlating the differential expression patterns of the CREs and TFs using a method described by Kim and Kim [20]. This information not only aids in the prioritization of candidate rice CREs and TFs for genetic and biochemical analyses, the TF-CRE predictions also provide links for reconstructing CRE-anchored gene regulatory networks in rice.

## 2. Results and Discussion

In this study, using the algorithm reported by Geisler et al. [18], previously experimentally determined plant CREs were tested on the constructed rice expression meta-database prior to the prediction of rice CREs and the further testing of the biological significance of new CREs, CRE variants, and TFBSs. In addition, TFs associated with the rice CREs were also predicted. Throughout this paper, we distinguish CREs as: 1. Predicted if identified solely by expression correlation methods in this study. 2. Known if previously published using some other experimental methodology. 3. Validated, if two or more unrelated methods result in the same CRE sequence. We use the term CRE generically to refer to multiple classes, or to refer to the hypothetical true CRE.

### 2.1. Testing and Validation of Known Plant CREs on the Constructed Rice Expression Meta-Database

**Figure 1:**
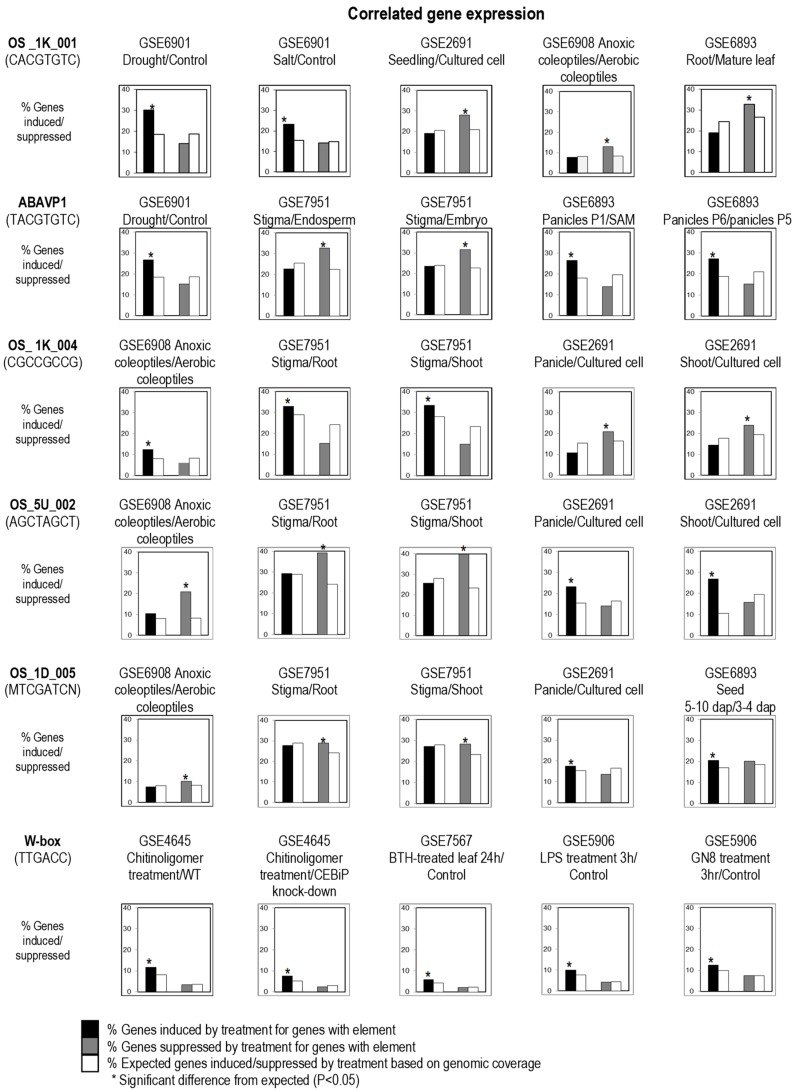
Regulatory fingerprints of cis-acting regulatory elements (CREs). Rice genes bearing predicted or known CREs were scored as either two-fold induced, two-fold suppressed, or neutral by pair-wise comparison of gene expression under two test conditions indicated above each bar chart. As a test of CRE function, the population of genes with the CRE was considered responsive to the test conditions if a significantly (*; *p* < 0.05) greater number of genes were induced (black bars) or suppressed (grey bars) than that expected by chance (white bars). SAM, shoot apical meristem; P1, panicles at 0–3 cm; P5, panicles at 15–22 cm; P6, panicles at 22–30 cm; dap, days after pollination; BTH, benzothiadiazole; GN8, N-acetylchitooligosaccharide; and LPS, lipopolysaccharides.

The algorithm that was originally used by Geisler et al. [18] for the identification and testing CREs and their potential regulatory roles from the co-expressed genes of *A. thaliana* was examined to evaluate whether the algorithm works equally efficient and reliably for the rice genomic and expression data by using previously known experimentally determined CREs. The PATMATCH application [21] was used to generate a list of patterns matching sequences from the rice genome that contain a particular CRE at 1 kb upstream of the annotated transcription start (TSS). The genomic distribution of known CREs from rice was correlated with rice expression data from multiple DNA microarrays covering various experimental conditions. In our terminology hereafter, a predicted CRE is considered if: 1. The frequency of occurrence in the 1 kb upstream was significantly higher than expected based on random nucleotide model in a forward approach; and 2. The genes with the putative CRE in the 1 kb upstream are significantly more induced or suppressed compared with all the genes in the genome using a chi-squared test (*p* < 0.05) in a reverse approach. A rice gene expression meta-database consisting of 231 relative gene expression (log_2_ [ratio]) values of multiple genes under different conditions or treatments (Supplementary Table S1) was assembled and computed for this purpose. A chi-squared test then determined the likelihood (*p*-value) that the observed enrichment of differential regulation of genes with the CRE was likely due to chance. The pattern of differential expression enrichment across multiple experiments associated with a predicted or known CRE in their 1-kb upstream region is potentially diagnostic of the regulatory function of that CRE and the trans-acting factors associating with it. Thus, the enrichment pattern is similar to a fingerprint of regulation, and is hereafter referred to as the regulatory fingerprint of the CRE. This provided experimental evidence for the biological functionality, the type of regulation (induction or suppression), and the signal pathways (by treatment) eliciting a response for a CRE in planta. In this test, we assume that a gene bearing a true CRE should be significantly more upregulated or downregulated under one or more specific treatments compared to the average of the whole genome (Figure 1).

We tested the method with a list of known CREs containing the core abscisic acid (ABA) responsive element (ABRE; ACGT) (Table 1), W-box (TTGACC/T) (Table 2), and ethylene-responsive element (ERE, AGCCGCC) (Table 2) that have been reported and validated previously. The CREs with ABRE core in the sequences such as ABRE-M3, ABREA, ABRE/EM/RAB21, ABRE (MEME), and CBF3 were found to be significantly more likely to be induced by drought and salt, respectively (Table 1). Our results are supported by previous findings that reported the enrichment of ABRE elements in the promoter of the genes associated with plant abiotic stresses, including drought and salt [18,22]. 

The ABRE core binds specifically to basic leucine zipper (bZIP) TFs, whereby some of these TFs are involved the regulation of abiotic stress response related to ABA [23]. In addition, genes containing W-box in their promoters were found to be significantly more likely to be induced by chitin, liposaccharides, and benzothiadiazole (BTH), which can activate the salicylic-acid (SA) signaling pathway. The W-box binds specifically to the WRKY TF with a conserved amino acid sequence WRKYGQK at its N-terminal beta strand, and a four-stranded beta sheet with a zinc-binding pocket [24]. Our result concurs with previous findings that WRKY is involved in the regulation of plant defense against biotic stresses [25]. Meanwhile, the ERE was found to be correlated with the upregulation of genes in anoxic rice coleoptiles, and in cultured cells compared to the seedlings (Table 1). It is not surprising, because ethylene production was found to be enhanced by anaerobic condition [26]. The rate of ethylene production also increased during rapid cell growth in vitro [27], and ethylene production has been reported in plant suspension cultures whereby the amount of ethylene produced varied with species [28].

In summary, we demonstrated that the algorithm was able to validate the known and well-characterized plant CREs tested in this study and provide potential regulatory roles and signals related to their previously known biological functions. The results not only further confirmed the reliability of the algorithm in testing the biological significance of plant CREs, but also demonstrated the applicability of the rice gene expression meta-data that we have assembled and computed for this study for the ensuing analyses.

### 2.2. Identification of New CREs and Their Potential Function and Testing of Alternative Regulatory Roles of Variants of Known CRE and TFBSs

In forward searches, co-expressed genes were identified from the rice gene expression meta-data assembled in this study. CREs that were overrepresented in co-expressed genes across multiple arrays were compared with the whole genome using a species-specific background model and were then identified by MotifSampler [29]. In this study, CREs located in five regions around co-expressed rice genes: the 3-kb and 1-kb upstream sequences from TSS, 5′-untranslated regions (UTRs), introns, and 1-kb downstream sequences from transcription stop codons were analyzed. The CREs were named OS _AA_NNN where OS = *Oryza sativa*; AA = 1K or 1D or 3K or 5U, and UT, which indicates the location from which the CRE was found i.e., 1 kb upstream, 1 kb downstream, 3 kb upstream, 5′-UTR, and introns, respectively; and NNN = order of discovery. The CREs predicted could be either TFBSs, targets for miRNA suppression, recognition sites for nucleosome positioning, chromatin remodeling, methylation, and other non-coding sequence-specific regulatory mechanisms. 

The forward search was reported to have high rates of false-positive results [30]. Furthermore, the biological significance of many known CREs that occur frequently in plant promoter sequences such as ACGT, which is found in nearly every plant gene promoter, is questionable [18]. By correlating the distribution of CREs throughout all the promoter regions of the rice genome with the regulation of gene expression under specific treatments, these dubious known CREs can be either removed or selected for further verification [18]. Hence, significantly overrepresented expression patterns of genes with a predicted or known CRE (retrieved by PATMATCH) were also identified in reverse searches in this study. The overrepresented differential expression patterns of genes with a CRE may show regulation by the same trans-acting “cause” (Table 1). For example, genes with OS_1K_001 (a predicted CRE with an ABRE core) was found to have overrepresented expression patterns related to drought and salt, which are both due to water stresses, and also sucrose starvation and reproductive plant development (Table 1, Figure 1). This could be caused by the involvement of the hormone ABA (as a common cause) in both plant responses to environmental stresses and developmental programs [31]. Thus, the regulatory fingerprint allows us to examine how different inputs (treatments) affect the regulation of genes bearing the same CRE. Furthermore, a CRE that showed correlation with two or more related stresses in different experiments by different experimenters, such as the case with OS_1K_001 (salt and drought), is generally a much more reliable indication of the biological relevance of the CRE. 

Since many CREs identified in the forward searches were found to contain the ABRE core and GCC-box, we tested the sequence specificity of a list of ABRE- and GCC- containing CREs, including some reported CREs in literature and a few new sequences discovered by the forward search in this study e.g., OS_1K_001 and the OS_1K_004 (Table 1). In this study, we have also tested genes bearing variants of a known CRE motif (both natural occurrence and mismatches created specifically for this study) and also a list of TFBSs discovered by Franco-Zorrilla et al. [1].

Mueller et al. [32] reported that plant bZIP TF can bind to A-box (TACGTA), C-box (GACGTC), and G-box (CACGTG); however, our findings demonstrated that rice bZIPs may preferentially bind to G-box and A-box, because the genes that contain A- or G-boxes were found to be significantly more induced/suppressed, while the genes enriched with C-box was found to have no biological significance (Table 1). Although the ABRE core motif is commonly found in the binding sites of bZIP TFs, variations in flanking nucleotides at both ends were found to be associated with differences in regulatory fingerprints and have a different level of significance in the correlation analysis. For example, ABAVPI (**T**ACGTGTC) and OS_1K_001 (**C**ACGTGTC), which differ in a single nucleotide before the core sequence, were shown to be associated with different regulatory fingerprints. The genes containing ABAVPI were significantly more induced by sucrose starvation compared to drought stress, while those enriched with OS_1K_001 were significantly more induced by drought and salt stresses, but only weakly correlated with sucrose starvation (Table 1). Similarly, the genes bearing ACGT flanked by T at the 5′-end such as ABRE (Arabidopsis), ABRE (Monocot), and ABRE (Rice) were significantly more induced by sucrose starvation compared to OS_1K_001 and OS_1K_002 (with ABRE flanked by C at 5′-end), which have weak correlation to sucrose starvation treatment. We also noticed that the genes containing ABRE_M4 (CACGTGGC) were significantly more induced by white-backed plant hopper (WBPH), whereas those containing OS_1K_001 (CACGTGTC) were not. Since the bZIP TF family has 89 members in rice [33], different bZIP TFs could be involved in ABA response during sucrose signaling, abiotic stresses, and other plant developmental programs. The adjacent sequence of the conserved ABRE core may affect the binding specificity of different bZIP TFs, orchestrating a different downstream response accordingly. Subtle differences in the primary DNA-recognition elements were also found for TFs that have up to 79% amino acid identity in Arabidopsis [1].

In this study, we also tested the genes bearing a coupling element (CE), which was reported to co-occur with ABRE in the promoter sequence [34]. We found that CE-M2 (MCGCGTCD) and CE3 (CACGCG) were more induced by drought stress but not salt stress or sucrose starvation, whereas a few other reported CEs were more induced by sucrose starvation (Table 1). In mouse, closely related TFs that share similar high-affinity binding sequences were found to have different preferred low-affinity sites that result in distinct DNA-binding profiles for different TFs [2]. Franco-Zorrilla et al. [1] also revealed that a high number of TFs in *A. thaliana*, in addition to their high affinities to their primary elements, have similar or slightly lower affinities to their secondary elements. It is unknown whether CEs with slight differences in sequence actually serve as the secondary elements for different bZIP TFs that are involved in drought stress and sucrose signaling, respectively. 

Among the new predicted CREs that we have tested, OS_1K_004 (CGCCGCCG), which contains the GCC-box, was found to have tight correlation with the genes upregulated in anoxic coleoptiles and cultured cells, and was in fact significantly more induced/suppressed in rice compared to the reported ERE (AGCCGCC) under similar conditions or in the same tissues (Table 2). GCC-box interacts with TFs in the ethylene-responsive factor (ERF) subfamily, which are usually involved in biotic stress response and ethylene signaling [35,36]. ERF1 was shown to bind specifically to GCC box [37], while other TFs in this subfamily have moderate to high affinity for other GCC variants (GCCGCA, GCCGAC, GCCGTC, and GCCGGC). Although genes with GCC variants in the promoters were upregulated in anoxic coleoptiles and cultured cells, a double repeat of GCC in the predicted CRE is critical for a higher correlation. Since the dehydration responsive element binding (DREB) family belongs to the APETALA2/ETHYLENE RESPONSIVE ELEMENT BINDING PROTEIN (AP2/EREBP), it should not be surprising that their TFBSs, i.e., the drought-responsive element (DRE; RCCGAC) and DREB1A CRE (RCCGACNT) may also contain the GCC variant (GCCGAC) sequence. Similar to other known CREs with GCC-box, both DRE and DREB1A were found to have correlation with the genes upregulated in anoxic coleoptiles (Table 2). However, neither of these known CREs was found to be overrepresented in the promoter of rice genes induced by drought nor cold stresses, as reported by [38]. Franco-Zorrilla et al. [1] suggested that the DNA binding of DREBs is complex, where some DREBs have a broader range of DNA binding by recognizing DRE and GCC-related elements. The overrepresentation of GCC-box among the upregulated genes in stigma compared with other tissues may be due to the overlapping genetic programs regulating pollination and stress responses [39]. Pollination and auxin can regulate ethylene production in plant reproductive organs [40], and GCC-box has been found in many dehydration and pathogen responsive genes that were co-expressed in stigma [39].

Table 3 shows that genes with their 1-kb upstream sequence enriched for three groups of predicted and known CREs containing either one of the following core sequences, CTAG, CATG, or TCGA, were more suppressed in anoxic rice coleoptiles, stigma, and cultured cells, in contrast to those of CREs containing the GCC-box. These CREs were also overrepresented in the 1-kb upstream sequence of rice genes that were upregulated in young inflorescence (YF) and young panicles (P1) compared to the shoot apical meristem (SAM). The core sequence CTAG was found in STY1 and STY2, which belong to a group of TFs containing a zinc-finger similar to RING domains that regulate the development of the gynoecium, stamen, and leaf [2,41,42]. STY1 was found to enhance the synthesis of auxins [41]. The genes with their 1-kb upstream sequence containing TCGA were also found to be significantly more induced in the late stage of panicle development (22–30 cm) and early stage of seed development (5–10 days after pollination; dap) (Table 4). The core sequence TCGA is found in a palindromic sequence TGTCGACA, and is identified as a secondary motif for ETTIN (Auxin responsive factor, ARF3) [1], which could be involved in auxin signaling. Auxin and ethylene have been demonstrated to act as both collaborators and competitors in growth and developmental processes. They were reported to act synergistically in root elongation and root hair formation, but act antagonistically in lateral root initiation and hypocotyl elongation [43]. Hence, it is not surprising that genes with GCC-box in their promoters have contrasting gene expression patterns with genes that are enriched in CREs with CTAG and TCGA core motifs in their promoters, respectively. 

Genes with their 1-kb upstream sequence that are enriched with CATGCA and CATGCATG were found to be significantly more induced or/and suppressed in rice seeds (Table 4). The former was also more induced or/and suppressed across different stages of panicle development, whereas genes that are enriched with CATGCATG in their 1-kb upstream sequence were only found to be more induced or/and suppressed during certain stages of panicle development (Table 4). Although CATG is also present in the secondary binding site of PHYTOCHROME-INTERACTING FACTOR (PIF) 3, PIF4, and MYC2 [44,45] i.e., the PBE-box, we found that the genes containing the PBE-box (CACATG and ACACATG) were only significantly more induced/suppressed in rice panicles tissues, but not in the seeds (Table 4).

Genes with their 1-kb upstream sequence containing the primary (CAATCA) and secondary binding motif (TAATTA) of WOX13, which belongs to a group of homeodomain (HD) TFs [1], share the same regulatory fingerprints; i.e., they are significantly more induced in young panicles compared to SAM, and root compared to cultured cells, as well as more suppressed in rice anoxic coleoptile and stigma (Table 3). These motifs are partially identical to that recognized by WUSCHEL (WUS) TF during the transcriptional activation of AGAMOUS [46]. The AT-rich motifs in HD proteins containing a leucine zipper domain (HD-ZIP) such as INCURVATA4 (ICU4), LATE MERISTEM IDENTITY1 (ATHB51), and Yabby shared some of the regulatory fingerprints of WOX13 (Table 4). The current prediction can only identify the consensus motif-binding site of these TFs; individual TFs in this group may have preferred binding sites or secondary elements in addition to the consensus-binding motif or act in concert with other TFs to direct different plant developmental programs.

Other examples of CRE include OS_CRE_3K_002 (AACCAAC) which correlates with genes upregulated in ice anoxic coleoptiles, OS_1D_002 (TAATTAAT) enriched in genes that are more suppressed in stigma compared to root and shoot, and OS_1K_005 (CAAAACGC), which correlates with genes expressed in pollen. Other CREs not shown in this study were identical to previously identified elements.

### 2.3. Identification of Co-Existing Motifs

To identify other co-existing motifs that are also enriched among the same sequences (including the secondary elements that bind to the same TF), we used the promoter or upstream sequences containing a particular predicted CRE of interest (from PATMATCH) as input for MotifSampler. By doing that, a motif (CGTGKCNS) was found to co-exist and enriched in the 1-kb upstream rice sequences containing OS_1K_001 (CACGTGTC). This motif was found to share some similarities with that of ATCE (GACRCGTGKC), which is known to be a coupling element of ABRE. The 1-kb upstream rice sequences containing the following CREs: ABAVPI (TACGTGTC), RiceABRE (CGTACGTGTC), ABRE_M4 (CACGTGGC), and ABRE_M3 (MCACGTGKC) were also searched; however, the CE were not found to co-occur with ABRE (Rice) and ABAVPI that are flanked by T immediately upstream of ACGT, whereas CG-rich or G-rich CREs were found in the promoter of many sequences containing ABRE_M3 and ABRE_M4.

The predicted CREs that co-occur with OS_5U_002 (AGCTAGCT) i.e., GATCGATC and MGATCGAK, have overlapping nucleotides GATC with those of OS_5U_005 (NMTCGATC) and OS_1D_005(MTCGATCN) that were predicted earlier and shared the same expression patterns with OS_5U_002. GATA-type proteins (TFs that bind to DNA sequence “GATA”) such as GATA12, GATA nirate-inducible carbon-metabolism involved protein (GNC), and GNC-like (GNL) were found to bind to TFBD with the GATC core [1]. Both GNC and GNL are repressors of gibberellin signaling in plant developmental programs [47]. The A-rich CRE, AAARAAAA, was found to co-occur with OS_5U_002, whereby AAAG is known to be recognized by DNA-binding with one finger (DOF) domain proteins [1]. We demonstrated that it is possible to identify co-existing CREs from a list of gene sequences, that form a possible regulatory module. This process can be repeated iteratively in the future if necessary to identify all of the elements of a larger cis-regulatory module.

### 2.4. Effects of Position, Orientation, and Copy Number on the Biological Significance of CREs

**Figure 2:**
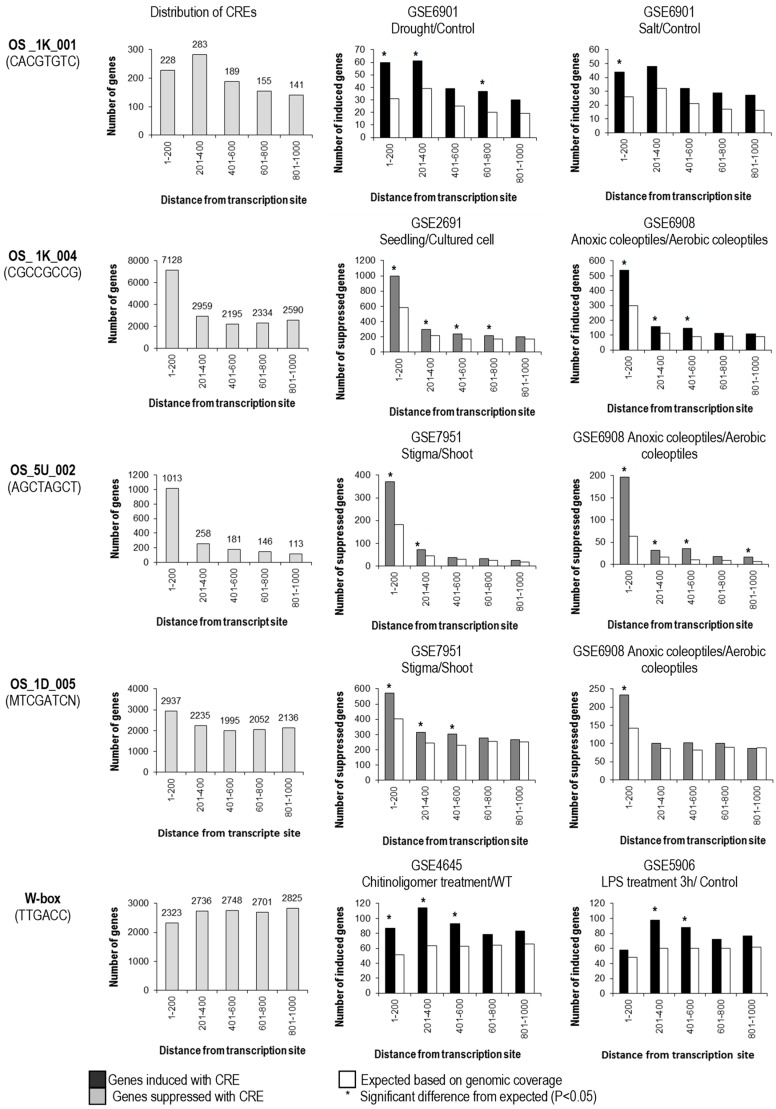
Distribution of CREs within the 1-kb upstream sequence from the transcription start site and positional requirement for the effectiveness of CREs. Genes bearing CREs (shown at the right panel) in different locations upstream of the promoter were subdivided and assayed separately for a significant effect on regulation. By comparison to expected number regulated genes randomly drawn from the genome (white bars), CREs at different positions were scored as having a significant (*; *p* < 0.05) or non-significant effect on regulation in each microarray experiment.

Most predicted CREs were found to be enriched within the 0.2-kb upstream sequences from TSS (such as OS_1K_004 and OS_5U_002). OS_1K_001 has the highest distribution at 201–400 bp upstream, while in contrast, OS_1D_005 and W-box were quite evenly distributed in the 1-kb upstream sequence from TSS (Figure 2). The distance of CREs from the TSS was demonstrated to affect the biological significance (association of the presence of the CRE with differential regulation) of some CREs. OS_1K_004 and OS_5U_002 were found to be biologically significant when located between 1 and 800 bp upstream of TSS; whereas OS_1K_001, OS_1D_005 and W-box were found to be effective at 1–600 bp upstream of TSS (Figure 2). Predicted CREs with significant correlation to gene expression were from the 1-kb upstream sequence from TSS; however, a few CREs that were predicted from the 5′-UTRs and 1-kb downstream of the TSC were also found to be biologically significant at the 1-kb upstream sequence, notably OS _5U_005 (NMTCGATC) and OS_5U_002 (AGCTAGCT) (Figure 2). Relatively, very few CREs with significant correlation were found in the introns. Since OS_5U_005 shares high sequence identities to OS_1D_005 (MTCGATCN), which was predicted in the 1-kb downstream sequence, only the positional effect of the latter was analyzed (Figure 2).

**Figure 3:**
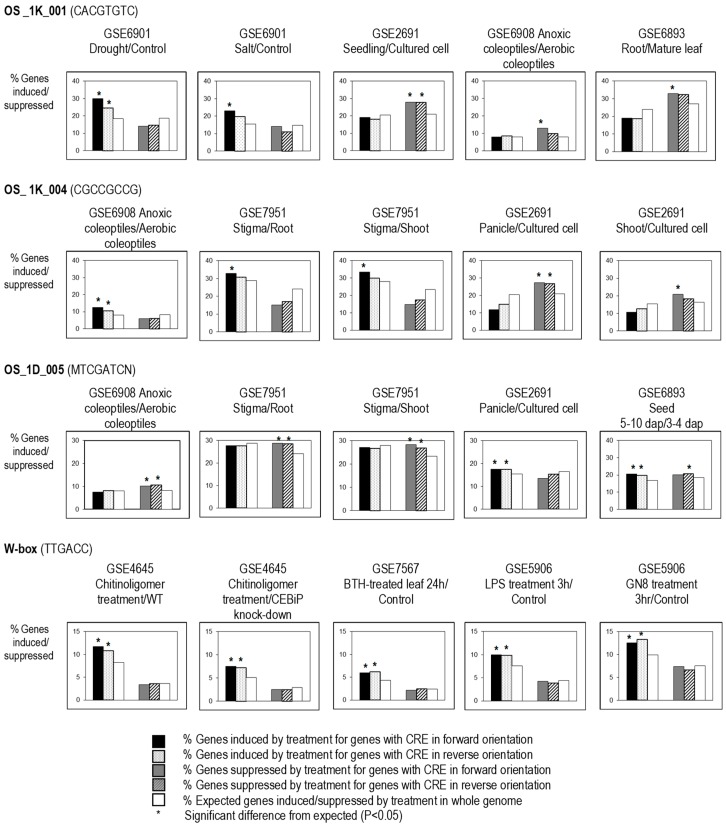
Orientation requirement for the effectiveness of CREs. Genes with non-palindromic CREs in either forward (on the sense strand) or reverse (antisense) orientation in the 1-kb upstream sequence from the transcription start site were tested: where the population of genes with the element is significantly (*; *p*-value < 0.05) more induced or suppressed than expected in both orientations, the element is considered to be bidirectional; where only one orientation of CRE shows significantly different expression than expected, then it is considered orientation-sensitive or unidirectional.

The effectiveness of several non-palindromic predicted CREs were also found to be affected by their orientation (forward or reverse with respect to the gene); for example, OS_1K_001 (CACGTGTC) was found to be significantly correlated to genes upregulated by salt, in anoxic coleoptiles versus aerobic coleoptiles and in root versus mature leaf, but only in forward orientation, whereas it was not significantly correlated to these conditions when it occurred in the reverse orientation. However, OS_1K_001 was significantly correlated to genes upregulated by drought, and seedlings versus cultured cells in both orientations (Figure 3). We might presume that multiple TFs from different signaling pathways may compete for the same CRE, and that the TF binding to the CRE could functional in either orientation (i.e., TFs involved in drought signaling), with other TFs might be orientation sensitive (i.e., TFs involved in salt signaling). Likewise, OS_1K_004 was only significantly correlated to upregulated genes in stigma versus root or shoot, and to downregulated genes in shoot versus cultured cell in forward orientation, but was significantly correlated to anoxic coleoptiles and to downregulated genes in panicles versus cultured cell in both orientations. On the other hand, W box and OS_1D_005 were found to be effective in both orientations in most of the regulatory fingerprints (Figure 3).

**Figure 4:**
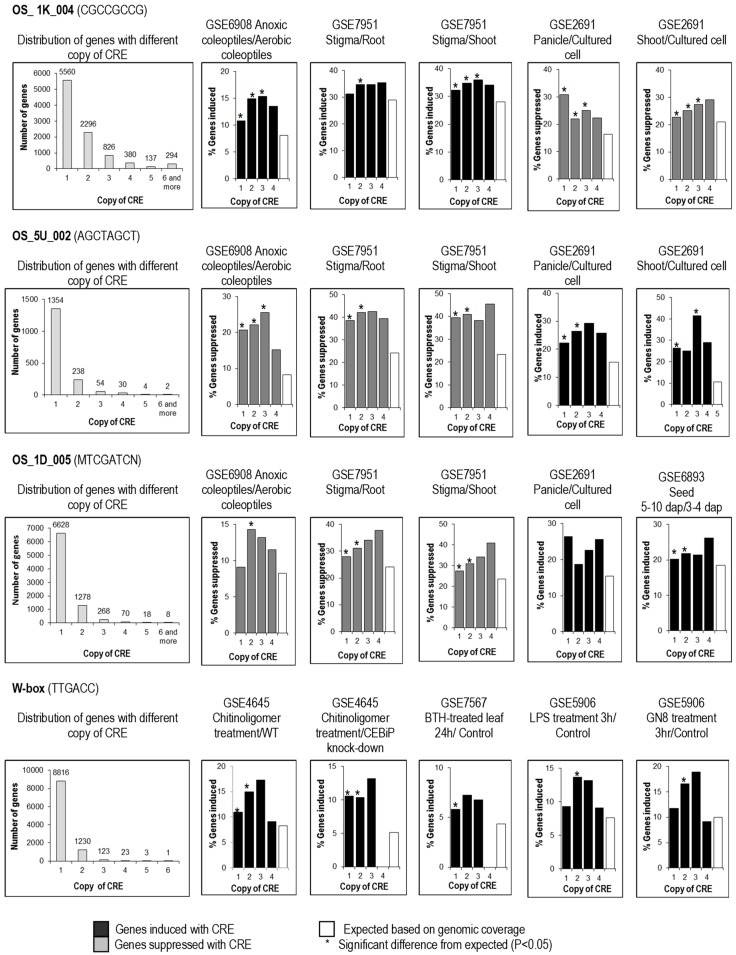
Distribution of genes with different copy numbers of CRE and copy number requirement for effectiveness of CREs. Genes were scored for the number of copies of CRE in their 1-kb upstream sequence from the transcription start site. The number of genes differentially regulated (vertical axis) was calculated in five DNA microarrays. By comparison to expected number regulated genes randomly drawn from the genome (white bars), CREs in different copy numbers (1–4) were scored as having a significant (*; *p* < 0.05) or non-significant effect on regulation in each microarray experiment.

In a related study, we examined the influence of multiple copies of the same predicted CRE in the same promoter region. Our analysis showed that the predicted CREs examined occur naturally in 1–2 copies in most 1-kb upstream gene sequences bearing them; however, they were also found in multiple copies in 1-kb upstream gene sequences of a small number of genes (Figure 4). OS_1K_004 and OS_5U_002 were found to be biologically significant from 1–3 copies (Figure 4), while W box and OS_1D_005 were only biologically significant in 1–2 copies. Thus, the probability of an effect seems not to be much affected by copy number; however, the magnitude of effect (the fold change of the affected genes) may be affected, and should be further studied, but was beyond the scope of this study.

### 2.5. Correlation Analyses of Putative CREs and Associated TFs

In this study, we retrieved the expression data of a list of genes containing a particular CRE, and created a z-score using the methods of [20], which represents the average expression pattern of this gene group. Then, this z-score was compared to the expression patterns of 1825 individual TFs (out of 1869 TFs from the PlantTFDB classified into 56 families including bHLH, NAC, ERF, MYB, FAR1, C2H2, WRKY, and bZIP) available in the rice cDNA expression database, to identify TFs whose expression was most closely matched to the expression pattern of the cohort of genes with a particular CRE enriched in their promoters. Table 5 lists the TFs that were most likely correlated (r ≥ 0.45 or r ≤ −0.45) with the CRE-containing gene sets, while the complete set of TFs is provided in Supplementary Table S2. A few of these predicted TFs were found to possess the CRE of interest in their promoter sequences. Thus, these few TFs are potentially autoregulated. In these autoregulated TFs, the expression correlation between TF and target genes is expected to be higher, as the TF is also among the target promoters. In other cases, the treatment and control experiments are presumed to have reached a steady state or are sufficiently separated by the time that the regulation of the TF and its downstream targets can both be observed in the treatment experiment. Where the time of treatment is too short, there is a risk of not capturing the TF due to the lag between the regulation of TF and the subsequent regulation of targets of that TF. Additionally, TFs that function without themselves being transcriptionally regulated and act entirely through, for example, protein interaction, phosphorylation, or movement into the nucleus will not be captured by this method.

**Figure 5:**
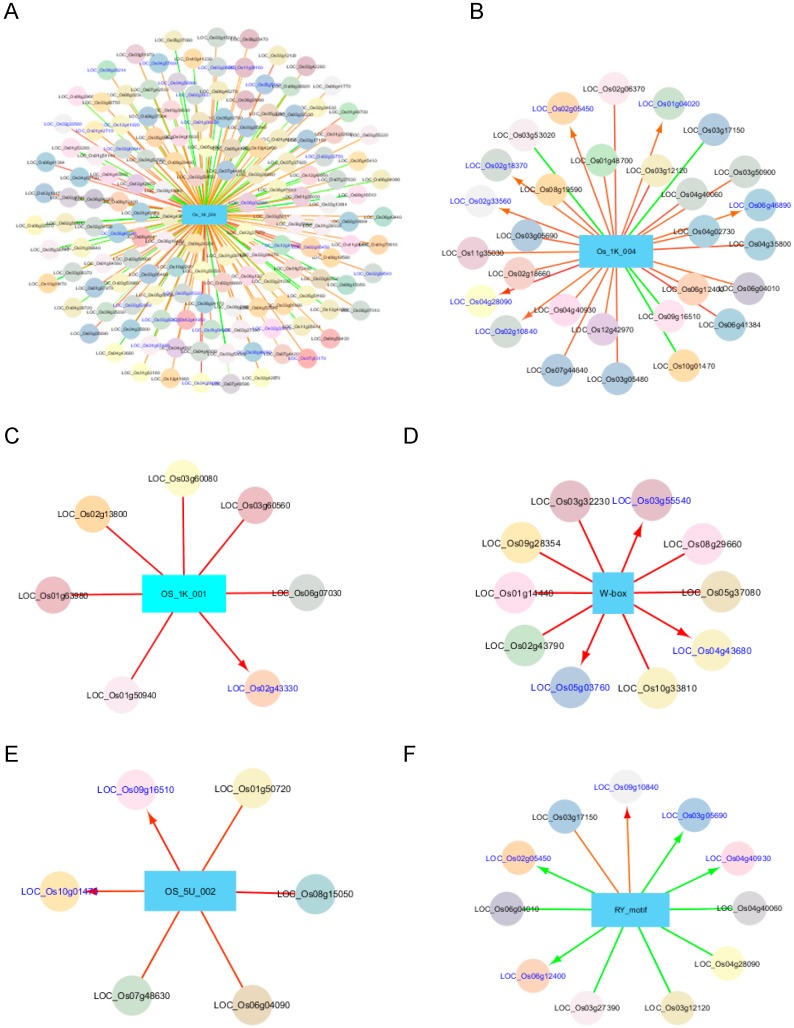
Selected CREs and their associated TFs. (**A**) The significantly associated TFs with OS_1K_004; (**B–F**), the associated TFs (R ≥ |0.45|) with OS_1K_004, OS_1K_001, W-box, OS_5U_002, and RY_motif, respectively. The rectangular shape represents the CRE with its name labeled; circles, the associated TFs with the CRE with the gene name labeled; color of circles, different classes of TFs; red line, positive correlation; green line, negative correlation; thickness of line, the degree of correlation (in proportion to the thickness); arrow head and blue labeling, gene with the CRE in its 1-kb upstream sequence from the transcription start site.

Figure 5A shows all the TFs associated with OS_1K_004, while Figure 5B shows the most associated TFs (r ≥ 0.45 or r ≤ 0.45) to the same CRE. LOC_Os06g12400 (HAZ1), which was found to be positively associated with a few predicted CREs containing GCC-box (0.44 ≤ r ≤ 0.51), is involved in the differentiation of a radial axis in a globular embryo (Ito et al. 2004). In addition, there are other GCC-box associated TFs belonging to different TF families that are also upregulated in embryo, pistil, and callus, including LOC_Os03g06860, LOC_Os02g10840, LOC_Os06g45840, LOC_Os06g41384, and LOC_Os04g28090 based on RNA-seq expression values (Rice Genome Annotation Project; http://rice.plantbiology.msu.edu/). Interestingly, the TFs that were negatively associated with these CREs were found to be upregulated in shoots and seedlings (Rice Genome Annotation Project), coinciding with the regulatory fingerprints of GCC-rich CREs.

We analyzed and compared the TFs that were most likely correlated with the genes enriched with ABRE core elements (i.e., OS_1K_001, G-box, ABRE_M3, ABAVPI, ABRE_M4) in their promoters. The most associated TFs with OS_1K_001 (r ≥ 0.45) were shown in Figure 5C. Two TFs coding genes LOC_Os02g43330 (homolog of ATHB-6) and LOC_Os03g60560 (C2H2) were found to be correlated with all the gene sets bearing the ABRE core tested, except for that of ABAVPI. The findings of this study correspond with the experimental results reported in literature that ATHB-6 is a HD-zip transcription activator that may act as a growth regulator in response to water deficit, by interacting with the core sequence 5′-CAATTATTA-3′ in response to ABA and in an ABI1-dependent manner [48,49]. It was also involved in the negative regulation of the ABA signaling pathway [48]. LOC_Os02g43330 (also known as Oshox24) was shown to correspond to drought or desiccation stress and salt stress by two independent groups using reverse transcription PCR and microarray analyses [50,51]. In addition, Oshox24 was demonstrated to be upregulated during the maturation of panicles [50], corresponding to the regulatory fingerprints of the genes with the promoter enriched with CREs containing the ABRE core. Agarwal et al. [52] also showed that LOC_Os03g60560 (or ZOS3-21) was upregulated by drought and salt, matching the regulatory fingerprints of genes with a promoter enriched with CREs containing the ABRE core.

In addition to that, a few other TFs in the families of NAC, bHLH, and MYB were found to be associated with the gene sets containing some of these ABRE-core containing predicted CREs (Table 5). LOC_Os05g49420 (or OsbZIP45) was reported to be upregulated under stress condition, and had an overlapping expression pattern at one or more stages of seed development [33], matching the expression fingerprints of genes with the promoter-containing CREs predicted in this study (Table 1). LOC_Os05g49420 (G-Box binding protein and bZIP protein) was only found to be positively and moderately correlated with the genes enriched with the ABRE core element in their promoter sequence tested (0.44 ≤ r ≤ 0.50), but was weakly related to genes enriched with ABAVP1 in their promoter sequences (r < 0.27). However, the correlation test does not imply causation; thus, we cannot conclude from this study whether LOC_Os05g49420 binds to the ABRE core sequence in these gene sets. It is noteworthy that there are also other bZIP TFs that were more weakly related to the gene sets tested.

The expression profile of LOC_Os01g64000, the rice ABA-Insensitive 5 (ABI5) homolog, was found to have a stronger relationship to that of ABAVP1 (r = 0.41) compared to that of LOC_Os05g49420 (r = 0.27). The Arabidopsis ABI5 was reported to be responsive to abiotic stresses and sugar [53,54], coinciding with the expression fingerprints of ABAVPI (Table 1).

Among the three TFs that had the highest correlation (0.48 ≤ r ≤ 0.50) to ABAVPI were three TFs in the NAC family (LOC_Os05g34830, LOC_Os07g48550, and LOC_Os11g03300). LOC_Os05g34830 (OsNAC52) was found to respond to ABA and confer drought tolerance in transgenic plants previously [55], and in fact, all three genes were found to be upregulated by salt and cold stresses [56]. It was noteworthy that a few of these TFs mentioned above were also positively and strongly correlated (r ≥ 0.50) with the genes enriched with CE_M6 (MMCGCGTS) in their promoter sequences (Table 5), including LOC_Os01g50940, LOC_Os03g60560, LOC_Os03g60080, and LOC_Os02g43330 (r = 0.49), demonstrating some degree of overlapping of TFs associated with CE and ABRE.

Four TFs, i.e., LOC_Os04g43680 (Myb), LOC_Os03g32230 (C2H2), LOC_Os05g03760 (C3H), and LOC_Os01g14440 (WRKY) were most likely correlated (r ≥ 0.5) with the W-box-containing gene set positively (Table 5, Figure 5D). LOC_Os04g43680 plays a role in cold stress response [57,58,59], including the expression of genes involved in reactive oxygen species (ROS) scavenging, while LOC_Os03g32230 is required for the regulation of the cross-talk between NADPH oxidase, hydrogen peroxide, and mitogen-activated protein (MAP) kinase in ABA signaling, which is important for the tolerance to water stress and oxidative stress [60]. Although none of these TFs were reported to be directly related to biotic stress, they could be related to the scavenging of ROS, which are produced during the defense response of plants to pathogen-associated molecular patterns (PAMPs) such as chitin and lipopolysaccharides [61]. No literature was found on the other TFs. More than 10 WRKY TFs were found to have a moderate to strong positive relationship (0.30 < r < 0.51) to the genes enriched with WRKY in their promoter sequences, implying their involvement in plant biotic response.

Oshox15, which was shown to be closely related to dicot HD-Zip genes CpHB-2, HAT9, and HAT22, was upregulated during the maturation of panicles in addition to its expression in stem, root, sheath, and blade [50]. This supported our finding, which demonstrated its correlation to OS_1D_005 (Figure 5E), a predicted CRE correlated to the upregulation of genes in seeds at 5–10 dap versus 3–4 dap and 11–20 dap, respectively to the roots and shoots versus stigma, respectively.

Figure 5F shows the most associated TFs with the RY-motif. The RY motif, a negative element repressing expression in non-seed tissues, is reported to be responsible for the high-level expression of several seed-specific genes by binding to TFs with the B3 domain [62]. LOC_Os03g12120, LOC_Os03g27390, LOC_Os02g05450, and LOC_Os04g40060 were among the TFs that were negatively associated (i.e., an abundance of the TF is correlated with the downregulation of genes) with RY-motif, with r values ranging between −0.60 and −0.51 in respective order. Little information is available about the TFs that correlate to the RY-motif. It is noteworthy that one of these TFs, LOC_Os03g12120, encodes a TF belonging to the NAM, ATAF and CUC (NAC) family consisting of NAM (No Apical Meristem), ATAF, and CUC (cup-shaped cotyledon) TFs, which share a TFBS (CATGTG; [63]), partially overlapping with the RY-motif (CATGCA). The associated TFs are useful in the reconstruction of a regulatory network, and the subsequent understanding of network properties.

## 3. Materials and Methods 

### 3.1. Rice Gene Expression Data Files

In total, 50 rice gene expression series (GSE) were retrieved from the National Center for Biotechnology Information (NCBI) Gene Expression Omnibus (http://www.ncbi.nlm.nih.gov/geo/). These GSE consist of experiments covering abiotic stresses, biotic stresses, developmental stages, hormone treatments, tissue specificity, sucrose starvation, nitrogen assimilation, and other aspects in rice (Supplementary Table S1). Global scaling normalization was applied to data sets from Affymetrix prior to further analyses. The mean or median of expression values calculated from biological and technical replicates of processed data sets were used for the computing of log_2_ (the ratio of expression signals), which are also known as M-values. A rice gene expression database consisting of 231 M-values for each gene was assembled in Microsoft Excel. The genes were divided into three groups: two-fold induced, two-fold suppressed, and neutral for each array except for a few arrays with low M-values, whereby a cutoff of 1.5-fold was applied.

### 3.2. Identification of CREs in Rice 

The co-expression patterns of rice genes were analyzed using hierarchical clustering and K-means clustering in Clustal 3.0 package (http://rana.lbl.gov/EisenSoftware.htm) and viewed using Java Treeview (http://jtreeview.sourceforge.net/, [64]). The 3-kb and 1-kb upstream sequences from transcriptional start (TSS), 5′-untranslated region (UTR), introns, and 1-kb downstream sequence from translational stop codon (TSC) (including 3′-UTR) of co-expressed rice (*Oryza sativa* ssp. japonica) genes were retrieved using Biomart from Gramene (http://www.gramene.org/), and subjected to the identification of putative CREs using MotifSampler 2.0 (http://homes.esat.kuleuven.ac.be/~thijs/work/MotifSampler.html; [29]). Rice background models were created for 3-kb and 1-kb upstream sequences from TSS, 5′ UTRs, exons, and introns, and 1-kb downstream sequences from TSCs, respectively, for the search of CREs that were between 8–25 nucleotides in length. 

### 3.3. Correlation Analysis of Rice Genes Containing CREs 

Patmatch 1.2 (http://www.arabidopsis.org) was used to retrieve a list of rice sequences that contain at least one copy of known CREs (retrieved from literature) or putative CREs generated by MotifSampler from co-expressed genes in this study. The M-values of rice genes with a CRE were used for correlation analysis, as described by Geisler et al. [18]. The observed and expected numbers of rice genes with a CRE that were induced, suppressed, and neutral in each array were compared, respectively. A CRE was considered to be biologically significant if the genes containing a CRE were significantly more induced or suppressed using a chi-square test (*p* < 0.05). The CRE was considered not biologically significant if the number of genes bearing it was not significantly different from the number of induced or suppressed genes randomly selected from the genome. A high correlation value indicates a high probability that the selected CRE is biologically active. The correlation analysis was also repeated for a list of genes with CRE located at different positions, orientation relative to TSS, and with different copy numbers in the 1-kb upstream sequence. 

### 3.4. Search for Co-Existing Motifs 

The 1-kb upstream rice sequences containing the following CREs—ABAVPI (TACGTGTC), OS_1K_001 (CACGTGTC), ABRE_M4 (CACGTGGC), ABRE (Rice), ABRE_M3, W-box, OS_1K_004 (CGCCGCCG), and OS_5U_002 (AGCTAGCT) were used as input files for the identification of putative co-existing motifs using MotifSampler 2.0 with the 1-kb upstream sequences as a background model. The motifs present in the sequences were ranked by MotifRanking in the MotifSampler Package.

### 3.5. Prediction of Corresponding Trans-Acting Factors

A list of rice genes encoding TFs were obtained from Plant TFDB 4.0 (Jin et al. 2017; http://planttfdb.cbi.pku.edu.cn/). The prediction of TFs was performed by calculating Z scores (Kim and Kim 2006), Z= (X-µ)√n/δ for a list of genes containing a CRE, where X is the mean of the fold-change values of genes having the same predicted CRE; µ is the mean of fold-change values of total genes in a data set and δ is the standard deviation for the fold-change values of total genes in a data set, and n is the size of the gene set. Then, the correlation between Z scores and the fold-change values of a TF among multiple microarray data sets was calculated using the Pearson correlation coefficient. The statistical significance of each correlation was inferred from a t-test with n − 2 degrees of freedom, and a T score = r*√[(n − 2)/(1 − r^2^)], where r is the Pearson correlation coefficient and n is the number of arrays (n > 184, which is more than 80% of the total number of data sets being compared). The TFs that are associated with the CRE were ranked according to r and *p*-values. TFs with r ≥ 0.5 and r ≤ −0.5 were considered to have strong positive and negative correlations to the CRE tested, respectively while TFs with 0.3 < r < 0.5 or −0.5 < r < −0.3 were considered to have moderate correlations; and TFs with −0.3 < r < 0 were considered to have weak correlations to the CRE tested. The CREs and associated TFs were visualized using Cytoscape 3.7.1 ([65]; chianti.ucsd.edu).

## 4. Conclusions

We have adapted and applied algorithms previously used for other model systems in a rice model for the prediction of CREs, the co-existing motifs, and TFBSs. The reliability of our methods in the predictions of biologically significant CREs and their corresponding TFs were supported by previous wet lab experimental data and a literature review. The information generated in this study contributes to the prioritization of candidates for further analysis toward the understanding of the gene regulatory network in japonica rice. The construction of synthetic promoters, which contain a minimal promoter and multiple copies of one or more CREs controlling the expression of a reporter gene, has been found to be useful in testing the function of CREs in vitro [18,66]. In addition, we also identified small cis-regulatory modules with CREs and TFs. Such findings may help in the future to reveal the transcription complexity or expression fingerprints that result from the CREs. The testing conducted in this study also paves the way for the development of automated pipelines and the investment of a high throughput computing resources for a systemic approach to future analyses of CRE and TF in rice subpopulations that are known to have many single nucleotide polymorphisms (SNPs). The understanding on how SNPs in CREs change the regulatory patterns in different rice subpopulations or varieties may prove valuable for crop improvement.

## Figures and Tables

**Table 1:** Known or predicted CREs with abscisic acid responsive element (ABRE) core and coupling element (CE) and their correlation to related experiments.

CRE Name	CRE Sequence	Reference	*p*-Value of Correlation to Selected Microarray			
			GSE6901 Drought/ Control	GSE6901 Salt/Control	GSE5853 Sucrose Starvation 12h/Sorbitol	GSE6893 Panicles P5/ Panicles P4
C−box	G**ACGT**C	Franco-Zorrilla et al. 2014	N	N	N	N
A−box	T**ACGT**A	Franco-Zorrilla et al. 2014	4.77 × 10^−4^ *	1.95 × 10^−5^	N	2.99 × 10^−5^ *
G−box	C**ACGT**G	Franco-Zorrilla et al. 2014	7.84 × 10^−13^	6.15 × 10^−8^	N	1.94 × 10^−7^
ABRE_M1	MNN**ACGT**GKC	This study	4.97 × 10^−11^	4.65 × 10^−8^	N	N
ABRE (MEME)	YKMC**ACGT**GKC	Zhang et al. 2005	1.16 × 10^−10^	1.38 × 10^−12^	N	4.88 × 10^−5^
ABRE_M3	MC**ACGT**GKC	This study	2.59 × 10^−22^	3.43 × 10^−17^	N	9.24 × 10^−8^
CBF3	Y**ACGT**GGC	Oh et al. 2005	7.64 × 10^−10^	4.21 × 10^−10^	N	4.66 × 10^−5^
ABRE_M4	C**ACGT**GGC	This study	6.18 × 10^−13^	2.44 × 10^−11^	N	1.59 × 10^−5^
OS_1K_001	C**ACGTG**TC	This study	1.59 × 10^−14^	2.27 × 10^−8^	4.22 × 10^−1^	N
OS_1K_002	AC**ACGT**GTC	This study	5.67 × 10^−11^	5.79 × 10^−8^	1.10 × 10^−5^	N
ABRE	**ACGT**GKC	Hattori et al. 2002	3.47 × 10^−22^	2.10 × 10^−17^	N	9.11 × 10^−6^
ABRE	**ACGT**GTC	Hattori et al. 2002	1.70 × 10^−20^	3.51 × 10^−13^	2.39 × 10^−8^	1.45 × 10^−4^
ABRE	**ACGT**GGC	Michel et al. 1993	3.00 × 10^−5^	8.41 × 10^−6^	N	N
ABREOSRAB21	**ACGT**SSSC	Marcotte et al. 1989	9.80 × 10^−7^	8.70 × 10^−5^	N	N
ABRE (Arabidopsis)	SRT**ACGT**GTC	Zhang et al. 2005	N	N	3.00 × 10^−15^	9.84 × 10^−6^
ABRE (Monocot)	MGT**ACGT**GKC	Zhang et al. 2005	1.93 × 10^−4^	4.16 × 10^−5^	2.43 × 10^−16^	1.67 × 10^−11^
ABRE (Rice)	CGT**ACGT**GTC	Hobo et al. 1999	N	N	2.07 × 10^−28^	3.21 × 10^−8^
OS_1K_003	CGT**ACG**YG	This study	3.46 × 10^−7^	3.93 × 10^−6^	N	2.84 × 10^−9^
ABA responsive	AGT**ACGT**GGC	Ono et al. 1996	N	N	1.36 × 10^−6^	N
ABAVP1	T**ACGT**GTC	Hattori et al. 1995	1.80 × 10^−4^	N	6.87 × 10^−11^	3.32 × 10^−4^
ABRE	T**ACGT**GC	Hattori et al. 2002	2.44 × 10^−6^	3.25 × 10^−8^	N	4.81 × 10^−6^
GluB−1	GT**ACGT**G	Washida et al. 1999	5.45 × 10^−6^	1.05 × 10^−9^	N	1.50 × 10^−5^
CE3	CA**CGCG**	Maruyama et al. 2012	1.20 × 10^−10^	N	N	9.93 × 10^−11^
CE_M3	**CGCGT**GKC	This study	4.22 × 10^−4^	N	4.25 × 10^−4^	N
CE_M4	**CGCGT**CKC	This study	2.05 × 10^−7^	N	N	1.35 × 10^−6^
CE_M5	**CGCGTS**KC	This study	4.17 × 10^−9^	N	N	2.11 × 10^−5^
CE_M6	**MCGCGTS**	This study	1.05 × 10^−11^	N	N	4.25 × 10^−16^
CE_M2	**MCGCGT**CD	This study	7.55 × 10^−9^	N	N	1.91 × 10^−13^
CE (Arabidopsis)	GA**C**R**CGT**GKC	Zhang et al. 2005	N	N	4.89 × 10^−13^	N
CE	GM**CGCGT**GKC	Zhang et al. 2005	N	N	1.89 × 10^−11^	N
CE (Rice)	GA**CGCGT**GTC	Hobo et al. 1999	N	N	1.36 × 10^−6^	N

N, non-significant correlation (*p* ≥ 0.05); *, *p*-value for all differentially expressed genes in the cDNA microarray experiment; value in gray background, *p*-value for induced genes only; value in white background, *p*-value for suppressed genes only.

**Table 2:** CREs with ABRE core and CE and their correlation to related experiments.

CRE Name	CRE Sequence	Reference	*p*-Value of Correlation to Selected Microarray			
			GSE6908 Anoxic Coleoptiles/ Aerobic Coleoptiles	GSE7951 Stigma/Root	GSE7951 Stigma/ Shoot	GSE2691 Seedling/ Cultured Cell
Dreb1A	R**CCG**ACNT	Maruyama et al. 2012	1.62 × 10^−4^	N	N	N
DRE	R**CCG**AC	Rushton et al. 2002	1.95 × 10^−6^	N	N	N
ERE	A**GCCGCC**	Rushton et al. 2002	3.93 × 10^−11^	N	N	1.03 × 10^−11^
Os_1K_004	C**GCCGCC**G	This study	1.36 × 10^−39^	9.54 × 10^−19^	4.18 × 10^−13^	1.28 × 10^−64^
GCC box	**GCCGCC**	Franco-Zorrilla et al. 2014	4.56 × 10^−31^	6.35 × 10^−10^	2.70 × 10^−12^	1.43 × 10^−47^
RAP2.6	**GCCGC**A	Franco-Zorrilla et al. 2014	1.05 × 10^−17^	4.19 × 10^−6^	1.77 × 10^−5^	2.90 × 10^−11^
RAP2.3	**GCCG**A**C**	Franco-Zorrilla et al. 2014	6.87 × 10^−6^	N	N	N
RRTF1; RAP2.6	**GCCG**T**C**	Franco-Zorrilla et al. 2014	8.06 × 10^−7^	3.76 × 10^−8^	5.69 × 10^−5^	3.50 × 10^−10^
RAP2.6	**GCCG**G**C**	Franco-Zorrilla et al. 2014	4.06 × 10^−6^	1.70 × 10^−4^	N	6.37 × 10^−6^
GCCCA_M1	AG**GCC**CAA	This study	4.17 × 10^−6^	N	N	6.03 × 10^−8^
GCCCA_M2	CG**GCC**CAA	This study	2.77 × 10^−5^	N	N	2.15 × 10^−19^
GCCCA_M3	GG**GCC**CAA	This study	1.35 × 10^−10^	N	N	4.28 × 10^−13^
TCP Clas II	GYG**G**N**C**CC	Franco-Zorrilla et al. 2014	1.68 × 10^−9^	3.90 × 10^−4^	4.89 × 10^−6^	5.42 × 10^−7^
Site IIA	A**GCC**CA	Maruyama et al. 2012	6.47 × 10^−14^	N	N	4.24 × 10^−3^
Site IIA	G**GCC**CA	Maruyama et al. 2012	7.22 × 10^−13^	N	N	2.42 × 10^−30^
Site IIA	TGG**GCC**	Maruyama et al. 2012	2.98 × 10^−13^	N	N	4.32 × 10^−29^
			**GSE4645** **Chitinoligomer Treatment/CEBiP knock - down**	**GSE4645** **Chitinoligomer Treatment/WT**	**GSE5906** **GN8 Treatment 3h/Control**	**GSE5906** **LPS Treatment 3h/Control**
W−box	TTGACC	de Pater et al. 1996	3.19 × 10^−10^	7.21 × 10^−13^	6.13 × 10^−7^	1.60 × 10^−7^
W−box	TTGACY	Franco-Zorrilla et al. 2014	6.22 × 10^−10^	7.77 × 10^−12^	1.18 × 10^−5^	3.04 × 10^−8^

N, non-significant correlation (*p* ≥ 0.05); *, *p*-value for all differentially expressed genes in the cDNA microarray experiment; value in gray background, *p*-value for induced genes only; value in white background, *p*-value for suppressed genes only. P5, panicles at 15–22 cm; P6, panicles at 22–30 cm; GN8, N-acetylchitooligosaccharide; and LPS, lipopolysaccharides.

**Table 3:** CREs with contrasting regulation to CREs.

CRE Name	CRE Sequence	Reference	*p*-Value of Correlation to Selected Microarray			
			GSE6908 Anoxic Coleoptiles/ Aerobic Coleoptiles	GSE7951 Stigma/Root	GSE7951 Stigma/Shoot	GSE2691 Shoot/Cultured Cell
GLK recognition motif	C**CAATC**	Kobayashi et al. 2012	1.60 × 10^−5^	N	N	N
WOX13	**CAATC**A	Franco-Zorrilla et al. 2014	1.49 × 10^−6^	1.58 × 10^−6^	8.82 × 10^−6^	2.32 × 10^−4^
WOX13 secondary motif	**TAATTA**	Franco-Zorrilla et al. 2014	1.21 × 10^−2^	1.17 × 10^−17^	1.35 × 10^−8^	N
OS_1D_002	**TAATTA**AT	This study	N	1.97 × 10^−15^	5.50 × 10^−16^	N
ICU4/ATHB51	**AAT**W**AT**T	Franco-Zorrilla et al. 2014	1.38 × 10^−7^	7.77 × 10^−5^	1.28 × 10^−4^	N
HD−ZIP binding site	**AAT**N**AT**T	Franco-Zorrilla et al. 2014	9.77 × 10^−7^	N	N	N
Yabby binding site	**AAT**N**AT**TA	Franco-Zorrilla et al. 2014	3.13 × 10^−7^	7.51 × 10^−8^	9.72 × 10^−5^	N
Yabby binding site	**AAT**N**AT**AA	Franco-Zorrilla et al. 2014	N	N	N	N
OS_3K_004	G**CTAGCT**N	This study	4.60 × 10^−27^	1.04 × 10^−65^	2.07 × 10^−41^	4.66 × 10^−15^
OS_1U_002	AG**CTAGCT**	This study	1.30 × 10^−66^	1.88 × 10^−39^	6.26 × 10^−47^	9.53 × 10^−16^
SHI/STY	**CTAGCTA**G	Franco-Zorrilla et al. 2014	7.80 × 10^−20^	1.54 × 10^−53^	5.97 × 10^−32^	N
OS_UT_002	**TAGCTA**KN	This study	8.60 × 10^−5^/ 8.49 × 10^−15^	7.49 × 10^−5^	N	8.86 × 10^−7^
RY motif	**CATGCA**	Reidt et al. 2000	N	1.70 × 10^−27^	1.35 × 10^−15^	N
OS_1D_003	**CATGCA**TG	This study	N	4.08 × 10^−15^	N	N
PBE−box	CA**CATG**	Franco-Zorrilla et al. 2014	N	9.99 × 10^−5^	N	N
DPBF Core DCDC3	ACA**CATG**	Kim et al. 2002; PLACE	1.80 × 10^−5^ *		N	N
OS_5U_005	N**MTCGATC**	This study	3.02 × 10^−8^/ 4.64 × 10^−61^	2.79 × 10^−4^/ 7.03 × 10^−11^	5.74 × 10^−9^	N
OS_1D_005	**MTCGATC**N	This study	6.22 × 10^−7^	3.87 × 10^−15^	7.19 × 10^−17^	N
OS_5U_004	NCGAWCGM	This study	2.85 × 10^−51^	9.58 × 10^−13^	8.27 × 10^−12^	N
SPL	**CGTAC**	Franco-Zorrilla et al. 2014	3.20 × 10^−9^	2.66 × 10^−7^	1.14 × 10^−10^	4.60 × 10^−5^
SPL	C**CGTAC**	Franco-Zorrilla et al. 2014	2.10 × 10^−4^	N	N	N

N, non-significant correlation (*p* ≥ 0.05); *, *p*-value for all differentially expressed genes in the cDNA microarray experiment; value in gray background, *p*-value for induced genes only; value in white background, *p*-value for suppressed genes only.

**Table 4:** CREs that can be correlated to experiments related to floral and grain development stages.

CRE Name	CRE Sequence	Reference	*p*-Value of Correlation to Selected Microarray									
			GSE6893 Panicles P1/ SAM	GSE6893 Panicles P2/ Panicles P1	GSE6893 Panicles P3/ Panicles P2	GSE6893 Panicles P4 /Panicles P3	GSE6893 Panicles P5/ Panicles P4	GSE6893 Panicles P6/ Panicles P5	GSE6893 Seed 3–4 dap /Seed 0–2 dap	GSE6893 Seed 5–10 dap/Seed 3–4 dap	GSE6893 Seed 11–20 dap/Seed 5–10 dap	GSE6893 Seed 21–29 dap/Seed 11–20 dap
RY motif	**CATGCA**	Franco-Zorrilla et al. 2014	6.42 × 10^−5^	2.34 × 10^−14^	3.60 × 10^−15^	2.83 × 10^−10^/ 3.49 × 10^−6^	3.16 × 10^−10^	2.28 × 10^−9^/ 2.12 × 10^−9^	2.64 × 10^−11^/1.90 × 10^−6^	6.95 × 10^−12^/ 1.72 × 10^−8^	1.46 × 10^−19^	1.35 × 10^−6^/ 1.95 × 10^−11^
OS_1D_003	**CATGCA**TG	This study	N	N	1.19 × 10^−5^	N	4.52 × 10^−5^	3.28 × 10^−6^	4.48 × 10^−4^	1.34 × 10^−5^	1.64 × 10^−10^	6.66 × 10^−5^
PBE-box	**CACATG**	Franco-Zorrilla et al. 2014	N	4.06 × 10^−5^	N	N	N	N	N	N	N	N
DPBF Core DCDC3	A**CACATG**	Kim et al. 2002; PLACE	2.88 × 10^−4^	2.61 × 10^−4^	8.14 × 10^−6^	2.26 × 10^−4^	N	N	N	N	N	N
OS_5U_005	N**MTCGAT**C	This study	1.32 × 10^−26^	N	N	N	N	1.50 × 10^−28^	N	2.66 × 10^−18^	N	N
OS_1D_005	**MTCGAT**CN	This study	6.63 × 10^−10^	N	N	N	N	1.78 × 10^−9^	N	5.80 × 10^−12^	N	N
OS_5U_004	NCGAWCGM	This study	5.55 × 10^−34^	N	N	N	N	2.71 × 10^−34^	N	2.60 × 10^−22^	N	N

N, non-significant correlation (*p* ≥ 0.05); *, *p*-value for all differentially expressed genes in the cDNA microarray experiment; value in gray background, *p*-value for induced genes only; value in white background, *p*-value for suppressed genes only; dap, day after pollination; P1, panicles at 0–3 cm; P2, panicles at 3–5 cm; P3, panicles at 5–10 cm; P4, panicles at 10–15 cm; P5, panicles at 15–22 cm; P6, panicles at 22–30 cm. Tissues compared to earlier developmental stage or shoot apical meristem (for P1) or seeds 0–2 dap (for seed 3–4 dap).

**Table 5:** Transcription factors (TFs) that are significantly (*p* < 0.05) and highly associated to predicted CREs.

CRE	Pub_Locus of TF	TF Class	Correlation (r)	T Score	*p*-Value
Os_1K_004	LOC_Os04g28090 *	MYB	0.63	12.25	9.87 × 10^−27^
	LOC_Os03g12120	NAC	0.59	10.77	5.06 × 10^−22^
	LOC_Os06g41384	C3H	0.58	9.73	2.57 × 10^−18^
	LOC_Os04g40060	FAR1	0.54	9.56	2.45 × 10^−18^
	LOC_Os04g35800	C3H	0.54	8.99	1.94 × 10^−16^
	LOC_Os02g06370	Whirly	0.54	8.92	3.11 × 10^−16^
	LOC_Os02g33560 *	bZIP	0.51	8.88	2.45 × 10^−16^
	LOC_Os06g12400	HB−PHD	0.51	8.50	4.30 × 10^−15^
	LOC_Os03g05480	C2H2	0.51	8.81	3.96 × 10^−16^
	LOC_Os08g19590	HD−ZIP	0.51	8.26	2.24 × 10^−14^
	LOC_Os03g05690	C2H2	0.50	8.48	3.57 × 10^−15^
	LOC_Os02g05450 *	HB−other	0.50	8.16	3.52 × 10^−14^
	LOC_Os07g44640	C2H2	0.50	8.42	5.39 × 10^−15^
OS_1K_001	LOC_Os02g43330 *	HD−ZIP	0.59	10.17	8.75 × 10^−20^
	LOC_Os03g60560	C2H2	0.52	9.03	8.41 × 10^−17^
G−box	LOC_Os03g60080 *	NAC	0.58	9.93	3.98 × 10^−19^
	LOC_Os02g43330 *	HD−ZIP	0.56	9.61	3.56 × 10^−18^
	LOC_Os01g50940	bHLH	0.55	8.95	3.65 × 10^−16^
	LOC_Os01g64360	MYB	0.52	8.40	1.11 × 10^−14^
	LOC_Os03g60560	C2H2	0.52	9.11	4.89 × 10^−17^
ABRE_M4	LOC_Os02g43330	HD−ZIP	0.54	8.97	2.38 × 10^−16^
	LOC_Os01g50940	bHLH	0.53	8.51	5.88 × 10^−15^
	LOC_Os03g60080	NAC	0.52	8.63	1.98 × 10^−15^
ABRE_M3	LOC_Os02g43330 *	HD−ZIP	0.62	11.13	1.26 × 10^−22^
	LOC_Os03g60080	NAC	0.55	9.35	1.90 × 10^−17^
	LOC_Os03g60560	C2H2	0.55	9.80	4.38 × 10^−19^
	LOC_Os01g50940	bHLH	0.54	8.76	1.25 × 10^−15^
	LOC_Os05g37060	MYB	0.50	8.19	2.91 × 10^−14^
	LOC_Os05g49420 *	bZIP	0.50	8.08	6.38 × 10^−14^
ABAVP1	LOC_Os05g34830	NAC	0.50	7.77	5.37 × 10^−13^
	LOC_Os10g17630	B3	−0.50	−8.30	1.31 × 10^−14^
CE_M5	LOC_Os01g64360 *	MYB	0.67	12.43	3.29 × 10^−26^
	LOC_Os01g50940 *	bHLH	0.66	11.82	2.18 × 10^−24^
	LOC_Os07g36170	GRAS	0.64	12.42	2.85 × 10^−27^
	LOC_Os03g60560	C2H2	0.62	11.75	4.18 × 10^−25^
	LOC_Os02g26430	WRKY	0.60	10.64	3.37 × 10^−21^
	LOC_Os03g12370	HSF	0.59	11.02	6.31 × 10^−23^
	LOC_Os06g44010	WRKY	0.58	10.01	2.73 × 10^−19^
	LOC_Os02g52670	ERF	0.57	10.36	9.37 × 10^−21^
	LOC_Os03g60080	NAC	0.57	9.79	1.04 × 10^−18^
	LOC_Os01g63980 *	C2H2	0.57	10.19	3.33 × 10^−20^
	LOC_Os09g32040	NAC	0.56	9.61	3.5 × 10^−18^
	LOC_Os05g41780	ERF	0.55	9.84	3.25 × 10^−19^
	LOC_Os01g07120	ERF	0.55	9.39	1.26 × 10^−17^
	LOC_Os03g02160	C3H	0.53	9.45	4.7 × 10^−18^
	LOC_Os03g32230 *	C2H2	0.53	9.37	8.4 × 10^−18^
	LOC_Os01g15640	NAC	0.53	9.08	7.76 × 10^−17^
	LOC_Os07g39470	GRAS	0.53	9.11	6.17 × 10^−17^
	LOC_Os05g07120	bHLH	0.53	8.92	2.38 × 10^−16^
	LOC_Os01g58420	ERF	0.52	9.22	2.23 × 10^−17^
	LOC_Os04g43680	MYB	0.52	9.27	1.49 × 10^−17^
	LOC_Os02g43790	ERF	0.52	8.51	4.4 × 10^−15^
	LOC_Os03g55080 *	WRKY	0.51	8.91	1.83 × 10^−16^
	LOC_Os07g07974 *	CPP	0.51	8.32	1.45 × 10^−14^
	LOC_Os05g49420 *	bZIP	0.50	8.13	4.68 × 10^−14^
W−box	LOC_Os04g43680 *	MYB	0.58	10.62	1.18 × 10^−21^
	LOC_Os03g32230	C2H2	0.51	8.95	1.42 × 10^−16^
	LOC_Os05g03760 *	C3H	0.51	8.29	1.63 × 10^−14^
	LOC_Os01g14440	WRKY	0.51	8.74	5.68 × 10^−16^
OS_5U_002	LOC_Os08g15050	CO−like	0.57	9.67	2.21 × 10^−18^
	LOC_Os09g16510 *	WRKY	0.51	8.38	9.74 × 10^−15^
RY_motif	LOC_Os04g40060	FAR1	−0.51	−8.82	3.53 × 10^−16^
	LOC_Os02g05450 *	HB−other	−0.52	−8.63	1.86 × 10^−15^
	LOC_Os03g27390	bHLH	−0.53	−9.37	8.93 × 10^−18^
	LOC_Os03g12120	NAC	−0.60	−11.26	1.48 × 10^−23^

Significance at *p* < 0.05, correlation r ≥ 0.50 or r ≤ –0.50. * indicates that the presence of CRE in the 1-kb upstream promoter sequence of TFs.

## Supplementary Materials

The following are available online at https://www.mdpi.com/2223-7747/8/11/441/s1, Supplementary Table S1. Rice gene expression series (GSE) used for the identification of co-expressed genes and statistical correlation of CREs. Supplementary Table S2. TFs significantly associated to CREs (*p* < 0.05).
