# Supplementary material for: Genome-Wide Computational Identification of Biologically Significant Cis-Regulatory Elements and Associated Transcription Factors from Rice
Source: Plants (Basel). 2019 Oct 23;8(11):441. doi: 10.3390/plants8110441 (PMC6918188; doi:10.3390/plants8110441)
Supplement: Supplementary file 1 [file plants-08-00441-s001.pdf]

**Supplementary Table S1. Rice gene expression series (GSE) used for the identification of co-expressed genes and statistical correlation of CREs.**

| GSE No. | Type of Platform | Experiment | Pubmed ID |
|---|---|---|---|
| GSE10857 | Affymetrix | Gene expression of rice root tips before, at and buckled by a hard layer in two rice varieties | None |
| GSE10872 | Affymetrix | Molecular characterization and genetic analysis reveal SA0420 as an early senescing rice mutant with pleotropic | None |
| GSE12069 | Affymetrix | Microarray analyses reveal that plant mutagenesis may induce more transcriptomic changes than transgene insertion | 18303117 |
| GSE3053 | Affymetrix | Rice salt expression | 16183841 |
| GSE4438 | Affymetrix | Expression data from rice under salinity stress | 17160619 |
| GSE4471 | Affymetrix | Expression data from rice varieties Azucena and Bala grown in 0 and 1ppm arsenate | None |
| GSE6719 | Affymetrix | Cytokinin responsive genes in rice | 17293362 |
| GSE6720 | Affymetrix | Gene expression in OsRR6-overexpression line | 17293362 |
| GSE6893 | Affymetrix | Expression data for reproductive development in rice | 17293439 |
| GSE6901 | Affymetrix | Expression data for stress treatment in rice seedlings | 17293439 |
| GSE6908 | Affymetrix | Transcript Profiling of the Aerobic and Anoxic Rice Coleoptile | 17369434 |
| GSE7256 | Affymetrix | Identification of rice genes differentially expressed upon virulent infection by Magnaporthe grisea | 17905473 |
| GSE7951 | Affymetrix | Genome-wide gene expression profiling of rice stigma | 17556504 |
| GSE8380 | Affymetrix | Genome Wide Identification of Signaling Pathways via OsWRKY13 Transcription Factor in Rice | None |
| GSE9498 | Affymetrix | Global gene expression profiles of Oryza sativa wild type Zhonghua11 and mutant gif1 in filling stage | None |
| GSE10098 | Agilent 22k | Identification of downstream genes regulated by RFL | 18305171 |
| GSE11021 | Agilent 22k | Gene Expression in Response to excess Copper Stress in Rice Leaves | 18676621 |
| GSE11157 | Agilent 22k | Microarray analysis of rice plants fumigated with ozone | 18517257 |
| GSE11158 | Agilent 22k | Microarray analysis of rice plants exposed to jasmonic acid (JA) | 17711315 |
| GSE4409 | Agilent 22k | Dissecting the rice genes responsible for long time changes of nitrogen supply forms and nitrogen starvation | None |
| GSE5286 | Agilent 22k | Rice plants infected by Rice dwarf virus | 17378427 |
| GSE5853 | Agilent 22k | Transcriptomic adaptations in rice suspension cells under sucrose starvation | 17115300 |
| GSE5906 | Agilent 22k | Bacterial lipopolysaccharides induce defense responses associated with Programmed cell death in rice cell | 17018557 |
| GSE6124 | Agilent 22k | Comparative gene expression analysis between virus-susceptible and -resistant rice lines (TW16-4 and -69) | None |
| GSE6125 | Agilent 22k | Comparative gene expression analysis between virus-susceptible and -resistant rice lines (TW16-1263 and -1029) | None |
| GSE6126 | Agilent 22k | Comparative gene expression analysis between virus-susceptible and -resistant rice lines (NIL37 and NIL22) | None |
| GSE6244 | Agilent 22k | Transcriptional profiling of indica rice cultivar IET8585 (Ajaya) infected with bacterial leaf blight pathogen (Xoo) | 17870590 |
| GSE6362 | Agilent 22k | Transcriptional profiling of indica rice cultivar FR13A under submergence treatment. | 18182856 |

| | | | |
|---|---|---|---|
| GSE6600 | Agilent 22k | Transcriptional profiling of indica rice cultivar FR13A under salt stress condition. | None |
| GSE7531 | Agilent 22k | Differentially expressed genes from different developmantal stages in rice | None |
| GSE7532 | Agilent 22k | Differentially expressed genes under different stress conditions in rice | None |
| GSE8811 | Agilent 22k | Rice plants infested with planthoppers | None |
| GSE9450 | Agilent 22k | Rice plants infected by Rice blast fungus | None |
| GSE9765 | Agilent 22k | Microarray analysis of rice plants irradiated with carbon ion beams (HIMAC) | 18414000 |
| GSE661 | Agilent 22k | ABA or GA calli treatment | 14982972 |
| GSE2415 | Agilent 22k | Rice Seedling Stress treatment | None |
| GSE4645 | Agilent 22k | The function of CEBiP in the chitin elicitor signaling in rice cells | 16829581 |
| GSE7071 | de los Reyes Lab Rice cDNA array | Reactive oxygen species trigger a regulatory module involved in the early responses of rice seedlings to cold stress | 17577400 |
| GSE6552 | LIMA_SlideA and B | Transcriptome associates with rice organogenesis | 17072560 |
| GSE2691 | LIMA_SlideA and B | Rice Transcriptome - Slide A; Slide B | 16140994 |
| GSE11712 | LIMA_SlideA and B | Gene expression level of six rice lineages | None |
| GSE11896 | Oligonucleotide array from Plant Sciences Institute at Iowa State University | Global Transcriptome Profiles in the Nodes of Rice During Spatial and Temporal Development | None |
| GSE2360 | Rice Genome Oligo Set V1.0 | Comparison analysis of rice and Arabidopsis photomorphogenesis | 16284311 |
| GSE6533 | Rice Genome Oligo Set V1.0 | Genome-wide Expression Profiles of Drought and High-salinity Stresses-responsive Genes in Rice (Oryza Sativa L. Indica) | 17225073 |
| GSE2619 | Rice Genome Oligo Set V1.0 | Udt1-1 anther and Wild type_anther | 16141453 |
| GSE2211 | Rice Genome Oligo Set V1.0 | Abiotic stress | 15834008 |
| GSE9653 | Rice Oligo array version 2 | Dissect Rice Defense Response Pathways | None |
| GSE7374 | Whole rice locus 44K array | Validation of gene expression from rice locus (dye-swap experiment) | 18043742 |
| GSE7567 | Rice44K | BTH-responsive rice genes | 17601827 |
| GSE11451 | Rice44K | OsGLK1 regulated genes in rice | None |

## Supplementary Table S2. TFs significantly associated to CREs (P<0.05)

| CRE | pub_locus of TF | TF Class | Correlation ( r) | T score | P-value |
|---|---|---|---|---|---|
| OS_1K_001 | LOC_Os02g43330 | HD-ZIP | 0.59 | 10.17 | 8.75E-20 |
| | LOC_Os03g60560 | C2H2 | 0.52 | 9.03 | 8.41E-17 |
| | LOC_Os02g13800 | HSF | 0.48 | 7.63 | 1.06E-12 |
| | LOC_Os01g63980 | C2H2 | 0.47 | 7.93 | 1.13E-13 |
| | LOC_Os03g60080 | NAC | 0.46 | 7.30 | 6.89E-12 |
| | LOC_Os01g50940 | bHLH | 0.45 | 6.89 | 8.27E-11 |
| | LOC_Os06g07030 | ERF | 0.45 | 6.88 | 8.96E-11 |
| | LOC_Os05g49420 | bZIP | 0.44 | 6.91 | 6.57E-11 |
| | LOC_Os05g37060 | MYB | 0.44 | 6.95 | 4.93E-11 |
| | LOC_Os05g10670 | C3H | 0.44 | 6.82 | 1.1E-10 |
| | LOC_Os01g64360 | MYB | 0.43 | 6.57 | 4.83E-10 |
| | LOC_Os10g41200 | MYB_related | 0.42 | 6.94 | 4.01E-11 |
| | LOC_Os05g34830 | NAC | 0.41 | 6.15 | 4.66E-09 |
| | LOC_Os05g07120 | bHLH | 0.41 | 6.53 | 4.97E-10 |
| | LOC_Os04g45810 | HD-ZIP | 0.39 | 6.29 | 1.68E-09 |
| | LOC_Os11g03300 | NAC | 0.39 | 5.93 | 1.33E-08 |
| | LOC_Os03g12370 | HSF | 0.39 | 6.32 | 1.36E-09 |
| | LOC_Os01g07120 | ERF | 0.38 | 5.93 | 1.3E-08 |
| | LOC_Os08g06110 | MYB_related | 0.38 | 6.16 | 3.32E-09 |
| | LOC_Os09g28210 | bHLH | 0.38 | 5.91 | 1.42E-08 |
| | LOC_Os02g52670 | ERF | 0.38 | 6.05 | 6.14E-09 |
| | LOC_Os02g32590 | HSF | 0.37 | 5.89 | 1.53E-08 |
| | LOC_Os08g31580 | ERF | 0.37 | 5.50 | 1.27E-07 |
| | LOC_Os01g48320 | Trihelix | 0.37 | 5.95 | 1E-08 |
| | LOC_Os06g43090 | MYB | 0.36 | 5.53 | 1.01E-07 |
| | LOC_Os08g04840 | MYB_related | 0.36 | 5.18 | 5.71E-07 |
| | LOC_Os01g64730 | bZIP | 0.35 | 5.46 | 1.32E-07 |
| | LOC_Os07g48630 | EIL | 0.35 | 5.52 | 9.28E-08 |
| | LOC_Os07g38090 | C3H | 0.34 | 5.41 | 1.68E-07 |
| | LOC_Os09g35790 | HSF | 0.34 | 5.11 | 7.54E-07 |
| | LOC_Os10g41460 | Trihelix | 0.34 | 5.23 | 4.08E-07 |
| | LOC_Os03g31230 | MYB_related | 0.34 | 5.28 | 3.24E-07 |
| | LOC_Os08g36790 | bZIP | 0.34 | 5.04 | 1.06E-06 |
| | LOC_Os02g26430 | WRKY | 0.34 | 5.01 | 1.2E-06 |
| | LOC_Os09g28440 | ERF | 0.33 | 5.00 | 1.27E-06 |
| | LOC_Os03g55080 | WRKY | 0.33 | 5.26 | 3.29E-07 |
| | LOC_Os01g39020 | HSF | 0.33 | 5.02 | 1.1E-06 |
| | LOC_Os07g12340 | NAC | 0.33 | 5.16 | 5.39E-07 |
| | LOC_Os06g44010 | WRKY | 0.33 | 4.80 | 3.09E-06 |
| | LOC_Os07g36170 | GRAS | 0.32 | 5.11 | 7.05E-07 |
| | LOC_Os01g40260 | WRKY | 0.32 | 4.70 | 4.88E-06 |
| | LOC_Os02g36360 | C2H2 | 0.32 | 4.99 | 1.25E-06 |
| | LOC_Os01g58420 | ERF | 0.32 | 5.00 | 1.17E-06 |
| | LOC_Os01g15640 | NAC | 0.32 | 4.82 | 2.73E-06 |
| | LOC_Os04g58020 | MYB_related | 0.31 | 4.80 | 3.06E-06 |
| | LOC_Os01g21120 | ERF | 0.31 | 4.90 | 1.85E-06 |
| | LOC_Os10g41770 | STAT | 0.31 | 4.85 | 2.32E-06 |
| | LOC_Os01g72490 | SRS | 0.31 | 4.62 | 6.91E-06 |
| | LOC_Os04g51320 | Trihelix | 0.31 | 4.86 | 2.24E-06 |
| | LOC_Os04g46440 | ERF | 0.31 | 4.83 | 2.5E-06 |
| | LOC_Os08g15050 | CO-like | 0.31 | 4.52 | 1.05E-05 |
| | LOC_Os03g64260 | ERF | 0.30 | 4.47 | 1.3E-05 |
| | LOC_Os03g02160 | C3H | 0.30 | 4.67 | 5.23E-06 |
| | LOC_Os10g22950 | CAMTA | 0.30 | 4.71 | 4.36E-06 |
| | LOC_Os06g07010 | FAR1 | 0.30 | 4.52 | 1.02E-05 |
| | LOC_Os12g37690 | MYB | 0.30 | 4.46 | 1.36E-05 |
| | LOC_Os04g32620 | ERF | 0.29 | 4.52 | 9.9E-06 |
| | LOC_Os06g45840 | MYB_related | 0.29 | 4.31 | 2.57E-05 |
| | LOC_Os03g21030 | NAC | 0.28 | 4.18 | 4.33E-05 |
| | LOC_Os09g35760 | HD-ZIP | 0.28 | 3.99 | 9.48E-05 |
| | LOC_Os01g09280 | MYB_related | 0.28 | 4.40 | 1.68E-05 |

| | | | | |
|---|---|---|---|---|
| LOC_Os02g43790 | ERF | 0.28 | 4.08 | 6.53E-05 |
| LOC_Os02g52340 | MIKC_MADS | 0.28 | 4.17 | 4.47E-05 |
| LOC_Os04g55520 | ERF | 0.28 | 4.30 | 2.59E-05 |
| LOC_Os08g01090 | B3 | 0.27 | 4.23 | 3.48E-05 |
| LOC_Os10g07080 | C2H2 | 0.27 | 3.95 | 0.000107 |
| LOC_Os10g33810 | MYB | 0.27 | 3.82 | 0.000183 |
| LOC_Os01g54600 | WRKY | 0.27 | 3.98 | 9.69E-05 |
| LOC_Os12g07120 | GATA | 0.27 | 4.14 | 4.86E-05 |
| LOC_Os01g01870 | bHLH | 0.27 | 4.15 | 4.74E-05 |
| LOC_Os08g36920 | ERF | 0.27 | 3.88 | 0.000143 |
| LOC_Os09g28310 | bZIP | 0.26 | 4.09 | 6.09E-05 |
| LOC_Os05g41780 | ERF | 0.26 | 4.06 | 6.78E-05 |
| LOC_Os02g44120 | C2H2 | 0.26 | 3.76 | 0.000221 |
| LOC_Os03g32230 | C2H2 | 0.26 | 3.98 | 9.28E-05 |
| LOC_Os07g39470 | GRAS | 0.26 | 3.88 | 0.00014 |
| LOC_Os11g06170 | bZIP | 0.26 | 3.73 | 0.000251 |
| LOC_Os03g55590 | G2-like | 0.26 | 3.99 | 8.79E-05 |
| LOC_Os01g70310 | bHLH | 0.25 | 3.95 | 0.000103 |
| LOC_Os07g48570 | Dof | 0.25 | 3.90 | 0.000126 |
| LOC_Os07g07974 | CPP | 0.25 | 3.64 | 0.00035 |
| LOC_Os05g41450 | NF-YC | 0.25 | 3.60 | 0.000404 |
| LOC_Os08g38210 | bHLH | 0.25 | 3.84 | 0.000162 |
| LOC_Os04g53540 | HD-ZIP | 0.25 | 3.57 | 0.000451 |
| LOC_Os03g20780 | EIL | 0.24 | 3.54 | 0.000496 |
| LOC_Os01g14440 | WRKY | 0.24 | 3.76 | 0.000216 |
| LOC_Os03g48970 | NF-YA | 0.24 | 3.74 | 0.000236 |
| LOC_Os10g41230 | HD-ZIP | 0.24 | 3.39 | 0.000848 |
| LOC_Os09g31400 | EIL | 0.24 | 3.49 | 0.000594 |
| LOC_Os01g54550 | HSF | 0.24 | 3.70 | 0.000272 |
| LOC_Os09g32040 | NAC | 0.24 | 3.49 | 0.000604 |
| LOC_Os01g67480 | bHLH | 0.24 | 3.48 | 0.000612 |
| LOC_Os03g51330 | GRAS | 0.23 | 3.61 | 0.000381 |
| LOC_Os03g06630 | HSF | 0.23 | 3.54 | 0.000494 |
| LOC_Os04g57600 | C3H | 0.23 | 3.57 | 0.000435 |
| LOC_Os05g41760 | ERF | 0.23 | 3.52 | 0.000522 |
| LOC_Os02g08150 | CO-like | 0.23 | 3.36 | 0.00095 |
| LOC_Os07g39480 | WRKY | 0.23 | 3.55 | 0.00047 |
| LOC_Os02g05450 | HB-other | 0.23 | 3.31 | 0.001098 |
| LOC_Os09g01960 | MYB | 0.23 | 3.25 | 0.001358 |
| LOC_Os06g12400 | HB-PHD | 0.22 | 3.28 | 0.001224 |
| LOC_Os04g58190 | Dof | 0.22 | 3.41 | 0.000759 |
| LOC_Os03g12350 | ARR-B | 0.22 | 3.32 | 0.001066 |
| LOC_Os09g12770 | G2-like | 0.22 | 3.26 | 0.001315 |
| LOC_Os02g35770 | HD-ZIP | 0.22 | 3.37 | 0.000879 |
| LOC_Os09g38570 | Trihelix | 0.22 | 3.22 | 0.001488 |
| LOC_Os04g41560 | DBB | 0.22 | 3.11 | 0.002196 |
| LOC_Os03g26210 | bHLH | 0.22 | 3.38 | 0.000864 |
| LOC_Os01g09620 | C3H | 0.22 | 3.17 | 0.001774 |
| LOC_Os05g37190 | C2H2 | 0.22 | 3.34 | 0.000985 |
| LOC_Os03g15010 | FAR1 | 0.22 | 3.32 | 0.001039 |
| LOC_Os03g58160 | HSF | 0.21 | 3.31 | 0.001069 |
| LOC_Os01g68370 | B3 | 0.21 | 3.26 | 0.00129 |
| LOC_Os01g61080 | WRKY | 0.21 | 3.24 | 0.001371 |
| LOC_Os06g45890 | G2-like | 0.21 | 3.05 | 0.002623 |
| LOC_Os01g64000 | bZIP | 0.21 | 3.17 | 0.001734 |
| LOC_Os07g22730 | ERF | 0.21 | 2.97 | 0.003409 |
| LOC_Os03g06860 | FAR1 | 0.21 | 3.17 | 0.001737 |
| LOC_Os01g70270 | ARF | 0.21 | 3.19 | 0.001617 |
| LOC_Os03g09100 | CAMTA | 0.21 | 3.18 | 0.001708 |
| LOC_Os04g58000 | B3 | 0.21 | 3.14 | 0.001893 |
| LOC_Os07g48550 | NAC | 0.21 | 3.16 | 0.001803 |
| LOC_Os01g13540 | Nin-like | 0.21 | 3.15 | 0.001851 |
| LOC_Os01g13460 | bHLH | 0.21 | 3.05 | 0.002551 |
| LOC_Os12g21700 | C3H | 0.21 | 3.03 | 0.002716 |
| LOC_Os09g29930 | bHLH | 0.21 | 2.84 | 0.005076 |

| | | | | | |
|---|---|---|---|---|---|
| | LOC_Os05g27930 | ERF | 0.20 | 2.83 | 0.005094 |
| | LOC_Os01g32770 | LBD | 0.20 | 3.06 | 0.002524 |
| | LOC_Os01g65900 | GRAS | 0.20 | 3.05 | 0.002577 |
| | LOC_Os12g10540 | MIKC_MADS | 0.20 | 2.94 | 0.003695 |
| | LOC_Os04g21950 | WRKY | 0.20 | 2.87 | 0.004553 |
| | LOC_Os02g45450 | ERF | 0.20 | 2.79 | 0.005893 |
| | LOC_Os01g10504 | MIKC_MADS | 0.20 | 2.78 | 0.006 |
| | LOC_Os03g53960 | MYB_related | 0.20 | 2.97 | 0.003337 |
| | LOC_Os05g29810 | ERF | 0.20 | 2.72 | 0.007227 |
| | LOC_Os05g09020 | WRKY | 0.19 | 2.71 | 0.007433 |
| | LOC_Os12g40570 | WRKY | 0.19 | 2.93 | 0.003783 |
| | LOC_Os04g43680 | MYB | 0.19 | 2.95 | 0.003548 |
| | LOC_Os11g02480 | WRKY | 0.19 | 2.68 | 0.008107 |
| | LOC_Os01g15900 | Dof | 0.19 | 2.83 | 0.005148 |
| | LOC_Os03g53020 | bHLH | 0.19 | 2.88 | 0.004312 |
| | LOC_Os03g08490 | ERF | 0.19 | 2.72 | 0.007104 |
| | LOC_Os08g14400 | WOX | 0.19 | 2.88 | 0.004416 |
| | LOC_Os07g39310 | C2H2 | 0.19 | 2.70 | 0.00756 |
| | LOC_Os12g41210 | CPP | 0.19 | 2.86 | 0.0046 |
| | LOC_Os02g05510 | GATA | 0.18 | 2.82 | 0.005183 |
| | LOC_Os09g28354 | HSF | 0.18 | 2.62 | 0.009488 |
| | LOC_Os05g45020 | C3H | 0.18 | 2.59 | 0.010259 |
| | LOC_Os03g43840 | LSD | 0.18 | 2.69 | 0.007717 |
| | LOC_Os11g06410 | Trihelix | 0.18 | 2.60 | 0.01006 |
| | LOC_Os09g35880 | DBB | 0.18 | 2.58 | 0.010698 |
| | LOC_Os01g14870 | C3H | 0.18 | 2.57 | 0.010884 |
| | LOC_Os03g42290 | B3 | 0.18 | 2.70 | 0.007539 |
| | LOC_Os08g17400 | WRKY | 0.18 | 2.68 | 0.007832 |
| | LOC_Os03g21240 | G2-like | 0.18 | 2.71 | 0.007293 |
| | LOC_Os09g25060 | WRKY | 0.17 | 2.50 | 0.013073 |
| | LOC_Os09g35030 | ERF | 0.17 | 2.48 | 0.013929 |
| | LOC_Os07g48870 | MYB | 0.17 | 2.63 | 0.009066 |
| | LOC_Os03g21710 | WRKY | 0.17 | 2.50 | 0.013112 |
| | LOC_Os03g54160 | MIKC_MADS | 0.17 | 2.44 | 0.015732 |
| | LOC_Os03g60120 | ERF | 0.17 | 2.61 | 0.009637 |
| | LOC_Os03g17810 | LBD | 0.17 | 2.44 | 0.015574 |
| | LOC_Os03g55540 | C2H2 | 0.17 | 2.61 | 0.009771 |
| | LOC_Os02g40530 | MYB | 0.17 | 2.58 | 0.010593 |
| | LOC_Os05g37080 | NAC | 0.17 | 2.55 | 0.011341 |
| | LOC_Os03g05690 | C2H2 | 0.17 | 2.55 | 0.011382 |
| | LOC_Os05g03760 | C3H | 0.17 | 2.43 | 0.016006 |
| | LOC_Os04g42020 | CO-like | 0.17 | 2.43 | 0.016066 |
| | LOC_Os04g51560 | WRKY | 0.17 | 2.37 | 0.018947 |
| | LOC_Os09g13940 | ERF | 0.16 | 2.35 | 0.019685 |
| | LOC_Os06g11970 | M-type_MADS | 0.16 | 2.34 | 0.020455 |
| | LOC_Os07g35870 | bHLH | 0.16 | 2.49 | 0.013596 |
| | LOC_Os03g11614 | MIKC_MADS | 0.16 | 2.25 | 0.025916 |
| | LOC_Os08g39980 | MYB_related | 0.16 | 2.46 | 0.014752 |
| | LOC_Os05g45410 | HSF | 0.16 | 2.25 | 0.025358 |
| | LOC_Os04g42950 | MYB | 0.16 | 2.32 | 0.02136 |
| | LOC_Os02g45480 | C3H | 0.16 | 2.27 | 0.024414 |
| | LOC_Os01g04020 | ERF | 0.16 | 2.40 | 0.017438 |
| | LOC_Os06g41770 | bZIP | 0.16 | 2.31 | 0.021642 |
| | LOC_Os10g22430 | GRAS | 0.16 | 2.44 | 0.015323 |
| | LOC_Os02g58670 | bZIP | 0.16 | 2.43 | 0.015887 |
| | LOC_Os02g49986 | MYB | 0.16 | 2.20 | 0.02905 |
| | LOC_Os07g37630 | FAR1 | 0.16 | 2.36 | 0.01917 |
| | LOC_Os01g34060 | MYB_related | 0.16 | 2.24 | 0.02588 |
| | LOC_Os03g08500 | ERF | 0.16 | 2.23 | 0.026705 |
| | LOC_Os11g29870 | WRKY | 0.16 | 2.22 | 0.027365 |
| | LOC_Os08g04390 | bHLH | 0.16 | 2.30 | 0.022294 |
| | LOC_Os02g07170 | G2-like | 0.16 | 2.18 | 0.03037 |
| | LOC_Os02g42950 | YABBY | 0.15 | 2.19 | 0.029829 |
| | LOC_Os09g24820 | ZF-HD | 0.15 | 2.13 | 0.034353 |
| | LOC_Os02g57650 | NAC | 0.15 | 2.20 | 0.029052 |

| | | | | |
|---|---|---|---|---|
| LOC_Os06g14190 | NF-X1 | 0.15 | 2.18 | 0.030204 |
| LOC_Os03g10210 | HD-ZIP | 0.15 | 2.17 | 0.031103 |
| LOC_Os10g27360 | NAC | 0.15 | 2.27 | 0.023929 |
| LOC_Os04g40140 | NAC | 0.15 | 2.16 | 0.032033 |
| LOC_Os09g30400 | WRKY | 0.15 | 2.16 | 0.032066 |
| LOC_Os07g48820 | bZIP | 0.15 | 2.17 | 0.031457 |
| LOC_Os04g49450 | MYB_related | 0.15 | 2.25 | 0.025363 |
| LOC_Os01g62660 | G2-like | 0.15 | 2.13 | 0.03416 |
| LOC_Os11g39000 | bHLH | 0.15 | 2.21 | 0.028269 |
| LOC_Os04g59430 | ARF | 0.15 | 2.24 | 0.025813 |
| LOC_Os01g53650 | C3H | 0.15 | 2.09 | 0.037527 |
| LOC_Os04g47990 | Dof | 0.15 | 2.24 | 0.026318 |
| LOC_Os08g06140 | NAC | 0.15 | 2.19 | 0.029729 |
| LOC_Os02g10840 | FAR1 | 0.15 | 2.24 | 0.026074 |
| LOC_Os01g51690 | WRKY | 0.15 | 2.14 | 0.033767 |
| LOC_Os08g28214 | CPP | 0.15 | 2.05 | 0.041851 |
| LOC_Os01g55340 | Dof | 0.15 | 2.07 | 0.039495 |
| LOC_Os03g05480 | C2H2 | 0.14 | 2.17 | 0.031326 |
| LOC_Os11g03370 | NAC | 0.14 | 2.17 | 0.030753 |
| LOC_Os10g30719 | MYB_related | 0.14 | 2.02 | 0.044714 |
| LOC_Os08g04170 | C3H | 0.14 | 2.17 | 0.031322 |
| LOC_Os12g06380 | FAR1 | 0.14 | 2.14 | 0.033834 |
| LOC_Os01g43650 | WRKY | 0.14 | 2.12 | 0.034851 |
| LOC_Os07g37920 | NAC | 0.14 | 2.05 | 0.041574 |
| LOC_Os12g13170 | bZIP | 0.14 | 2.10 | 0.036613 |
| LOC_Os07g04700 | MYB | 0.14 | 2.10 | 0.036803 |
| LOC_Os02g51670 | ERF | 0.14 | 2.12 | 0.035158 |
| LOC_Os04g55970 | AP2 | 0.14 | 2.04 | 0.042907 |
| LOC_Os03g63750 | HSF | 0.13 | 2.02 | 0.044104 |
| LOC_Os04g46860 | GRAS | 0.13 | 2.01 | 0.045416 |
| LOC_Os07g01820 | MIKC_MADS | 0.13 | 2.00 | 0.046982 |
| LOC_Os01g60270 | WOX | 0.13 | 2.01 | 0.045283 |
| LOC_Os01g47560 | WRKY | 0.13 | 1.98 | 0.049449 |
| LOC_Os06g04010 | BBR-BPC | -0.13 | -2.01 | 0.045988 |
| LOC_Os03g55760 | G2-like | -0.13 | -1.97 | 0.049685 |
| LOC_Os02g40070 | AP2 | -0.14 | -2.05 | 0.041512 |
| LOC_Os02g15340 | NAC | -0.14 | -2.09 | 0.037886 |
| LOC_Os06g02230 | B3 | -0.14 | -2.13 | 0.033918 |
| LOC_Os03g55220 | bHLH | -0.15 | -2.19 | 0.029453 |
| LOC_Os12g41860 | HD-ZIP | -0.15 | -2.22 | 0.027441 |
| LOC_Os01g57650 | C2H2 | -0.15 | -2.12 | 0.035107 |
| LOC_Os09g12380 | NAC | -0.15 | -2.08 | 0.038646 |
| LOC_Os06g08440 | ARR-B | -0.15 | -2.10 | 0.036782 |
| LOC_Os06g49040 | G2-like | -0.15 | -2.13 | 0.034112 |
| LOC_Os06g01480 | NAC | -0.15 | -2.28 | 0.023397 |
| LOC_Os07g44640 | C2H2 | -0.16 | -2.32 | 0.021315 |
| LOC_Os02g12790 | GATA | -0.16 | -2.29 | 0.022908 |
| LOC_Os03g05500 | LBD | -0.16 | -2.27 | 0.024585 |
| LOC_Os06g04850 | HD-ZIP | -0.16 | -2.30 | 0.022391 |
| LOC_Os06g46270 | NAC | -0.16 | -2.32 | 0.021426 |
| LOC_Os03g07450 | HD-ZIP | -0.16 | -2.22 | 0.027714 |
| LOC_Os01g08160 | G2-like | -0.16 | -2.46 | 0.014678 |
| LOC_Os12g41230 | CPP | -0.16 | -2.46 | 0.014632 |
| LOC_Os05g11070 | bHLH | -0.17 | -2.34 | 0.020378 |
| LOC_Os09g10840 | bZIP | -0.17 | -2.34 | 0.020352 |
| LOC_Os12g43950 | TALE | -0.17 | -2.47 | 0.014134 |
| LOC_Os04g54474 | bZIP | -0.17 | -2.38 | 0.018185 |
| LOC_Os01g60640 | WRKY | -0.17 | -2.41 | 0.016961 |
| LOC_Os08g09900 | WRKY | -0.17 | -2.54 | 0.011809 |
| LOC_Os06g09717 | ERF | -0.17 | -2.41 | 0.016784 |
| LOC_Os03g15440 | bHLH | -0.17 | -2.61 | 0.009539 |
| LOC_Os11g06840 | C2H2 | -0.17 | -2.59 | 0.010183 |
| LOC_Os01g53040 | WRKY | -0.17 | -2.62 | 0.00933 |
| LOC_Os12g39330 | ERF | -0.17 | -2.40 | 0.017162 |
| LOC_Os07g48596 | G2-like | -0.17 | -2.40 | 0.017212 |

| | | | | | |
|---|---|---|---|---|---|
| | LOC_Os05g03884 | TALE | -0.18 | -2.52 | 0.012474 |
| | LOC_Os03g42420 | B3 | -0.18 | -2.54 | 0.011931 |
| | LOC_Os03g46860 | bHLH | -0.18 | -2.56 | 0.011105 |
| | LOC_Os09g13570 | bZIP | -0.18 | -2.56 | 0.011064 |
| | LOC_Os07g48260 | WRKY | -0.18 | -2.65 | 0.00867 |
| | LOC_Os01g74410 | MYB | -0.18 | -2.73 | 0.006819 |
| | LOC_Os08g02300 | NAC | -0.18 | -2.70 | 0.007441 |
| | LOC_Os08g41940 | SBP | -0.18 | -2.73 | 0.006757 |
| | LOC_Os01g03720 | MYB | -0.18 | -2.73 | 0.006804 |
| | LOC_Os01g11350 | bZIP | -0.18 | -2.78 | 0.005958 |
| | LOC_Os11g05740 | B3 | -0.19 | -2.80 | 0.005598 |
| | LOC_Os08g33750 | G2-like | -0.19 | -2.80 | 0.00565 |
| | LOC_Os07g03770 | TALE | -0.19 | -2.64 | 0.00889 |
| | LOC_Os05g38120 | TALE | -0.19 | -2.73 | 0.006919 |
| | LOC_Os12g37970 | MYB | -0.19 | -2.96 | 0.003435 |
| | LOC_Os05g48850 | NAC | -0.20 | -2.80 | 0.005623 |
| | LOC_Os04g43560 | NAC | -0.20 | -2.82 | 0.005367 |
| | LOC_Os09g33490 | NAC | -0.20 | -2.87 | 0.004531 |
| | LOC_Os01g63460 | MYB | -0.20 | -3.04 | 0.002695 |
| | LOC_Os04g36590 | FAR1 | -0.20 | -2.91 | 0.004015 |
| | LOC_Os05g02420 | MYB_related | -0.20 | -2.99 | 0.003146 |
| | LOC_Os02g46560 | bHLH | -0.21 | -3.12 | 0.002077 |
| | LOC_Os10g17630 | B3 | -0.21 | -3.04 | 0.002686 |
| | LOC_Os04g08060 | C2H2 | -0.21 | -2.96 | 0.003408 |
| | LOC_Os11g05480 | bZIP | -0.21 | -2.92 | 0.003967 |
| | LOC_Os02g39140 | bHLH | -0.21 | -3.00 | 0.003018 |
| | LOC_Os06g35140 | G2-like | -0.21 | -3.01 | 0.002917 |
| | LOC_Os05g41240 | G2-like | -0.21 | -3.02 | 0.00283 |
| | LOC_Os01g09850 | C2H2 | -0.21 | -2.99 | 0.003158 |
| | LOC_Os09g29820 | bZIP | -0.22 | -3.09 | 0.002276 |
| | LOC_Os03g04310 | bHLH | -0.22 | -3.29 | 0.001152 |
| | LOC_Os02g44360 | GRAS | -0.23 | -3.36 | 0.000937 |
| | LOC_Os01g36460 | MYB | -0.23 | -3.32 | 0.001062 |
| | LOC_Os05g04820 | MYB | -0.24 | -3.47 | 0.000643 |
| | LOC_Os08g44830 | C2H2 | -0.24 | -3.44 | 0.00071 |
| | LOC_Os05g50080 | C3H | -0.24 | -3.48 | 0.000627 |
| | LOC_Os04g35010 | bHLH | -0.24 | -3.54 | 0.000505 |
| | LOC_Os01g53260 | WRKY | -0.24 | -3.48 | 0.000619 |
| | LOC_Os07g39800 | HRT-like | -0.24 | -3.76 | 0.000214 |
| | LOC_Os02g52190 | bHLH | -0.24 | -3.40 | 0.000831 |
| | LOC_Os04g50770 | MYB | -0.25 | -3.67 | 0.000314 |
| | LOC_Os04g50920 | WRKY | -0.25 | -3.75 | 0.000234 |
| | LOC_Os05g02390 | C2H2 | -0.25 | -3.69 | 0.000287 |
| | LOC_Os02g50630 | E2F/DP | -0.26 | -3.78 | 0.000211 |
| | LOC_Os10g42850 | WRKY | -0.27 | -3.94 | 0.000114 |
| | LOC_Os01g18240 | MYB | -0.27 | -3.96 | 0.000105 |
| | LOC_Os01g68700 | bHLH | -0.28 | -4.34 | 2.12E-05 |
| | LOC_Os05g51160 | MYB_related | -0.28 | -4.23 | 3.53E-05 |
| | LOC_Os03g42630 | NAC | -0.29 | -4.32 | 2.44E-05 |
| | LOC_Os07g47330 | ERF | -0.29 | -4.21 | 3.98E-05 |
| | LOC_Os06g09420 | B3 | -0.30 | -4.49 | 1.18E-05 |
| | LOC_Os12g42400 | NF-YA | -0.32 | -5.08 | 7.92E-07 |
| | LOC_Os02g46780 | MYB | -0.33 | -5.14 | 6.15E-07 |
| | LOC_Os01g17000 | Dof | -0.34 | -5.27 | 3.25E-07 |
| | LOC_Os01g50720 | MYB | -0.35 | -5.25 | 4.03E-07 |
| | LOC_Os05g20930 | C2H2 | -0.37 | -5.45 | 1.54E-07 |
| | LOC_Os04g44440 | TCP | -0.38 | -5.89 | 1.49E-08 |
| | LOC_Os09g36730 | MYB | -0.38 | -5.89 | 1.64E-08 |
| | LOC_Os08g33660 | MYB | -0.39 | -6.30 | 1.58E-09 |
| G_box | LOC_Os03g60080 | NAC | 0.58 | 9.93 | 3.98E-19 |
| | LOC_Os02g43330 | HD-ZIP | 0.56 | 9.61 | 3.56E-18 |
| | LOC_Os01g50940 | bHLH | 0.55 | 8.95 | 3.65E-16 |
| | LOC_Os01g64360 | MYB | 0.52 | 8.40 | 1.11E-14 |
| | LOC_Os03g60560 | C2H2 | 0.52 | 9.11 | 4.89E-17 |
| | LOC_Os05g37060 | MYB | 0.49 | 7.91 | 1.68E-13 |

| | | | | |
|---|---|---|---|---|
| LOC_Os05g49420 | bZIP | 0.48 | 7.75 | 4.62E-13 |
| LOC_Os03g12370 | HSF | 0.47 | 7.96 | 8.2E-14 |
| LOC_Os01g48320 | Trihelix | 0.46 | 7.83 | 1.9E-13 |
| LOC_Os05g07120 | bHLH | 0.46 | 7.43 | 2.69E-12 |
| LOC_Os01g63980 | C2H2 | 0.46 | 7.58 | 9.64E-13 |
| LOC_Os06g07030 | ERF | 0.44 | 6.78 | 1.55E-10 |
| LOC_Os01g07120 | ERF | 0.44 | 7.03 | 3.08E-11 |
| LOC_Os02g52670 | ERF | 0.44 | 7.28 | 5.84E-12 |
| LOC_Os02g26430 | WRKY | 0.43 | 6.76 | 1.53E-10 |
| LOC_Os02g32590 | HSF | 0.42 | 6.83 | 8.91E-11 |
| LOC_Os06g43090 | MYB | 0.41 | 6.44 | 8.63E-10 |
| LOC_Os01g21120 | ERF | 0.41 | 6.77 | 1.15E-10 |
| LOC_Os02g13800 | HSF | 0.41 | 6.26 | 2.41E-09 |
| LOC_Os06g44010 | WRKY | 0.40 | 6.13 | 4.91E-09 |
| LOC_Os04g46440 | ERF | 0.40 | 6.46 | 6.47E-10 |
| LOC_Os07g36170 | GRAS | 0.40 | 6.48 | 5.72E-10 |
| LOC_Os10g41200 | MYB_related | 0.40 | 6.50 | 4.9E-10 |
| LOC_Os01g64730 | bZIP | 0.39 | 6.25 | 2.17E-09 |
| LOC_Os07g48630 | EIL | 0.39 | 6.27 | 1.8E-09 |
| LOC_Os01g58420 | ERF | 0.38 | 6.19 | 2.87E-09 |
| LOC_Os07g12340 | NAC | 0.38 | 6.16 | 3.38E-09 |
| LOC_Os01g40260 | WRKY | 0.38 | 5.74 | 3.45E-08 |
| LOC_Os04g45810 | HD-ZIP | 0.37 | 5.89 | 1.45E-08 |
| LOC_Os01g39020 | HSF | 0.36 | 5.65 | 5.21E-08 |
| LOC_Os05g41780 | ERF | 0.36 | 5.81 | 2.18E-08 |
| LOC_Os10g33810 | MYB | 0.36 | 5.26 | 3.97E-07 |
| LOC_Os09g38570 | Trihelix | 0.36 | 5.47 | 1.33E-07 |
| LOC_Os01g72490 | SRS | 0.36 | 5.39 | 1.96E-07 |
| LOC_Os03g32230 | C2H2 | 0.35 | 5.63 | 5.26E-08 |
| LOC_Os07g07974 | CPP | 0.35 | 5.25 | 3.82E-07 |
| LOC_Os09g35790 | HSF | 0.35 | 5.28 | 3.43E-07 |
| LOC_Os03g55080 | WRKY | 0.35 | 5.56 | 7.52E-08 |
| LOC_Os07g39470 | GRAS | 0.35 | 5.39 | 1.88E-07 |
| LOC_Os03g21030 | NAC | 0.34 | 5.16 | 5.93E-07 |
| LOC_Os04g51320 | Trihelix | 0.34 | 5.46 | 1.29E-07 |
| LOC_Os11g03300 | NAC | 0.34 | 5.06 | 9.66E-07 |
| LOC_Os03g64260 | ERF | 0.34 | 5.12 | 7.05E-07 |
| LOC_Os08g06110 | MYB_related | 0.34 | 5.38 | 1.85E-07 |
| LOC_Os09g28440 | ERF | 0.34 | 5.01 | 1.22E-06 |
| LOC_Os04g53540 | HD-ZIP | 0.33 | 4.96 | 1.52E-06 |
| LOC_Os08g01090 | B3 | 0.33 | 5.25 | 3.62E-07 |
| LOC_Os05g34830 | NAC | 0.33 | 4.77 | 3.7E-06 |
| LOC_Os02g43790 | ERF | 0.33 | 4.88 | 2.15E-06 |
| LOC_Os08g04840 | MYB_related | 0.33 | 4.69 | 5.4E-06 |
| LOC_Os09g28210 | bHLH | 0.33 | 4.97 | 1.38E-06 |
| LOC_Os08g36920 | ERF | 0.33 | 4.85 | 2.49E-06 |
| LOC_Os04g32620 | ERF | 0.32 | 5.13 | 6.35E-07 |
| LOC_Os10g41460 | Trihelix | 0.32 | 4.88 | 2.13E-06 |
| LOC_Os08g38210 | bHLH | 0.32 | 5.01 | 1.07E-06 |
| LOC_Os03g51330 | GRAS | 0.31 | 4.97 | 1.35E-06 |
| LOC_Os07g38090 | C3H | 0.31 | 4.83 | 2.51E-06 |
| LOC_Os04g58190 | Dof | 0.31 | 4.85 | 2.37E-06 |
| LOC_Os12g07120 | GATA | 0.31 | 4.85 | 2.37E-06 |
| LOC_Os03g02160 | C3H | 0.31 | 4.85 | 2.32E-06 |
| LOC_Os09g32040 | NAC | 0.31 | 4.56 | 8.98E-06 |
| LOC_Os08g31580 | ERF | 0.31 | 4.41 | 1.74E-05 |
| LOC_Os04g43680 | MYB | 0.31 | 4.86 | 2.16E-06 |
| LOC_Os01g14440 | WRKY | 0.31 | 4.81 | 2.83E-06 |
| LOC_Os02g36360 | C2H2 | 0.30 | 4.73 | 4.03E-06 |
| LOC_Os06g45840 | MYB_related | 0.30 | 4.49 | 1.18E-05 |
| LOC_Os01g54600 | WRKY | 0.30 | 4.44 | 1.46E-05 |
| LOC_Os01g15640 | NAC | 0.30 | 4.50 | 1.11E-05 |
| LOC_Os09g35760 | HD-ZIP | 0.30 | 4.20 | 4.14E-05 |
| LOC_Os10g07080 | C2H2 | 0.29 | 4.26 | 3.19E-05 |
| LOC_Os03g31230 | MYB_related | 0.29 | 4.43 | 1.48E-05 |

| | | | | | |
|---|---|---|---|---|---|
| | LOC_Os12g37690 | MYB | 0.28 | 4.28 | 2.89E-05 |
| | LOC_Os01g65900 | GRAS | 0.28 | 4.36 | 1.95E-05 |
| | LOC_Os05g10670 | C3H | 0.28 | 4.08 | 6.57E-05 |
| | LOC_Os02g44120 | C2H2 | 0.28 | 4.05 | 7.31E-05 |
| | LOC_Os04g55520 | ERF | 0.27 | 4.25 | 3.15E-05 |
| | LOC_Os02g05450 | HB-other | 0.27 | 4.03 | 7.84E-05 |
| | LOC_Os08g36790 | bZIP | 0.27 | 3.93 | 0.00012 |
| | LOC_Os05g41760 | ERF | 0.27 | 4.12 | 5.45E-05 |
| | LOC_Os01g54550 | HSF | 0.27 | 4.13 | 5.09E-05 |
| | LOC_Os01g32770 | LBD | 0.27 | 4.14 | 5.02E-05 |
| | LOC_Os01g01870 | bHLH | 0.26 | 4.10 | 5.89E-05 |
| | LOC_Os01g70310 | bHLH | 0.26 | 4.11 | 5.51E-05 |
| | LOC_Os04g57600 | C3H | 0.26 | 4.08 | 6.36E-05 |
| | LOC_Os10g41770 | STAT | 0.26 | 4.02 | 8.02E-05 |
| | LOC_Os09g25060 | WRKY | 0.26 | 3.81 | 0.000184 |
| | LOC_Os03g53020 | bHLH | 0.26 | 3.99 | 8.84E-05 |
| | LOC_Os04g58020 | MYB_related | 0.26 | 3.83 | 0.000167 |
| | LOC_Os05g29810 | ERF | 0.26 | 3.59 | 0.000429 |
| | LOC_Os10g41230 | HD-ZIP | 0.25 | 3.50 | 0.000578 |
| | LOC_Os09g35030 | ERF | 0.25 | 3.62 | 0.00037 |
| | LOC_Os02g05510 | GATA | 0.25 | 3.87 | 0.00014 |
| | LOC_Os01g68370 | B3 | 0.25 | 3.83 | 0.000164 |
| | LOC_Os03g06860 | FAR1 | 0.25 | 3.79 | 0.000193 |
| | LOC_Os04g41560 | DBB | 0.25 | 3.47 | 0.000644 |
| | LOC_Os05g45020 | C3H | 0.24 | 3.49 | 0.000591 |
| | LOC_Os05g37190 | C2H2 | 0.24 | 3.74 | 0.000233 |
| | LOC_Os04g21950 | WRKY | 0.24 | 3.54 | 0.000494 |
| | LOC_Os01g09620 | C3H | 0.24 | 3.52 | 0.000527 |
| | LOC_Os02g52340 | MIKC_MADS | 0.24 | 3.61 | 0.000376 |
| | LOC_Os05g41450 | NF-YC | 0.24 | 3.49 | 0.000591 |
| | LOC_Os03g55590 | G2-like | 0.24 | 3.75 | 0.000226 |
| | LOC_Os01g61080 | WRKY | 0.24 | 3.66 | 0.000317 |
| | LOC_Os06g41770 | bZIP | 0.24 | 3.52 | 0.000524 |
| | LOC_Os01g70270 | ARF | 0.23 | 3.58 | 0.00042 |
| | LOC_Os03g06630 | HSF | 0.23 | 3.48 | 0.000613 |
| | LOC_Os02g45450 | ERF | 0.23 | 3.23 | 0.00148 |
| | LOC_Os03g55540 | C2H2 | 0.23 | 3.51 | 0.000549 |
| | LOC_Os04g55970 | AP2 | 0.23 | 3.48 | 0.000599 |
| | LOC_Os09g24820 | ZF-HD | 0.23 | 3.19 | 0.001654 |
| | LOC_Os06g07010 | FAR1 | 0.23 | 3.39 | 0.000838 |
| | LOC_Os09g31400 | EIL | 0.23 | 3.23 | 0.001461 |
| | LOC_Os03g12350 | ARR-B | 0.22 | 3.35 | 0.000952 |
| | LOC_Os10g22950 | CAMTA | 0.22 | 3.49 | 0.000586 |
| | LOC_Os03g43840 | LSD | 0.22 | 3.29 | 0.001185 |
| | LOC_Os01g04020 | ERF | 0.22 | 3.29 | 0.001172 |
| | LOC_Os01g09280 | MYB_related | 0.22 | 3.34 | 0.000978 |
| | LOC_Os12g41210 | CPP | 0.22 | 3.32 | 0.001044 |
| | LOC_Os08g14400 | WOX | 0.21 | 3.27 | 0.001238 |
| | LOC_Os04g51560 | WRKY | 0.21 | 3.08 | 0.002396 |
| | LOC_Os01g13460 | bHLH | 0.21 | 3.14 | 0.001925 |
| | LOC_Os03g54160 | MIKC_MADS | 0.21 | 2.97 | 0.003398 |
| | LOC_Os07g39310 | C2H2 | 0.21 | 2.99 | 0.003104 |
| | LOC_Os01g62660 | G2-like | 0.21 | 2.97 | 0.0033 |
| | LOC_Os09g29930 | bHLH | 0.20 | 2.82 | 0.005327 |
| | LOC_Os03g26210 | bHLH | 0.20 | 3.11 | 0.00208 |
| | LOC_Os01g15900 | Dof | 0.20 | 3.00 | 0.00304 |
| | LOC_Os01g64000 | bZIP | 0.20 | 3.00 | 0.002993 |
| | LOC_Os11g06170 | bZIP | 0.20 | 2.88 | 0.00443 |
| | LOC_Os02g35770 | HD-ZIP | 0.20 | 3.02 | 0.00285 |
| | LOC_Os11g02480 | WRKY | 0.20 | 2.78 | 0.006 |
| | LOC_Os01g67480 | bHLH | 0.20 | 2.88 | 0.004362 |
| | LOC_Os04g45650 | GATA | 0.20 | 2.96 | 0.003367 |
| | LOC_Os01g41900 | MYB_related | 0.20 | 2.80 | 0.005641 |
| | LOC_Os03g20780 | EIL | 0.19 | 2.79 | 0.005866 |
| | LOC_Os05g03760 | C3H | 0.19 | 2.80 | 0.005662 |

| | | | | |
|---|---|---|---|---|
| LOC_Os08g15050 | CO-like | 0.19 | 2.78 | 0.005871 |
| LOC_Os03g42290 | B3 | 0.19 | 2.91 | 0.003937 |
| LOC_Os06g12400 | HB-PHD | 0.19 | 2.78 | 0.005948 |
| LOC_Os10g30719 | MYB_related | 0.19 | 2.69 | 0.007688 |
| LOC_Os11g06410 | Trihelix | 0.19 | 2.71 | 0.007272 |
| LOC_Os12g40570 | WRKY | 0.19 | 2.88 | 0.00432 |
| LOC_Os05g37080 | NAC | 0.19 | 2.84 | 0.004996 |
| LOC_Os01g10504 | MIKC_MADS | 0.19 | 2.64 | 0.008902 |
| LOC_Os03g55990 | HB-other | 0.19 | 2.86 | 0.004567 |
| LOC_Os07g48870 | MYB | 0.19 | 2.84 | 0.004898 |
| LOC_Os09g25070 | WRKY | 0.19 | 2.82 | 0.005283 |
| LOC_Os06g45140 | bZIP | 0.18 | 2.62 | 0.009419 |
| LOC_Os07g22730 | ERF | 0.18 | 2.55 | 0.011663 |
| LOC_Os03g17810 | LBD | 0.18 | 2.57 | 0.011 |
| LOC_Os03g58160 | HSF | 0.18 | 2.77 | 0.006107 |
| LOC_Os05g45410 | HSF | 0.18 | 2.49 | 0.013567 |
| LOC_Os04g58000 | B3 | 0.18 | 2.70 | 0.007449 |
| LOC_Os12g10540 | MIKC_MADS | 0.18 | 2.61 | 0.009847 |
| LOC_Os01g64310 | NAC | 0.18 | 2.65 | 0.008737 |
| LOC_Os06g14190 | NF-X1 | 0.18 | 2.53 | 0.012269 |
| LOC_Os09g35880 | DBB | 0.18 | 2.49 | 0.013616 |
| LOC_Os06g45890 | G2-like | 0.17 | 2.49 | 0.013506 |
| LOC_Os01g62410 | MYB | 0.17 | 2.63 | 0.009033 |
| LOC_Os10g42490 | HD-ZIP | 0.17 | 2.65 | 0.008734 |
| LOC_Os02g10840 | FAR1 | 0.17 | 2.65 | 0.008548 |
| LOC_Os02g40530 | MYB | 0.17 | 2.60 | 0.009906 |
| LOC_Os02g45480 | C3H | 0.17 | 2.42 | 0.016254 |
| LOC_Os04g49450 | MYB_related | 0.17 | 2.57 | 0.010713 |
| LOC_Os01g51690 | WRKY | 0.17 | 2.49 | 0.01366 |
| LOC_Os08g29660 | WRKY | 0.17 | 2.59 | 0.010306 |
| LOC_Os11g03370 | NAC | 0.17 | 2.56 | 0.011242 |
| LOC_Os02g07170 | G2-like | 0.17 | 2.36 | 0.019277 |
| LOC_Os03g42430 | B3 | 0.17 | 2.52 | 0.012597 |
| LOC_Os02g58670 | bZIP | 0.17 | 2.53 | 0.012 |
| LOC_Os09g28354 | HSF | 0.17 | 2.35 | 0.019963 |
| LOC_Os03g21710 | WRKY | 0.17 | 2.38 | 0.018364 |
| LOC_Os01g06550 | NF-X1 | 0.16 | 2.48 | 0.013792 |
| LOC_Os11g39000 | bHLH | 0.16 | 2.42 | 0.016539 |
| LOC_Os07g44690 | HSF | 0.16 | 2.23 | 0.026643 |
| LOC_Os04g49110 | GRAS | 0.16 | 2.43 | 0.015712 |
| LOC_Os09g28310 | bZIP | 0.16 | 2.41 | 0.016597 |
| LOC_Os01g62460 | GRAS | 0.16 | 2.32 | 0.021052 |
| LOC_Os07g48570 | Dof | 0.16 | 2.38 | 0.018106 |
| LOC_Os05g09020 | WRKY | 0.16 | 2.18 | 0.030665 |
| LOC_Os03g48970 | NF-YA | 0.16 | 2.37 | 0.018706 |
| LOC_Os09g12770 | G2-like | 0.15 | 2.24 | 0.025853 |
| LOC_Os01g55340 | Dof | 0.15 | 2.20 | 0.029081 |
| LOC_Os01g12440 | ERF | 0.15 | 2.20 | 0.029319 |
| LOC_Os03g05690 | C2H2 | 0.15 | 2.30 | 0.022208 |
| LOC_Os12g21700 | C3H | 0.15 | 2.25 | 0.0255 |
| LOC_Os02g08150 | CO-like | 0.15 | 2.20 | 0.029008 |
| LOC_Os03g08500 | ERF | 0.15 | 2.19 | 0.029724 |
| LOC_Os07g04700 | MYB | 0.15 | 2.32 | 0.021198 |
| LOC_Os06g09370 | bHLH | 0.15 | 2.19 | 0.030034 |
| LOC_Os02g47280 | GRF | 0.15 | 2.16 | 0.032313 |
| LOC_Os08g04390 | bHLH | 0.15 | 2.24 | 0.025997 |
| LOC_Os09g01960 | MYB | 0.15 | 2.09 | 0.038204 |
| LOC_Os03g58250 | bZIP | 0.15 | 2.17 | 0.031129 |
| LOC_Os03g53960 | MYB_related | 0.15 | 2.18 | 0.030479 |
| LOC_Os01g53650 | C3H | 0.15 | 2.06 | 0.04093 |
| LOC_Os03g11370 | B3 | 0.14 | 2.03 | 0.044233 |
| LOC_Os07g35870 | bHLH | 0.14 | 2.17 | 0.030977 |
| LOC_Os01g51610 | B3 | 0.14 | 2.07 | 0.039513 |
| LOC_Os07g48550 | NAC | 0.14 | 2.15 | 0.032758 |
| LOC_Os02g45850 | B3 | 0.14 | 1.99 | 0.047548 |

| | | | | | |
|---|---|---|---|---|---|
| | LOC_Os03g05480 | C2H2 | 0.14 | 2.10 | 0.037128 |
| | LOC_Os01g06320 | MYB_related | 0.14 | 2.08 | 0.038499 |
| | LOC_Os03g10210 | HD-ZIP | 0.14 | 1.98 | 0.049486 |
| | LOC_Os02g44130 | C2H2 | 0.14 | 1.98 | 0.049522 |
| | LOC_Os08g39980 | MYB_related | 0.14 | 2.08 | 0.038391 |
| | LOC_Os03g03900 | Nin-like | 0.14 | 2.00 | 0.047002 |
| | LOC_Os02g51670 | ERF | 0.14 | 2.08 | 0.038499 |
| | LOC_Os08g17400 | WRKY | 0.13 | 2.01 | 0.045522 |
| | LOC_Os02g12310 | NAC | 0.13 | 2.02 | 0.044168 |
| | LOC_Os01g10610 | BES1 | 0.13 | 2.02 | 0.044524 |
| | LOC_Os10g37240 | Trihelix | 0.13 | 1.97 | 0.049559 |
| | LOC_Os02g06910 | ARF | 0.13 | 1.99 | 0.047623 |
| | LOC_Os03g20900 | G2-like | -0.13 | -1.97 | 0.049682 |
| | LOC_Os02g47060 | WRKY | -0.13 | -2.01 | 0.04538 |
| | LOC_Os07g44640 | C2H2 | -0.14 | -2.01 | 0.045212 |
| | LOC_Os08g42470 | bHLH | -0.14 | -2.05 | 0.041319 |
| | LOC_Os03g58330 | bHLH | -0.14 | -1.99 | 0.048094 |
| | LOC_Os03g17150 | C2H2 | -0.14 | -2.08 | 0.038925 |
| | LOC_Os06g02230 | B3 | -0.15 | -2.19 | 0.029565 |
| | LOC_Os03g19370 | bZIP | -0.15 | -2.21 | 0.027934 |
| | LOC_Os01g09550 | NAC | -0.15 | -2.24 | 0.025862 |
| | LOC_Os03g07880 | NF-YA | -0.15 | -2.20 | 0.02873 |
| | LOC_Os03g46860 | bHLH | -0.15 | -2.12 | 0.034864 |
| | LOC_Os06g04010 | BBR-BPC | -0.15 | -2.31 | 0.02182 |
| | LOC_Os12g01490 | G2-like | -0.15 | -2.21 | 0.028371 |
| | LOC_Os02g46560 | bHLH | -0.16 | -2.34 | 0.020037 |
| | LOC_Os05g35170 | NAC | -0.16 | -2.27 | 0.024261 |
| | LOC_Os06g04850 | HD-ZIP | -0.16 | -2.30 | 0.022253 |
| | LOC_Os08g41940 | SBP | -0.16 | -2.47 | 0.014185 |
| | LOC_Os02g15340 | NAC | -0.17 | -2.54 | 0.011613 |
| | LOC_Os11g05740 | B3 | -0.17 | -2.54 | 0.011806 |
| | LOC_Os12g06200 | E2F/DP | -0.17 | -2.61 | 0.009634 |
| | LOC_Os03g55760 | G2-like | -0.17 | -2.55 | 0.011458 |
| | LOC_Os04g43910 | ARF | -0.17 | -2.52 | 0.012593 |
| | LOC_Os12g39330 | ERF | -0.18 | -2.43 | 0.015862 |
| | LOC_Os02g34970 | NAC | -0.18 | -2.68 | 0.007931 |
| | LOC_Os01g03720 | MYB | -0.18 | -2.66 | 0.008363 |
| | LOC_Os02g12790 | GATA | -0.18 | -2.55 | 0.011614 |
| | LOC_Os01g74140 | WRKY | -0.18 | -2.63 | 0.009058 |
| | LOC_Os01g53040 | WRKY | -0.18 | -2.72 | 0.007113 |
| | LOC_Os02g44370 | GRAS | -0.18 | -2.62 | 0.00959 |
| | LOC_Os08g33750 | G2-like | -0.18 | -2.75 | 0.006468 |
| | LOC_Os01g11350 | bZIP | -0.19 | -2.80 | 0.005614 |
| | LOC_Os03g04310 | bHLH | -0.19 | -2.78 | 0.005858 |
| | LOC_Os05g41240 | G2-like | -0.19 | -2.67 | 0.008112 |
| | LOC_Os03g15440 | bHLH | -0.19 | -2.85 | 0.004723 |
| | LOC_Os01g48130 | NAC | -0.19 | -2.76 | 0.006299 |
| | LOC_Os06g35140 | G2-like | -0.19 | -2.70 | 0.007527 |
| | LOC_Os06g09717 | ERF | -0.19 | -2.67 | 0.008213 |
| | LOC_Os06g01480 | NAC | -0.19 | -2.86 | 0.004671 |
| | LOC_Os09g26420 | ERF | -0.19 | -2.80 | 0.005532 |
| | LOC_Os03g07450 | HD-ZIP | -0.19 | -2.65 | 0.008767 |
| | LOC_Os05g38120 | TALE | -0.19 | -2.73 | 0.006887 |
| | LOC_Os09g13570 | bZIP | -0.20 | -2.88 | 0.004368 |
| | LOC_Os09g32510 | bHLH | -0.20 | -2.82 | 0.005326 |
| | LOC_Os01g08160 | G2-like | -0.20 | -3.05 | 0.002572 |
| | LOC_Os06g04090 | NAC | -0.20 | -2.91 | 0.004006 |
| | LOC_Os08g02300 | NAC | -0.20 | -3.06 | 0.002465 |
| | LOC_Os05g41540 | bZIP | -0.20 | -2.94 | 0.00364 |
| | LOC_Os06g46270 | NAC | -0.20 | -2.97 | 0.003351 |
| | LOC_Os02g50630 | E2F/DP | -0.21 | -2.94 | 0.003712 |
| | LOC_Os05g02390 | C2H2 | -0.21 | -3.02 | 0.002857 |
| | LOC_Os06g49040 | G2-like | -0.21 | -3.04 | 0.002677 |
| | LOC_Os12g41680 | NAC | -0.22 | -3.27 | 0.001266 |
| | LOC_Os02g39140 | bHLH | -0.22 | -3.13 | 0.002002 |

| | | | | | |
|---|---|---|---|---|---|
| | LOC_Os03g05500 | LBD | -0.22 | -3.15 | 0.001884 |
| | LOC_Os01g63460 | MYB | -0.22 | -3.34 | 0.000975 |
| | LOC_Os01g09850 | C2H2 | -0.22 | -3.16 | 0.001838 |
| | LOC_Os04g43560 | NAC | -0.23 | -3.29 | 0.001195 |
| | LOC_Os07g22770 | ERF | -0.23 | -3.58 | 0.000417 |
| | LOC_Os04g36590 | FAR1 | -0.24 | -3.38 | 0.000886 |
| | LOC_Os04g08060 | C2H2 | -0.24 | -3.41 | 0.000789 |
| | LOC_Os07g48596 | G2-like | -0.24 | -3.30 | 0.001169 |
| | LOC_Os06g08440 | ARR-B | -0.24 | -3.37 | 0.000917 |
| | LOC_Os02g52190 | bHLH | -0.24 | -3.36 | 0.000951 |
| | LOC_Os10g17630 | B3 | -0.24 | -3.59 | 0.000417 |
| | LOC_Os12g37970 | MYB | -0.24 | -3.73 | 0.000242 |
| | LOC_Os05g02420 | MYB_related | -0.24 | -3.61 | 0.000382 |
| | LOC_Os12g43950 | TALE | -0.25 | -3.73 | 0.000246 |
| | LOC_Os05g50080 | C3H | -0.25 | -3.57 | 0.000447 |
| | LOC_Os06g09420 | B3 | -0.25 | -3.65 | 0.000333 |
| | LOC_Os11g05480 | bZIP | -0.25 | -3.59 | 0.000418 |
| | LOC_Os04g50920 | WRKY | -0.26 | -3.81 | 0.000185 |
| | LOC_Os08g44830 | C2H2 | -0.26 | -3.84 | 0.000162 |
| | LOC_Os02g44360 | GRAS | -0.26 | -3.85 | 0.000161 |
| | LOC_Os09g10840 | bZIP | -0.26 | -3.82 | 0.000181 |
| | LOC_Os01g74410 | MYB | -0.27 | -4.12 | 5.45E-05 |
| | LOC_Os04g54474 | bZIP | -0.27 | -3.95 | 0.000108 |
| | LOC_Os01g53260 | WRKY | -0.27 | -3.96 | 0.000106 |
| | LOC_Os05g04820 | MYB | -0.28 | -4.18 | 4.38E-05 |
| | LOC_Os01g36460 | MYB | -0.28 | -4.05 | 7.59E-05 |
| | LOC_Os09g33490 | NAC | -0.28 | -4.14 | 5.15E-05 |
| | LOC_Os04g50770 | MYB | -0.29 | -4.30 | 2.73E-05 |
| | LOC_Os03g42630 | NAC | -0.29 | -4.32 | 2.46E-05 |
| | LOC_Os07g47330 | ERF | -0.29 | -4.22 | 3.77E-05 |
| | LOC_Os09g29820 | bZIP | -0.31 | -4.56 | 8.93E-06 |
| | LOC_Os04g35010 | bHLH | -0.32 | -4.74 | 4.04E-06 |
| | LOC_Os10g42850 | WRKY | -0.32 | -4.75 | 3.79E-06 |
| | LOC_Os01g68700 | bHLH | -0.32 | -5.00 | 1.14E-06 |
| | LOC_Os07g39800 | HRT-like | -0.32 | -5.17 | 5.12E-07 |
| | LOC_Os05g48850 | NAC | -0.35 | -5.11 | 7.85E-07 |
| | LOC_Os02g46780 | MYB | -0.35 | -5.49 | 1.12E-07 |
| | LOC_Os12g42400 | NF-YA | -0.36 | -5.69 | 3.97E-08 |
| | LOC_Os05g51160 | MYB_related | -0.37 | -5.66 | 5.11E-08 |
| | LOC_Os01g50720 | MYB | -0.37 | -5.64 | 6.02E-08 |
| | LOC_Os01g17000 | Dof | -0.38 | -6.01 | 7.57E-09 |
| | LOC_Os01g18240 | MYB | -0.38 | -5.69 | 4.65E-08 |
| | LOC_Os09g36730 | MYB | -0.38 | -5.89 | 1.62E-08 |
| | LOC_Os05g20930 | C2H2 | -0.39 | -5.89 | 1.69E-08 |
| | LOC_Os04g44440 | TCP | -0.40 | -6.41 | 9.19E-10 |
| | LOC_Os08g33660 | MYB | -0.45 | -7.54 | 1.18E-12 |
| ABRE_M4 | LOC_Os02g43330 | HD-ZIP | 0.54 | 8.97 | 2.38E-16 |
| | LOC_Os01g50940 | bHLH | 0.53 | 8.51 | 5.88E-15 |
| | LOC_Os03g60080 | NAC | 0.52 | 8.63 | 1.98E-15 |
| | LOC_Os09g28210 | bHLH | 0.46 | 7.50 | 1.81E-12 |
| | LOC_Os03g60560 | C2H2 | 0.45 | 7.45 | 2.05E-12 |
| | LOC_Os05g49420 | bZIP | 0.44 | 6.92 | 6.12E-11 |
| | LOC_Os05g37060 | MYB | 0.44 | 6.90 | 6.55E-11 |
| | LOC_Os02g52670 | ERF | 0.43 | 6.97 | 3.67E-11 |
| | LOC_Os02g26430 | WRKY | 0.41 | 6.42 | 1E-09 |
| | LOC_Os01g64360 | MYB | 0.40 | 5.91 | 1.64E-08 |
| | LOC_Os04g45810 | HD-ZIP | 0.40 | 6.37 | 1.13E-09 |
| | LOC_Os06g07030 | ERF | 0.38 | 5.63 | 6.49E-08 |
| | LOC_Os02g13800 | HSF | 0.38 | 5.65 | 5.59E-08 |
| | LOC_Os03g12370 | HSF | 0.37 | 6.05 | 5.77E-09 |
| | LOC_Os02g32590 | HSF | 0.37 | 5.82 | 2.13E-08 |
| | LOC_Os07g36170 | GRAS | 0.37 | 5.87 | 1.6E-08 |
| | LOC_Os09g35790 | HSF | 0.36 | 5.54 | 9.68E-08 |
| | LOC_Os07g12340 | NAC | 0.35 | 5.56 | 7.47E-08 |
| | LOC_Os06g43090 | MYB | 0.35 | 5.25 | 3.87E-07 |

| | | | | | |
|---|---|---|---|---|---|
| | LOC_Os02g43790 | ERF | 0.34 | 5.12 | 7.24E-07 |
| | LOC_Os01g63980 | C2H2 | 0.34 | 5.30 | 2.89E-07 |
| | LOC_Os03g21030 | NAC | 0.34 | 5.02 | 1.17E-06 |
| | LOC_Os08g36920 | ERF | 0.33 | 4.99 | 1.33E-06 |
| | LOC_Os11g03300 | NAC | 0.33 | 4.92 | 1.8E-06 |
| | LOC_Os09g38570 | Trihelix | 0.33 | 4.98 | 1.38E-06 |
| | LOC_Os01g48320 | Trihelix | 0.32 | 5.14 | 6.12E-07 |
| | LOC_Os01g07120 | ERF | 0.32 | 4.90 | 1.95E-06 |
| | LOC_Os01g67480 | bHLH | 0.32 | 4.89 | 2.07E-06 |
| | LOC_Os04g46440 | ERF | 0.32 | 5.03 | 1.03E-06 |
| | LOC_Os05g07120 | bHLH | 0.32 | 4.88 | 2.09E-06 |
| | LOC_Os01g58420 | ERF | 0.31 | 4.96 | 1.39E-06 |
| | LOC_Os06g44010 | WRKY | 0.31 | 4.61 | 7.32E-06 |
| | LOC_Os01g15640 | NAC | 0.31 | 4.75 | 3.75E-06 |
| | LOC_Os08g04840 | MYB_related | 0.31 | 4.39 | 1.94E-05 |
| | LOC_Os10g41200 | MYB_related | 0.31 | 4.88 | 1.96E-06 |
| | LOC_Os10g07080 | C2H2 | 0.31 | 4.54 | 9.85E-06 |
| | LOC_Os04g32620 | ERF | 0.31 | 4.82 | 2.68E-06 |
| | LOC_Os12g37690 | MYB | 0.31 | 4.64 | 6.28E-06 |
| | LOC_Os10g33810 | MYB | 0.31 | 4.37 | 2.09E-05 |
| | LOC_Os03g64260 | ERF | 0.30 | 4.49 | 1.21E-05 |
| | LOC_Os09g35760 | HD-ZIP | 0.30 | 4.29 | 2.88E-05 |
| | LOC_Os02g36360 | C2H2 | 0.30 | 4.66 | 5.43E-06 |
| | LOC_Os04g58020 | MYB_related | 0.30 | 4.53 | 9.97E-06 |
| | LOC_Os08g38210 | bHLH | 0.30 | 4.68 | 4.88E-06 |
| | LOC_Os02g40530 | MYB | 0.30 | 4.60 | 7.22E-06 |
| | LOC_Os07g48630 | EIL | 0.30 | 4.63 | 6.34E-06 |
| | LOC_Os10g41460 | Trihelix | 0.30 | 4.48 | 1.21E-05 |
| | LOC_Os01g21120 | ERF | 0.29 | 4.60 | 7.12E-06 |
| | LOC_Os05g10670 | C3H | 0.29 | 4.25 | 3.36E-05 |
| | LOC_Os04g41560 | DBB | 0.29 | 4.15 | 5.05E-05 |
| | LOC_Os07g38090 | C3H | 0.29 | 4.43 | 1.46E-05 |
| | LOC_Os01g01870 | bHLH | 0.29 | 4.46 | 1.3E-05 |
| | LOC_Os01g40260 | WRKY | 0.28 | 4.15 | 4.96E-05 |
| | LOC_Os01g72490 | SRS | 0.28 | 4.17 | 4.54E-05 |
| | LOC_Os03g55080 | WRKY | 0.28 | 4.31 | 2.43E-05 |
| | LOC_Os01g64730 | bZIP | 0.28 | 4.22 | 3.67E-05 |
| | LOC_Os01g39020 | HSF | 0.28 | 4.14 | 5.12E-05 |
| | LOC_Os03g31230 | MYB_related | 0.27 | 4.17 | 4.5E-05 |
| | LOC_Os02g45450 | ERF | 0.27 | 3.91 | 0.000129 |
| | LOC_Os03g55590 | G2-like | 0.27 | 4.26 | 3.03E-05 |
| | LOC_Os09g28440 | ERF | 0.27 | 3.96 | 0.000106 |
| | LOC_Os07g07974 | CPP | 0.27 | 3.94 | 0.000111 |
| | LOC_Os08g06110 | MYB_related | 0.27 | 4.17 | 4.37E-05 |
| | LOC_Os01g61080 | WRKY | 0.27 | 4.14 | 5E-05 |
| | LOC_Os07g39470 | GRAS | 0.27 | 4.06 | 6.82E-05 |
| | LOC_Os03g54160 | MIKC_MADS | 0.27 | 3.86 | 0.000156 |
| | LOC_Os08g31580 | ERF | 0.27 | 3.78 | 0.000214 |
| | LOC_Os10g41770 | STAT | 0.27 | 4.08 | 6.2E-05 |
| | LOC_Os04g43680 | MYB | 0.27 | 4.15 | 4.6E-05 |
| | LOC_Os01g14440 | WRKY | 0.26 | 4.06 | 6.8E-05 |
| | LOC_Os03g32230 | C2H2 | 0.26 | 4.02 | 7.87E-05 |
| | LOC_Os01g54600 | WRKY | 0.26 | 3.80 | 0.000191 |
| | LOC_Os02g08150 | CO-like | 0.26 | 3.74 | 0.000237 |
| | LOC_Os03g12350 | ARR-B | 0.26 | 3.85 | 0.000156 |
| | LOC_Os03g53020 | bHLH | 0.25 | 3.83 | 0.000168 |
| | LOC_Os08g01090 | B3 | 0.25 | 3.77 | 0.000213 |
| | LOC_Os08g15050 | CO-like | 0.25 | 3.57 | 0.000451 |
| | LOC_Os09g35030 | ERF | 0.25 | 3.54 | 0.0005 |
| | LOC_Os03g48970 | NF-YA | 0.24 | 3.76 | 0.000218 |
| | LOC_Os10g41230 | HD-ZIP | 0.24 | 3.41 | 0.000795 |
| | LOC_Os03g43840 | LSD | 0.24 | 3.64 | 0.000341 |
| | LOC_Os05g41780 | ERF | 0.24 | 3.76 | 0.000219 |
| | LOC_Os01g10504 | MIKC_MADS | 0.24 | 3.45 | 0.000679 |
| | LOC_Os12g21700 | C3H | 0.24 | 3.62 | 0.000365 |

| | | | | |
|---|---|---|---|---|
| LOC_Os08g04390 | bHLH | 0.24 | 3.64 | 0.000337 |
| LOC_Os10g22950 | CAMTA | 0.24 | 3.75 | 0.000225 |
| LOC_Os11g06170 | bZIP | 0.24 | 3.49 | 0.000604 |
| LOC_Os01g15900 | Dof | 0.24 | 3.59 | 0.000416 |
| LOC_Os02g44120 | C2H2 | 0.24 | 3.42 | 0.000777 |
| LOC_Os04g51320 | Trihelix | 0.24 | 3.66 | 0.00032 |
| LOC_Os05g41760 | ERF | 0.24 | 3.57 | 0.000435 |
| LOC_Os01g70310 | bHLH | 0.24 | 3.64 | 0.000342 |
| LOC_Os05g34830 | NAC | 0.23 | 3.24 | 0.001425 |
| LOC_Os03g51330 | GRAS | 0.23 | 3.57 | 0.000435 |
| LOC_Os01g06320 | MYB_related | 0.23 | 3.52 | 0.000527 |
| LOC_Os08g36790 | bZIP | 0.23 | 3.28 | 0.00121 |
| LOC_Os07g48570 | Dof | 0.23 | 3.49 | 0.000582 |
| LOC_Os03g55540 | C2H2 | 0.23 | 3.50 | 0.00057 |
| LOC_Os05g03760 | C3H | 0.23 | 3.29 | 0.001191 |
| LOC_Os10g30719 | MYB_related | 0.23 | 3.22 | 0.001491 |
| LOC_Os05g37080 | NAC | 0.23 | 3.42 | 0.000749 |
| LOC_Os12g07120 | GATA | 0.22 | 3.43 | 0.000729 |
| LOC_Os03g20780 | EIL | 0.22 | 3.21 | 0.001547 |
| LOC_Os01g65900 | GRAS | 0.22 | 3.40 | 0.000812 |
| LOC_Os09g28310 | bZIP | 0.22 | 3.38 | 0.000855 |
| LOC_Os05g09020 | WRKY | 0.22 | 3.05 | 0.002615 |
| LOC_Os02g35770 | HD-ZIP | 0.22 | 3.30 | 0.001136 |
| LOC_Os09g35880 | DBB | 0.22 | 3.08 | 0.002357 |
| LOC_Os04g53540 | HD-ZIP | 0.21 | 3.07 | 0.002464 |
| LOC_Os03g02160 | C3H | 0.21 | 3.24 | 0.001396 |
| LOC_Os09g24820 | ZF-HD | 0.21 | 2.95 | 0.003622 |
| LOC_Os02g51670 | ERF | 0.21 | 3.24 | 0.00137 |
| LOC_Os01g32770 | LBD | 0.21 | 3.19 | 0.001612 |
| LOC_Os08g41030 | ERF | 0.21 | 3.13 | 0.00198 |
| LOC_Os01g09620 | C3H | 0.21 | 3.00 | 0.003031 |
| LOC_Os09g32040 | NAC | 0.21 | 2.99 | 0.003163 |
| LOC_Os03g11370 | B3 | 0.20 | 2.90 | 0.004201 |
| LOC_Os04g42020 | CO-like | 0.20 | 2.95 | 0.0036 |
| LOC_Os05g10690 | MYB_related | 0.20 | 2.96 | 0.003476 |
| LOC_Os04g51560 | WRKY | 0.20 | 2.88 | 0.004451 |
| LOC_Os04g55520 | ERF | 0.19 | 2.95 | 0.003523 |
| LOC_Os01g55340 | Dof | 0.19 | 2.72 | 0.007101 |
| LOC_Os09g29930 | bHLH | 0.19 | 2.62 | 0.009614 |
| LOC_Os02g52340 | MIKC_MADS | 0.19 | 2.79 | 0.00571 |
| LOC_Os03g42430 | B3 | 0.19 | 2.84 | 0.00487 |
| LOC_Os06g14190 | NF-X1 | 0.19 | 2.69 | 0.007744 |
| LOC_Os06g45840 | MYB_related | 0.19 | 2.71 | 0.007348 |
| LOC_Os03g11614 | MIKC_MADS | 0.19 | 2.56 | 0.011247 |
| LOC_Os04g58190 | Dof | 0.19 | 2.80 | 0.005513 |
| LOC_Os01g09280 | MYB_related | 0.18 | 2.82 | 0.00531 |
| LOC_Os05g11414 | MIKC_MADS | 0.18 | 2.62 | 0.00948 |
| LOC_Os11g02480 | WRKY | 0.18 | 2.55 | 0.011724 |
| LOC_Os01g60270 | WOX | 0.18 | 2.76 | 0.006292 |
| LOC_Os03g26210 | bHLH | 0.18 | 2.74 | 0.006557 |
| LOC_Os12g10540 | MIKC_MADS | 0.18 | 2.64 | 0.008864 |
| LOC_Os02g05510 | GATA | 0.18 | 2.73 | 0.006894 |
| LOC_Os05g45410 | HSF | 0.18 | 2.49 | 0.013593 |
| LOC_Os04g21950 | WRKY | 0.18 | 2.54 | 0.011706 |
| LOC_Os01g65080 | C2H2 | 0.18 | 2.70 | 0.007553 |
| LOC_Os12g42610 | YABBY | 0.18 | 2.68 | 0.007894 |
| LOC_Os03g31240 | C2H2 | 0.18 | 2.60 | 0.009923 |
| LOC_Os07g48550 | NAC | 0.18 | 2.66 | 0.008346 |
| LOC_Os02g49700 | HD-ZIP | 0.17 | 2.44 | 0.015624 |
| LOC_Os03g08960 | HD-ZIP | 0.17 | 2.63 | 0.009011 |
| LOC_Os01g64310 | NAC | 0.17 | 2.59 | 0.010308 |
| LOC_Os03g06630 | HSF | 0.17 | 2.56 | 0.011077 |
| LOC_Os04g47990 | Dof | 0.17 | 2.59 | 0.010091 |
| LOC_Os02g05450 | HB-other | 0.17 | 2.44 | 0.015353 |
| LOC_Os07g22730 | ERF | 0.17 | 2.34 | 0.020449 |

| | | | | | |
|---|---|---|---|---|---|
| | LOC_Os01g13540 | Nin-like | 0.17 | 2.51 | 0.012676 |
| | LOC_Os04g49110 | GRAS | 0.17 | 2.51 | 0.012844 |
| | LOC_Os01g62660 | G2-like | 0.16 | 2.35 | 0.019558 |
| | LOC_Os03g58160 | HSF | 0.16 | 2.52 | 0.012534 |
| | LOC_Os11g29870 | WRKY | 0.16 | 2.33 | 0.020664 |
| | LOC_Os07g01820 | MIKC_MADS | 0.16 | 2.46 | 0.014687 |
| | LOC_Os06g45890 | G2-like | 0.16 | 2.31 | 0.022022 |
| | LOC_Os06g07010 | FAR1 | 0.16 | 2.38 | 0.017969 |
| | LOC_Os06g40960 | C2H2 | 0.16 | 2.28 | 0.023768 |
| | LOC_Os08g23470 | B3 | 0.16 | 2.26 | 0.024723 |
| | LOC_Os09g28354 | HSF | 0.16 | 2.22 | 0.027633 |
| | LOC_Os09g25060 | WRKY | 0.16 | 2.22 | 0.027697 |
| | LOC_Os02g43170 | DBB | 0.15 | 2.19 | 0.029639 |
| | LOC_Os12g41210 | CPP | 0.15 | 2.34 | 0.020422 |
| | LOC_Os02g08540 | bZIP | 0.15 | 2.16 | 0.03176 |
| | LOC_Os02g12310 | NAC | 0.15 | 2.31 | 0.02195 |
| | LOC_Os09g35910 | HD-ZIP | 0.15 | 2.09 | 0.038094 |
| | LOC_Os03g17810 | LBD | 0.15 | 2.11 | 0.036346 |
| | LOC_Os09g12770 | G2-like | 0.15 | 2.17 | 0.031445 |
| | LOC_Os03g06930 | TALE | 0.15 | 2.24 | 0.026355 |
| | LOC_Os04g49450 | MYB_related | 0.15 | 2.20 | 0.028521 |
| | LOC_Os05g37190 | C2H2 | 0.15 | 2.22 | 0.027773 |
| | LOC_Os05g27930 | ERF | 0.15 | 2.02 | 0.044364 |
| | LOC_Os07g38240 | C2H2 | 0.15 | 2.09 | 0.037527 |
| | LOC_Os08g41950 | MIKC_MADS | 0.14 | 2.01 | 0.045638 |
| | LOC_Os01g54550 | HSF | 0.14 | 2.16 | 0.032006 |
| | LOC_Os07g41370 | MIKC_MADS | 0.14 | 2.16 | 0.032105 |
| | LOC_Os07g48820 | bZIP | 0.14 | 2.05 | 0.042029 |
| | LOC_Os01g43590 | HSF | 0.14 | 2.05 | 0.041508 |
| | LOC_Os11g39000 | bHLH | 0.14 | 2.10 | 0.036585 |
| | LOC_Os04g42950 | MYB | 0.14 | 2.03 | 0.044084 |
| | LOC_Os02g29550 | ERF | 0.14 | 2.02 | 0.045024 |
| | LOC_Os01g34060 | MYB_related | 0.14 | 1.98 | 0.049077 |
| | LOC_Os01g13460 | bHLH | 0.14 | 2.04 | 0.042928 |
| | LOC_Os07g48870 | MYB | 0.14 | 2.09 | 0.037853 |
| | LOC_Os12g13170 | bZIP | 0.14 | 2.06 | 0.040292 |
| | LOC_Os07g37920 | NAC | 0.14 | 2.01 | 0.046157 |
| | LOC_Os03g09100 | CAMTA | 0.14 | 2.04 | 0.042146 |
| | LOC_Os08g06140 | NAC | 0.13 | 1.98 | 0.049127 |
| | LOC_Os02g58670 | bZIP | 0.13 | 1.97 | 0.049827 |
| | LOC_Os08g42470 | bHLH | -0.13 | -1.99 | 0.047532 |
| | LOC_Os03g47140 | GRF | -0.13 | -1.98 | 0.048577 |
| | LOC_Os04g56850 | ARF | -0.14 | -2.03 | 0.043199 |
| | LOC_Os02g18370 | FAR1 | -0.14 | -2.09 | 0.037592 |
| | LOC_Os01g09550 | NAC | -0.14 | -2.17 | 0.030865 |
| | LOC_Os06g04090 | NAC | -0.14 | -2.06 | 0.040675 |
| | LOC_Os06g17480 | NF-YB | -0.15 | -1.99 | 0.047763 |
| | LOC_Os05g51830 | C2H2 | -0.15 | -2.20 | 0.029146 |
| | LOC_Os01g63160 | MYB | -0.15 | -2.19 | 0.029399 |
| | LOC_Os01g59350 | bZIP | -0.15 | -2.24 | 0.026261 |
| | LOC_Os06g04850 | HD-ZIP | -0.15 | -2.13 | 0.034259 |
| | LOC_Os01g53040 | WRKY | -0.15 | -2.25 | 0.025636 |
| | LOC_Os03g42630 | NAC | -0.15 | -2.17 | 0.031395 |
| | LOC_Os08g37730 | bHLH | -0.15 | -2.17 | 0.031539 |
| | LOC_Os01g48130 | NAC | -0.15 | -2.25 | 0.025575 |
| | LOC_Os01g54990 | ARF | -0.15 | -2.29 | 0.023095 |
| | LOC_Os02g40070 | AP2 | -0.15 | -2.34 | 0.019944 |
| | LOC_Os02g13310 | TALE | -0.16 | -2.21 | 0.028356 |
| | LOC_Os10g38834 | NAC | -0.16 | -2.17 | 0.031511 |
| | LOC_Os06g08340 | ERF | -0.16 | -2.16 | 0.031907 |
| | LOC_Os01g69910 | CAMTA | -0.16 | -2.25 | 0.025604 |
| | LOC_Os07g48260 | WRKY | -0.16 | -2.33 | 0.020541 |
| | LOC_Os06g01230 | NAC | -0.16 | -2.37 | 0.018848 |
| | LOC_Os07g48596 | G2-like | -0.16 | -2.21 | 0.028586 |
| | LOC_Os05g03884 | TALE | -0.16 | -2.29 | 0.022989 |

| | | | | |
|---|---|---|---|---|
| LOC_Os02g35140 | ARF | -0.16 | -2.36 | 0.019291 |
| LOC_Os06g02560 | GRF | -0.16 | -2.44 | 0.015302 |
| LOC_Os08g33590 | bHLH | -0.16 | -2.46 | 0.014516 |
| LOC_Os08g33750 | G2-like | -0.17 | -2.47 | 0.014213 |
| LOC_Os03g07880 | NF-YA | -0.17 | -2.49 | 0.013654 |
| LOC_Os04g52560 | FAR1 | -0.17 | -2.57 | 0.010697 |
| LOC_Os01g08160 | G2-like | -0.17 | -2.58 | 0.010454 |
| LOC_Os05g38120 | TALE | -0.17 | -2.43 | 0.015932 |
| LOC_Os05g11070 | bHLH | -0.17 | -2.46 | 0.014619 |
| LOC_Os04g52770 | bHLH | -0.17 | -2.42 | 0.01629 |
| LOC_Os04g36054 | ARF | -0.17 | -2.40 | 0.017443 |
| LOC_Os05g02390 | C2H2 | -0.17 | -2.49 | 0.013432 |
| LOC_Os04g57610 | ARF | -0.18 | -2.64 | 0.008946 |
| LOC_Os06g35140 | G2-like | -0.18 | -2.51 | 0.012734 |
| LOC_Os07g43420 | MYB | -0.18 | -2.78 | 0.005946 |
| LOC_Os03g42420 | B3 | -0.18 | -2.62 | 0.009587 |
| LOC_Os01g11350 | bZIP | -0.18 | -2.77 | 0.00611 |
| LOC_Os05g04820 | MYB | -0.18 | -2.67 | 0.008091 |
| LOC_Os06g02230 | B3 | -0.18 | -2.79 | 0.005715 |
| LOC_Os09g33490 | NAC | -0.18 | -2.62 | 0.009501 |
| LOC_Os06g04010 | BBR-BPC | -0.19 | -2.84 | 0.00486 |
| LOC_Os02g15340 | NAC | -0.19 | -2.82 | 0.005174 |
| LOC_Os02g07780 | SBP | -0.19 | -2.66 | 0.008523 |
| LOC_Os01g09850 | C2H2 | -0.19 | -2.63 | 0.009214 |
| LOC_Os06g01480 | NAC | -0.19 | -2.88 | 0.004424 |
| LOC_Os03g08370 | FAR1 | -0.19 | -2.92 | 0.003875 |
| LOC_Os08g19590 | HD-ZIP | -0.19 | -2.76 | 0.00641 |
| LOC_Os01g54210 | GATA | -0.19 | -2.84 | 0.004958 |
| LOC_Os04g43560 | NAC | -0.20 | -2.76 | 0.006377 |
| LOC_Os05g01256 | bHLH | -0.20 | -2.81 | 0.005403 |
| LOC_Os09g10840 | bZIP | -0.20 | -2.80 | 0.005638 |
| LOC_Os02g46780 | MYB | -0.20 | -2.97 | 0.003324 |
| LOC_Os05g02420 | MYB_related | -0.20 | -2.91 | 0.004054 |
| LOC_Os04g54474 | bZIP | -0.20 | -2.86 | 0.004635 |
| LOC_Os12g37970 | MYB | -0.20 | -3.06 | 0.002499 |
| LOC_Os12g42970 | GATA | -0.20 | -2.98 | 0.003253 |
| LOC_Os01g36460 | MYB | -0.20 | -2.85 | 0.004875 |
| LOC_Os03g05500 | LBD | -0.20 | -2.88 | 0.004439 |
| LOC_Os02g08500 | ARR-B | -0.20 | -2.84 | 0.005073 |
| LOC_Os06g08440 | ARR-B | -0.20 | -2.87 | 0.00456 |
| LOC_Os06g03710 | GRAS | -0.20 | -3.11 | 0.002085 |
| LOC_Os02g46560 | bHLH | -0.21 | -3.13 | 0.001991 |
| LOC_Os05g50080 | C3H | -0.21 | -2.99 | 0.003186 |
| LOC_Os01g53260 | WRKY | -0.21 | -3.01 | 0.002988 |
| LOC_Os01g68700 | bHLH | -0.21 | -3.25 | 0.001346 |
| LOC_Os11g06840 | C2H2 | -0.21 | -3.20 | 0.001558 |
| LOC_Os02g12790 | GATA | -0.21 | -3.10 | 0.002235 |
| LOC_Os02g52190 | bHLH | -0.21 | -2.99 | 0.003119 |
| LOC_Os08g41940 | SBP | -0.21 | -3.28 | 0.001185 |
| LOC_Os08g02300 | NAC | -0.21 | -3.25 | 0.00134 |
| LOC_Os07g03770 | TALE | -0.22 | -3.09 | 0.002283 |
| LOC_Os04g36590 | FAR1 | -0.22 | -3.12 | 0.002056 |
| LOC_Os01g03720 | MYB | -0.22 | -3.41 | 0.000768 |
| LOC_Os12g41860 | HD-ZIP | -0.22 | -3.41 | 0.000766 |
| LOC_Os08g09900 | WRKY | -0.23 | -3.42 | 0.00076 |
| LOC_Os01g74410 | MYB | -0.23 | -3.57 | 0.000431 |
| LOC_Os06g49040 | G2-like | -0.24 | -3.42 | 0.000751 |
| LOC_Os01g57650 | C2H2 | -0.24 | -3.48 | 0.000623 |
| LOC_Os02g44360 | GRAS | -0.24 | -3.50 | 0.000577 |
| LOC_Os12g06200 | E2F/DP | -0.24 | -3.76 | 0.000213 |
| LOC_Os05g02150 | Dof | -0.24 | -3.55 | 0.000484 |
| LOC_Os02g34970 | NAC | -0.24 | -3.76 | 0.000215 |
| LOC_Os02g39140 | bHLH | -0.24 | -3.53 | 0.000509 |
| LOC_Os05g50270 | GATA | -0.25 | -3.58 | 0.000431 |
| LOC_Os12g41230 | CPP | -0.25 | -3.85 | 0.000153 |

| | | | | | |
|---|---|---|---|---|---|
| | LOC_Os11g05480 | bZIP | -0.26 | -3.63 | 0.000362 |
| | LOC_Os09g29820 | bZIP | -0.26 | -3.74 | 0.00024 |
| | LOC_Os01g18240 | MYB | -0.26 | -3.84 | 0.000168 |
| | LOC_Os03g04310 | bHLH | -0.27 | -4.06 | 7E-05 |
| | LOC_Os04g50920 | WRKY | -0.27 | -3.95 | 0.000106 |
| | LOC_Os04g50770 | MYB | -0.27 | -3.91 | 0.000129 |
| | LOC_Os05g48850 | NAC | -0.27 | -3.85 | 0.000158 |
| | LOC_Os10g17630 | B3 | -0.28 | -4.28 | 2.84E-05 |
| | LOC_Os07g44640 | C2H2 | -0.29 | -4.50 | 1.12E-05 |
| | LOC_Os01g07480 | LBD | -0.29 | -4.35 | 2.21E-05 |
| | LOC_Os09g36730 | MYB | -0.30 | -4.43 | 1.58E-05 |
| | LOC_Os05g51160 | MYB_related | -0.30 | -4.55 | 9.14E-06 |
| | LOC_Os04g35010 | bHLH | -0.31 | -4.58 | 7.98E-06 |
| | LOC_Os01g50720 | MYB | -0.31 | -4.58 | 8.12E-06 |
| | LOC_Os10g42850 | WRKY | -0.31 | -4.67 | 5.43E-06 |
| | LOC_Os02g50630 | E2F/DP | -0.31 | -4.60 | 7.77E-06 |
| | LOC_Os07g39800 | HRT-like | -0.32 | -5.11 | 6.78E-07 |
| | LOC_Os03g15440 | bHLH | -0.32 | -5.05 | 9.34E-07 |
| | LOC_Os05g20930 | C2H2 | -0.35 | -5.21 | 4.96E-07 |
| | LOC_Os01g17000 | Dof | -0.36 | -5.73 | 3.35E-08 |
| | LOC_Os07g47330 | ERF | -0.37 | -5.45 | 1.57E-07 |
| | LOC_Os06g09420 | B3 | -0.37 | -5.71 | 4.01E-08 |
| | LOC_Os08g33660 | MYB | -0.37 | -6.02 | 7.18E-09 |
| | LOC_Os12g42400 | NF-YA | -0.40 | -6.45 | 6.88E-10 |
| | LOC_Os04g44440 | TCP | -0.43 | -6.91 | 5.57E-11 |
| ABRE_M3 | LOC_Os02g43330 | HD-ZIP | 0.62 | 11.13 | 1.26E-22 |
| | LOC_Os03g60080 | NAC | 0.55 | 9.35 | 1.9E-17 |
| | LOC_Os03g60560 | C2H2 | 0.55 | 9.80 | 4.38E-19 |
| | LOC_Os01g50940 | bHLH | 0.54 | 8.76 | 1.25E-15 |
| | LOC_Os05g37060 | MYB | 0.50 | 8.19 | 2.91E-14 |
| | LOC_Os05g49420 | bZIP | 0.50 | 8.08 | 6.38E-14 |
| | LOC_Os09g28210 | bHLH | 0.48 | 7.98 | 9.96E-14 |
| | LOC_Os02g13800 | HSF | 0.46 | 7.26 | 9.18E-12 |
| | LOC_Os01g63980 | C2H2 | 0.46 | 7.72 | 4.26E-13 |
| | LOC_Os01g64360 | MYB | 0.46 | 7.10 | 2.57E-11 |
| | LOC_Os03g12370 | HSF | 0.45 | 7.66 | 5.18E-13 |
| | LOC_Os02g26430 | WRKY | 0.45 | 7.08 | 2.4E-11 |
| | LOC_Os02g52670 | ERF | 0.45 | 7.42 | 2.52E-12 |
| | LOC_Os06g07030 | ERF | 0.45 | 6.81 | 1.27E-10 |
| | LOC_Os02g32590 | HSF | 0.43 | 6.95 | 4.47E-11 |
| | LOC_Os05g07120 | bHLH | 0.42 | 6.62 | 3E-10 |
| | LOC_Os07g36170 | GRAS | 0.41 | 6.80 | 9.61E-11 |
| | LOC_Os10g41200 | MYB_related | 0.41 | 6.82 | 8.28E-11 |
| | LOC_Os11g03300 | NAC | 0.41 | 6.21 | 3.08E-09 |
| | LOC_Os06g43090 | MYB | 0.40 | 6.19 | 3.37E-09 |
| | LOC_Os09g35790 | HSF | 0.40 | 6.15 | 4.09E-09 |
| | LOC_Os04g45810 | HD-ZIP | 0.40 | 6.41 | 8.84E-10 |
| | LOC_Os01g07120 | ERF | 0.39 | 6.12 | 4.71E-09 |
| | LOC_Os01g48320 | Trihelix | 0.39 | 6.36 | 1.11E-09 |
| | LOC_Os07g48630 | EIL | 0.39 | 6.28 | 1.79E-09 |
| | LOC_Os07g12340 | NAC | 0.38 | 6.10 | 4.73E-09 |
| | LOC_Os05g10670 | C3H | 0.38 | 5.66 | 5.43E-08 |
| | LOC_Os05g34830 | NAC | 0.37 | 5.45 | 1.6E-07 |
| | LOC_Os03g21030 | NAC | 0.37 | 5.66 | 5.13E-08 |
| | LOC_Os07g38090 | C3H | 0.37 | 5.94 | 1.11E-08 |
| | LOC_Os03g55080 | WRKY | 0.37 | 5.99 | 8.46E-09 |
| | LOC_Os06g44010 | WRKY | 0.37 | 5.58 | 7.79E-08 |
| | LOC_Os03g31230 | MYB_related | 0.37 | 5.80 | 2.41E-08 |
| | LOC_Os01g58420 | ERF | 0.37 | 5.91 | 1.29E-08 |
| | LOC_Os02g43790 | ERF | 0.37 | 5.53 | 9.89E-08 |
| | LOC_Os01g64730 | bZIP | 0.36 | 5.70 | 3.85E-08 |
| | LOC_Os04g58020 | MYB_related | 0.36 | 5.62 | 5.96E-08 |
| | LOC_Os08g31580 | ERF | 0.36 | 5.22 | 4.76E-07 |
| | LOC_Os09g28440 | ERF | 0.36 | 5.37 | 2.2E-07 |
| | LOC_Os10g41460 | Trihelix | 0.36 | 5.53 | 9.55E-08 |

| | | | | |
|---|---|---|---|---|
| LOC_Os08g04840 | MYB_related | 0.36 | 5.15 | 6.58E-07 |
| LOC_Os01g15640 | NAC | 0.35 | 5.50 | 1.09E-07 |
| LOC_Os01g40260 | WRKY | 0.35 | 5.31 | 2.98E-07 |
| LOC_Os08g36920 | ERF | 0.35 | 5.20 | 4.94E-07 |
| LOC_Os03g64260 | ERF | 0.35 | 5.20 | 4.86E-07 |
| LOC_Os04g51320 | Trihelix | 0.34 | 5.41 | 1.61E-07 |
| LOC_Os07g39470 | GRAS | 0.34 | 5.27 | 3.28E-07 |
| LOC_Os10g07080 | C2H2 | 0.33 | 4.96 | 1.5E-06 |
| LOC_Os01g21120 | ERF | 0.33 | 5.26 | 3.39E-07 |
| LOC_Os12g37690 | MYB | 0.33 | 5.07 | 8.98E-07 |
| LOC_Os10g41770 | STAT | 0.33 | 5.19 | 4.7E-07 |
| LOC_Os08g36790 | bZIP | 0.33 | 4.92 | 1.8E-06 |
| LOC_Os02g36360 | C2H2 | 0.32 | 5.12 | 6.68E-07 |
| LOC_Os01g67480 | bHLH | 0.32 | 4.90 | 1.95E-06 |
| LOC_Os10g33810 | MYB | 0.32 | 4.66 | 6.04E-06 |
| LOC_Os08g06110 | MYB_related | 0.32 | 5.10 | 7.32E-07 |
| LOC_Os04g46440 | ERF | 0.32 | 5.07 | 8.22E-07 |
| LOC_Os05g41780 | ERF | 0.32 | 5.07 | 8.44E-07 |
| LOC_Os01g39020 | HSF | 0.32 | 4.84 | 2.55E-06 |
| LOC_Os08g15050 | CO-like | 0.31 | 4.59 | 7.78E-06 |
| LOC_Os09g35760 | HD-ZIP | 0.31 | 4.37 | 2.04E-05 |
| LOC_Os01g54600 | WRKY | 0.30 | 4.53 | 1.02E-05 |
| LOC_Os04g41560 | DBB | 0.30 | 4.35 | 2.24E-05 |
| LOC_Os08g01090 | B3 | 0.30 | 4.71 | 4.31E-06 |
| LOC_Os04g32620 | ERF | 0.30 | 4.73 | 4E-06 |
| LOC_Os03g32230 | C2H2 | 0.30 | 4.73 | 4.04E-06 |
| LOC_Os01g72490 | SRS | 0.30 | 4.47 | 1.31E-05 |
| LOC_Os08g38210 | bHLH | 0.30 | 4.75 | 3.57E-06 |
| LOC_Os03g55590 | G2-like | 0.30 | 4.72 | 4.1E-06 |
| LOC_Os07g07974 | CPP | 0.30 | 4.37 | 1.96E-05 |
| LOC_Os03g02160 | C3H | 0.30 | 4.65 | 5.66E-06 |
| LOC_Os01g01870 | bHLH | 0.30 | 4.62 | 6.57E-06 |
| LOC_Os01g14440 | WRKY | 0.29 | 4.58 | 7.87E-06 |
| LOC_Os04g55520 | ERF | 0.29 | 4.56 | 8.37E-06 |
| LOC_Os10g22950 | CAMTA | 0.29 | 4.61 | 6.73E-06 |
| LOC_Os09g38570 | Trihelix | 0.29 | 4.28 | 2.86E-05 |
| LOC_Os01g61080 | WRKY | 0.29 | 4.45 | 1.34E-05 |
| LOC_Os02g44120 | C2H2 | 0.29 | 4.16 | 4.82E-05 |
| LOC_Os09g32040 | NAC | 0.29 | 4.21 | 3.9E-05 |
| LOC_Os01g65900 | GRAS | 0.28 | 4.37 | 1.9E-05 |
| LOC_Os11g06170 | bZIP | 0.28 | 4.11 | 5.69E-05 |
| LOC_Os03g12350 | ARR-B | 0.28 | 4.19 | 4.09E-05 |
| LOC_Os09g28310 | bZIP | 0.28 | 4.30 | 2.59E-05 |
| LOC_Os01g09280 | MYB_related | 0.28 | 4.31 | 2.42E-05 |
| LOC_Os07g48570 | Dof | 0.28 | 4.29 | 2.66E-05 |
| LOC_Os01g70310 | bHLH | 0.27 | 4.27 | 2.82E-05 |
| LOC_Os12g07120 | GATA | 0.27 | 4.16 | 4.61E-05 |
| LOC_Os03g55540 | C2H2 | 0.27 | 4.15 | 4.65E-05 |
| LOC_Os10g41230 | HD-ZIP | 0.27 | 3.75 | 0.000238 |
| LOC_Os03g53020 | bHLH | 0.27 | 4.10 | 5.77E-05 |
| LOC_Os02g08150 | CO-like | 0.27 | 3.88 | 0.00014 |
| LOC_Os04g43680 | MYB | 0.26 | 4.15 | 4.72E-05 |
| LOC_Os02g45450 | ERF | 0.26 | 3.76 | 0.00023 |
| LOC_Os09g35030 | ERF | 0.26 | 3.82 | 0.000178 |
| LOC_Os03g54160 | MIKC_MADS | 0.26 | 3.79 | 0.0002 |
| LOC_Os05g41760 | ERF | 0.26 | 4.00 | 8.59E-05 |
| LOC_Os03g06630 | HSF | 0.26 | 3.98 | 9.35E-05 |
| LOC_Os02g40530 | MYB | 0.26 | 3.98 | 9.29E-05 |
| LOC_Os04g53540 | HD-ZIP | 0.26 | 3.73 | 0.000253 |
| LOC_Os06g45840 | MYB_related | 0.26 | 3.79 | 0.000201 |
| LOC_Os06g07010 | FAR1 | 0.26 | 3.87 | 0.000145 |
| LOC_Os05g03760 | C3H | 0.26 | 3.73 | 0.00025 |
| LOC_Os01g10504 | MIKC_MADS | 0.25 | 3.61 | 0.000387 |
| LOC_Os03g20780 | EIL | 0.25 | 3.67 | 0.000309 |
| LOC_Os03g43840 | LSD | 0.25 | 3.77 | 0.000216 |

| | | | | | |
|---|---|---|---|---|---|
| | LOC_Os03g51330 | GRAS | 0.25 | 3.88 | 0.000139 |
| | LOC_Os02g52340 | MIKC_MADS | 0.25 | 3.74 | 0.000234 |
| | LOC_Os05g09020 | WRKY | 0.24 | 3.45 | 0.000699 |
| | LOC_Os03g48970 | NF-YA | 0.24 | 3.75 | 0.000227 |
| | LOC_Os04g58190 | Dof | 0.24 | 3.70 | 0.000272 |
| | LOC_Os05g37080 | NAC | 0.24 | 3.63 | 0.00035 |
| | LOC_Os04g42020 | CO-like | 0.24 | 3.43 | 0.000734 |
| | LOC_Os09g29930 | bHLH | 0.23 | 3.22 | 0.00154 |
| | LOC_Os10g30719 | MYB_related | 0.23 | 3.28 | 0.001245 |
| | LOC_Os12g21700 | C3H | 0.23 | 3.41 | 0.00078 |
| | LOC_Os02g51670 | ERF | 0.23 | 3.50 | 0.000562 |
| | LOC_Os12g10540 | MIKC_MADS | 0.22 | 3.33 | 0.001027 |
| | LOC_Os05g27930 | ERF | 0.22 | 3.18 | 0.001705 |
| | LOC_Os02g35770 | HD-ZIP | 0.22 | 3.42 | 0.000746 |
| | LOC_Os09g12770 | G2-like | 0.22 | 3.28 | 0.001232 |
| | LOC_Os08g04390 | bHLH | 0.22 | 3.34 | 0.000997 |
| | LOC_Os05g45410 | HSF | 0.22 | 3.10 | 0.002243 |
| | LOC_Os01g32770 | LBD | 0.22 | 3.36 | 0.000914 |
| | LOC_Os05g41450 | NF-YC | 0.22 | 3.14 | 0.001972 |
| | LOC_Os01g70270 | ARF | 0.22 | 3.33 | 0.001006 |
| | LOC_Os03g58160 | HSF | 0.22 | 3.36 | 0.000906 |
| | LOC_Os01g13540 | Nin-like | 0.22 | 3.32 | 0.001044 |
| | LOC_Os04g21950 | WRKY | 0.22 | 3.13 | 0.002033 |
| | LOC_Os09g25060 | WRKY | 0.21 | 3.10 | 0.002187 |
| | LOC_Os05g37190 | C2H2 | 0.21 | 3.27 | 0.001249 |
| | LOC_Os07g48550 | NAC | 0.21 | 3.25 | 0.001315 |
| | LOC_Os04g51560 | WRKY | 0.21 | 3.05 | 0.002617 |
| | LOC_Os01g09620 | C3H | 0.21 | 3.06 | 0.002552 |
| | LOC_Os01g15900 | Dof | 0.21 | 3.14 | 0.001954 |
| | LOC_Os06g45890 | G2-like | 0.21 | 3.01 | 0.002936 |
| | LOC_Os09g31400 | EIL | 0.21 | 2.97 | 0.003342 |
| | LOC_Os01g06320 | MYB_related | 0.21 | 3.13 | 0.001963 |
| | LOC_Os07g22730 | ERF | 0.21 | 2.91 | 0.004036 |
| | LOC_Os04g57600 | C3H | 0.21 | 3.16 | 0.001802 |
| | LOC_Os09g24820 | ZF-HD | 0.21 | 2.88 | 0.004444 |
| | LOC_Os02g05510 | GATA | 0.21 | 3.16 | 0.001772 |
| | LOC_Os01g54550 | HSF | 0.21 | 3.13 | 0.001972 |
| | LOC_Os02g05450 | HB-other | 0.20 | 2.95 | 0.003593 |
| | LOC_Os09g28354 | HSF | 0.20 | 2.88 | 0.004405 |
| | LOC_Os11g29870 | WRKY | 0.20 | 2.88 | 0.0044 |
| | LOC_Os12g41210 | CPP | 0.20 | 3.05 | 0.002593 |
| | LOC_Os03g26210 | bHLH | 0.20 | 3.05 | 0.002557 |
| | LOC_Os01g13460 | bHLH | 0.20 | 2.95 | 0.003529 |
| | LOC_Os03g09100 | CAMTA | 0.20 | 3.02 | 0.002847 |
| | LOC_Os11g39000 | bHLH | 0.20 | 2.97 | 0.003355 |
| | LOC_Os03g21710 | WRKY | 0.20 | 2.82 | 0.005261 |
| | LOC_Os09g35880 | DBB | 0.19 | 2.76 | 0.006306 |
| | LOC_Os07g39310 | C2H2 | 0.19 | 2.79 | 0.005803 |
| | LOC_Os11g02480 | WRKY | 0.19 | 2.67 | 0.008316 |
| | LOC_Os09g01960 | MYB | 0.19 | 2.70 | 0.007633 |
| | LOC_Os07g39480 | WRKY | 0.19 | 2.85 | 0.004801 |
| | LOC_Os03g31240 | C2H2 | 0.19 | 2.77 | 0.006182 |
| | LOC_Os07g48870 | MYB | 0.19 | 2.82 | 0.005239 |
| | LOC_Os01g60270 | WOX | 0.19 | 2.84 | 0.004981 |
| | LOC_Os03g11370 | B3 | 0.18 | 2.61 | 0.009857 |
| | LOC_Os07g01820 | MIKC_MADS | 0.18 | 2.76 | 0.006176 |
| | LOC_Os06g14190 | NF-X1 | 0.18 | 2.61 | 0.009756 |
| | LOC_Os08g14400 | WOX | 0.18 | 2.77 | 0.006023 |
| | LOC_Os01g14870 | C3H | 0.18 | 2.54 | 0.011951 |
| | LOC_Os07g38240 | C2H2 | 0.18 | 2.57 | 0.010827 |
| | LOC_Os01g34060 | MYB_related | 0.18 | 2.53 | 0.012315 |
| | LOC_Os06g41770 | bZIP | 0.18 | 2.56 | 0.011158 |
| | LOC_Os03g11614 | MIKC_MADS | 0.18 | 2.41 | 0.016841 |
| | LOC_Os07g41370 | MIKC_MADS | 0.17 | 2.64 | 0.008907 |
| | LOC_Os01g55340 | Dof | 0.17 | 2.47 | 0.014505 |

| | | | | |
|---|---|---|---|---|
| LOC_Os03g07360 | Dof | 0.17 | 2.58 | 0.010673 |
| LOC_Os12g42610 | YABBY | 0.17 | 2.57 | 0.010742 |
| LOC_Os03g60120 | ERF | 0.17 | 2.55 | 0.01141 |
| LOC_Os02g08540 | bZIP | 0.17 | 2.40 | 0.017486 |
| LOC_Os12g13170 | bZIP | 0.17 | 2.52 | 0.012512 |
| LOC_Os03g08500 | ERF | 0.17 | 2.37 | 0.018804 |
| LOC_Os05g29810 | ERF | 0.17 | 2.27 | 0.024351 |
| LOC_Os01g64310 | NAC | 0.16 | 2.45 | 0.015092 |
| LOC_Os03g17810 | LBD | 0.16 | 2.33 | 0.02107 |
| LOC_Os07g37920 | NAC | 0.16 | 2.41 | 0.016844 |
| LOC_Os07g35870 | bHLH | 0.16 | 2.48 | 0.013806 |
| LOC_Os05g10690 | MYB_related | 0.16 | 2.35 | 0.01984 |
| LOC_Os03g21240 | G2-like | 0.16 | 2.44 | 0.015651 |
| LOC_Os08g06140 | NAC | 0.16 | 2.39 | 0.017678 |
| LOC_Os07g48820 | bZIP | 0.16 | 2.32 | 0.021517 |
| LOC_Os09g30400 | WRKY | 0.16 | 2.30 | 0.022222 |
| LOC_Os05g45020 | C3H | 0.16 | 2.26 | 0.025217 |
| LOC_Os12g40570 | WRKY | 0.16 | 2.43 | 0.015752 |
| LOC_Os04g47990 | Dof | 0.16 | 2.42 | 0.016333 |
| LOC_Os06g12400 | HB-PHD | 0.16 | 2.30 | 0.022736 |
| LOC_Os04g49110 | GRAS | 0.16 | 2.42 | 0.01652 |
| LOC_Os05g11414 | MIKC_MADS | 0.16 | 2.25 | 0.025724 |
| LOC_Os04g46220 | ERF | 0.16 | 2.23 | 0.027026 |
| LOC_Os01g43590 | HSF | 0.16 | 2.29 | 0.023068 |
| LOC_Os01g65080 | C2H2 | 0.16 | 2.39 | 0.017457 |
| LOC_Os01g62660 | G2-like | 0.16 | 2.25 | 0.025629 |
| LOC_Os04g42950 | MYB | 0.16 | 2.24 | 0.026081 |
| LOC_Os02g57650 | NAC | 0.16 | 2.23 | 0.026933 |
| LOC_Os01g68370 | B3 | 0.15 | 2.33 | 0.020809 |
| LOC_Os03g53960 | MYB_related | 0.15 | 2.31 | 0.021947 |
| LOC_Os03g42290 | B3 | 0.15 | 2.30 | 0.022158 |
| LOC_Os03g05690 | C2H2 | 0.15 | 2.28 | 0.023581 |
| LOC_Os08g41030 | ERF | 0.15 | 2.23 | 0.026498 |
| LOC_Os01g13740 | G2-like | 0.15 | 2.28 | 0.023793 |
| LOC_Os09g16510 | WRKY | 0.15 | 2.12 | 0.035314 |
| LOC_Os04g58000 | B3 | 0.15 | 2.23 | 0.026763 |
| LOC_Os03g56580 | NAC | 0.15 | 2.22 | 0.027384 |
| LOC_Os12g10660 | DBB | 0.15 | 2.23 | 0.026696 |
| LOC_Os04g45650 | GATA | 0.15 | 2.20 | 0.028581 |
| LOC_Os03g06860 | FAR1 | 0.15 | 2.17 | 0.031121 |
| LOC_Os01g64000 | bZIP | 0.14 | 2.15 | 0.032401 |
| LOC_Os04g49450 | MYB_related | 0.14 | 2.16 | 0.031518 |
| LOC_Os02g58670 | bZIP | 0.14 | 2.20 | 0.029029 |
| LOC_Os03g08490 | ERF | 0.14 | 2.05 | 0.041662 |
| LOC_Os10g01470 | HD-ZIP | 0.14 | 2.00 | 0.046845 |
| LOC_Os11g06410 | Trihelix | 0.14 | 2.01 | 0.045867 |
| LOC_Os06g09370 | bHLH | 0.14 | 2.02 | 0.044713 |
| LOC_Os03g15010 | FAR1 | 0.14 | 2.05 | 0.041605 |
| LOC_Os02g10840 | FAR1 | 0.14 | 2.05 | 0.041113 |
| LOC_Os10g37240 | Trihelix | 0.13 | 2.02 | 0.044653 |
| LOC_Os11g03370 | NAC | 0.13 | 2.03 | 0.043831 |
| LOC_Os04g46670 | C2H2 | 0.13 | 2.02 | 0.044153 |
| LOC_Os08g17400 | WRKY | 0.13 | 1.99 | 0.047962 |
| LOC_Os10g21560 | NAC | 0.13 | 1.97 | 0.049623 |
| LOC_Os12g12380 | FAR1 | -0.13 | -1.99 | 0.048294 |
| LOC_Os05g51830 | C2H2 | -0.13 | -1.99 | 0.047872 |
| LOC_Os02g47060 | WRKY | -0.14 | -2.04 | 0.042688 |
| LOC_Os01g63460 | MYB | -0.14 | -2.03 | 0.043306 |
| LOC_Os04g57610 | ARF | -0.14 | -2.04 | 0.042961 |
| LOC_Os10g39750 | bHLH | -0.14 | -1.99 | 0.047936 |
| LOC_Os08g42470 | bHLH | -0.14 | -2.09 | 0.03752 |
| LOC_Os01g48130 | NAC | -0.14 | -2.02 | 0.044411 |
| LOC_Os09g36250 | MYB | -0.14 | -1.99 | 0.048485 |
| LOC_Os03g51970 | GRF | -0.14 | -2.09 | 0.037513 |
| LOC_Os09g12750 | G2-like | -0.14 | -2.04 | 0.042359 |

| | | | | | |
|---|---|---|---|---|---|
| | LOC_Os06g08340 | ERF | -0.14 | -1.99 | 0.048354 |
| | LOC_Os03g08370 | FAR1 | -0.15 | -2.19 | 0.029677 |
| | LOC_Os01g59350 | bZIP | -0.15 | -2.25 | 0.025695 |
| | LOC_Os03g58830 | bHLH | -0.15 | -2.14 | 0.0333 |
| | LOC_Os02g44370 | GRAS | -0.15 | -2.13 | 0.034164 |
| | LOC_Os08g37400 | ZF-HD | -0.15 | -2.24 | 0.025967 |
| | LOC_Os10g38834 | NAC | -0.15 | -2.10 | 0.036972 |
| | LOC_Os03g07880 | NF-YA | -0.15 | -2.25 | 0.025791 |
| | LOC_Os03g01890 | HD-ZIP | -0.16 | -2.37 | 0.018805 |
| | LOC_Os07g22770 | ERF | -0.16 | -2.33 | 0.020445 |
| | LOC_Os02g35140 | ARF | -0.16 | -2.27 | 0.024183 |
| | LOC_Os04g36054 | ARF | -0.16 | -2.15 | 0.033184 |
| | LOC_Os02g13310 | TALE | -0.16 | -2.23 | 0.026548 |
| | LOC_Os12g39330 | ERF | -0.16 | -2.18 | 0.030339 |
| | LOC_Os04g46580 | SBP | -0.16 | -2.22 | 0.027355 |
| | LOC_Os03g55220 | bHLH | -0.16 | -2.39 | 0.0178 |
| | LOC_Os03g55760 | G2-like | -0.16 | -2.37 | 0.018866 |
| | LOC_Os06g02230 | B3 | -0.16 | -2.43 | 0.015774 |
| | LOC_Os05g01256 | bHLH | -0.16 | -2.32 | 0.021576 |
| | LOC_Os06g04010 | BBR-BPC | -0.16 | -2.50 | 0.013012 |
| | LOC_Os03g07450 | HD-ZIP | -0.17 | -2.27 | 0.024492 |
| | LOC_Os01g54210 | GATA | -0.17 | -2.53 | 0.012014 |
| | LOC_Os11g05740 | B3 | -0.17 | -2.63 | 0.009233 |
| | LOC_Os05g11070 | bHLH | -0.18 | -2.49 | 0.013589 |
| | LOC_Os06g02560 | GRF | -0.18 | -2.65 | 0.008526 |
| | LOC_Os06g04850 | HD-ZIP | -0.18 | -2.54 | 0.011977 |
| | LOC_Os06g03710 | GRAS | -0.18 | -2.70 | 0.007546 |
| | LOC_Os07g48260 | WRKY | -0.18 | -2.65 | 0.008624 |
| | LOC_Os06g09717 | ERF | -0.18 | -2.53 | 0.012048 |
| | LOC_Os01g08160 | G2-like | -0.18 | -2.72 | 0.006956 |
| | LOC_Os05g41240 | G2-like | -0.18 | -2.60 | 0.01016 |
| | LOC_Os07g48596 | G2-like | -0.18 | -2.52 | 0.012459 |
| | LOC_Os08g33750 | G2-like | -0.19 | -2.81 | 0.005458 |
| | LOC_Os08g19590 | HD-ZIP | -0.19 | -2.66 | 0.008351 |
| | LOC_Os12g42970 | GATA | -0.19 | -2.81 | 0.005464 |
| | LOC_Os04g08060 | C2H2 | -0.19 | -2.72 | 0.007134 |
| | LOC_Os03g46860 | bHLH | -0.19 | -2.76 | 0.006295 |
| | LOC_Os05g38120 | TALE | -0.19 | -2.76 | 0.006325 |
| | LOC_Os06g49040 | G2-like | -0.19 | -2.76 | 0.00642 |
| | LOC_Os02g40070 | AP2 | -0.19 | -2.97 | 0.003264 |
| | LOC_Os09g10840 | bZIP | -0.20 | -2.82 | 0.00524 |
| | LOC_Os05g03884 | TALE | -0.20 | -2.85 | 0.004779 |
| | LOC_Os09g33490 | NAC | -0.20 | -2.88 | 0.004473 |
| | LOC_Os02g15340 | NAC | -0.20 | -3.06 | 0.002477 |
| | LOC_Os01g07480 | LBD | -0.20 | -2.92 | 0.003934 |
| | LOC_Os02g12790 | GATA | -0.20 | -2.94 | 0.00366 |
| | LOC_Os02g34970 | NAC | -0.20 | -3.13 | 0.001987 |
| | LOC_Os12g06200 | E2F/DP | -0.21 | -3.17 | 0.001762 |
| | LOC_Os01g74410 | MYB | -0.21 | -3.15 | 0.001863 |
| | LOC_Os01g09850 | C2H2 | -0.21 | -2.91 | 0.004072 |
| | LOC_Os05g02420 | MYB_related | -0.21 | -3.04 | 0.002647 |
| | LOC_Os06g01480 | NAC | -0.21 | -3.14 | 0.001927 |
| | LOC_Os05g02150 | Dof | -0.21 | -3.02 | 0.002892 |
| | LOC_Os01g11350 | bZIP | -0.21 | -3.17 | 0.001728 |
| | LOC_Os01g53040 | WRKY | -0.21 | -3.18 | 0.001685 |
| | LOC_Os03g42420 | B3 | -0.21 | -3.00 | 0.003027 |
| | LOC_Os08g41940 | SBP | -0.21 | -3.20 | 0.001552 |
| | LOC_Os08g44830 | C2H2 | -0.21 | -3.05 | 0.002576 |
| | LOC_Os06g35140 | G2-like | -0.21 | -3.05 | 0.002569 |
| | LOC_Os04g36590 | FAR1 | -0.22 | -3.08 | 0.002391 |
| | LOC_Os12g37970 | MYB | -0.22 | -3.31 | 0.001091 |
| | LOC_Os07g03770 | TALE | -0.22 | -3.11 | 0.002154 |
| | LOC_Os04g54474 | bZIP | -0.22 | -3.16 | 0.001826 |
| | LOC_Os03g05500 | LBD | -0.22 | -3.15 | 0.001865 |
| | LOC_Os11g06840 | C2H2 | -0.22 | -3.34 | 0.000974 |

| | | | | | |
|---|---|---|---|---|---|
| | LOC_Os08g09900 | WRKY | -0.23 | -3.43 | 0.000727 |
| | LOC_Os06g08440 | ARR-B | -0.23 | -3.25 | 0.00137 |
| | LOC_Os05g50270 | GATA | -0.23 | -3.32 | 0.001057 |
| | LOC_Os08g02300 | NAC | -0.23 | -3.51 | 0.000545 |
| | LOC_Os05g04820 | MYB | -0.23 | -3.42 | 0.000762 |
| | LOC_Os12g41860 | HD-ZIP | -0.23 | -3.58 | 0.000424 |
| | LOC_Os02g39140 | bHLH | -0.24 | -3.39 | 0.000833 |
| | LOC_Os01g03720 | MYB | -0.24 | -3.64 | 0.000343 |
| | LOC_Os04g43560 | NAC | -0.24 | -3.45 | 0.000701 |
| | LOC_Os07g44640 | C2H2 | -0.24 | -3.67 | 0.000305 |
| | LOC_Os02g46560 | bHLH | -0.25 | -3.80 | 0.000185 |
| | LOC_Os02g44360 | GRAS | -0.25 | -3.65 | 0.000332 |
| | LOC_Os12g41230 | CPP | -0.25 | -3.83 | 0.000167 |
| | LOC_Os01g57650 | C2H2 | -0.25 | -3.66 | 0.000321 |
| | LOC_Os05g02390 | C2H2 | -0.26 | -3.71 | 0.000265 |
| | LOC_Os09g29820 | bZIP | -0.26 | -3.70 | 0.000283 |
| | LOC_Os01g53260 | WRKY | -0.26 | -3.71 | 0.000268 |
| | LOC_Os01g68700 | bHLH | -0.26 | -4.00 | 8.65E-05 |
| | LOC_Os05g50080 | C3H | -0.26 | -3.80 | 0.000191 |
| | LOC_Os05g48850 | NAC | -0.26 | -3.78 | 0.000209 |
| | LOC_Os10g17630 | B3 | -0.27 | -3.99 | 9.32E-05 |
| | LOC_Os01g36460 | MYB | -0.27 | -3.84 | 0.000171 |
| | LOC_Os02g52190 | bHLH | -0.27 | -3.82 | 0.000182 |
| | LOC_Os03g42630 | NAC | -0.28 | -4.07 | 6.73E-05 |
| | LOC_Os11g05480 | bZIP | -0.28 | -3.99 | 9.39E-05 |
| | LOC_Os04g50770 | MYB | -0.28 | -4.10 | 6.1E-05 |
| | LOC_Os04g50920 | WRKY | -0.28 | -4.18 | 4.42E-05 |
| | LOC_Os04g35010 | bHLH | -0.28 | -4.22 | 3.72E-05 |
| | LOC_Os03g15440 | bHLH | -0.29 | -4.51 | 1.03E-05 |
| | LOC_Os03g04310 | bHLH | -0.29 | -4.45 | 1.39E-05 |
| | LOC_Os07g39800 | HRT-like | -0.31 | -4.85 | 2.25E-06 |
| | LOC_Os10g42850 | WRKY | -0.31 | -4.62 | 6.91E-06 |
| | LOC_Os02g46780 | MYB | -0.31 | -4.82 | 2.74E-06 |
| | LOC_Os05g51160 | MYB_related | -0.33 | -4.93 | 1.73E-06 |
| | LOC_Os01g18240 | MYB | -0.33 | -4.85 | 2.55E-06 |
| | LOC_Os02g50630 | E2F/DP | -0.34 | -5.04 | 1.04E-06 |
| | LOC_Os01g50720 | MYB | -0.36 | -5.37 | 2.17E-07 |
| | LOC_Os09g36730 | MYB | -0.37 | -5.68 | 4.64E-08 |
| | LOC_Os07g47330 | ERF | -0.37 | -5.54 | 1.03E-07 |
| | LOC_Os06g09420 | B3 | -0.39 | -6.00 | 8.95E-09 |
| | LOC_Os01g17000 | Dof | -0.40 | -6.37 | 1.13E-09 |
| | LOC_Os05g20930 | C2H2 | -0.40 | -6.03 | 8.46E-09 |
| | LOC_Os12g42400 | NF-YA | -0.40 | -6.52 | 4.61E-10 |
| | LOC_Os08g33660 | MYB | -0.40 | -6.63 | 2.53E-10 |
| | LOC_Os04g44440 | TCP | -0.45 | -7.24 | 8.11E-12 |
| ABAVP1 | LOC_Os05g34830 | NAC | 0.50 | 7.77 | 5.37E-13 |
| | LOC_Os07g48550 | NAC | 0.49 | 8.47 | 3.28E-15 |
| | LOC_Os11g03300 | NAC | 0.48 | 7.70 | 6.74E-13 |
| | LOC_Os02g43330 | HD-ZIP | 0.48 | 7.68 | 7.48E-13 |
| | LOC_Os02g13800 | HSF | 0.48 | 7.51 | 2.1E-12 |
| | LOC_Os01g64360 | MYB | 0.47 | 7.18 | 1.59E-11 |
| | LOC_Os03g21030 | NAC | 0.44 | 6.92 | 5.98E-11 |
| | LOC_Os05g37060 | MYB | 0.44 | 6.97 | 4.32E-11 |
| | LOC_Os09g28354 | HSF | 0.42 | 6.51 | 6.18E-10 |
| | LOC_Os01g64000 | bZIP | 0.42 | 6.76 | 1.26E-10 |
| | LOC_Os01g63980 | C2H2 | 0.41 | 6.65 | 2.33E-10 |
| | LOC_Os02g36880 | NAC | 0.41 | 6.22 | 2.96E-09 |
| | LOC_Os01g40260 | WRKY | 0.41 | 6.28 | 2.11E-09 |
| | LOC_Os05g37190 | C2H2 | 0.40 | 6.55 | 4.01E-10 |
| | LOC_Os01g07120 | ERF | 0.40 | 6.22 | 2.72E-09 |
| | LOC_Os01g53650 | C3H | 0.39 | 5.97 | 1.09E-08 |
| | LOC_Os05g48010 | MYB | 0.38 | 5.90 | 1.55E-08 |
| | LOC_Os05g45410 | HSF | 0.38 | 5.70 | 4.67E-08 |
| | LOC_Os06g44010 | WRKY | 0.38 | 5.72 | 3.89E-08 |
| | LOC_Os01g39020 | HSF | 0.38 | 5.90 | 1.42E-08 |

| | | | | | |
|---|---|---|---|---|---|
| | LOC_Os06g07030 | ERF | 0.38 | 5.59 | 8E-08 |
| | LOC_Os11g29870 | WRKY | 0.37 | 5.62 | 6.49E-08 |
| | LOC_Os04g45810 | HD-ZIP | 0.37 | 5.81 | 2.17E-08 |
| | LOC_Os03g32230 | C2H2 | 0.36 | 5.84 | 1.82E-08 |
| | LOC_Os01g61080 | WRKY | 0.36 | 5.76 | 2.89E-08 |
| | LOC_Os03g08460 | ERF | 0.36 | 5.74 | 3.06E-08 |
| | LOC_Os01g09640 | MYB_related | 0.35 | 5.24 | 3.99E-07 |
| | LOC_Os03g60080 | NAC | 0.35 | 5.20 | 4.87E-07 |
| | LOC_Os04g51320 | Trihelix | 0.34 | 5.49 | 1.1E-07 |
| | LOC_Os03g64260 | ERF | 0.34 | 5.08 | 8.74E-07 |
| | LOC_Os01g48320 | Trihelix | 0.34 | 5.33 | 2.37E-07 |
| | LOC_Os11g03370 | NAC | 0.33 | 5.27 | 3.21E-07 |
| | LOC_Os05g45020 | C3H | 0.33 | 4.88 | 2.24E-06 |
| | LOC_Os01g09280 | MYB_related | 0.33 | 5.26 | 3.35E-07 |
| | LOC_Os01g51690 | WRKY | 0.33 | 4.96 | 1.45E-06 |
| | LOC_Os03g20910 | WOX | 0.33 | 5.09 | 7.54E-07 |
| | LOC_Os06g09370 | bHLH | 0.33 | 4.87 | 2.31E-06 |
| | LOC_Os06g44750 | ERF | 0.33 | 4.73 | 4.46E-06 |
| | LOC_Os01g07930 | C3H | 0.32 | 4.79 | 3.32E-06 |
| | LOC_Os08g15050 | CO-like | 0.32 | 4.80 | 3.06E-06 |
| | LOC_Os04g58190 | Dof | 0.32 | 5.06 | 8.87E-07 |
| | LOC_Os07g22730 | ERF | 0.32 | 4.63 | 6.65E-06 |
| | LOC_Os07g48630 | EIL | 0.32 | 4.97 | 1.36E-06 |
| | LOC_Os10g41230 | HD-ZIP | 0.31 | 4.48 | 1.32E-05 |
| | LOC_Os01g09990 | bHLH | 0.31 | 4.67 | 5.46E-06 |
| | LOC_Os01g14440 | WRKY | 0.31 | 4.93 | 1.61E-06 |
| | LOC_Os06g45890 | G2-like | 0.31 | 4.61 | 7.31E-06 |
| | LOC_Os08g14400 | WOX | 0.31 | 4.86 | 2.25E-06 |
| | LOC_Os09g28440 | ERF | 0.31 | 4.55 | 9.15E-06 |
| | LOC_Os01g54600 | WRKY | 0.31 | 4.61 | 7.12E-06 |
| | LOC_Os07g42510 | ERF | 0.31 | 4.61 | 7.15E-06 |
| | LOC_Os02g26430 | WRKY | 0.31 | 4.55 | 9.48E-06 |
| | LOC_Os07g12340 | NAC | 0.30 | 4.79 | 2.99E-06 |
| | LOC_Os01g64730 | bZIP | 0.30 | 4.70 | 4.68E-06 |
| | LOC_Os03g12370 | HSF | 0.30 | 4.83 | 2.46E-06 |
| | LOC_Os04g43680 | MYB | 0.30 | 4.81 | 2.78E-06 |
| | LOC_Os08g02070 | MIKC_MADS | 0.30 | 4.67 | 5.33E-06 |
| | LOC_Os02g36360 | C2H2 | 0.30 | 4.65 | 5.69E-06 |
| | LOC_Os10g26620 | Dof | 0.30 | 4.68 | 5.02E-06 |
| | LOC_Os04g55970 | AP2 | 0.29 | 4.56 | 8.55E-06 |
| | LOC_Os01g43650 | WRKY | 0.29 | 4.57 | 8.12E-06 |
| | LOC_Os05g34050 | bZIP | 0.29 | 4.30 | 2.67E-05 |
| | LOC_Os02g56600 | NAC | 0.29 | 4.46 | 1.28E-05 |
| | LOC_Os01g50110 | MYB | 0.28 | 4.28 | 2.86E-05 |
| | LOC_Os10g22430 | GRAS | 0.28 | 4.42 | 1.5E-05 |
| | LOC_Os04g46220 | ERF | 0.28 | 4.07 | 6.93E-05 |
| | LOC_Os02g43790 | ERF | 0.28 | 4.12 | 5.6E-05 |
| | LOC_Os03g21710 | WRKY | 0.28 | 4.13 | 5.31E-05 |
| | LOC_Os03g17810 | LBD | 0.28 | 4.07 | 6.93E-05 |
| | LOC_Os09g16510 | WRKY | 0.28 | 4.11 | 5.71E-05 |
| | LOC_Os07g37920 | NAC | 0.28 | 4.21 | 3.79E-05 |
| | LOC_Os05g29810 | ERF | 0.28 | 3.90 | 0.000134 |
| | LOC_Os06g45140 | bZIP | 0.27 | 3.97 | 9.96E-05 |
| | LOC_Os01g39330 | bHLH | 0.27 | 4.02 | 8.4E-05 |
| | LOC_Os04g46440 | ERF | 0.27 | 4.21 | 3.72E-05 |
| | LOC_Os03g60560 | C2H2 | 0.27 | 4.21 | 3.75E-05 |
| | LOC_Os10g33810 | MYB | 0.27 | 3.84 | 0.00017 |
| | LOC_Os01g68370 | B3 | 0.27 | 4.19 | 4.11E-05 |
| | LOC_Os05g07120 | bHLH | 0.27 | 4.06 | 7.02E-05 |
| | LOC_Os05g49420 | bZIP | 0.27 | 3.93 | 0.000118 |
| | LOC_Os07g48820 | bZIP | 0.27 | 3.96 | 0.000104 |
| | LOC_Os03g17150 | C2H2 | 0.27 | 3.99 | 9.07E-05 |
| | LOC_Os09g10840 | bZIP | 0.27 | 3.87 | 0.000147 |
| | LOC_Os03g53020 | bHLH | 0.27 | 4.11 | 5.51E-05 |
| | LOC_Os05g10670 | C3H | 0.27 | 3.87 | 0.000149 |

| | | | | |
|---|---|---|---|---|
| LOC_Os01g43550 | WRKY | 0.27 | 4.13 | 5.05E-05 |
| LOC_Os06g12230 | TCP | 0.27 | 3.87 | 0.000146 |
| LOC_Os02g08500 | ARR-B | 0.27 | 3.77 | 0.000217 |
| LOC_Os08g39630 | bHLH | 0.27 | 3.93 | 0.000114 |
| LOC_Os03g08490 | ERF | 0.26 | 3.85 | 0.000157 |
| LOC_Os02g52670 | ERF | 0.26 | 4.04 | 7.46E-05 |
| LOC_Os04g58000 | B3 | 0.26 | 4.04 | 7.49E-05 |
| LOC_Os05g37080 | NAC | 0.26 | 4.00 | 8.81E-05 |
| LOC_Os03g22170 | ERF | 0.26 | 4.03 | 7.52E-05 |
| LOC_Os01g15640 | NAC | 0.26 | 3.90 | 0.000131 |
| LOC_Os07g36170 | GRAS | 0.26 | 3.99 | 8.8E-05 |
| LOC_Os07g48870 | MYB | 0.26 | 3.97 | 9.64E-05 |
| LOC_Os03g10210 | HD-ZIP | 0.26 | 3.71 | 0.000271 |
| LOC_Os03g07880 | NF-YA | 0.26 | 3.87 | 0.000147 |
| LOC_Os01g21120 | ERF | 0.25 | 3.94 | 0.000111 |
| LOC_Os03g26210 | bHLH | 0.25 | 3.93 | 0.000115 |
| LOC_Os01g09590 | MYB | 0.25 | 3.62 | 0.000369 |
| LOC_Os09g25070 | WRKY | 0.25 | 3.84 | 0.000158 |
| LOC_Os04g49450 | MYB_related | 0.25 | 3.75 | 0.00023 |
| LOC_Os11g02480 | WRKY | 0.25 | 3.48 | 0.000632 |
| LOC_Os03g42430 | B3 | 0.24 | 3.69 | 0.00028 |
| LOC_Os08g41030 | ERF | 0.24 | 3.64 | 0.000345 |
| LOC_Os06g01230 | NAC | 0.24 | 3.63 | 0.000361 |
| LOC_Os12g41680 | NAC | 0.24 | 3.62 | 0.000366 |
| LOC_Os09g35790 | HSF | 0.24 | 3.49 | 0.000597 |
| LOC_Os02g52340 | MIKC_MADS | 0.24 | 3.57 | 0.000437 |
| LOC_Os01g09100 | WRKY | 0.24 | 3.46 | 0.000666 |
| LOC_Os03g55080 | WRKY | 0.24 | 3.65 | 0.000327 |
| LOC_Os03g03900 | Nin-like | 0.24 | 3.50 | 0.000561 |
| LOC_Os01g11910 | bHLH | 0.23 | 3.27 | 0.001286 |
| LOC_Os02g12310 | NAC | 0.23 | 3.52 | 0.000517 |
| LOC_Os04g58020 | MYB_related | 0.23 | 3.37 | 0.00088 |
| LOC_Os02g43970 | ERF | 0.23 | 3.15 | 0.001915 |
| LOC_Os04g40140 | NAC | 0.23 | 3.28 | 0.001212 |
| LOC_Os10g01470 | HD-ZIP | 0.23 | 3.22 | 0.001506 |
| LOC_Os08g04840 | MYB_related | 0.22 | 3.12 | 0.002095 |
| LOC_Os07g39470 | GRAS | 0.22 | 3.36 | 0.000918 |
| LOC_Os05g41760 | ERF | 0.22 | 3.37 | 0.000898 |
| LOC_Os12g01490 | G2-like | 0.22 | 3.22 | 0.001474 |
| LOC_Os10g42130 | NAC | 0.22 | 3.12 | 0.002101 |
| LOC_Os04g38720 | NAC | 0.22 | 3.21 | 0.001565 |
| LOC_Os09g31390 | bZIP | 0.22 | 3.15 | 0.00191 |
| LOC_Os10g21560 | NAC | 0.22 | 3.34 | 0.000978 |
| LOC_Os01g47560 | WRKY | 0.22 | 3.30 | 0.001127 |
| LOC_Os02g35770 | HD-ZIP | 0.22 | 3.28 | 0.001198 |
| LOC_Os02g47190 | G2-like | 0.21 | 3.12 | 0.002046 |
| LOC_Os04g41560 | DBB | 0.21 | 2.98 | 0.003271 |
| LOC_Os08g39980 | MYB_related | 0.21 | 3.23 | 0.001444 |
| LOC_Os01g06640 | bHLH | 0.21 | 3.21 | 0.001546 |
| LOC_Os03g15660 | ERF | 0.21 | 3.16 | 0.00182 |
| LOC_Os11g05614 | NAC | 0.21 | 2.98 | 0.00328 |
| LOC_Os02g15350 | Dof | 0.21 | 3.00 | 0.003064 |
| LOC_Os09g35910 | HD-ZIP | 0.21 | 2.91 | 0.00408 |
| LOC_Os03g45300 | FAR1 | 0.21 | 3.08 | 0.002322 |
| LOC_Os01g15900 | Dof | 0.20 | 3.04 | 0.002684 |
| LOC_Os01g13740 | G2-like | 0.20 | 3.07 | 0.002406 |
| LOC_Os01g09550 | NAC | 0.20 | 3.06 | 0.0025 |
| LOC_Os06g04090 | NAC | 0.20 | 2.83 | 0.005117 |
| LOC_Os10g25170 | ERF | 0.20 | 2.99 | 0.003134 |
| LOC_Os09g25060 | WRKY | 0.20 | 2.83 | 0.005184 |
| LOC_Os01g18870 | bHLH | 0.19 | 2.93 | 0.003782 |
| LOC_Os10g41200 | MYB_related | 0.19 | 2.96 | 0.003456 |
| LOC_Os08g29660 | WRKY | 0.19 | 2.93 | 0.003697 |
| LOC_Os05g25260 | ERF | 0.19 | 2.72 | 0.007062 |
| LOC_Os03g29614 | MYB | 0.19 | 2.63 | 0.009192 |

| | | | | | |
|---|---|---|---|---|---|
| | LOC_Os03g37920 | FAR1 | 0.19 | 2.87 | 0.004527 |
| | LOC_Os01g52540 | B3 | 0.19 | 2.65 | 0.008709 |
| | LOC_Os09g28210 | bHLH | 0.19 | 2.73 | 0.006855 |
| | LOC_Os04g32620 | ERF | 0.19 | 2.82 | 0.005182 |
| | LOC_Os08g36920 | ERF | 0.18 | 2.65 | 0.008749 |
| | LOC_Os01g58420 | ERF | 0.18 | 2.81 | 0.005362 |
| | LOC_Os02g39140 | bHLH | 0.18 | 2.63 | 0.009222 |
| | LOC_Os04g47860 | C2H2 | 0.18 | 2.77 | 0.006021 |
| | LOC_Os04g45690 | DBB | 0.18 | 2.76 | 0.006202 |
| | LOC_Os03g55540 | C2H2 | 0.18 | 2.73 | 0.006768 |
| | LOC_Os02g29550 | ERF | 0.18 | 2.59 | 0.01032 |
| | LOC_Os02g32590 | HSF | 0.18 | 2.65 | 0.008731 |
| | LOC_Os04g51560 | WRKY | 0.18 | 2.56 | 0.011339 |
| | LOC_Os03g51330 | GRAS | 0.18 | 2.72 | 0.007133 |
| | LOC_Os05g49620 | WRKY | 0.18 | 2.56 | 0.011165 |
| | LOC_Os01g51610 | B3 | 0.18 | 2.58 | 0.010527 |
| | LOC_Os03g20900 | G2-like | 0.17 | 2.66 | 0.008258 |
| | LOC_Os08g10080 | NAC | 0.17 | 2.48 | 0.014137 |
| | LOC_Os03g08930 | bHLH | 0.17 | 2.38 | 0.018173 |
| | LOC_Os02g49986 | MYB | 0.17 | 2.37 | 0.018972 |
| | LOC_Os01g53040 | WRKY | 0.17 | 2.58 | 0.010581 |
| | LOC_Os08g02300 | NAC | 0.17 | 2.54 | 0.011834 |
| | LOC_Os06g11970 | M-type_MADS | 0.17 | 2.39 | 0.017717 |
| | LOC_Os09g38340 | C2H2 | 0.17 | 2.37 | 0.018908 |
| | LOC_Os08g44820 | NAC | 0.16 | 2.49 | 0.013648 |
| | LOC_Os01g53260 | WRKY | 0.16 | 2.34 | 0.020314 |
| | LOC_Os08g43334 | HSF | 0.16 | 2.27 | 0.02409 |
| | LOC_Os10g38820 | bZIP | 0.16 | 2.32 | 0.021172 |
| | LOC_Os08g34360 | ERF | 0.16 | 2.32 | 0.021566 |
| | LOC_Os09g32040 | NAC | 0.16 | 2.30 | 0.02258 |
| | LOC_Os04g53990 | bHLH | 0.16 | 2.43 | 0.015866 |
| | LOC_Os12g07120 | GATA | 0.16 | 2.40 | 0.017043 |
| | LOC_Os01g48130 | NAC | 0.16 | 2.30 | 0.022243 |
| | LOC_Os01g70110 | NAC | 0.16 | 2.16 | 0.031869 |
| | LOC_Os01g70310 | bHLH | 0.16 | 2.37 | 0.018853 |
| | LOC_Os08g36790 | bZIP | 0.16 | 2.21 | 0.028591 |
| | LOC_Os05g34310 | NAC | 0.15 | 2.20 | 0.028784 |
| | LOC_Os01g60270 | WOX | 0.15 | 2.32 | 0.021003 |
| | LOC_Os07g49530 | MYB_related | 0.15 | 2.26 | 0.024988 |
| | LOC_Os03g09100 | CAMTA | 0.15 | 2.28 | 0.023829 |
| | LOC_Os01g14420 | Nin-like | 0.15 | 2.10 | 0.037383 |
| | LOC_Os10g27360 | NAC | 0.15 | 2.17 | 0.030765 |
| | LOC_Os02g44130 | C2H2 | 0.15 | 2.06 | 0.040787 |
| | LOC_Os07g41720 | NF-YA | 0.14 | 2.15 | 0.032841 |
| | LOC_Os09g19950 | LBD | 0.14 | 1.98 | 0.049569 |
| | LOC_Os06g43090 | MYB | 0.14 | 2.04 | 0.042484 |
| | LOC_Os03g56580 | NAC | 0.14 | 2.12 | 0.035449 |
| | LOC_Os03g48970 | NF-YA | 0.14 | 2.08 | 0.038918 |
| | LOC_Os03g12350 | ARR-B | 0.14 | 2.02 | 0.044503 |
| | LOC_Os02g02820 | bHLH | 0.14 | 2.07 | 0.03925 |
| | LOC_Os06g03710 | GRAS | 0.13 | 1.99 | 0.047294 |
| | LOC_Os12g41920 | MYB_related | -0.13 | -1.97 | 0.049866 |
| | LOC_Os08g01330 | NAC | -0.13 | -1.97 | 0.04999 |
| | LOC_Os03g08960 | HD-ZIP | -0.13 | -1.98 | 0.049409 |
| | LOC_Os04g50060 | GRAS | -0.13 | -2.02 | 0.045027 |
| | LOC_Os01g14720 | GeBP | -0.13 | -1.99 | 0.047341 |
| | LOC_Os12g41230 | CPP | -0.13 | -1.99 | 0.047636 |
| | LOC_Os03g63530 | NF-YC | -0.13 | -2.02 | 0.044795 |
| | LOC_Os04g45650 | GATA | -0.14 | -2.04 | 0.042101 |
| | LOC_Os08g33660 | MYB | -0.14 | -2.09 | 0.037868 |
| | LOC_Os08g41940 | SBP | -0.14 | -2.10 | 0.036959 |
| | LOC_Os02g44370 | GRAS | -0.14 | -1.98 | 0.048571 |
| | LOC_Os02g47660 | bHLH | -0.14 | -2.05 | 0.041566 |
| | LOC_Os09g29460 | HD-ZIP | -0.14 | -1.99 | 0.048522 |
| | LOC_Os05g41070 | bZIP | -0.14 | -1.99 | 0.047537 |

| | | | | |
|---|---|---|---|---|
| LOC_Os01g63460 | MYB | -0.14 | -2.13 | 0.034222 |
| LOC_Os07g39220 | BES1 | -0.14 | -2.15 | 0.032892 |
| LOC_Os03g47740 | TALE | -0.14 | -2.17 | 0.03118 |
| LOC_Os01g06320 | MYB_related | -0.15 | -2.17 | 0.030929 |
| LOC_Os01g68860 | C3H | -0.15 | -2.21 | 0.027814 |
| LOC_Os12g06380 | FAR1 | -0.15 | -2.19 | 0.029555 |
| LOC_Os08g04170 | C3H | -0.15 | -2.24 | 0.026104 |
| LOC_Os01g69850 | M-type_MADS | -0.15 | -2.06 | 0.040704 |
| LOC_Os12g42610 | YABBY | -0.15 | -2.27 | 0.024114 |
| LOC_Os09g13940 | ERF | -0.15 | -2.14 | 0.033582 |
| LOC_Os01g62660 | G2-like | -0.15 | -2.15 | 0.032795 |
| LOC_Os03g13790 | MYB_related | -0.15 | -2.27 | 0.023985 |
| LOC_Os07g35870 | bHLH | -0.15 | -2.28 | 0.023281 |
| LOC_Os11g30484 | C2H2 | -0.15 | -2.16 | 0.031912 |
| LOC_Os02g35140 | ARF | -0.15 | -2.22 | 0.027282 |
| LOC_Os02g33750 | FAR1 | -0.15 | -2.21 | 0.028347 |
| LOC_Os01g64560 | bHLH | -0.16 | -2.34 | 0.020186 |
| LOC_Os09g12380 | NAC | -0.16 | -2.15 | 0.032475 |
| LOC_Os03g50900 | FAR1 | -0.16 | -2.36 | 0.019374 |
| LOC_Os06g14190 | NF-X1 | -0.16 | -2.29 | 0.022819 |
| LOC_Os01g09760 | MYB_related | -0.16 | -2.45 | 0.015168 |
| LOC_Os06g41384 | C3H | -0.16 | -2.22 | 0.027313 |
| LOC_Os08g39830 | EIL | -0.16 | -2.28 | 0.023594 |
| LOC_Os05g43760 | TCP | -0.16 | -2.26 | 0.024759 |
| LOC_Os01g68160 | C2H2 | -0.16 | -2.38 | 0.018344 |
| LOC_Os12g41860 | HD-ZIP | -0.16 | -2.47 | 0.014296 |
| LOC_Os03g58330 | bHLH | -0.17 | -2.42 | 0.016505 |
| LOC_Os07g28430 | GRF | -0.17 | -2.38 | 0.01804 |
| LOC_Os02g29340 | HSF | -0.17 | -2.54 | 0.011647 |
| LOC_Os03g63750 | HSF | -0.17 | -2.53 | 0.012198 |
| LOC_Os05g11414 | MIKC_MADS | -0.17 | -2.38 | 0.018156 |
| LOC_Os07g47330 | ERF | -0.17 | -2.36 | 0.019488 |
| LOC_Os07g44200 | GeBP | -0.17 | -2.59 | 0.010288 |
| LOC_Os04g40060 | FAR1 | -0.17 | -2.59 | 0.010238 |
| LOC_Os03g08370 | FAR1 | -0.17 | -2.58 | 0.010549 |
| LOC_Os03g11370 | B3 | -0.17 | -2.43 | 0.015886 |
| LOC_Os08g44830 | C2H2 | -0.17 | -2.49 | 0.013483 |
| LOC_Os11g32100 | bHLH | -0.17 | -2.50 | 0.013262 |
| LOC_Os12g42970 | GATA | -0.17 | -2.58 | 0.010585 |
| LOC_Os07g08140 | HSF | -0.17 | -2.63 | 0.009195 |
| LOC_Os05g35170 | NAC | -0.18 | -2.52 | 0.012641 |
| LOC_Os08g23470 | B3 | -0.18 | -2.53 | 0.012221 |
| LOC_Os10g42490 | HD-ZIP | -0.18 | -2.67 | 0.008113 |
| LOC_Os04g55560 | AP2 | -0.18 | -2.67 | 0.008266 |
| LOC_Os02g42870 | MYB | -0.18 | -2.55 | 0.011367 |
| LOC_Os07g41370 | MIKC_MADS | -0.18 | -2.72 | 0.007001 |
| LOC_Os06g11860 | ERF | -0.18 | -2.54 | 0.011996 |
| LOC_Os03g50110 | GeBP | -0.18 | -2.73 | 0.006887 |
| LOC_Os03g60120 | ERF | -0.18 | -2.75 | 0.006488 |
| LOC_Os06g40150 | ERF | -0.18 | -2.60 | 0.010158 |
| LOC_Os11g47900 | GRAS | -0.18 | -2.55 | 0.011469 |
| LOC_Os01g42970 | C3H | -0.18 | -2.58 | 0.010613 |
| LOC_Os03g13600 | C2H2 | -0.18 | -2.73 | 0.006787 |
| LOC_Os02g49480 | bHLH | -0.18 | -2.70 | 0.0074 |
| LOC_Os08g37904 | C2H2 | -0.18 | -2.58 | 0.010593 |
| LOC_Os06g47150 | ARF | -0.19 | -2.63 | 0.009173 |
| LOC_Os02g58440 | C3H | -0.19 | -2.81 | 0.005421 |
| LOC_Os09g24820 | ZF-HD | -0.19 | -2.61 | 0.00991 |
| LOC_Os08g41950 | MIKC_MADS | -0.19 | -2.66 | 0.008451 |
| LOC_Os01g55150 | bZIP | -0.19 | -2.87 | 0.004537 |
| LOC_Os01g63160 | MYB | -0.19 | -2.83 | 0.005086 |
| LOC_Os01g18240 | MYB | -0.19 | -2.71 | 0.007268 |
| LOC_Os03g07450 | HD-ZIP | -0.19 | -2.64 | 0.00912 |
| LOC_Os04g52560 | FAR1 | -0.19 | -2.94 | 0.003578 |
| LOC_Os12g06200 | E2F/DP | -0.19 | -2.99 | 0.0031 |

| | | | | | |
|---|---|---|---|---|---|
| | LOC_Os08g25799 | G2-like | -0.20 | -2.84 | 0.004934 |
| | LOC_Os05g03020 | C2H2 | -0.20 | -2.80 | 0.005699 |
| | LOC_Os01g15460 | C3H | -0.20 | -2.98 | 0.003223 |
| | LOC_Os01g67970 | C2H2 | -0.20 | -2.85 | 0.004758 |
| | LOC_Os12g37410 | bZIP | -0.20 | -2.86 | 0.004633 |
| | LOC_Os04g11830 | TCP | -0.20 | -2.82 | 0.005257 |
| | LOC_Os01g07480 | LBD | -0.20 | -2.88 | 0.004431 |
| | LOC_Os01g69910 | CAMTA | -0.20 | -2.86 | 0.004704 |
| | LOC_Os05g25320 | FAR1 | -0.20 | -2.89 | 0.004336 |
| | LOC_Os06g08440 | ARR-B | -0.20 | -2.85 | 0.004835 |
| | LOC_Os02g12790 | GATA | -0.20 | -2.92 | 0.003923 |
| | LOC_Os06g02560 | GRF | -0.20 | -3.08 | 0.002316 |
| | LOC_Os05g04820 | MYB | -0.21 | -3.01 | 0.002901 |
| | LOC_Os01g55750 | TCP | -0.21 | -3.04 | 0.002697 |
| | LOC_Os09g33580 | bHLH | -0.21 | -2.94 | 0.003691 |
| | LOC_Os01g13520 | ARF | -0.21 | -3.19 | 0.001643 |
| | LOC_Os11g05740 | B3 | -0.21 | -3.24 | 0.001399 |
| | LOC_Os01g68560 | M-type_MADS | -0.21 | -3.23 | 0.001447 |
| | LOC_Os04g35800 | C3H | -0.22 | -3.13 | 0.001998 |
| | LOC_Os11g09160 | B3 | -0.22 | -3.14 | 0.001922 |
| | LOC_Os02g03960 | bZIP | -0.22 | -3.16 | 0.001831 |
| | LOC_Os02g03580 | bZIP | -0.22 | -3.17 | 0.001738 |
| | LOC_Os02g06370 | Whirly | -0.22 | -3.18 | 0.001716 |
| | LOC_Os07g48660 | bZIP | -0.22 | -3.38 | 0.000871 |
| | LOC_Os07g25710 | G2-like | -0.22 | -3.39 | 0.000837 |
| | LOC_Os04g46580 | SBP | -0.22 | -3.17 | 0.001796 |
| | LOC_Os05g41540 | bZIP | -0.22 | -3.22 | 0.001509 |
| | LOC_Os02g39540 | FAR1 | -0.23 | -3.47 | 0.000635 |
| | LOC_Os09g11460 | ERF | -0.23 | -3.49 | 0.000592 |
| | LOC_Os11g11100 | bZIP | -0.23 | -3.32 | 0.00109 |
| | LOC_Os07g42400 | FAR1 | -0.23 | -3.54 | 0.000482 |
| | LOC_Os02g53670 | MYB_related | -0.23 | -3.57 | 0.000431 |
| | LOC_Os01g15350 | C3H | -0.23 | -3.63 | 0.000349 |
| | LOC_Os04g36590 | FAR1 | -0.24 | -3.39 | 0.000856 |
| | LOC_Os02g07780 | SBP | -0.24 | -3.39 | 0.000833 |
| | LOC_Os03g01890 | HD-ZIP | -0.24 | -3.68 | 0.000295 |
| | LOC_Os01g48700 | E2F/DP | -0.24 | -3.51 | 0.000545 |
| | LOC_Os09g12750 | G2-like | -0.24 | -3.47 | 0.000649 |
| | LOC_Os12g12380 | FAR1 | -0.24 | -3.67 | 0.00031 |
| | LOC_Os08g40900 | ARF | -0.24 | -3.51 | 0.00055 |
| | LOC_Os09g01140 | GeBP | -0.24 | -3.48 | 0.000625 |
| | LOC_Os09g24810 | ZF-HD | -0.24 | -3.45 | 0.000683 |
| | LOC_Os04g47890 | G2-like | -0.24 | -3.78 | 0.000202 |
| | LOC_Os04g08060 | C2H2 | -0.25 | -3.55 | 0.000475 |
| | LOC_Os09g13570 | bZIP | -0.25 | -3.59 | 0.000423 |
| | LOC_Os02g18370 | FAR1 | -0.25 | -3.82 | 0.000174 |
| | LOC_Os02g06910 | ARF | -0.25 | -3.92 | 0.000116 |
| | LOC_Os04g46020 | GATA | -0.25 | -3.85 | 0.000157 |
| | LOC_Os04g02730 | C3H | -0.25 | -3.69 | 0.000285 |
| | LOC_Os01g11350 | bZIP | -0.26 | -3.91 | 0.000121 |
| | LOC_Os04g43910 | ARF | -0.26 | -3.83 | 0.000171 |
| | LOC_Os08g37580 | HD-ZIP | -0.26 | -3.91 | 0.000124 |
| | LOC_Os10g41260 | MYB_related | -0.26 | -3.87 | 0.000144 |
| | LOC_Os10g40390 | GRAS | -0.26 | -3.72 | 0.000268 |
| | LOC_Os01g68700 | bHLH | -0.26 | -4.06 | 6.86E-05 |
| | LOC_Os12g29520 | ARF | -0.26 | -4.08 | 6.2E-05 |
| | LOC_Os07g43420 | MYB | -0.27 | -4.15 | 4.7E-05 |
| | LOC_Os06g16400 | bHLH | -0.27 | -4.06 | 6.71E-05 |
| | LOC_Os03g56110 | TALE | -0.27 | -3.88 | 0.000143 |
| | LOC_Os03g02900 | B3 | -0.27 | -4.16 | 4.59E-05 |
| | LOC_Os05g11510 | DBB | -0.27 | -4.01 | 8.54E-05 |
| | LOC_Os12g18150 | C2H2 | -0.27 | -4.29 | 2.65E-05 |
| | LOC_Os10g41130 | ERF | -0.28 | -4.32 | 2.32E-05 |
| | LOC_Os02g45850 | B3 | -0.28 | -4.03 | 7.92E-05 |
| | LOC_Os02g33610 | Trihelix | -0.28 | -3.93 | 0.000118 |

| | | | | | |
|---|---|---|---|---|---|
| | LOC_Os07g44640 | C2H2 | -0.28 | -4.23 | 3.39E-05 |
| | LOC_Os02g18660 | GeBP | -0.28 | -4.07 | 6.83E-05 |
| | LOC_Os03g54170 | MIKC_MADS | -0.28 | -4.28 | 2.8E-05 |
| | LOC_Os12g06640 | Trihelix | -0.28 | -4.44 | 1.39E-05 |
| | LOC_Os06g40960 | C2H2 | -0.28 | -4.15 | 5.02E-05 |
| | LOC_Os12g41950 | ARF | -0.29 | -4.42 | 1.61E-05 |
| | LOC_Os11g35030 | GRF | -0.30 | -4.38 | 1.96E-05 |
| | LOC_Os06g09420 | B3 | -0.30 | -4.47 | 1.3E-05 |
| | LOC_Os08g43160 | TCP | -0.30 | -4.35 | 2.23E-05 |
| | LOC_Os03g42280 | B3 | -0.30 | -4.46 | 1.34E-05 |
| | LOC_Os02g47060 | WRKY | -0.30 | -4.69 | 4.71E-06 |
| | LOC_Os02g49440 | Dof | -0.30 | -4.73 | 4.05E-06 |
| | LOC_Os02g42380 | TCP | -0.30 | -4.74 | 3.9E-06 |
| | LOC_Os04g28090 | MYB | -0.31 | -4.78 | 3.14E-06 |
| | LOC_Os05g41240 | G2-like | -0.31 | -4.51 | 1.12E-05 |
| | LOC_Os07g13170 | AP2 | -0.31 | -4.53 | 1.01E-05 |
| | LOC_Os03g27390 | bHLH | -0.31 | -4.85 | 2.31E-06 |
| | LOC_Os03g63810 | WRKY | -0.32 | -4.65 | 6.06E-06 |
| | LOC_Os06g04850 | HD-ZIP | -0.32 | -4.81 | 2.97E-06 |
| | LOC_Os06g40710 | G2-like | -0.33 | -4.90 | 1.97E-06 |
| | LOC_Os06g02230 | B3 | -0.33 | -5.21 | 4.37E-07 |
| | LOC_Os03g55220 | bHLH | -0.34 | -5.29 | 2.95E-07 |
| | LOC_Os05g50080 | C3H | -0.34 | -5.15 | 6.17E-07 |
| | LOC_Os03g12120 | NAC | -0.35 | -5.62 | 5.71E-08 |
| | LOC_Os06g04870 | HD-ZIP | -0.36 | -5.68 | 4.25E-08 |
| | LOC_Os01g32890 | GeBP | -0.36 | -5.46 | 1.4E-07 |
| | LOC_Os12g42400 | NF-YA | -0.36 | -5.75 | 2.88E-08 |
| | LOC_Os06g46410 | ARF | -0.36 | -5.39 | 2E-07 |
| | LOC_Os08g19590 | HD-ZIP | -0.36 | -5.44 | 1.58E-07 |
| | LOC_Os06g49010 | SBP | -0.37 | -5.39 | 2.05E-07 |
| | LOC_Os05g37730 | MYB | -0.39 | -5.88 | 1.83E-08 |
| | LOC_Os02g44360 | GRAS | -0.39 | -6.00 | 9.29E-09 |
| | LOC_Os03g58830 | bHLH | -0.39 | -6.07 | 6.3E-09 |
| | LOC_Os09g36730 | MYB | -0.40 | -6.20 | 3.12E-09 |
| | LOC_Os01g17000 | Dof | -0.40 | -6.51 | 5.02E-10 |
| | LOC_Os04g57610 | ARF | -0.41 | -6.61 | 2.87E-10 |
| | LOC_Os06g04010 | BBR-BPC | -0.42 | -6.87 | 5.96E-11 |
| | LOC_Os10g17630 | B3 | -0.50 | -8.30 | 1.31E-14 |
| CE_M6 | LOC_Os01g64360 | MYB | 0.67 | 12.43 | 3.29E-26 |
| | LOC_Os01g50940 | bHLH | 0.66 | 11.82 | 2.18E-24 |
| | LOC_Os07g36170 | GRAS | 0.64 | 12.42 | 2.85E-27 |
| | LOC_Os03g60560 | C2H2 | 0.62 | 11.75 | 4.18E-25 |
| | LOC_Os02g26430 | WRKY | 0.60 | 10.64 | 3.37E-21 |
| | LOC_Os03g12370 | HSF | 0.59 | 11.02 | 6.31E-23 |
| | LOC_Os06g44010 | WRKY | 0.58 | 10.01 | 2.73E-19 |
| | LOC_Os02g52670 | ERF | 0.57 | 10.36 | 9.37E-21 |
| | LOC_Os03g60080 | NAC | 0.57 | 9.79 | 1.04E-18 |
| | LOC_Os01g63980 | C2H2 | 0.57 | 10.19 | 3.33E-20 |
| | LOC_Os09g32040 | NAC | 0.56 | 9.61 | 3.5E-18 |
| | LOC_Os05g41780 | ERF | 0.55 | 9.84 | 3.25E-19 |
| | LOC_Os01g07120 | ERF | 0.55 | 9.39 | 1.26E-17 |
| | LOC_Os03g02160 | C3H | 0.53 | 9.45 | 4.7E-18 |
| | LOC_Os03g32230 | C2H2 | 0.53 | 9.37 | 8.4E-18 |
| | LOC_Os01g15640 | NAC | 0.53 | 9.08 | 7.76E-17 |
| | LOC_Os07g39470 | GRAS | 0.53 | 9.11 | 6.17E-17 |
| | LOC_Os05g07120 | bHLH | 0.53 | 8.92 | 2.38E-16 |
| | LOC_Os01g58420 | ERF | 0.52 | 9.22 | 2.23E-17 |
| | LOC_Os04g43680 | MYB | 0.52 | 9.27 | 1.49E-17 |
| | LOC_Os02g43790 | ERF | 0.52 | 8.51 | 4.4E-15 |
| | LOC_Os03g55080 | WRKY | 0.51 | 8.91 | 1.83E-16 |
| | LOC_Os07g07974 | CPP | 0.51 | 8.32 | 1.45E-14 |
| | LOC_Os05g49420 | bZIP | 0.50 | 8.13 | 4.68E-14 |
| | LOC_Os01g40260 | WRKY | 0.49 | 7.96 | 1.31E-13 |
| | LOC_Os07g48630 | EIL | 0.49 | 8.47 | 3.34E-15 |
| | LOC_Os08g36920 | ERF | 0.49 | 7.96 | 1.33E-13 |

| | | | | | |
|---|---|---|---|---|---|
| | LOC_Os10g33810 | MYB | 0.49 | 7.66 | 1.01E-12 |
| | LOC_Os02g32590 | HSF | 0.49 | 8.18 | 2.61E-14 |
| | LOC_Os06g43090 | MYB | 0.49 | 7.93 | 1.54E-13 |
| | LOC_Os07g12340 | NAC | 0.49 | 8.37 | 6.21E-15 |
| | LOC_Os02g43330 | HD-ZIP | 0.49 | 7.84 | 2.81E-13 |
| | LOC_Os01g14440 | WRKY | 0.48 | 8.21 | 1.75E-14 |
| | LOC_Os03g55540 | C2H2 | 0.48 | 8.22 | 1.67E-14 |
| | LOC_Os04g51320 | Trihelix | 0.47 | 7.97 | 8.33E-14 |
| | LOC_Os05g37060 | MYB | 0.46 | 7.42 | 3.25E-12 |
| | LOC_Os05g03760 | C3H | 0.46 | 7.28 | 7.38E-12 |
| | LOC_Os09g35030 | ERF | 0.46 | 7.22 | 1.09E-11 |
| | LOC_Os09g28440 | ERF | 0.46 | 7.25 | 9.05E-12 |
| | LOC_Os03g53020 | bHLH | 0.45 | 7.54 | 1.22E-12 |
| | LOC_Os03g64260 | ERF | 0.45 | 7.16 | 1.47E-11 |
| | LOC_Os01g21120 | ERF | 0.45 | 7.51 | 1.38E-12 |
| | LOC_Os07g38090 | C3H | 0.45 | 7.38 | 3.26E-12 |
| | LOC_Os03g21030 | NAC | 0.44 | 6.92 | 5.98E-11 |
| | LOC_Os01g48320 | Trihelix | 0.44 | 7.34 | 3.81E-12 |
| | LOC_Os09g25060 | WRKY | 0.44 | 6.87 | 7.87E-11 |
| | LOC_Os05g37080 | NAC | 0.43 | 6.99 | 3.41E-11 |
| | LOC_Os09g35790 | HSF | 0.43 | 6.66 | 2.56E-10 |
| | LOC_Os08g38210 | bHLH | 0.43 | 7.09 | 1.69E-11 |
| | LOC_Os09g38570 | Trihelix | 0.42 | 6.64 | 2.84E-10 |
| | LOC_Os02g45450 | ERF | 0.42 | 6.38 | 1.3E-09 |
| | LOC_Os11g03300 | NAC | 0.42 | 6.46 | 8.33E-10 |
| | LOC_Os02g13800 | HSF | 0.42 | 6.39 | 1.21E-09 |
| | LOC_Os03g43840 | LSD | 0.41 | 6.53 | 4.95E-10 |
| | LOC_Os01g65900 | GRAS | 0.41 | 6.65 | 2.17E-10 |
| | LOC_Os08g29660 | WRKY | 0.40 | 6.62 | 2.56E-10 |
| | LOC_Os01g72490 | SRS | 0.40 | 6.24 | 2.62E-09 |
| | LOC_Os01g01870 | bHLH | 0.40 | 6.56 | 3.81E-10 |
| | LOC_Os05g37190 | C2H2 | 0.40 | 6.52 | 4.62E-10 |
| | LOC_Os04g45810 | HD-ZIP | 0.39 | 6.30 | 1.59E-09 |
| | LOC_Os01g64730 | bZIP | 0.39 | 6.23 | 2.4E-09 |
| | LOC_Os04g58190 | Dof | 0.39 | 6.27 | 1.82E-09 |
| | LOC_Os10g41200 | MYB_related | 0.39 | 6.32 | 1.35E-09 |
| | LOC_Os04g32620 | ERF | 0.39 | 6.26 | 1.94E-09 |
| | LOC_Os01g61080 | WRKY | 0.39 | 6.20 | 2.8E-09 |
| | LOC_Os06g07030 | ERF | 0.38 | 5.64 | 6.29E-08 |
| | LOC_Os01g54600 | WRKY | 0.38 | 5.86 | 1.86E-08 |
| | LOC_Os03g31230 | MYB_related | 0.37 | 5.83 | 1.99E-08 |
| | LOC_Os10g22950 | CAMTA | 0.37 | 5.99 | 8.06E-09 |
| | LOC_Os01g64310 | NAC | 0.37 | 5.78 | 2.59E-08 |
| | LOC_Os05g27930 | ERF | 0.37 | 5.42 | 1.77E-07 |
| | LOC_Os09g31400 | EIL | 0.37 | 5.48 | 1.3E-07 |
| | LOC_Os03g51330 | GRAS | 0.36 | 5.80 | 2.3E-08 |
| | LOC_Os06g09370 | bHLH | 0.36 | 5.44 | 1.54E-07 |
| | LOC_Os04g55520 | ERF | 0.36 | 5.74 | 3.08E-08 |
| | LOC_Os09g01960 | MYB | 0.35 | 5.34 | 2.55E-07 |
| | LOC_Os06g41770 | bZIP | 0.35 | 5.35 | 2.41E-07 |
| | LOC_Os01g51690 | WRKY | 0.35 | 5.35 | 2.37E-07 |
| | LOC_Os03g12350 | ARR-B | 0.35 | 5.39 | 1.86E-07 |
| | LOC_Os01g09280 | MYB_related | 0.34 | 5.50 | 1.02E-07 |
| | LOC_Os07g38240 | C2H2 | 0.34 | 5.17 | 5.68E-07 |
| | LOC_Os04g46440 | ERF | 0.33 | 5.21 | 4.31E-07 |
| | LOC_Os04g21950 | WRKY | 0.33 | 4.91 | 1.86E-06 |
| | LOC_Os12g40570 | WRKY | 0.33 | 5.20 | 4.51E-07 |
| | LOC_Os01g54550 | HSF | 0.33 | 5.15 | 5.89E-07 |
| | LOC_Os09g28354 | HSF | 0.32 | 4.72 | 4.53E-06 |
| | LOC_Os10g07080 | C2H2 | 0.32 | 4.73 | 4.27E-06 |
| | LOC_Os04g51560 | WRKY | 0.31 | 4.65 | 6.12E-06 |
| | LOC_Os09g28210 | bHLH | 0.31 | 4.75 | 3.71E-06 |
| | LOC_Os01g70270 | ARF | 0.31 | 4.92 | 1.71E-06 |
| | LOC_Os02g05450 | HB-other | 0.31 | 4.62 | 6.92E-06 |
| | LOC_Os03g21710 | WRKY | 0.31 | 4.54 | 9.64E-06 |

| | | | | | |
|---|---|---|---|---|---|
| | LOC_Os04g57600 | C3H | 0.31 | 4.78 | 3.14E-06 |
| | LOC_Os01g10610 | BES1 | 0.30 | 4.79 | 2.97E-06 |
| | LOC_Os02g36360 | C2H2 | 0.30 | 4.66 | 5.41E-06 |
| | LOC_Os03g20780 | EIL | 0.30 | 4.34 | 2.27E-05 |
| | LOC_Os09g12770 | G2-like | 0.29 | 4.38 | 1.91E-05 |
| | LOC_Os04g49110 | GRAS | 0.29 | 4.53 | 9.55E-06 |
| | LOC_Os07g48870 | MYB | 0.29 | 4.48 | 1.18E-05 |
| | LOC_Os12g37690 | MYB | 0.29 | 4.35 | 2.16E-05 |
| | LOC_Os05g41450 | NF-YC | 0.28 | 4.16 | 4.67E-05 |
| | LOC_Os11g06170 | bZIP | 0.28 | 4.17 | 4.62E-05 |
| | LOC_Os01g62660 | G2-like | 0.28 | 4.15 | 5.01E-05 |
| | LOC_Os01g32770 | LBD | 0.28 | 4.37 | 1.93E-05 |
| | LOC_Os05g45410 | HSF | 0.28 | 4.00 | 9.07E-05 |
| | LOC_Os08g15050 | CO-like | 0.27 | 4.00 | 8.82E-05 |
| | LOC_Os11g02480 | WRKY | 0.27 | 3.89 | 0.000137 |
| | LOC_Os09g16510 | WRKY | 0.27 | 3.98 | 9.5E-05 |
| | LOC_Os01g67480 | bHLH | 0.27 | 4.02 | 8.13E-05 |
| | LOC_Os05g41760 | ERF | 0.27 | 4.12 | 5.41E-05 |
| | LOC_Os03g55590 | G2-like | 0.27 | 4.20 | 3.81E-05 |
| | LOC_Os05g09020 | WRKY | 0.27 | 3.77 | 0.00022 |
| | LOC_Os02g44130 | C2H2 | 0.26 | 3.86 | 0.000156 |
| | LOC_Os03g10210 | HD-ZIP | 0.26 | 3.80 | 0.000192 |
| | LOC_Os09g30400 | WRKY | 0.26 | 3.81 | 0.000188 |
| | LOC_Os04g53540 | HD-ZIP | 0.26 | 3.76 | 0.000224 |
| | LOC_Os11g03370 | NAC | 0.26 | 3.99 | 9.14E-05 |
| | LOC_Os08g36790 | bZIP | 0.26 | 3.75 | 0.000231 |
| | LOC_Os07g43530 | bHLH | 0.26 | 3.81 | 0.000181 |
| | LOC_Os01g13460 | bHLH | 0.26 | 3.83 | 0.000168 |
| | LOC_Os08g06140 | NAC | 0.25 | 3.86 | 0.00015 |
| | LOC_Os08g06110 | MYB_related | 0.25 | 3.89 | 0.000132 |
| | LOC_Os02g43170 | DBB | 0.25 | 3.65 | 0.000334 |
| | LOC_Os10g41330 | ERF | 0.25 | 3.64 | 0.000348 |
| | LOC_Os06g14190 | NF-X1 | 0.25 | 3.60 | 0.000403 |
| | LOC_Os01g39020 | HSF | 0.25 | 3.69 | 0.000291 |
| | LOC_Os06g45890 | G2-like | 0.25 | 3.57 | 0.000453 |
| | LOC_Os07g39310 | C2H2 | 0.25 | 3.58 | 0.000438 |
| | LOC_Os06g45840 | MYB_related | 0.24 | 3.60 | 0.000393 |
| | LOC_Os03g13614 | bZIP | 0.24 | 3.53 | 0.000512 |
| | LOC_Os08g31580 | ERF | 0.24 | 3.41 | 0.000795 |
| | LOC_Os02g05510 | GATA | 0.24 | 3.75 | 0.000227 |
| | LOC_Os10g38820 | bZIP | 0.24 | 3.51 | 0.000562 |
| | LOC_Os01g43650 | WRKY | 0.24 | 3.70 | 0.000274 |
| | LOC_Os01g18850 | SBP | 0.24 | 3.64 | 0.000339 |
| | LOC_Os01g06550 | NF-X1 | 0.24 | 3.67 | 0.000301 |
| | LOC_Os02g44120 | C2H2 | 0.24 | 3.43 | 0.000733 |
| | LOC_Os03g58250 | bZIP | 0.23 | 3.51 | 0.000549 |
| | LOC_Os04g58020 | MYB_related | 0.23 | 3.44 | 0.000713 |
| | LOC_Os02g57650 | NAC | 0.23 | 3.34 | 0.00101 |
| | LOC_Os07g48550 | NAC | 0.23 | 3.54 | 0.000488 |
| | LOC_Os05g35500 | MYB | 0.23 | 3.32 | 0.001058 |
| | LOC_Os07g22730 | ERF | 0.23 | 3.24 | 0.001402 |
| | LOC_Os03g56580 | NAC | 0.23 | 3.49 | 0.000578 |
| | LOC_Os01g09100 | WRKY | 0.22 | 3.24 | 0.001412 |
| | LOC_Os03g27390 | bHLH | 0.22 | 3.41 | 0.000771 |
| | LOC_Os08g01090 | B3 | 0.22 | 3.40 | 0.00079 |
| | LOC_Os06g06750 | MIKC_MADS | 0.22 | 3.15 | 0.001872 |
| | LOC_Os07g35870 | bHLH | 0.22 | 3.39 | 0.000825 |
| | LOC_Os01g59660 | MYB | 0.22 | 3.35 | 0.000942 |
| | LOC_Os09g13940 | ERF | 0.22 | 3.16 | 0.001816 |
| | LOC_Os09g35760 | HD-ZIP | 0.22 | 3.05 | 0.002589 |
| | LOC_Os03g53960 | MYB_related | 0.22 | 3.30 | 0.001127 |
| | LOC_Os03g17810 | LBD | 0.22 | 3.09 | 0.002263 |
| | LOC_Os01g06320 | MYB_related | 0.22 | 3.26 | 0.001298 |
| | LOC_Os07g37630 | FAR1 | 0.21 | 3.22 | 0.001498 |
| | LOC_Os09g25070 | WRKY | 0.21 | 3.16 | 0.001821 |

| | | | | |
|---|---|---|---|---|
| LOC_Os02g08540 | bZIP | 0.21 | 2.97 | 0.003338 |
| LOC_Os01g04020 | ERF | 0.21 | 3.12 | 0.002082 |
| LOC_Os10g41770 | STAT | 0.21 | 3.11 | 0.002112 |
| LOC_Os06g35900 | BES1 | 0.21 | 2.93 | 0.003761 |
| LOC_Os04g55970 | AP2 | 0.20 | 3.11 | 0.00213 |
| LOC_Os01g09990 | bHLH | 0.20 | 2.95 | 0.003554 |
| LOC_Os01g47560 | WRKY | 0.20 | 3.08 | 0.002312 |
| LOC_Os12g10540 | MIKC_MADS | 0.20 | 2.97 | 0.003375 |
| LOC_Os01g51610 | B3 | 0.20 | 2.95 | 0.003589 |
| LOC_Os04g40140 | NAC | 0.20 | 2.89 | 0.004331 |
| LOC_Os05g10670 | C3H | 0.20 | 2.83 | 0.005142 |
| LOC_Os03g21240 | G2-like | 0.20 | 3.02 | 0.002788 |
| LOC_Os08g04390 | bHLH | 0.20 | 2.94 | 0.003624 |
| LOC_Os11g29870 | WRKY | 0.20 | 2.83 | 0.005193 |
| LOC_Os01g39330 | bHLH | 0.20 | 2.83 | 0.005159 |
| LOC_Os01g62460 | GRAS | 0.20 | 2.91 | 0.003996 |
| LOC_Os07g25710 | G2-like | 0.20 | 2.96 | 0.003414 |
| LOC_Os05g45020 | C3H | 0.19 | 2.75 | 0.006445 |
| LOC_Os03g58160 | HSF | 0.19 | 2.98 | 0.003197 |
| LOC_Os01g10504 | MIKC_MADS | 0.19 | 2.69 | 0.007703 |
| LOC_Os05g49620 | WRKY | 0.19 | 2.77 | 0.006221 |
| LOC_Os01g64560 | bHLH | 0.19 | 2.90 | 0.004073 |
| LOC_Os02g10840 | FAR1 | 0.19 | 2.93 | 0.00372 |
| LOC_Os08g37920 | C2H2 | 0.19 | 2.83 | 0.005093 |
| LOC_Os02g07170 | G2-like | 0.19 | 2.66 | 0.008515 |
| LOC_Os01g70810 | HB-other | 0.19 | 2.79 | 0.005731 |
| LOC_Os04g58000 | B3 | 0.19 | 2.83 | 0.00507 |
| LOC_Os03g54160 | MIKC_MADS | 0.19 | 2.64 | 0.009065 |
| LOC_Os04g49150 | MIKC_MADS | 0.18 | 2.74 | 0.006678 |
| LOC_Os02g08150 | CO-like | 0.18 | 2.61 | 0.009873 |
| LOC_Os01g14870 | C3H | 0.18 | 2.59 | 0.010382 |
| LOC_Os03g06860 | FAR1 | 0.18 | 2.71 | 0.007264 |
| LOC_Os09g24820 | ZF-HD | 0.18 | 2.49 | 0.013584 |
| LOC_Os05g35170 | NAC | 0.18 | 2.57 | 0.010872 |
| LOC_Os02g51670 | ERF | 0.18 | 2.72 | 0.007007 |
| LOC_Os03g05690 | C2H2 | 0.18 | 2.64 | 0.008946 |
| LOC_Os06g12400 | HB-PHD | 0.17 | 2.52 | 0.012435 |
| LOC_Os12g13170 | bZIP | 0.17 | 2.64 | 0.008954 |
| LOC_Os02g40530 | MYB | 0.17 | 2.62 | 0.009279 |
| LOC_Os02g45480 | C3H | 0.17 | 2.46 | 0.014967 |
| LOC_Os03g15660 | ERF | 0.17 | 2.58 | 0.010464 |
| LOC_Os01g10370 | ERF | 0.17 | 2.56 | 0.011106 |
| LOC_Os01g15900 | Dof | 0.17 | 2.53 | 0.012171 |
| LOC_Os01g13540 | Nin-like | 0.17 | 2.59 | 0.010159 |
| LOC_Os07g48450 | NAC | 0.17 | 2.54 | 0.011657 |
| LOC_Os10g41460 | Trihelix | 0.17 | 2.50 | 0.01311 |
| LOC_Os08g28214 | CPP | 0.17 | 2.39 | 0.017764 |
| LOC_Os03g09100 | CAMTA | 0.17 | 2.57 | 0.010935 |
| LOC_Os03g08460 | ERF | 0.17 | 2.56 | 0.011224 |
| LOC_Os07g01820 | MIKC_MADS | 0.17 | 2.51 | 0.012669 |
| LOC_Os01g62410 | MYB | 0.17 | 2.52 | 0.012486 |
| LOC_Os03g06630 | HSF | 0.17 | 2.46 | 0.014486 |
| LOC_Os11g05614 | NAC | 0.16 | 2.32 | 0.021359 |
| LOC_Os10g37240 | Trihelix | 0.16 | 2.45 | 0.015171 |
| LOC_Os10g27360 | NAC | 0.16 | 2.41 | 0.016844 |
| LOC_Os01g68370 | B3 | 0.16 | 2.42 | 0.016263 |
| LOC_Os12g41210 | CPP | 0.16 | 2.42 | 0.01625 |
| LOC_Os01g18870 | bHLH | 0.16 | 2.38 | 0.018005 |
| LOC_Os12g06640 | Trihelix | 0.16 | 2.41 | 0.016581 |
| LOC_Os06g07010 | FAR1 | 0.16 | 2.33 | 0.020504 |
| LOC_Os01g69830 | SBP | 0.16 | 2.18 | 0.03025 |
| LOC_Os08g04170 | C3H | 0.15 | 2.30 | 0.022158 |
| LOC_Os10g42490 | HD-ZIP | 0.15 | 2.29 | 0.022764 |
| LOC_Os01g68160 | C2H2 | 0.15 | 2.18 | 0.030041 |
| LOC_Os03g42290 | B3 | 0.15 | 2.27 | 0.024466 |

| | | | | |
|---|---|---|---|---|
| LOC_Os08g38020 | bZIP | 0.15 | 2.21 | 0.028223 |
| LOC_Os07g42510 | ERF | 0.15 | 2.13 | 0.034697 |
| LOC_Os03g08960 | HD-ZIP | 0.15 | 2.22 | 0.027438 |
| LOC_Os01g55340 | Dof | 0.15 | 2.08 | 0.038954 |
| LOC_Os01g43550 | WRKY | 0.14 | 2.18 | 0.030581 |
| LOC_Os03g54170 | MIKC_MADS | 0.14 | 2.14 | 0.033829 |
| LOC_Os06g11970 | M-type_MADS | 0.14 | 2.03 | 0.043796 |
| LOC_Os04g41560 | DBB | 0.14 | 1.98 | 0.048961 |
| LOC_Os03g15010 | FAR1 | 0.14 | 2.15 | 0.032298 |
| LOC_Os06g45140 | bZIP | 0.14 | 1.99 | 0.048438 |
| LOC_Os02g34630 | MYB_related | 0.14 | 2.08 | 0.038547 |
| LOC_Os03g37920 | FAR1 | 0.14 | 2.10 | 0.036902 |
| LOC_Os02g06910 | ARF | 0.14 | 2.13 | 0.034126 |
| LOC_Os12g38490 | GRAS | 0.14 | 2.00 | 0.046825 |
| LOC_Os03g55990 | HB-other | 0.14 | 2.09 | 0.037978 |
| LOC_Os06g16400 | bHLH | 0.13 | 2.01 | 0.045761 |
| LOC_Os01g15350 | C3H | 0.13 | 2.04 | 0.042561 |
| LOC_Os12g41230 | CPP | -0.13 | -1.97 | 0.049594 |
| LOC_Os08g41940 | SBP | -0.13 | -2.02 | 0.044841 |
| LOC_Os07g48660 | bZIP | -0.14 | -2.02 | 0.044551 |
| LOC_Os08g42470 | bHLH | -0.14 | -2.11 | 0.036012 |
| LOC_Os04g43910 | ARF | -0.14 | -2.03 | 0.044108 |
| LOC_Os02g13310 | TALE | -0.14 | -1.99 | 0.047667 |
| LOC_Os11g06840 | C2H2 | -0.14 | -2.11 | 0.035993 |
| LOC_Os06g04090 | NAC | -0.14 | -2.03 | 0.0432 |
| LOC_Os05g50270 | GATA | -0.14 | -2.05 | 0.042041 |
| LOC_Os12g38400 | MYB | -0.15 | -2.13 | 0.034432 |
| LOC_Os01g68700 | bHLH | -0.15 | -2.19 | 0.029892 |
| LOC_Os10g39030 | TALE | -0.15 | -2.16 | 0.031811 |
| LOC_Os04g23910 | MIKC_MADS | -0.15 | -2.07 | 0.039359 |
| LOC_Os01g48130 | NAC | -0.15 | -2.20 | 0.028577 |
| LOC_Os07g03770 | TALE | -0.16 | -2.17 | 0.03086 |
| LOC_Os12g43950 | TALE | -0.16 | -2.33 | 0.020831 |
| LOC_Os09g26420 | ERF | -0.16 | -2.27 | 0.024243 |
| LOC_Os04g46580 | SBP | -0.16 | -2.21 | 0.028393 |
| LOC_Os01g57650 | C2H2 | -0.16 | -2.24 | 0.02627 |
| LOC_Os01g63160 | MYB | -0.16 | -2.35 | 0.019487 |
| LOC_Os11g11100 | bZIP | -0.16 | -2.25 | 0.025799 |
| LOC_Os03g15440 | bHLH | -0.16 | -2.42 | 0.016422 |
| LOC_Os09g13570 | bZIP | -0.16 | -2.28 | 0.023409 |
| LOC_Os12g41860 | HD-ZIP | -0.16 | -2.43 | 0.016035 |
| LOC_Os03g46860 | bHLH | -0.16 | -2.32 | 0.021562 |
| LOC_Os10g40390 | GRAS | -0.16 | -2.25 | 0.025609 |
| LOC_Os12g42970 | GATA | -0.16 | -2.44 | 0.015705 |
| LOC_Os09g12380 | NAC | -0.17 | -2.31 | 0.022256 |
| LOC_Os07g44640 | C2H2 | -0.17 | -2.50 | 0.013345 |
| LOC_Os01g08160 | G2-like | -0.17 | -2.52 | 0.01247 |
| LOC_Os09g12750 | G2-like | -0.17 | -2.40 | 0.017325 |
| LOC_Os05g01256 | bHLH | -0.17 | -2.40 | 0.017327 |
| LOC_Os06g50900 | bHLH | -0.17 | -2.37 | 0.018626 |
| LOC_Os03g42420 | B3 | -0.17 | -2.44 | 0.015507 |
| LOC_Os03g04310 | bHLH | -0.17 | -2.56 | 0.011083 |
| LOC_Os08g37400 | ZF-HD | -0.17 | -2.60 | 0.010072 |
| LOC_Os01g74590 | MYB | -0.17 | -2.49 | 0.013666 |
| LOC_Os05g41240 | G2-like | -0.18 | -2.52 | 0.012603 |
| LOC_Os08g37580 | HD-ZIP | -0.18 | -2.61 | 0.009805 |
| LOC_Os05g11070 | bHLH | -0.18 | -2.50 | 0.013236 |
| LOC_Os02g40070 | AP2 | -0.18 | -2.74 | 0.00669 |
| LOC_Os03g13600 | C2H2 | -0.18 | -2.70 | 0.007458 |
| LOC_Os01g74140 | WRKY | -0.18 | -2.68 | 0.00791 |
| LOC_Os05g38120 | TALE | -0.18 | -2.61 | 0.009686 |
| LOC_Os12g40590 | bHLH | -0.19 | -2.72 | 0.007164 |
| LOC_Os02g44370 | GRAS | -0.19 | -2.73 | 0.006909 |
| LOC_Os01g03720 | MYB | -0.19 | -2.87 | 0.004487 |
| LOC_Os09g38340 | C2H2 | -0.19 | -2.75 | 0.006534 |

| | | | | | |
|---|---|---|---|---|---|
| | LOC_Os01g70870 | C2H2 | -0.20 | -2.84 | 0.005 |
| | LOC_Os05g03884 | TALE | -0.20 | -2.93 | 0.003775 |
| | LOC_Os01g68560 | M-type_MADS | -0.20 | -3.08 | 0.002359 |
| | LOC_Os03g07880 | NF-YA | -0.20 | -3.06 | 0.00253 |
| | LOC_Os07g47330 | ERF | -0.21 | -2.91 | 0.004082 |
| | LOC_Os06g49040 | G2-like | -0.21 | -3.03 | 0.002751 |
| | LOC_Os06g35140 | G2-like | -0.21 | -3.08 | 0.002393 |
| | LOC_Os06g09420 | B3 | -0.22 | -3.15 | 0.001882 |
| | LOC_Os09g10840 | bZIP | -0.22 | -3.10 | 0.00223 |
| | LOC_Os12g06200 | E2F/DP | -0.22 | -3.38 | 0.000864 |
| | LOC_Os02g39140 | bHLH | -0.22 | -3.18 | 0.001715 |
| | LOC_Os07g22770 | ERF | -0.22 | -3.41 | 0.000768 |
| | LOC_Os08g02300 | NAC | -0.22 | -3.40 | 0.000809 |
| | LOC_Os04g08060 | C2H2 | -0.22 | -3.23 | 0.001435 |
| | LOC_Os03g05500 | LBD | -0.23 | -3.24 | 0.00142 |
| | LOC_Os10g17630 | B3 | -0.24 | -3.58 | 0.000422 |
| | LOC_Os05g02420 | MYB_related | -0.24 | -3.60 | 0.000401 |
| | LOC_Os01g50720 | MYB | -0.25 | -3.54 | 0.000494 |
| | LOC_Os12g12380 | FAR1 | -0.25 | -3.77 | 0.00021 |
| | LOC_Os05g42130 | GRAS | -0.25 | -3.57 | 0.000441 |
| | LOC_Os01g55750 | TCP | -0.25 | -3.71 | 0.000267 |
| | LOC_Os08g44830 | C2H2 | -0.25 | -3.64 | 0.000346 |
| | LOC_Os09g29820 | bZIP | -0.25 | -3.61 | 0.000393 |
| | LOC_Os10g42850 | WRKY | -0.25 | -3.70 | 0.000278 |
| | LOC_Os04g50920 | WRKY | -0.25 | -3.74 | 0.000243 |
| | LOC_Os02g50630 | E2F/DP | -0.25 | -3.64 | 0.000344 |
| | LOC_Os01g09850 | C2H2 | -0.26 | -3.69 | 0.000296 |
| | LOC_Os03g55760 | G2-like | -0.26 | -3.93 | 0.000113 |
| | LOC_Os04g35010 | bHLH | -0.26 | -3.85 | 0.000155 |
| | LOC_Os06g08440 | ARR-B | -0.27 | -3.79 | 0.000199 |
| | LOC_Os12g37970 | MYB | -0.27 | -4.16 | 4.61E-05 |
| | LOC_Os05g04820 | MYB | -0.27 | -4.07 | 6.76E-05 |
| | LOC_Os01g18240 | MYB | -0.28 | -4.00 | 9.07E-05 |
| | LOC_Os02g46560 | bHLH | -0.28 | -4.26 | 3.04E-05 |
| | LOC_Os11g25610 | C2H2 | -0.28 | -3.97 | 0.000104 |
| | LOC_Os05g50080 | C3H | -0.28 | -4.08 | 6.64E-05 |
| | LOC_Os11g05740 | B3 | -0.28 | -4.39 | 1.79E-05 |
| | LOC_Os07g39800 | HRT-like | -0.29 | -4.53 | 9.56E-06 |
| | LOC_Os02g52190 | bHLH | -0.29 | -4.10 | 6.07E-05 |
| | LOC_Os02g12790 | GATA | -0.29 | -4.25 | 3.24E-05 |
| | LOC_Os06g09717 | ERF | -0.29 | -4.20 | 4.06E-05 |
| | LOC_Os12g42400 | NF-YA | -0.29 | -4.54 | 9.19E-06 |
| | LOC_Os04g54474 | bZIP | -0.29 | -4.30 | 2.69E-05 |
| | LOC_Os05g48850 | NAC | -0.29 | -4.24 | 3.53E-05 |
| | LOC_Os01g63460 | MYB | -0.30 | -4.57 | 8.32E-06 |
| | LOC_Os01g17000 | Dof | -0.30 | -4.73 | 3.97E-06 |
| | LOC_Os01g36460 | MYB | -0.32 | -4.67 | 5.78E-06 |
| | LOC_Os08g33750 | G2-like | -0.32 | -5.01 | 1.1E-06 |
| | LOC_Os04g44440 | TCP | -0.34 | -5.22 | 4.35E-07 |
| | LOC_Os04g50770 | MYB | -0.34 | -5.05 | 9.82E-07 |
| | LOC_Os09g33490 | NAC | -0.34 | -5.04 | 1.04E-06 |
| | LOC_Os08g33660 | MYB | -0.34 | -5.48 | 1.11E-07 |
| | LOC_Os05g20930 | C2H2 | -0.35 | -5.09 | 8.6E-07 |
| | LOC_Os05g02390 | C2H2 | -0.35 | -5.21 | 4.77E-07 |
| | LOC_Os09g36730 | MYB | -0.35 | -5.26 | 3.7E-07 |
| | LOC_Os01g53260 | WRKY | -0.35 | -5.23 | 4.27E-07 |
| | LOC_Os03g42630 | NAC | -0.36 | -5.36 | 2.3E-07 |
| | LOC_Os02g46780 | MYB | -0.39 | -6.14 | 4.01E-09 |
| | LOC_Os05g51160 | MYB_related | -0.41 | -6.39 | 1.14E-09 |
| Os_1K_004 | LOC_Os04g28090 | MYB | 0.63 | 12.25 | 9.87E-27 |
| | LOC_Os03g12120 | NAC | 0.59 | 10.77 | 5.06E-22 |
| | LOC_Os06g41384 | C3H | 0.58 | 9.73 | 2.57E-18 |
| | LOC_Os04g40060 | FAR1 | 0.54 | 9.56 | 2.45E-18 |
| | LOC_Os04g35800 | C3H | 0.54 | 8.99 | 1.94E-16 |
| | LOC_Os02g06370 | Whirly | 0.54 | 8.92 | 3.11E-16 |

| | | | | |
|---|---|---|---|---|
| LOC_Os02g33560 | bZIP | 0.51 | 8.88 | 2.45E-16 |
| LOC_Os06g12400 | HB-PHD | 0.51 | 8.50 | 4.3E-15 |
| LOC_Os03g05480 | C2H2 | 0.51 | 8.81 | 3.96E-16 |
| LOC_Os08g19590 | HD-ZIP | 0.51 | 8.26 | 2.24E-14 |
| LOC_Os03g05690 | C2H2 | 0.50 | 8.48 | 3.57E-15 |
| LOC_Os02g05450 | HB-other | 0.50 | 8.16 | 3.52E-14 |
| LOC_Os07g44640 | C2H2 | 0.50 | 8.42 | 5.39E-15 |
| LOC_Os01g48700 | E2F/DP | 0.49 | 8.07 | 5.73E-14 |
| LOC_Os02g18660 | GeBP | 0.49 | 7.80 | 3.58E-13 |
| LOC_Os03g50900 | FAR1 | 0.48 | 8.06 | 5.51E-14 |
| LOC_Os04g40930 | Trihelix | 0.48 | 7.97 | 1.04E-13 |
| LOC_Os04g02730 | C3H | 0.48 | 7.78 | 3.92E-13 |
| LOC_Os01g04020 | ERF | 0.48 | 8.01 | 6.69E-14 |
| LOC_Os12g42970 | GATA | 0.47 | 7.82 | 2.46E-13 |
| LOC_Os02g18370 | FAR1 | 0.46 | 7.83 | 2E-13 |
| LOC_Os06g04010 | BBR-BPC | 0.46 | 7.75 | 3.04E-13 |
| LOC_Os06g46890 | C3H | 0.45 | 7.16 | 1.54E-11 |
| LOC_Os11g35030 | GRF | 0.45 | 7.04 | 3.24E-11 |
| LOC_Os02g10840 | FAR1 | 0.45 | 7.47 | 1.71E-12 |
| LOC_Os06g45840 | MYB_related | 0.44 | 7.03 | 3.09E-11 |
| LOC_Os03g06860 | FAR1 | 0.44 | 7.23 | 7.94E-12 |
| LOC_Os01g72490 | SRS | 0.43 | 6.78 | 1.3E-10 |
| LOC_Os03g27390 | bHLH | 0.43 | 7.13 | 1.44E-11 |
| LOC_Os12g18150 | C2H2 | 0.43 | 7.15 | 1.22E-11 |
| LOC_Os06g02560 | GRF | 0.43 | 7.04 | 2.42E-11 |
| LOC_Os01g67970 | C2H2 | 0.43 | 6.71 | 1.91E-10 |
| LOC_Os02g42870 | MYB | 0.42 | 6.64 | 2.95E-10 |
| LOC_Os12g41920 | MYB_related | 0.42 | 6.84 | 7.73E-11 |
| LOC_Os05g25320 | FAR1 | 0.41 | 6.39 | 1.17E-09 |
| LOC_Os05g09630 | HB-other | 0.41 | 6.79 | 9.76E-11 |
| LOC_Os01g06550 | NF-X1 | 0.41 | 6.66 | 2.14E-10 |
| LOC_Os12g41230 | CPP | 0.41 | 6.60 | 3.04E-10 |
| LOC_Os02g39540 | FAR1 | 0.40 | 6.56 | 3.62E-10 |
| LOC_Os02g34630 | MYB_related | 0.40 | 6.31 | 1.57E-09 |
| LOC_Os01g42710 | LSD | 0.40 | 6.47 | 6.09E-10 |
| LOC_Os03g13614 | bZIP | 0.39 | 6.05 | 7.08E-09 |
| LOC_Os03g63750 | HSF | 0.39 | 6.29 | 1.66E-09 |
| LOC_Os04g11830 | TCP | 0.39 | 5.90 | 1.59E-08 |
| LOC_Os04g45650 | GATA | 0.39 | 6.20 | 2.73E-09 |
| LOC_Os01g63160 | MYB | 0.38 | 6.10 | 4.88E-09 |
| LOC_Os04g53540 | HD-ZIP | 0.38 | 5.78 | 2.82E-08 |
| LOC_Os08g37904 | C2H2 | 0.38 | 5.67 | 5.25E-08 |
| LOC_Os03g50110 | GeBP | 0.38 | 6.10 | 4.8E-09 |
| LOC_Os03g60120 | ERF | 0.38 | 6.10 | 4.54E-09 |
| LOC_Os01g70810 | HB-other | 0.38 | 5.91 | 1.34E-08 |
| LOC_Os03g51970 | GRF | 0.38 | 5.89 | 1.48E-08 |
| LOC_Os08g04170 | C3H | 0.38 | 6.10 | 4.63E-09 |
| LOC_Os12g41860 | HD-ZIP | 0.37 | 5.93 | 1.14E-08 |
| LOC_Os03g53960 | MYB_related | 0.37 | 5.91 | 1.27E-08 |
| LOC_Os07g13170 | AP2 | 0.37 | 5.56 | 8.85E-08 |
| LOC_Os01g32890 | GeBP | 0.36 | 5.50 | 1.15E-07 |
| LOC_Os01g64560 | bHLH | 0.36 | 5.75 | 2.94E-08 |
| LOC_Os03g01890 | HD-ZIP | 0.36 | 5.82 | 1.94E-08 |
| LOC_Os09g29460 | HD-ZIP | 0.36 | 5.41 | 1.8E-07 |
| LOC_Os08g28214 | CPP | 0.36 | 5.32 | 2.9E-07 |
| LOC_Os10g42490 | HD-ZIP | 0.36 | 5.73 | 3.18E-08 |
| LOC_Os06g16400 | bHLH | 0.36 | 5.65 | 5E-08 |
| LOC_Os03g08370 | FAR1 | 0.35 | 5.60 | 6.38E-08 |
| LOC_Os03g42280 | B3 | 0.35 | 5.34 | 2.55E-07 |
| LOC_Os03g15010 | FAR1 | 0.35 | 5.58 | 7.11E-08 |
| LOC_Os05g41450 | NF-YC | 0.35 | 5.22 | 4.47E-07 |
| LOC_Os04g57600 | C3H | 0.35 | 5.54 | 8.48E-08 |
| LOC_Os02g19804 | C3H | 0.35 | 5.15 | 6.25E-07 |
| LOC_Os05g51830 | C2H2 | 0.35 | 5.44 | 1.42E-07 |
| LOC_Os01g09760 | MYB_related | 0.35 | 5.51 | 9.94E-08 |

| | | | | | |
|---|---|---|---|---|---|
| | LOC_Os08g42440 | CO-like | 0.35 | 5.53 | 8.83E-08 |
| | LOC_Os08g01090 | B3 | 0.34 | 5.45 | 1.34E-07 |
| | LOC_Os06g46410 | ARF | 0.34 | 4.97 | 1.47E-06 |
| | LOC_Os08g23470 | B3 | 0.34 | 5.07 | 9.15E-07 |
| | LOC_Os06g41770 | bZIP | 0.34 | 5.09 | 8.12E-07 |
| | LOC_Os06g07010 | FAR1 | 0.33 | 5.14 | 6.04E-07 |
| | LOC_Os03g62660 | FAR1 | 0.33 | 5.11 | 7.12E-07 |
| | LOC_Os07g37630 | FAR1 | 0.33 | 5.10 | 7.46E-07 |
| | LOC_Os06g49080 | C3H | 0.32 | 4.84 | 2.6E-06 |
| | LOC_Os03g55220 | bHLH | 0.32 | 4.99 | 1.21E-06 |
| | LOC_Os02g53150 | GeBP | 0.32 | 4.83 | 2.68E-06 |
| | LOC_Os04g59430 | ARF | 0.32 | 4.99 | 1.25E-06 |
| | LOC_Os01g42970 | C3H | 0.32 | 4.67 | 5.69E-06 |
| | LOC_Os10g22950 | CAMTA | 0.31 | 5.02 | 1.03E-06 |
| | LOC_Os02g34590 | FAR1 | 0.31 | 4.93 | 1.62E-06 |
| | LOC_Os12g41950 | ARF | 0.31 | 4.81 | 2.85E-06 |
| | LOC_Os07g44200 | GeBP | 0.31 | 4.90 | 1.83E-06 |
| | LOC_Os11g09160 | B3 | 0.31 | 4.60 | 7.43E-06 |
| | LOC_Os03g58830 | bHLH | 0.30 | 4.52 | 1.06E-05 |
| | LOC_Os02g33750 | FAR1 | 0.30 | 4.50 | 1.17E-05 |
| | LOC_Os02g33610 | Trihelix | 0.30 | 4.31 | 2.62E-05 |
| | LOC_Os04g52560 | FAR1 | 0.30 | 4.67 | 5.14E-06 |
| | LOC_Os11g06410 | Trihelix | 0.30 | 4.35 | 2.19E-05 |
| | LOC_Os03g63530 | NF-YC | 0.30 | 4.62 | 6.61E-06 |
| | LOC_Os05g03020 | C2H2 | 0.29 | 4.30 | 2.68E-05 |
| | LOC_Os10g17630 | B3 | 0.29 | 4.42 | 1.6E-05 |
| | LOC_Os03g13790 | MYB_related | 0.29 | 4.55 | 8.77E-06 |
| | LOC_Os08g17400 | WRKY | 0.29 | 4.51 | 1.06E-05 |
| | LOC_Os07g08140 | HSF | 0.29 | 4.53 | 9.61E-06 |
| | LOC_Os09g24820 | ZF-HD | 0.29 | 4.15 | 5.08E-05 |
| | LOC_Os07g30774 | CAMTA | 0.29 | 4.35 | 2.13E-05 |
| | LOC_Os12g06380 | FAR1 | 0.29 | 4.47 | 1.26E-05 |
| | LOC_Os02g06910 | ARF | 0.29 | 4.54 | 9.04E-06 |
| | LOC_Os04g56850 | ARF | 0.29 | 4.42 | 1.55E-05 |
| | LOC_Os07g39480 | WRKY | 0.28 | 4.38 | 1.86E-05 |
| | LOC_Os01g48060 | ARF | 0.28 | 4.37 | 1.94E-05 |
| | LOC_Os07g07974 | CPP | 0.28 | 4.12 | 5.54E-05 |
| | LOC_Os05g38460 | MYB | 0.28 | 4.14 | 5.22E-05 |
| | LOC_Os03g55990 | HB-other | 0.28 | 4.37 | 1.94E-05 |
| | LOC_Os07g35870 | bHLH | 0.28 | 4.35 | 2.07E-05 |
| | LOC_Os09g13940 | ERF | 0.28 | 4.07 | 6.67E-05 |
| | LOC_Os02g05510 | GATA | 0.28 | 4.33 | 2.21E-05 |
| | LOC_Os04g50060 | GRAS | 0.28 | 4.34 | 2.14E-05 |
| | LOC_Os07g43420 | MYB | 0.27 | 4.27 | 2.82E-05 |
| | LOC_Os01g15350 | C3H | 0.27 | 4.23 | 3.38E-05 |
| | LOC_Os01g09620 | C3H | 0.27 | 3.92 | 0.00012 |
| | LOC_Os07g43030 | CAMTA | 0.27 | 4.03 | 7.74E-05 |
| | LOC_Os02g58670 | bZIP | 0.26 | 4.13 | 5.21E-05 |
| | LOC_Os07g25710 | G2-like | 0.26 | 4.01 | 8.36E-05 |
| | LOC_Os09g12750 | G2-like | 0.26 | 3.80 | 0.000192 |
| | LOC_Os03g11614 | MIKC_MADS | 0.26 | 3.64 | 0.000355 |
| | LOC_Os01g17000 | Dof | 0.26 | 3.97 | 9.59E-05 |
| | LOC_Os03g21800 | bZIP | 0.26 | 3.89 | 0.000131 |
| | LOC_Os12g29520 | ARF | 0.25 | 3.92 | 0.000116 |
| | LOC_Os12g41210 | CPP | 0.25 | 3.88 | 0.000139 |
| | LOC_Os06g50900 | bHLH | 0.25 | 3.55 | 0.000484 |
| | LOC_Os06g09420 | B3 | 0.25 | 3.67 | 0.000311 |
| | LOC_Os03g06850 | B3 | 0.25 | 3.59 | 0.00042 |
| | LOC_Os12g40570 | WRKY | 0.25 | 3.81 | 0.000182 |
| | LOC_Os09g35760 | HD-ZIP | 0.25 | 3.46 | 0.000673 |
| | LOC_Os05g50080 | C3H | 0.24 | 3.54 | 0.000496 |
| | LOC_Os02g45850 | B3 | 0.24 | 3.52 | 0.000536 |
| | LOC_Os01g68160 | C2H2 | 0.24 | 3.58 | 0.00043 |
| | LOC_Os01g61810 | NF-YB | 0.24 | 3.41 | 0.000805 |
| | LOC_Os08g25799 | G2-like | 0.24 | 3.57 | 0.000449 |

| | | | | | |
|---|---|---|---|---|---|
| | LOC_Os12g06200 | E2F/DP | 0.24 | 3.76 | 0.000218 |
| | LOC_Os05g50270 | GATA | 0.24 | 3.50 | 0.000579 |
| | LOC_Os06g40960 | C2H2 | 0.24 | 3.49 | 0.000594 |
| | LOC_Os01g54550 | HSF | 0.24 | 3.68 | 0.000298 |
| | LOC_Os07g28430 | GRF | 0.24 | 3.47 | 0.00064 |
| | LOC_Os06g04870 | HD-ZIP | 0.24 | 3.64 | 0.000336 |
| | LOC_Os03g58250 | bZIP | 0.24 | 3.60 | 0.000391 |
| | LOC_Os02g53670 | MYB_related | 0.24 | 3.63 | 0.000351 |
| | LOC_Os09g01140 | GeBP | 0.23 | 3.35 | 0.000964 |
| | LOC_Os01g15460 | C3H | 0.23 | 3.54 | 0.000486 |
| | LOC_Os06g14190 | NF-X1 | 0.23 | 3.37 | 0.000918 |
| | LOC_Os12g42400 | NF-YA | 0.23 | 3.50 | 0.000564 |
| | LOC_Os05g41070 | bZIP | 0.23 | 3.29 | 0.001171 |
| | LOC_Os04g57610 | ARF | 0.23 | 3.47 | 0.000626 |
| | LOC_Os03g54170 | MIKC_MADS | 0.23 | 3.46 | 0.000656 |
| | LOC_Os11g03110 | GRAS | 0.22 | 3.19 | 0.001676 |
| | LOC_Os01g62660 | G2-like | 0.22 | 3.22 | 0.001498 |
| | LOC_Os01g12440 | ERF | 0.22 | 3.18 | 0.001692 |
| | LOC_Os08g29500 | NF-YB | 0.22 | 3.18 | 0.001722 |
| | LOC_Os03g53340 | HSF | 0.22 | 3.41 | 0.000779 |
| | LOC_Os12g07120 | GATA | 0.22 | 3.34 | 0.000983 |
| | LOC_Os01g59350 | bZIP | 0.22 | 3.33 | 0.001015 |
| | LOC_Os08g36790 | bZIP | 0.22 | 3.11 | 0.00218 |
| | LOC_Os10g41130 | ERF | 0.22 | 3.32 | 0.001043 |
| | LOC_Os01g13300 | B3 | 0.21 | 3.20 | 0.001563 |
| | LOC_Os08g33590 | bHLH | 0.21 | 3.22 | 0.001459 |
| | LOC_Os02g45480 | C3H | 0.21 | 3.00 | 0.003084 |
| | LOC_Os04g45330 | YABBY | 0.21 | 2.96 | 0.00342 |
| | LOC_Os12g37410 | bZIP | 0.21 | 3.03 | 0.002748 |
| | LOC_Os02g38470 | B3 | 0.21 | 2.91 | 0.003997 |
| | LOC_Os01g14720 | GeBP | 0.21 | 3.16 | 0.001808 |
| | LOC_Os11g32100 | bHLH | 0.21 | 2.99 | 0.003186 |
| | LOC_Os06g49010 | SBP | 0.21 | 2.89 | 0.004281 |
| | LOC_Os04g46860 | GRAS | 0.20 | 3.10 | 0.002158 |
| | LOC_Os06g40710 | G2-like | 0.20 | 2.94 | 0.003717 |
| | LOC_Os06g02230 | B3 | 0.20 | 3.06 | 0.002502 |
| | LOC_Os08g40900 | ARF | 0.20 | 2.87 | 0.004615 |
| | LOC_Os02g43150 | GATA | 0.20 | 2.85 | 0.00477 |
| | LOC_Os10g41260 | MYB_related | 0.20 | 2.89 | 0.00421 |
| | LOC_Os01g70270 | ARF | 0.20 | 3.01 | 0.002876 |
| | LOC_Os02g39520 | FAR1 | 0.20 | 2.91 | 0.00398 |
| | LOC_Os08g09900 | WRKY | 0.20 | 2.94 | 0.003617 |
| | LOC_Os03g63810 | WRKY | 0.20 | 2.79 | 0.005789 |
| | LOC_Os02g47280 | GRF | 0.20 | 2.79 | 0.005791 |
| | LOC_Os08g37920 | C2H2 | 0.20 | 2.92 | 0.003853 |
| | LOC_Os08g37580 | HD-ZIP | 0.19 | 2.87 | 0.004529 |
| | LOC_Os12g12380 | FAR1 | 0.19 | 2.93 | 0.003771 |
| | LOC_Os02g42380 | TCP | 0.19 | 2.94 | 0.003637 |
| | LOC_Os03g02160 | C3H | 0.19 | 2.95 | 0.003497 |
| | LOC_Os06g19444 | CO-like | 0.19 | 2.78 | 0.005957 |
| | LOC_Os11g11100 | bZIP | 0.19 | 2.69 | 0.007678 |
| | LOC_Os05g27930 | ERF | 0.19 | 2.63 | 0.00923 |
| | LOC_Os01g68370 | B3 | 0.19 | 2.82 | 0.00521 |
| | LOC_Os09g39660 | C2H2 | 0.19 | 2.64 | 0.008859 |
| | LOC_Os06g33940 | NAC | 0.18 | 2.63 | 0.009207 |
| | LOC_Os03g21240 | G2-like | 0.18 | 2.78 | 0.005955 |
| | LOC_Os07g44690 | HSF | 0.18 | 2.53 | 0.012206 |
| | LOC_Os08g39830 | EIL | 0.18 | 2.57 | 0.010911 |
| | LOC_Os12g13170 | bZIP | 0.18 | 2.69 | 0.007773 |
| | LOC_Os06g04850 | HD-ZIP | 0.18 | 2.54 | 0.011748 |
| | LOC_Os01g21120 | ERF | 0.18 | 2.69 | 0.00773 |
| | LOC_Os03g43930 | HD-ZIP | 0.18 | 2.52 | 0.012481 |
| | LOC_Os12g38940 | C2H2 | 0.18 | 2.64 | 0.008805 |
| | LOC_Os10g41770 | STAT | 0.17 | 2.63 | 0.009177 |
| | LOC_Os01g48320 | Trihelix | 0.17 | 2.61 | 0.009718 |

| | | | | | |
|---|---|---|---|---|---|
| | LOC_Os11g47900 | GRAS | 0.17 | 2.41 | 0.017109 |
| | LOC_Os11g30484 | C2H2 | 0.17 | 2.43 | 0.015979 |
| | LOC_Os06g11860 | ERF | 0.17 | 2.40 | 0.0174 |
| | LOC_Os12g21700 | C3H | 0.17 | 2.46 | 0.014545 |
| | LOC_Os05g45020 | C3H | 0.17 | 2.34 | 0.020141 |
| | LOC_Os07g04700 | MYB | 0.16 | 2.50 | 0.013214 |
| | LOC_Os12g06640 | Trihelix | 0.16 | 2.46 | 0.014512 |
| | LOC_Os02g33430 | E2F/DP | 0.16 | 2.39 | 0.017563 |
| | LOC_Os07g38090 | C3H | 0.16 | 2.37 | 0.01865 |
| | LOC_Os05g43380 | CPP | 0.16 | 2.41 | 0.01691 |
| | LOC_Os04g55970 | AP2 | 0.16 | 2.36 | 0.019047 |
| | LOC_Os05g10670 | C3H | 0.16 | 2.22 | 0.027795 |
| | LOC_Os02g07780 | SBP | 0.16 | 2.21 | 0.027995 |
| | LOC_Os01g69850 | M-type_MADS | 0.16 | 2.16 | 0.031927 |
| | LOC_Os03g55590 | G2-like | 0.15 | 2.36 | 0.019155 |
| | LOC_Os06g49550 | FAR1 | 0.15 | 2.12 | 0.035153 |
| | LOC_Os12g10660 | DBB | 0.15 | 2.32 | 0.021478 |
| | LOC_Os04g36590 | FAR1 | 0.15 | 2.15 | 0.032594 |
| | LOC_Os01g10610 | BES1 | 0.15 | 2.30 | 0.022191 |
| | LOC_Os05g48820 | HB-other | 0.15 | 2.11 | 0.036207 |
| | LOC_Os04g47890 | G2-like | 0.15 | 2.25 | 0.025119 |
| | LOC_Os05g43950 | VOZ | 0.15 | 2.08 | 0.039069 |
| | LOC_Os11g06010 | bHLH | 0.15 | 2.08 | 0.038662 |
| | LOC_Os02g49440 | Dof | 0.15 | 2.24 | 0.026295 |
| | LOC_Os06g08340 | ERF | 0.15 | 2.04 | 0.043021 |
| | LOC_Os02g12790 | GATA | 0.15 | 2.10 | 0.03689 |
| | LOC_Os09g30400 | WRKY | 0.15 | 2.09 | 0.037505 |
| | LOC_Os02g34680 | C2H2 | 0.15 | 2.20 | 0.028872 |
| | LOC_Os02g47060 | WRKY | 0.15 | 2.20 | 0.028981 |
| | LOC_Os04g32620 | ERF | 0.15 | 2.20 | 0.028693 |
| | LOC_Os03g09280 | GRAS | 0.14 | 2.15 | 0.03259 |
| | LOC_Os05g25260 | ERF | 0.14 | 2.02 | 0.044524 |
| | LOC_Os08g41950 | MIKC_MADS | 0.14 | 2.01 | 0.045714 |
| | LOC_Os02g47660 | bHLH | 0.14 | 2.04 | 0.042461 |
| | LOC_Os04g55560 | AP2 | 0.13 | 1.99 | 0.048215 |
| | LOC_Os10g21560 | NAC | -0.13 | -2.00 | 0.047005 |
| | LOC_Os07g39800 | HRT-like | -0.13 | -2.03 | 0.043409 |
| | LOC_Os03g31240 | C2H2 | -0.14 | -1.99 | 0.047881 |
| | LOC_Os03g06930 | TALE | -0.14 | -2.04 | 0.042038 |
| | LOC_Os03g48970 | NF-YA | -0.14 | -2.08 | 0.038963 |
| | LOC_Os05g34050 | bZIP | -0.14 | -2.00 | 0.046874 |
| | LOC_Os07g48570 | Dof | -0.14 | -2.11 | 0.035774 |
| | LOC_Os01g03720 | MYB | -0.14 | -2.11 | 0.036035 |
| | LOC_Os04g46220 | ERF | -0.14 | -1.98 | 0.049601 |
| | LOC_Os01g08160 | G2-like | -0.14 | -2.12 | 0.035038 |
| | LOC_Os08g44830 | C2H2 | -0.14 | -2.04 | 0.042983 |
| | LOC_Os02g49480 | bHLH | -0.14 | -2.09 | 0.037419 |
| | LOC_Os01g70110 | NAC | -0.15 | -2.01 | 0.046055 |
| | LOC_Os05g02390 | C2H2 | -0.15 | -2.08 | 0.038829 |
| | LOC_Os02g34970 | NAC | -0.15 | -2.27 | 0.024372 |
| | LOC_Os02g44120 | C2H2 | -0.15 | -2.11 | 0.036585 |
| | LOC_Os10g25170 | ERF | -0.15 | -2.26 | 0.024597 |
| | LOC_Os04g47860 | C2H2 | -0.15 | -2.26 | 0.024689 |
| | LOC_Os05g20930 | C2H2 | -0.15 | -2.11 | 0.035932 |
| | LOC_Os11g06170 | bZIP | -0.15 | -2.16 | 0.032203 |
| | LOC_Os04g51320 | Trihelix | -0.15 | -2.29 | 0.023238 |
| | LOC_Os03g37920 | FAR1 | -0.15 | -2.29 | 0.022777 |
| | LOC_Os01g15640 | NAC | -0.15 | -2.25 | 0.025651 |
| | LOC_Os11g03420 | ZF-HD | -0.15 | -2.17 | 0.031299 |
| | LOC_Os03g05500 | LBD | -0.16 | -2.20 | 0.029212 |
| | LOC_Os03g46860 | bHLH | -0.16 | -2.24 | 0.026115 |
| | LOC_Os05g10690 | MYB_related | -0.16 | -2.27 | 0.024263 |
| | LOC_Os02g08540 | bZIP | -0.16 | -2.24 | 0.025946 |
| | LOC_Os05g37060 | MYB | -0.16 | -2.31 | 0.021662 |
| | LOC_Os07g36170 | GRAS | -0.16 | -2.47 | 0.014391 |

| | | | | | |
|---|---|---|---|---|---|
| | LOC_Os08g41030 | ERF | -0.16 | -2.43 | 0.016005 |
| | LOC_Os03g43840 | LSD | -0.17 | -2.43 | 0.015876 |
| | LOC_Os06g35140 | G2-like | -0.17 | -2.37 | 0.018991 |
| | LOC_Os09g28440 | ERF | -0.17 | -2.39 | 0.017568 |
| | LOC_Os03g07880 | NF-YA | -0.17 | -2.50 | 0.01326 |
| | LOC_Os03g12350 | ARR-B | -0.17 | -2.49 | 0.013498 |
| | LOC_Os03g08960 | HD-ZIP | -0.17 | -2.56 | 0.011065 |
| | LOC_Os06g49040 | G2-like | -0.17 | -2.41 | 0.017018 |
| | LOC_Os06g11970 | M-type_MADS | -0.17 | -2.44 | 0.01557 |
| | LOC_Os07g48260 | WRKY | -0.17 | -2.54 | 0.011903 |
| | LOC_Os07g39470 | GRAS | -0.17 | -2.55 | 0.011459 |
| | LOC_Os01g09850 | C2H2 | -0.17 | -2.41 | 0.017049 |
| | LOC_Os01g54600 | WRKY | -0.17 | -2.50 | 0.013331 |
| | LOC_Os10g42130 | NAC | -0.17 | -2.41 | 0.016907 |
| | LOC_Os05g04820 | MYB | -0.17 | -2.52 | 0.012418 |
| | LOC_Os05g37190 | C2H2 | -0.17 | -2.63 | 0.009245 |
| | LOC_Os02g52190 | bHLH | -0.17 | -2.41 | 0.016949 |
| | LOC_Os01g07120 | ERF | -0.17 | -2.53 | 0.012313 |
| | LOC_Os04g50920 | WRKY | -0.17 | -2.52 | 0.012394 |
| | LOC_Os11g03300 | NAC | -0.18 | -2.49 | 0.013548 |
| | LOC_Os01g09590 | MYB | -0.18 | -2.51 | 0.013008 |
| | LOC_Os04g54474 | bZIP | -0.18 | -2.54 | 0.011875 |
| | LOC_Os05g49620 | WRKY | -0.18 | -2.55 | 0.011445 |
| | LOC_Os03g07360 | Dof | -0.18 | -2.70 | 0.007498 |
| | LOC_Os02g46780 | MYB | -0.18 | -2.68 | 0.007847 |
| | LOC_Os01g36460 | MYB | -0.18 | -2.53 | 0.012212 |
| | LOC_Os02g49700 | HD-ZIP | -0.18 | -2.53 | 0.012104 |
| | LOC_Os03g08470 | ERF | -0.18 | -2.74 | 0.006608 |
| | LOC_Os11g25610 | C2H2 | -0.18 | -2.52 | 0.012669 |
| | LOC_Os11g05480 | bZIP | -0.18 | -2.56 | 0.011113 |
| | LOC_Os12g40590 | bHLH | -0.18 | -2.69 | 0.007811 |
| | LOC_Os08g39630 | bHLH | -0.18 | -2.69 | 0.007839 |
| | LOC_Os01g58420 | ERF | -0.19 | -2.83 | 0.005099 |
| | LOC_Os09g25060 | WRKY | -0.19 | -2.67 | 0.008144 |
| | LOC_Os01g50110 | MYB | -0.19 | -2.76 | 0.006368 |
| | LOC_Os10g33810 | MYB | -0.19 | -2.60 | 0.009979 |
| | LOC_Os01g65080 | C2H2 | -0.19 | -2.89 | 0.004269 |
| | LOC_Os01g63460 | MYB | -0.19 | -2.88 | 0.004397 |
| | LOC_Os02g02820 | bHLH | -0.19 | -2.93 | 0.00376 |
| | LOC_Os03g21710 | WRKY | -0.19 | -2.78 | 0.005939 |
| | LOC_Os06g45890 | G2-like | -0.20 | -2.80 | 0.005604 |
| | LOC_Os04g45020 | MYB | -0.20 | -2.99 | 0.003144 |
| | LOC_Os09g26420 | ERF | -0.20 | -2.92 | 0.003917 |
| | LOC_Os09g32510 | bHLH | -0.20 | -2.80 | 0.005684 |
| | LOC_Os05g42130 | GRAS | -0.20 | -2.87 | 0.00459 |
| | LOC_Os01g09990 | bHLH | -0.20 | -2.91 | 0.004006 |
| | LOC_Os01g69830 | SBP | -0.20 | -2.89 | 0.004307 |
| | LOC_Os09g11480 | ERF | -0.20 | -2.96 | 0.003484 |
| | LOC_Os01g43650 | WRKY | -0.21 | -3.13 | 0.001973 |
| | LOC_Os03g22170 | ERF | -0.21 | -3.13 | 0.001989 |
| | LOC_Os01g74140 | WRKY | -0.21 | -3.08 | 0.002346 |
| | LOC_Os01g64360 | MYB | -0.21 | -2.91 | 0.004023 |
| | LOC_Os01g61080 | WRKY | -0.21 | -3.16 | 0.001784 |
| | LOC_Os01g14440 | WRKY | -0.21 | -3.20 | 0.001599 |
| | LOC_Os03g47740 | TALE | -0.21 | -3.20 | 0.001568 |
| | LOC_Os07g48450 | NAC | -0.21 | -3.16 | 0.001801 |
| | LOC_Os07g22770 | ERF | -0.21 | -3.22 | 0.001484 |
| | LOC_Os06g24070 | G2-like | -0.21 | -3.06 | 0.002517 |
| | LOC_Os02g52670 | ERF | -0.21 | -3.26 | 0.001311 |
| | LOC_Os03g17810 | LBD | -0.22 | -3.09 | 0.002282 |
| | LOC_Os02g45450 | ERF | -0.22 | -3.07 | 0.002441 |
| | LOC_Os10g26620 | Dof | -0.22 | -3.38 | 0.00085 |
| | LOC_Os02g08500 | ARR-B | -0.22 | -3.06 | 0.002499 |
| | LOC_Os03g29614 | MYB | -0.22 | -3.06 | 0.002506 |
| | LOC_Os06g44010 | WRKY | -0.23 | -3.23 | 0.001463 |

| | | | | | |
|---|---|---|---|---|---|
| | LOC_Os07g48820 | bZIP | -0.23 | -3.29 | 0.001184 |
| | LOC_Os05g41760 | ERF | -0.23 | -3.42 | 0.000756 |
| | LOC_Os12g01490 | G2-like | -0.23 | -3.28 | 0.001206 |
| | LOC_Os08g19650 | TALE | -0.23 | -3.32 | 0.001077 |
| | LOC_Os01g13740 | G2-like | -0.23 | -3.54 | 0.000483 |
| | LOC_Os05g38120 | TALE | -0.23 | -3.32 | 0.001073 |
| | LOC_Os08g04390 | bHLH | -0.23 | -3.51 | 0.000541 |
| | LOC_Os01g43590 | HSF | -0.23 | -3.45 | 0.000684 |
| | LOC_Os01g09100 | WRKY | -0.24 | -3.43 | 0.000738 |
| | LOC_Os03g56580 | NAC | -0.24 | -3.66 | 0.000313 |
| | LOC_Os02g43790 | ERF | -0.24 | -3.47 | 0.000641 |
| | LOC_Os08g10080 | NAC | -0.24 | -3.50 | 0.00057 |
| | LOC_Os04g41560 | DBB | -0.24 | -3.43 | 0.000739 |
| | LOC_Os03g44710 | YABBY | -0.25 | -3.81 | 0.000176 |
| | LOC_Os01g09550 | NAC | -0.25 | -3.79 | 0.000194 |
| | LOC_Os09g31390 | bZIP | -0.25 | -3.54 | 0.000492 |
| | LOC_Os01g60640 | WRKY | -0.25 | -3.60 | 0.000408 |
| | LOC_Os02g13800 | HSF | -0.25 | -3.56 | 0.000475 |
| | LOC_Os02g26430 | WRKY | -0.25 | -3.60 | 0.000396 |
| | LOC_Os11g29870 | WRKY | -0.25 | -3.70 | 0.000281 |
| | LOC_Os09g33490 | NAC | -0.26 | -3.71 | 0.000271 |
| | LOC_Os02g40530 | MYB | -0.26 | -3.96 | 0.0001 |
| | LOC_Os01g52540 | B3 | -0.26 | -3.78 | 0.000205 |
| | LOC_Os01g40260 | WRKY | -0.26 | -3.81 | 0.000189 |
| | LOC_Os01g64310 | NAC | -0.26 | -4.02 | 8.01E-05 |
| | LOC_Os02g43970 | ERF | -0.26 | -3.71 | 0.000272 |
| | LOC_Os02g49880 | CO-like | -0.27 | -4.07 | 6.49E-05 |
| | LOC_Os08g26880 | bZIP | -0.27 | -4.10 | 5.76E-05 |
| | LOC_Os09g38340 | C2H2 | -0.27 | -3.89 | 0.000134 |
| | LOC_Os09g35910 | HD-ZIP | -0.27 | -3.82 | 0.00018 |
| | LOC_Os07g22730 | ERF | -0.27 | -3.82 | 0.000178 |
| | LOC_Os04g45810 | HD-ZIP | -0.27 | -4.13 | 5.07E-05 |
| | LOC_Os01g53040 | WRKY | -0.27 | -4.21 | 3.79E-05 |
| | LOC_Os01g51690 | WRKY | -0.27 | -4.07 | 6.72E-05 |
| | LOC_Os02g43300 | Trihelix | -0.28 | -4.04 | 7.59E-05 |
| | LOC_Os07g48550 | NAC | -0.28 | -4.30 | 2.53E-05 |
| | LOC_Os04g50770 | MYB | -0.28 | -4.06 | 7.09E-05 |
| | LOC_Os03g64260 | ERF | -0.28 | -4.09 | 6.35E-05 |
| | LOC_Os02g39140 | bHLH | -0.28 | -4.07 | 6.83E-05 |
| | LOC_Os04g53990 | bHLH | -0.28 | -4.40 | 1.69E-05 |
| | LOC_Os09g28210 | bHLH | -0.28 | -4.23 | 3.46E-05 |
| | LOC_Os07g48630 | EIL | -0.28 | -4.40 | 1.67E-05 |
| | LOC_Os03g42630 | NAC | -0.28 | -4.16 | 4.78E-05 |
| | LOC_Os03g08490 | ERF | -0.28 | -4.15 | 4.87E-05 |
| | LOC_Os05g48850 | NAC | -0.29 | -4.17 | 4.62E-05 |
| | LOC_Os03g20900 | G2-like | -0.29 | -4.58 | 7.75E-06 |
| | LOC_Os01g43550 | WRKY | -0.29 | -4.60 | 7.14E-06 |
| | LOC_Os09g35030 | ERF | -0.30 | -4.35 | 2.21E-05 |
| | LOC_Os03g32230 | C2H2 | -0.30 | -4.66 | 5.5E-06 |
| | LOC_Os07g37920 | NAC | -0.30 | -4.55 | 8.88E-06 |
| | LOC_Os06g12230 | TCP | -0.30 | -4.41 | 1.69E-05 |
| | LOC_Os08g02300 | NAC | -0.30 | -4.70 | 4.7E-06 |
| | LOC_Os08g29660 | WRKY | -0.30 | -4.76 | 3.4E-06 |
| | LOC_Os05g33810 | SBP | -0.31 | -4.68 | 5.07E-06 |
| | LOC_Os07g48596 | G2-like | -0.31 | -4.38 | 2.03E-05 |
| | LOC_Os01g39330 | bHLH | -0.31 | -4.57 | 8.39E-06 |
| | LOC_Os05g48010 | MYB | -0.32 | -4.85 | 2.53E-06 |
| | LOC_Os07g42510 | ERF | -0.33 | -4.91 | 1.9E-06 |
| | LOC_Os01g48130 | NAC | -0.33 | -5.05 | 9.7E-07 |
| | LOC_Os01g09640 | MYB_related | -0.33 | -4.96 | 1.53E-06 |
| | LOC_Os04g56990 | G2-like | -0.34 | -5.20 | 4.73E-07 |
| | LOC_Os10g39030 | TALE | -0.34 | -5.31 | 2.69E-07 |
| | LOC_Os12g37970 | MYB | -0.34 | -5.47 | 1.17E-07 |
| | LOC_Os01g53260 | WRKY | -0.34 | -5.12 | 7.16E-07 |
| | LOC_Os02g36880 | NAC | -0.34 | -5.09 | 8.35E-07 |

| | | | | | |
|---|---|---|---|---|---|
| | LOC_Os01g51140 | bHLH | -0.35 | -5.10 | 8.34E-07 |
| | LOC_Os05g45410 | HSF | -0.35 | -5.08 | 8.86E-07 |
| | LOC_Os11g05614 | NAC | -0.35 | -5.20 | 5.03E-07 |
| | LOC_Os09g28354 | HSF | -0.35 | -5.19 | 5.38E-07 |
| | LOC_Os08g36920 | ERF | -0.35 | -5.25 | 3.97E-07 |
| | LOC_Os05g02420 | MYB_related | -0.35 | -5.38 | 2E-07 |
| | LOC_Os03g21030 | NAC | -0.35 | -5.31 | 2.93E-07 |
| | LOC_Os10g41230 | HD-ZIP | -0.35 | -5.12 | 7.61E-07 |
| | LOC_Os04g38720 | NAC | -0.36 | -5.40 | 1.88E-07 |
| | LOC_Os02g56600 | NAC | -0.36 | -5.74 | 3.02E-08 |
| | LOC_Os06g04090 | NAC | -0.36 | -5.41 | 1.81E-07 |
| | LOC_Os05g51160 | MYB_related | -0.36 | -5.56 | 8.48E-08 |
| | LOC_Os08g02070 | MIKC_MADS | -0.37 | -5.86 | 1.68E-08 |
| | LOC_Os09g29820 | bZIP | -0.37 | -5.56 | 8.73E-08 |
| | LOC_Os05g03760 | C3H | -0.39 | -5.93 | 1.29E-08 |
| | LOC_Os12g41680 | NAC | -0.39 | -6.23 | 2.42E-09 |
| | LOC_Os04g43680 | MYB | -0.39 | -6.45 | 6.52E-10 |
| | LOC_Os12g43950 | TALE | -0.40 | -6.36 | 1.18E-09 |
| | LOC_Os05g38140 | bHLH | -0.41 | -6.25 | 2.42E-09 |
| | LOC_Os03g55540 | C2H2 | -0.41 | -6.68 | 1.88E-10 |
| | LOC_Os01g67480 | bHLH | -0.41 | -6.41 | 1.01E-09 |
| | LOC_Os05g37080 | NAC | -0.41 | -6.67 | 2.1E-10 |
| | LOC_Os09g10840 | bZIP | -0.41 | -6.32 | 1.77E-09 |
| | LOC_Os01g50720 | MYB | -0.42 | -6.41 | 1.08E-09 |
| | LOC_Os04g43560 | NAC | -0.42 | -6.37 | 1.33E-09 |
| | LOC_Os02g47190 | G2-like | -0.42 | -6.62 | 3.14E-10 |
| | LOC_Os06g46270 | NAC | -0.43 | -6.68 | 2.25E-10 |
| | LOC_Os08g15050 | CO-like | -0.44 | -6.82 | 1.07E-10 |
| | LOC_Os09g16510 | WRKY | -0.45 | -7.05 | 2.8E-11 |
| | LOC_Os03g53020 | bHLH | -0.45 | -7.50 | 1.56E-12 |
| | LOC_Os03g17150 | C2H2 | -0.46 | -7.49 | 1.97E-12 |
| | LOC_Os10g01470 | HD-ZIP | -0.49 | -7.76 | 4.77E-13 |
| W-box | LOC_Os04g43680 | MYB | 0.58 | 10.62 | 1.18E-21 |
| | LOC_Os03g32230 | C2H2 | 0.51 | 8.95 | 1.42E-16 |
| | LOC_Os05g03760 | C3H | 0.51 | 8.29 | 1.63E-14 |
| | LOC_Os01g14440 | WRKY | 0.51 | 8.74 | 5.68E-16 |
| | LOC_Os03g55540 | C2H2 | 0.49 | 8.34 | 7.69E-15 |
| | LOC_Os02g43790 | ERF | 0.48 | 7.69 | 6.81E-13 |
| | LOC_Os05g37080 | NAC | 0.47 | 7.83 | 2.1E-13 |
| | LOC_Os10g33810 | MYB | 0.47 | 7.22 | 1.3E-11 |
| | LOC_Os08g29660 | WRKY | 0.46 | 7.66 | 5.39E-13 |
| | LOC_Os09g28354 | HSF | 0.45 | 7.10 | 2.23E-11 |
| | LOC_Os06g44010 | WRKY | 0.44 | 6.87 | 8.53E-11 |
| | LOC_Os03g53020 | bHLH | 0.44 | 7.29 | 5.58E-12 |
| | LOC_Os02g26430 | WRKY | 0.44 | 6.90 | 6.77E-11 |
| | LOC_Os01g09990 | bHLH | 0.42 | 6.64 | 2.9E-10 |
| | LOC_Os01g07120 | ERF | 0.42 | 6.65 | 2.62E-10 |
| | LOC_Os07g42510 | ERF | 0.42 | 6.58 | 3.94E-10 |
| | LOC_Os01g39330 | bHLH | 0.42 | 6.47 | 7.58E-10 |
| | LOC_Os09g16510 | WRKY | 0.42 | 6.44 | 8.8E-10 |
| | LOC_Os05g45410 | HSF | 0.41 | 6.21 | 3.39E-09 |
| | LOC_Os01g40260 | WRKY | 0.40 | 6.17 | 3.88E-09 |
| | LOC_Os09g32040 | NAC | 0.40 | 6.07 | 6.58E-09 |
| | LOC_Os11g05614 | NAC | 0.39 | 5.90 | 1.55E-08 |
| | LOC_Os07g48630 | EIL | 0.39 | 6.25 | 2.04E-09 |
| | LOC_Os03g21030 | NAC | 0.39 | 5.89 | 1.66E-08 |
| | LOC_Os07g48450 | NAC | 0.39 | 6.15 | 3.65E-09 |
| | LOC_Os01g51690 | WRKY | 0.38 | 5.92 | 1.3E-08 |
| | LOC_Os09g35030 | ERF | 0.38 | 5.76 | 3.27E-08 |
| | LOC_Os01g15640 | NAC | 0.38 | 5.94 | 1.19E-08 |
| | LOC_Os01g65900 | GRAS | 0.38 | 6.08 | 5.24E-09 |
| | LOC_Os03g64260 | ERF | 0.38 | 5.74 | 3.43E-08 |
| | LOC_Os01g43650 | WRKY | 0.37 | 5.99 | 8.18E-09 |
| | LOC_Os07g36170 | GRAS | 0.37 | 5.94 | 1.09E-08 |
| | LOC_Os10g41330 | ERF | 0.37 | 5.61 | 6.74E-08 |

| | | | | | |
|---|---|---|---|---|---|
| | LOC_Os06g45890 | G2-like | 0.37 | 5.57 | 8.16E-08 |
| | LOC_Os07g48820 | bZIP | 0.37 | 5.61 | 6.57E-08 |
| | LOC_Os03g12370 | HSF | 0.37 | 5.97 | 9E-09 |
| | LOC_Os08g36920 | ERF | 0.36 | 5.48 | 1.26E-07 |
| | LOC_Os05g48010 | MYB | 0.36 | 5.40 | 1.86E-07 |
| | LOC_Os11g02480 | WRKY | 0.35 | 5.19 | 5.31E-07 |
| | LOC_Os05g37190 | C2H2 | 0.35 | 5.58 | 6.81E-08 |
| | LOC_Os01g61080 | WRKY | 0.35 | 5.50 | 1.07E-07 |
| | LOC_Os03g02160 | C3H | 0.35 | 5.53 | 8.89E-08 |
| | LOC_Os07g12340 | NAC | 0.35 | 5.52 | 9.2E-08 |
| | LOC_Os01g64360 | MYB | 0.34 | 4.90 | 2.11E-06 |
| | LOC_Os04g51560 | WRKY | 0.34 | 5.01 | 1.19E-06 |
| | LOC_Os03g56580 | NAC | 0.33 | 5.28 | 3.12E-07 |
| | LOC_Os07g48870 | MYB | 0.33 | 5.27 | 3.21E-07 |
| | LOC_Os05g49620 | WRKY | 0.33 | 5.00 | 1.23E-06 |
| | LOC_Os08g15050 | CO-like | 0.33 | 4.97 | 1.47E-06 |
| | LOC_Os01g67480 | bHLH | 0.33 | 5.02 | 1.13E-06 |
| | LOC_Os01g53040 | WRKY | 0.33 | 5.22 | 4.09E-07 |
| | LOC_Os07g39470 | GRAS | 0.33 | 5.11 | 7.06E-07 |
| | LOC_Os09g25060 | WRKY | 0.33 | 4.93 | 1.73E-06 |
| | LOC_Os02g36360 | C2H2 | 0.33 | 5.18 | 4.97E-07 |
| | LOC_Os04g43560 | NAC | 0.33 | 4.80 | 3.25E-06 |
| | LOC_Os06g46270 | NAC | 0.33 | 4.90 | 1.96E-06 |
| | LOC_Os01g18850 | SBP | 0.32 | 5.00 | 1.16E-06 |
| | LOC_Os02g36880 | NAC | 0.32 | 4.71 | 4.76E-06 |
| | LOC_Os05g38140 | bHLH | 0.32 | 4.73 | 4.25E-06 |
| | LOC_Os01g64310 | NAC | 0.32 | 4.87 | 2.15E-06 |
| | LOC_Os03g21710 | WRKY | 0.31 | 4.65 | 6.06E-06 |
| | LOC_Os07g48550 | NAC | 0.31 | 4.91 | 1.75E-06 |
| | LOC_Os09g38570 | Trihelix | 0.31 | 4.61 | 7.17E-06 |
| | LOC_Os11g03300 | NAC | 0.31 | 4.51 | 1.11E-05 |
| | LOC_Os11g29870 | WRKY | 0.30 | 4.49 | 1.19E-05 |
| | LOC_Os05g35170 | NAC | 0.30 | 4.50 | 1.16E-05 |
| | LOC_Os02g52670 | ERF | 0.30 | 4.69 | 4.88E-06 |
| | LOC_Os03g43840 | LSD | 0.30 | 4.55 | 9.28E-06 |
| | LOC_Os07g38240 | C2H2 | 0.29 | 4.36 | 2.03E-05 |
| | LOC_Os01g70110 | NAC | 0.29 | 4.15 | 5.04E-05 |
| | LOC_Os04g58190 | Dof | 0.28 | 4.34 | 2.13E-05 |
| | LOC_Os09g25070 | WRKY | 0.28 | 4.33 | 2.24E-05 |
| | LOC_Os04g51320 | Trihelix | 0.28 | 4.28 | 2.83E-05 |
| | LOC_Os09g35790 | HSF | 0.27 | 4.03 | 8.05E-05 |
| | LOC_Os01g54600 | WRKY | 0.27 | 4.05 | 7.31E-05 |
| | LOC_Os04g32620 | ERF | 0.27 | 4.19 | 4.06E-05 |
| | LOC_Os03g60080 | NAC | 0.27 | 3.93 | 0.000116 |
| | LOC_Os11g03370 | NAC | 0.27 | 4.15 | 4.66E-05 |
| | LOC_Os09g35910 | HD-ZIP | 0.27 | 3.83 | 0.000175 |
| | LOC_Os03g22170 | ERF | 0.26 | 4.06 | 6.85E-05 |
| | LOC_Os08g38210 | bHLH | 0.26 | 4.07 | 6.56E-05 |
| | LOC_Os03g12350 | ARR-B | 0.26 | 3.92 | 0.00012 |
| | LOC_Os04g45810 | HD-ZIP | 0.26 | 3.93 | 0.000114 |
| | LOC_Os07g22730 | ERF | 0.26 | 3.64 | 0.000352 |
| | LOC_Os03g08460 | ERF | 0.25 | 3.88 | 0.000136 |
| | LOC_Os01g09100 | WRKY | 0.25 | 3.62 | 0.000372 |
| | LOC_Os06g09370 | bHLH | 0.25 | 3.59 | 0.000419 |
| | LOC_Os03g60560 | C2H2 | 0.24 | 3.71 | 0.000266 |
| | LOC_Os03g55080 | WRKY | 0.24 | 3.69 | 0.000281 |
| | LOC_Os05g07120 | bHLH | 0.24 | 3.54 | 0.000486 |
| | LOC_Os03g01870 | NAC | 0.24 | 3.35 | 0.000975 |
| | LOC_Os03g17810 | LBD | 0.24 | 3.39 | 0.000833 |
| | LOC_Os05g49420 | bZIP | 0.23 | 3.38 | 0.000866 |
| | LOC_Os01g63980 | C2H2 | 0.23 | 3.54 | 0.000481 |
| | LOC_Os06g45140 | bZIP | 0.23 | 3.27 | 0.001276 |
| | LOC_Os12g41680 | NAC | 0.23 | 3.41 | 0.000764 |
| | LOC_Os01g06640 | bHLH | 0.23 | 3.45 | 0.000663 |
| | LOC_Os02g45450 | ERF | 0.22 | 3.14 | 0.001932 |

| | | | | |
|---|---|---|---|---|
| LOC_Os02g13800 | HSF | 0.22 | 3.17 | 0.001799 |
| LOC_Os01g50940 | bHLH | 0.22 | 3.08 | 0.002368 |
| LOC_Os08g34360 | ERF | 0.22 | 3.18 | 0.00172 |
| LOC_Os03g20900 | G2-like | 0.22 | 3.37 | 0.00087 |
| LOC_Os01g58420 | ERF | 0.21 | 3.27 | 0.001229 |
| LOC_Os09g31390 | bZIP | 0.21 | 3.02 | 0.002837 |
| LOC_Os01g11910 | bHLH | 0.21 | 2.98 | 0.003209 |
| LOC_Os10g42130 | NAC | 0.21 | 2.93 | 0.003837 |
| LOC_Os10g38820 | bZIP | 0.21 | 2.99 | 0.003101 |
| LOC_Os11g05480 | bZIP | 0.21 | 2.90 | 0.004173 |
| LOC_Os01g09280 | MYB_related | 0.21 | 3.15 | 0.001883 |
| LOC_Os05g33810 | SBP | 0.20 | 3.03 | 0.002769 |
| LOC_Os01g01870 | bHLH | 0.20 | 3.09 | 0.002278 |
| LOC_Os03g08470 | ERF | 0.20 | 3.06 | 0.002451 |
| LOC_Os01g52540 | B3 | 0.20 | 2.87 | 0.004568 |
| LOC_Os02g57650 | NAC | 0.20 | 2.88 | 0.004463 |
| LOC_Os02g43170 | DBB | 0.20 | 2.84 | 0.004956 |
| LOC_Os09g10840 | bZIP | 0.20 | 2.83 | 0.005185 |
| LOC_Os03g10210 | HD-ZIP | 0.20 | 2.84 | 0.004936 |
| LOC_Os09g11480 | ERF | 0.20 | 2.86 | 0.004719 |
| LOC_Os04g50920 | WRKY | 0.20 | 2.85 | 0.004768 |
| LOC_Os05g41780 | ERF | 0.20 | 2.99 | 0.003139 |
| LOC_Os03g08490 | ERF | 0.20 | 2.80 | 0.005634 |
| LOC_Os03g20780 | EIL | 0.20 | 2.79 | 0.005732 |
| LOC_Os09g30400 | WRKY | 0.19 | 2.81 | 0.005492 |
| LOC_Os02g39140 | bHLH | 0.19 | 2.77 | 0.006071 |
| LOC_Os08g02070 | MIKC_MADS | 0.19 | 2.94 | 0.003595 |
| LOC_Os05g37060 | MYB | 0.19 | 2.80 | 0.005544 |
| LOC_Os02g40530 | MYB | 0.19 | 2.87 | 0.0045 |
| LOC_Os01g60640 | WRKY | 0.19 | 2.72 | 0.007031 |
| LOC_Os10g26620 | Dof | 0.19 | 2.90 | 0.004061 |
| LOC_Os01g69830 | SBP | 0.19 | 2.64 | 0.00885 |
| LOC_Os09g01960 | MYB | 0.19 | 2.68 | 0.007987 |
| LOC_Os02g57490 | LBD | 0.19 | 2.82 | 0.00521 |
| LOC_Os03g17150 | C2H2 | 0.19 | 2.71 | 0.007356 |
| LOC_Os04g49110 | GRAS | 0.18 | 2.79 | 0.005745 |
| LOC_Os01g51140 | bHLH | 0.18 | 2.54 | 0.011899 |
| LOC_Os09g28440 | ERF | 0.18 | 2.58 | 0.010714 |
| LOC_Os02g34970 | NAC | 0.18 | 2.72 | 0.007096 |
| LOC_Os02g08540 | bZIP | 0.18 | 2.53 | 0.012038 |
| LOC_Os07g43530 | bHLH | 0.18 | 2.58 | 0.010425 |
| LOC_Os01g09640 | MYB_related | 0.18 | 2.51 | 0.012756 |
| LOC_Os03g42430 | B3 | 0.17 | 2.63 | 0.009228 |
| LOC_Os03g20550 | WRKY | 0.17 | 2.64 | 0.008807 |
| LOC_Os01g43550 | WRKY | 0.17 | 2.59 | 0.010335 |
| LOC_Os05g09020 | WRKY | 0.17 | 2.37 | 0.018999 |
| LOC_Os04g40140 | NAC | 0.17 | 2.44 | 0.015596 |
| LOC_Os04g45690 | DBB | 0.17 | 2.56 | 0.010991 |
| LOC_Os01g53260 | WRKY | 0.17 | 2.38 | 0.018069 |
| LOC_Os01g21120 | ERF | 0.17 | 2.52 | 0.012328 |
| LOC_Os12g39330 | ERF | 0.17 | 2.28 | 0.023473 |
| LOC_Os05g35500 | MYB | 0.16 | 2.31 | 0.022172 |
| LOC_Os06g35140 | G2-like | 0.16 | 2.28 | 0.023679 |
| LOC_Os06g12230 | TCP | 0.16 | 2.25 | 0.025691 |
| LOC_Os02g43330 | HD-ZIP | 0.16 | 2.24 | 0.026228 |
| LOC_Os03g51330 | GRAS | 0.16 | 2.38 | 0.018073 |
| LOC_Os09g29820 | bZIP | 0.16 | 2.21 | 0.028174 |
| LOC_Os04g55520 | ERF | 0.15 | 2.33 | 0.020876 |
| LOC_Os03g31230 | MYB_related | 0.15 | 2.27 | 0.024101 |
| LOC_Os02g08500 | ARR-B | 0.15 | 2.12 | 0.035443 |
| LOC_Os03g19370 | bZIP | 0.15 | 2.30 | 0.022606 |
| LOC_Os01g48320 | Trihelix | 0.15 | 2.28 | 0.02348 |
| LOC_Os12g40570 | WRKY | 0.15 | 2.27 | 0.024066 |
| LOC_Os01g65080 | C2H2 | 0.15 | 2.26 | 0.024592 |
| LOC_Os07g22770 | ERF | 0.15 | 2.24 | 0.025769 |

| | | | | | |
|---|---|---|---|---|---|
| | LOC_Os03g09100 | CAMTA | 0.15 | 2.25 | 0.02551 |
| | LOC_Os02g47190 | G2-like | 0.15 | 2.11 | 0.036092 |
| | LOC_Os07g38090 | C3H | 0.15 | 2.19 | 0.029554 |
| | LOC_Os10g01470 | HD-ZIP | 0.14 | 2.03 | 0.043301 |
| | LOC_Os10g22950 | CAMTA | 0.14 | 2.20 | 0.028931 |
| | LOC_Os06g43090 | MYB | 0.14 | 2.00 | 0.046659 |
| | LOC_Os08g04390 | bHLH | 0.14 | 2.05 | 0.04161 |
| | LOC_Os08g26880 | bZIP | 0.14 | 2.08 | 0.038368 |
| | LOC_Os02g15340 | NAC | 0.14 | 2.08 | 0.038725 |
| | LOC_Os02g58440 | C3H | 0.14 | 2.05 | 0.041449 |
| | LOC_Os01g64730 | bZIP | 0.14 | 2.03 | 0.043884 |
| | LOC_Os08g38020 | bZIP | 0.14 | 2.04 | 0.04286 |
| | LOC_Os07g48260 | WRKY | 0.14 | 2.00 | 0.046922 |
| | LOC_Os04g53990 | bHLH | 0.14 | 2.06 | 0.040451 |
| | LOC_Os03g15660 | ERF | 0.13 | 1.97 | 0.049867 |
| | LOC_Os10g25170 | ERF | 0.13 | 1.97 | 0.049956 |
| | LOC_Os05g43380 | CPP | -0.13 | -2.01 | 0.045288 |
| | LOC_Os03g21800 | bZIP | -0.13 | -1.98 | 0.048808 |
| | LOC_Os01g68700 | bHLH | -0.13 | -2.02 | 0.044727 |
| | LOC_Os06g04870 | HD-ZIP | -0.13 | -2.02 | 0.044622 |
| | LOC_Os02g34680 | C2H2 | -0.14 | -2.04 | 0.04286 |
| | LOC_Os09g28310 | bZIP | -0.14 | -2.05 | 0.041722 |
| | LOC_Os01g09760 | MYB_related | -0.14 | -2.09 | 0.037416 |
| | LOC_Os05g51830 | C2H2 | -0.14 | -2.07 | 0.039314 |
| | LOC_Os02g42380 | TCP | -0.14 | -2.11 | 0.035867 |
| | LOC_Os04g40930 | Trihelix | -0.14 | -2.06 | 0.040253 |
| | LOC_Os04g45330 | YABBY | -0.14 | -2.00 | 0.04724 |
| | LOC_Os03g63810 | WRKY | -0.14 | -2.02 | 0.044858 |
| | LOC_Os08g38990 | WRKY | -0.14 | -2.18 | 0.030342 |
| | LOC_Os09g24820 | ZF-HD | -0.14 | -1.99 | 0.047895 |
| | LOC_Os08g41940 | SBP | -0.14 | -2.19 | 0.029898 |
| | LOC_Os06g07010 | FAR1 | -0.15 | -2.16 | 0.031658 |
| | LOC_Os01g68560 | M-type_MADS | -0.15 | -2.19 | 0.029945 |
| | LOC_Os06g08440 | ARR-B | -0.15 | -2.05 | 0.041555 |
| | LOC_Os11g06410 | Trihelix | -0.15 | -2.09 | 0.03824 |
| | LOC_Os02g06370 | Whirly | -0.15 | -2.11 | 0.036284 |
| | LOC_Os08g37904 | C2H2 | -0.15 | -2.08 | 0.038896 |
| | LOC_Os07g48660 | bZIP | -0.15 | -2.23 | 0.026505 |
| | LOC_Os05g37730 | MYB | -0.15 | -2.10 | 0.036847 |
| | LOC_Os05g48870 | ARF | -0.15 | -2.15 | 0.032717 |
| | LOC_Os02g19804 | C3H | -0.15 | -2.13 | 0.034178 |
| | LOC_Os09g35760 | HD-ZIP | -0.15 | -2.09 | 0.038288 |
| | LOC_Os06g46410 | ARF | -0.15 | -2.15 | 0.032878 |
| | LOC_Os04g52560 | FAR1 | -0.15 | -2.30 | 0.022293 |
| | LOC_Os12g41210 | CPP | -0.15 | -2.32 | 0.021393 |
| | LOC_Os04g46060 | WRKY | -0.15 | -2.19 | 0.029549 |
| | LOC_Os08g41950 | MIKC_MADS | -0.16 | -2.19 | 0.029884 |
| | LOC_Os03g56950 | bHLH | -0.16 | -2.37 | 0.018862 |
| | LOC_Os09g29460 | HD-ZIP | -0.16 | -2.23 | 0.027025 |
| | LOC_Os01g14720 | GeBP | -0.16 | -2.37 | 0.018653 |
| | LOC_Os05g03020 | C2H2 | -0.16 | -2.24 | 0.026004 |
| | LOC_Os04g55560 | AP2 | -0.16 | -2.37 | 0.018719 |
| | LOC_Os02g49440 | Dof | -0.16 | -2.40 | 0.017017 |
| | LOC_Os02g03960 | bZIP | -0.16 | -2.32 | 0.021421 |
| | LOC_Os03g08370 | FAR1 | -0.16 | -2.44 | 0.015395 |
| | LOC_Os03g58830 | bHLH | -0.16 | -2.35 | 0.019898 |
| | LOC_Os03g42420 | B3 | -0.16 | -2.32 | 0.021173 |
| | LOC_Os02g39540 | FAR1 | -0.17 | -2.50 | 0.012989 |
| | LOC_Os08g17400 | WRKY | -0.17 | -2.51 | 0.012933 |
| | LOC_Os03g01890 | HD-ZIP | -0.17 | -2.57 | 0.010959 |
| | LOC_Os03g55760 | G2-like | -0.17 | -2.50 | 0.013168 |
| | LOC_Os10g42490 | HD-ZIP | -0.17 | -2.57 | 0.010867 |
| | LOC_Os01g54990 | ARF | -0.17 | -2.53 | 0.012255 |
| | LOC_Os03g54170 | MIKC_MADS | -0.17 | -2.56 | 0.011242 |
| | LOC_Os04g28090 | MYB | -0.17 | -2.60 | 0.009918 |

| | | | | |
|---|---|---|---|---|
| LOC_Os05g50270 | GATA | -0.17 | -2.46 | 0.01491 |
| LOC_Os06g40150 | ERF | -0.18 | -2.51 | 0.01293 |
| LOC_Os01g57890 | HD-ZIP | -0.18 | -2.49 | 0.013741 |
| LOC_Os12g42400 | NF-YA | -0.18 | -2.67 | 0.008042 |
| LOC_Os08g31580 | ERF | -0.18 | -2.46 | 0.014698 |
| LOC_Os01g17000 | Dof | -0.18 | -2.67 | 0.008054 |
| LOC_Os06g01934 | TALE | -0.18 | -2.58 | 0.010752 |
| LOC_Os05g11414 | MIKC_MADS | -0.18 | -2.58 | 0.010593 |
| LOC_Os06g49010 | SBP | -0.18 | -2.54 | 0.011752 |
| LOC_Os02g47280 | GRF | -0.18 | -2.59 | 0.010419 |
| LOC_Os05g34310 | NAC | -0.18 | -2.65 | 0.008748 |
| LOC_Os11g06010 | bHLH | -0.19 | -2.61 | 0.009698 |
| LOC_Os06g49550 | FAR1 | -0.19 | -2.61 | 0.009854 |
| LOC_Os01g13300 | B3 | -0.19 | -2.81 | 0.005423 |
| LOC_Os12g41230 | CPP | -0.19 | -2.83 | 0.005072 |
| LOC_Os03g13600 | C2H2 | -0.19 | -2.84 | 0.004938 |
| LOC_Os09g01140 | GeBP | -0.19 | -2.69 | 0.007827 |
| LOC_Os02g47660 | bHLH | -0.19 | -2.84 | 0.004888 |
| LOC_Os07g42400 | FAR1 | -0.19 | -2.91 | 0.003953 |
| LOC_Os04g40060 | FAR1 | -0.19 | -2.92 | 0.003911 |
| LOC_Os12g38940 | C2H2 | -0.19 | -2.93 | 0.003736 |
| LOC_Os04g45650 | GATA | -0.19 | -2.93 | 0.003702 |
| LOC_Os09g39660 | C2H2 | -0.20 | -2.78 | 0.005988 |
| LOC_Os01g48700 | E2F/DP | -0.20 | -2.85 | 0.004778 |
| LOC_Os05g25320 | FAR1 | -0.20 | -2.84 | 0.004923 |
| LOC_Os03g42280 | B3 | -0.20 | -2.88 | 0.004385 |
| LOC_Os05g11510 | DBB | -0.20 | -2.88 | 0.004461 |
| LOC_Os01g34060 | MYB_related | -0.20 | -2.88 | 0.004358 |
| LOC_Os08g39890 | SBP | -0.20 | -3.07 | 0.002425 |
| LOC_Os03g27390 | bHLH | -0.20 | -3.10 | 0.002183 |
| LOC_Os03g43930 | HD-ZIP | -0.21 | -2.97 | 0.003335 |
| LOC_Os06g02560 | GRF | -0.21 | -3.13 | 0.001982 |
| LOC_Os11g35030 | GRF | -0.21 | -2.98 | 0.003241 |
| LOC_Os01g68860 | C3H | -0.21 | -3.22 | 0.001485 |
| LOC_Os12g06200 | E2F/DP | -0.21 | -3.23 | 0.001432 |
| LOC_Os08g01090 | B3 | -0.21 | -3.19 | 0.001623 |
| LOC_Os07g44690 | HSF | -0.21 | -2.95 | 0.003543 |
| LOC_Os05g50080 | C3H | -0.21 | -3.03 | 0.002755 |
| LOC_Os06g50900 | bHLH | -0.21 | -2.99 | 0.003122 |
| LOC_Os05g43920 | ARF | -0.21 | -3.06 | 0.002514 |
| LOC_Os12g18150 | C2H2 | -0.21 | -3.28 | 0.001191 |
| LOC_Os02g42870 | MYB | -0.21 | -3.10 | 0.002183 |
| LOC_Os04g35800 | C3H | -0.22 | -3.11 | 0.002165 |
| LOC_Os11g30484 | C2H2 | -0.22 | -3.11 | 0.002159 |
| LOC_Os03g50110 | GeBP | -0.22 | -3.29 | 0.001182 |
| LOC_Os01g48060 | ARF | -0.22 | -3.30 | 0.001131 |
| LOC_Os12g41950 | ARF | -0.22 | -3.24 | 0.001392 |
| LOC_Os04g57610 | ARF | -0.22 | -3.30 | 0.001125 |
| LOC_Os03g05480 | C2H2 | -0.22 | -3.31 | 0.001092 |
| LOC_Os10g41460 | Trihelix | -0.22 | -3.26 | 0.001282 |
| LOC_Os02g05510 | GATA | -0.22 | -3.39 | 0.000838 |
| LOC_Os03g62660 | FAR1 | -0.22 | -3.36 | 0.000922 |
| LOC_Os02g39710 | CO-like | -0.22 | -3.23 | 0.00146 |
| LOC_Os12g41920 | MYB_related | -0.23 | -3.46 | 0.000645 |
| LOC_Os05g43760 | TCP | -0.23 | -3.19 | 0.00167 |
| LOC_Os01g70310 | bHLH | -0.23 | -3.49 | 0.000581 |
| LOC_Os06g40710 | G2-like | -0.23 | -3.33 | 0.001041 |
| LOC_Os02g13310 | TALE | -0.23 | -3.28 | 0.001228 |
| LOC_Os01g32890 | GeBP | -0.23 | -3.44 | 0.000719 |
| LOC_Os01g67970 | C2H2 | -0.24 | -3.51 | 0.000545 |
| LOC_Os03g50900 | FAR1 | -0.24 | -3.63 | 0.000359 |
| LOC_Os07g04700 | MYB | -0.24 | -3.74 | 0.000234 |
| LOC_Os08g04170 | C3H | -0.24 | -3.79 | 0.000192 |
| LOC_Os11g11100 | bZIP | -0.25 | -3.56 | 0.000473 |
| LOC_Os03g15010 | FAR1 | -0.25 | -3.83 | 0.000169 |

| | | | | | |
|---|---|---|---|---|---|
| | LOC_Os04g11830 | TCP | -0.25 | -3.60 | 0.0004 |
| | LOC_Os02g18370 | FAR1 | -0.25 | -3.85 | 0.000153 |
| | LOC_Os06g04010 | BBR-BPC | -0.25 | -3.96 | 0.000101 |
| | LOC_Os06g12400 | HB-PHD | -0.25 | -3.74 | 0.000236 |
| | LOC_Os02g12790 | GATA | -0.26 | -3.74 | 0.000244 |
| | LOC_Os06g46890 | C3H | -0.26 | -3.85 | 0.000158 |
| | LOC_Os07g44640 | C2H2 | -0.27 | -4.03 | 7.61E-05 |
| | LOC_Os07g43420 | MYB | -0.27 | -4.17 | 4.4E-05 |
| | LOC_Os04g02730 | C3H | -0.27 | -3.92 | 0.00012 |
| | LOC_Os12g07120 | GATA | -0.27 | -4.16 | 4.63E-05 |
| | LOC_Os12g42970 | GATA | -0.27 | -4.10 | 5.96E-05 |
| | LOC_Os12g29520 | ARF | -0.27 | -4.20 | 3.78E-05 |
| | LOC_Os12g41860 | HD-ZIP | -0.27 | -4.20 | 3.89E-05 |
| | LOC_Os07g08140 | HSF | -0.27 | -4.25 | 3.16E-05 |
| | LOC_Os01g63160 | MYB | -0.28 | -4.26 | 3.01E-05 |
| | LOC_Os12g12380 | FAR1 | -0.29 | -4.45 | 1.35E-05 |
| | LOC_Os06g09420 | B3 | -0.29 | -4.34 | 2.27E-05 |
| | LOC_Os05g09630 | HB-other | -0.29 | -4.64 | 5.97E-06 |
| | LOC_Os10g17630 | B3 | -0.30 | -4.48 | 1.26E-05 |
| | LOC_Os02g18660 | GeBP | -0.31 | -4.58 | 8.13E-06 |
| | LOC_Os03g60120 | ERF | -0.31 | -4.92 | 1.7E-06 |
| | LOC_Os11g32100 | bHLH | -0.32 | -4.73 | 4.24E-06 |
| | LOC_Os03g12120 | NAC | -0.32 | -5.00 | 1.15E-06 |
| | LOC_Os08g19590 | HD-ZIP | -0.32 | -4.78 | 3.46E-06 |
| OS_5U_002 | LOC_Os08g15050 | CO-like | 0.57 | 9.67 | 2.21E-18 |
| | LOC_Os09g16510 | WRKY | 0.51 | 8.38 | 9.74E-15 |
| | LOC_Os07g48630 | EIL | 0.46 | 7.82 | 2.15E-13 |
| | LOC_Os06g04090 | NAC | 0.46 | 7.32 | 6.08E-12 |
| | LOC_Os10g01470 | HD-ZIP | 0.45 | 7.01 | 3.94E-11 |
| | LOC_Os01g50720 | MYB | 0.45 | 7.04 | 3.24E-11 |
| | LOC_Os05g48850 | NAC | 0.44 | 6.85 | 9.95E-11 |
| | LOC_Os09g28354 | HSF | 0.44 | 6.81 | 1.17E-10 |
| | LOC_Os08g02070 | MIKC_MADS | 0.42 | 6.93 | 4.56E-11 |
| | LOC_Os02g13800 | HSF | 0.42 | 6.47 | 7.85E-10 |
| | LOC_Os05g37190 | C2H2 | 0.41 | 6.78 | 1.08E-10 |
| | LOC_Os03g53020 | bHLH | 0.41 | 6.70 | 1.72E-10 |
| | LOC_Os02g56600 | NAC | 0.41 | 6.72 | 1.49E-10 |
| | LOC_Os03g08490 | ERF | 0.41 | 6.28 | 2.08E-09 |
| | LOC_Os09g10840 | bZIP | 0.40 | 6.05 | 7.41E-09 |
| | LOC_Os01g09640 | MYB_related | 0.39 | 6.06 | 6.76E-09 |
| | LOC_Os07g22730 | ERF | 0.39 | 5.78 | 3.07E-08 |
| | LOC_Os12g41680 | NAC | 0.39 | 6.13 | 4.23E-09 |
| | LOC_Os01g43550 | WRKY | 0.38 | 6.18 | 2.99E-09 |
| | LOC_Os01g09100 | WRKY | 0.38 | 5.76 | 3.19E-08 |
| | LOC_Os03g20900 | G2-like | 0.38 | 6.17 | 3.11E-09 |
| | LOC_Os05g03760 | C3H | 0.38 | 5.77 | 3.05E-08 |
| | LOC_Os01g67480 | bHLH | 0.37 | 5.68 | 4.64E-08 |
| | LOC_Os01g09550 | NAC | 0.37 | 5.92 | 1.17E-08 |
| | LOC_Os07g42510 | ERF | 0.37 | 5.65 | 5.32E-08 |
| | LOC_Os03g64260 | ERF | 0.37 | 5.61 | 6.82E-08 |
| | LOC_Os05g48010 | MYB | 0.37 | 5.59 | 7.42E-08 |
| | LOC_Os01g09590 | MYB | 0.36 | 5.44 | 1.56E-07 |
| | LOC_Os07g48550 | NAC | 0.36 | 5.75 | 2.88E-08 |
| | LOC_Os05g38140 | bHLH | 0.36 | 5.41 | 1.79E-07 |
| | LOC_Os09g29820 | bZIP | 0.36 | 5.35 | 2.48E-07 |
| | LOC_Os01g39330 | bHLH | 0.35 | 5.36 | 2.23E-07 |
| | LOC_Os03g56580 | NAC | 0.35 | 5.56 | 7.68E-08 |
| | LOC_Os02g47190 | G2-like | 0.35 | 5.29 | 3.18E-07 |
| | LOC_Os08g02300 | NAC | 0.35 | 5.47 | 1.22E-07 |
| | LOC_Os05g38120 | TALE | 0.35 | 5.14 | 6.66E-07 |
| | LOC_Os03g55540 | C2H2 | 0.34 | 5.47 | 1.22E-07 |
| | LOC_Os07g48596 | G2-like | 0.34 | 4.89 | 2.16E-06 |
| | LOC_Os05g34830 | NAC | 0.34 | 4.87 | 2.39E-06 |
| | LOC_Os05g33810 | SBP | 0.33 | 5.15 | 5.8E-07 |
| | LOC_Os01g43650 | WRKY | 0.33 | 5.28 | 3.04E-07 |

| | | | | |
|---|---|---|---|---|
| LOC_Os05g45410 | HSF | 0.33 | 4.83 | 2.8E-06 |
| LOC_Os05g02420 | MYB_related | 0.33 | 5.02 | 1.1E-06 |
| LOC_Os04g45690 | DBB | 0.33 | 5.23 | 3.81E-07 |
| LOC_Os08g36920 | ERF | 0.33 | 4.91 | 1.89E-06 |
| LOC_Os03g32230 | C2H2 | 0.33 | 5.18 | 4.95E-07 |
| LOC_Os04g36054 | ARF | 0.32 | 4.64 | 6.62E-06 |
| LOC_Os04g43560 | NAC | 0.32 | 4.75 | 3.96E-06 |
| LOC_Os01g52540 | B3 | 0.32 | 4.78 | 3.39E-06 |
| LOC_Os04g43680 | MYB | 0.32 | 5.15 | 5.74E-07 |
| LOC_Os09g25060 | WRKY | 0.32 | 4.79 | 3.2E-06 |
| LOC_Os03g10210 | HD-ZIP | 0.32 | 4.75 | 3.95E-06 |
| LOC_Os08g39630 | bHLH | 0.32 | 4.82 | 2.79E-06 |
| LOC_Os04g53990 | bHLH | 0.32 | 5.04 | 9.63E-07 |
| LOC_Os09g01960 | MYB | 0.32 | 4.68 | 5.4E-06 |
| LOC_Os10g39030 | TALE | 0.31 | 4.86 | 2.22E-06 |
| LOC_Os04g38720 | NAC | 0.31 | 4.70 | 4.85E-06 |
| LOC_Os01g70110 | NAC | 0.31 | 4.48 | 1.31E-05 |
| LOC_Os03g17150 | C2H2 | 0.31 | 4.69 | 4.88E-06 |
| LOC_Os02g43970 | ERF | 0.31 | 4.41 | 1.78E-05 |
| LOC_Os01g11910 | bHLH | 0.31 | 4.50 | 1.17E-05 |
| LOC_Os12g43950 | TALE | 0.31 | 4.77 | 3.4E-06 |
| LOC_Os06g44010 | WRKY | 0.31 | 4.52 | 1.09E-05 |
| LOC_Os05g34050 | bZIP | 0.31 | 4.62 | 6.63E-06 |
| LOC_Os09g35910 | HD-ZIP | 0.31 | 4.42 | 1.64E-05 |
| LOC_Os10g41230 | HD-ZIP | 0.31 | 4.33 | 2.41E-05 |
| LOC_Os01g51690 | WRKY | 0.30 | 4.56 | 8.84E-06 |
| LOC_Os10g26620 | Dof | 0.30 | 4.79 | 3.01E-06 |
| LOC_Os01g64360 | MYB | 0.30 | 4.25 | 3.33E-05 |
| LOC_Os06g24070 | G2-like | 0.30 | 4.37 | 2.03E-05 |
| LOC_Os04g50770 | MYB | 0.29 | 4.34 | 2.29E-05 |
| LOC_Os06g46270 | NAC | 0.29 | 4.36 | 2.1E-05 |
| LOC_Os06g12230 | TCP | 0.29 | 4.28 | 2.87E-05 |
| LOC_Os05g37080 | NAC | 0.29 | 4.50 | 1.13E-05 |
| LOC_Os07g22770 | ERF | 0.29 | 4.54 | 9.36E-06 |
| LOC_Os10g38820 | bZIP | 0.29 | 4.29 | 2.76E-05 |
| LOC_Os09g36250 | MYB | 0.29 | 4.21 | 3.97E-05 |
| LOC_Os08g34360 | ERF | 0.29 | 4.26 | 3.09E-05 |
| LOC_Os01g09990 | bHLH | 0.29 | 4.24 | 3.42E-05 |
| LOC_Os08g38020 | bZIP | 0.29 | 4.43 | 1.47E-05 |
| LOC_Os01g65080 | C2H2 | 0.29 | 4.47 | 1.23E-05 |
| LOC_Os05g51160 | MYB_related | 0.28 | 4.22 | 3.7E-05 |
| LOC_Os07g48820 | bZIP | 0.28 | 4.19 | 4.23E-05 |
| LOC_Os10g42130 | NAC | 0.28 | 4.03 | 8.02E-05 |
| LOC_Os01g53040 | WRKY | 0.28 | 4.36 | 2.03E-05 |
| LOC_Os11g29870 | WRKY | 0.28 | 4.09 | 6.24E-05 |
| LOC_Os03g44710 | YABBY | 0.28 | 4.38 | 1.8E-05 |
| LOC_Os06g49040 | G2-like | 0.28 | 4.04 | 7.77E-05 |
| LOC_Os01g01870 | bHLH | 0.28 | 4.32 | 2.32E-05 |
| LOC_Os04g56990 | G2-like | 0.28 | 4.19 | 4.07E-05 |
| LOC_Os11g05614 | NAC | 0.28 | 4.03 | 7.93E-05 |
| LOC_Os09g33490 | NAC | 0.28 | 4.01 | 8.57E-05 |
| LOC_Os08g39980 | MYB_related | 0.28 | 4.27 | 2.85E-05 |
| LOC_Os02g39140 | bHLH | 0.27 | 3.98 | 9.59E-05 |
| LOC_Os08g19650 | TALE | 0.27 | 3.98 | 9.64E-05 |
| LOC_Os01g69830 | SBP | 0.27 | 3.91 | 0.00013 |
| LOC_Os04g47860 | C2H2 | 0.27 | 4.16 | 4.65E-05 |
| LOC_Os01g48130 | NAC | 0.27 | 3.98 | 9.66E-05 |
| LOC_Os09g31390 | bZIP | 0.27 | 3.85 | 0.000162 |
| LOC_Os01g63980 | C2H2 | 0.26 | 4.04 | 7.51E-05 |
| LOC_Os09g28440 | ERF | 0.26 | 3.80 | 0.000194 |
| LOC_Os08g29660 | WRKY | 0.26 | 4.03 | 7.56E-05 |
| LOC_Os02g08500 | ARR-B | 0.26 | 3.67 | 0.000315 |
| LOC_Os02g43790 | ERF | 0.26 | 3.77 | 0.000212 |
| LOC_Os03g21710 | WRKY | 0.25 | 3.73 | 0.000253 |
| LOC_Os09g26420 | ERF | 0.25 | 3.77 | 0.000214 |

| | | | | | |
|---|---|---|---|---|---|
| | LOC_Os05g03884 | TALE | 0.25 | 3.70 | 0.000284 |
| | LOC_Os09g38340 | C2H2 | 0.25 | 3.70 | 0.000281 |
| | LOC_Os08g32080 | HD-ZIP | 0.25 | 3.59 | 0.000416 |
| | LOC_Os08g04390 | bHLH | 0.25 | 3.81 | 0.000182 |
| | LOC_Os05g07120 | bHLH | 0.25 | 3.74 | 0.000237 |
| | LOC_Os05g37060 | MYB | 0.25 | 3.65 | 0.000329 |
| | LOC_Os12g37970 | MYB | 0.25 | 3.83 | 0.000166 |
| | LOC_Os03g21030 | NAC | 0.25 | 3.56 | 0.000464 |
| | LOC_Os01g13740 | G2-like | 0.25 | 3.78 | 0.000198 |
| | LOC_Os08g26880 | bZIP | 0.25 | 3.76 | 0.00022 |
| | LOC_Os03g29614 | MYB | 0.24 | 3.40 | 0.000817 |
| | LOC_Os01g54600 | WRKY | 0.24 | 3.58 | 0.000436 |
| | LOC_Os01g74140 | WRKY | 0.24 | 3.62 | 0.000371 |
| | LOC_Os10g25170 | ERF | 0.24 | 3.70 | 0.000269 |
| | LOC_Os05g49620 | WRKY | 0.24 | 3.49 | 0.000588 |
| | LOC_Os01g40260 | WRKY | 0.24 | 3.45 | 0.000696 |
| | LOC_Os03g37920 | FAR1 | 0.24 | 3.59 | 0.000402 |
| | LOC_Os07g38240 | C2H2 | 0.23 | 3.45 | 0.000691 |
| | LOC_Os08g33750 | G2-like | 0.23 | 3.54 | 0.000493 |
| | LOC_Os10g33810 | MYB | 0.23 | 3.26 | 0.001332 |
| | LOC_Os03g07880 | NF-YA | 0.23 | 3.49 | 0.000579 |
| | LOC_Os03g19370 | bZIP | 0.23 | 3.57 | 0.000438 |
| | LOC_Os10g39750 | bHLH | 0.23 | 3.37 | 0.000891 |
| | LOC_Os04g50920 | WRKY | 0.23 | 3.37 | 0.000907 |
| | LOC_Os07g48570 | Dof | 0.23 | 3.53 | 0.000506 |
| | LOC_Os02g02820 | bHLH | 0.23 | 3.52 | 0.000527 |
| | LOC_Os02g36880 | NAC | 0.23 | 3.27 | 0.001291 |
| | LOC_Os12g06080 | B3 | 0.23 | 3.46 | 0.000653 |
| | LOC_Os02g44130 | C2H2 | 0.22 | 3.23 | 0.001447 |
| | LOC_Os04g41560 | DBB | 0.22 | 3.15 | 0.0019 |
| | LOC_Os12g01490 | G2-like | 0.22 | 3.22 | 0.001473 |
| | LOC_Os08g42470 | bHLH | 0.22 | 3.40 | 0.000811 |
| | LOC_Os05g02150 | Dof | 0.22 | 3.21 | 0.001541 |
| | LOC_Os05g04820 | MYB | 0.22 | 3.25 | 0.001369 |
| | LOC_Os01g74410 | MYB | 0.22 | 3.38 | 0.000868 |
| | LOC_Os03g08470 | ERF | 0.22 | 3.35 | 0.000938 |
| | LOC_Os01g61080 | WRKY | 0.22 | 3.33 | 0.001023 |
| | LOC_Os04g54474 | bZIP | 0.22 | 3.16 | 0.001823 |
| | LOC_Os01g09280 | MYB_related | 0.22 | 3.36 | 0.000917 |
| | LOC_Os06g09390 | ERF | 0.22 | 3.07 | 0.002463 |
| | LOC_Os01g07120 | ERF | 0.22 | 3.18 | 0.001676 |
| | LOC_Os03g05500 | LBD | 0.22 | 3.10 | 0.002232 |
| | LOC_Os01g14440 | WRKY | 0.22 | 3.29 | 0.001151 |
| | LOC_Os06g35140 | G2-like | 0.21 | 3.08 | 0.002365 |
| | LOC_Os06g09370 | bHLH | 0.21 | 3.08 | 0.002343 |
| | LOC_Os02g52670 | ERF | 0.21 | 3.23 | 0.001415 |
| | LOC_Os03g22170 | ERF | 0.21 | 3.20 | 0.001566 |
| | LOC_Os02g35770 | HD-ZIP | 0.21 | 3.17 | 0.001719 |
| | LOC_Os07g36170 | GRAS | 0.21 | 3.19 | 0.001608 |
| | LOC_Os04g46020 | GATA | 0.21 | 3.14 | 0.001941 |
| | LOC_Os01g15640 | NAC | 0.21 | 3.09 | 0.002304 |
| | LOC_Os01g58420 | ERF | 0.21 | 3.18 | 0.001686 |
| | LOC_Os02g40530 | MYB | 0.21 | 3.14 | 0.001946 |
| | LOC_Os07g39470 | GRAS | 0.21 | 3.08 | 0.002321 |
| | LOC_Os04g51320 | Trihelix | 0.21 | 3.15 | 0.001849 |
| | LOC_Os05g41760 | ERF | 0.20 | 3.05 | 0.002564 |
| | LOC_Os09g35030 | ERF | 0.20 | 2.90 | 0.004182 |
| | LOC_Os03g55080 | WRKY | 0.20 | 3.09 | 0.002271 |
| | LOC_Os03g46860 | bHLH | 0.20 | 2.89 | 0.004314 |
| | LOC_Os08g10080 | NAC | 0.20 | 2.86 | 0.004711 |
| | LOC_Os01g53260 | WRKY | 0.20 | 2.85 | 0.00488 |
| | LOC_Os01g09850 | C2H2 | 0.20 | 2.79 | 0.00587 |
| | LOC_Os11g06170 | bZIP | 0.20 | 2.84 | 0.00492 |
| | LOC_Os11g03300 | NAC | 0.20 | 2.81 | 0.005478 |
| | LOC_Os06g45890 | G2-like | 0.20 | 2.82 | 0.005354 |

| | | | | | |
|---|---|---|---|---|---|
| | LOC_Os09g11480 | ERF | 0.20 | 2.83 | 0.005099 |
| | LOC_Os01g51140 | bHLH | 0.19 | 2.74 | 0.006754 |
| | LOC_Os02g51280 | TCP | 0.19 | 2.95 | 0.003556 |
| | LOC_Os02g57650 | NAC | 0.19 | 2.78 | 0.00596 |
| | LOC_Os04g45810 | HD-ZIP | 0.19 | 2.91 | 0.00398 |
| | LOC_Os01g18870 | bHLH | 0.19 | 2.92 | 0.003857 |
| | LOC_Os05g49420 | bZIP | 0.19 | 2.75 | 0.006602 |
| | LOC_Os07g12340 | NAC | 0.19 | 2.89 | 0.004238 |
| | LOC_Os12g39330 | ERF | 0.19 | 2.60 | 0.009943 |
| | LOC_Os03g20780 | EIL | 0.19 | 2.69 | 0.00782 |
| | LOC_Os09g28210 | bHLH | 0.19 | 2.73 | 0.006958 |
| | LOC_Os02g52190 | bHLH | 0.19 | 2.57 | 0.010865 |
| | LOC_Os10g42850 | WRKY | 0.19 | 2.69 | 0.007827 |
| | LOC_Os04g49110 | GRAS | 0.18 | 2.81 | 0.005344 |
| | LOC_Os05g42130 | GRAS | 0.18 | 2.64 | 0.009017 |
| | LOC_Os02g45450 | ERF | 0.18 | 2.57 | 0.010841 |
| | LOC_Os01g13540 | Nin-like | 0.18 | 2.78 | 0.005899 |
| | LOC_Os03g43840 | LSD | 0.18 | 2.66 | 0.008374 |
| | LOC_Os04g46220 | ERF | 0.18 | 2.55 | 0.011579 |
| | LOC_Os03g12350 | ARR-B | 0.18 | 2.64 | 0.008975 |
| | LOC_Os02g49700 | HD-ZIP | 0.18 | 2.49 | 0.013603 |
| | LOC_Os09g38570 | Trihelix | 0.18 | 2.55 | 0.011447 |
| | LOC_Os07g39800 | HRT-like | 0.18 | 2.72 | 0.007103 |
| | LOC_Os11g06840 | C2H2 | 0.18 | 2.63 | 0.009186 |
| | LOC_Os01g41900 | MYB_related | 0.18 | 2.49 | 0.013546 |
| | LOC_Os03g15440 | bHLH | 0.18 | 2.65 | 0.008503 |
| | LOC_Os10g39130 | MIKC_MADS | 0.17 | 2.65 | 0.008678 |
| | LOC_Os01g06640 | bHLH | 0.17 | 2.62 | 0.009471 |
| | LOC_Os07g41720 | NF-YA | 0.17 | 2.58 | 0.010486 |
| | LOC_Os01g18240 | MYB | 0.17 | 2.45 | 0.015325 |
| | LOC_Os01g50110 | MYB | 0.17 | 2.53 | 0.012276 |
| | LOC_Os07g03770 | TALE | 0.17 | 2.41 | 0.016742 |
| | LOC_Os03g04310 | bHLH | 0.17 | 2.55 | 0.011585 |
| | LOC_Os01g10370 | ERF | 0.17 | 2.53 | 0.012219 |
| | LOC_Os03g06930 | TALE | 0.17 | 2.55 | 0.011305 |
| | LOC_Os03g09100 | CAMTA | 0.17 | 2.55 | 0.011368 |
| | LOC_Os05g35170 | NAC | 0.17 | 2.42 | 0.016443 |
| | LOC_Os01g07930 | C3H | 0.17 | 2.39 | 0.017724 |
| | LOC_Os03g61760 | SBP | 0.17 | 2.46 | 0.014629 |
| | LOC_Os10g22430 | GRAS | 0.17 | 2.54 | 0.011806 |
| | LOC_Os11g05480 | bZIP | 0.16 | 2.29 | 0.023172 |
| | LOC_Os11g02480 | WRKY | 0.16 | 2.30 | 0.022817 |
| | LOC_Os03g17810 | LBD | 0.16 | 2.30 | 0.022393 |
| | LOC_Os01g70870 | C2H2 | 0.16 | 2.31 | 0.021789 |
| | LOC_Os09g12770 | G2-like | 0.16 | 2.33 | 0.020581 |
| | LOC_Os06g45140 | bZIP | 0.16 | 2.26 | 0.024902 |
| | LOC_Os02g49986 | MYB | 0.16 | 2.21 | 0.02851 |
| | LOC_Os01g14870 | C3H | 0.16 | 2.26 | 0.024732 |
| | LOC_Os07g48450 | NAC | 0.16 | 2.32 | 0.021475 |
| | LOC_Os06g07020 | C2H2 | 0.15 | 2.34 | 0.020317 |
| | LOC_Os04g44670 | ERF | 0.15 | 2.18 | 0.030508 |
| | LOC_Os03g15660 | ERF | 0.15 | 2.28 | 0.023875 |
| | LOC_Os04g56170 | SBP | 0.15 | 2.16 | 0.03203 |
| | LOC_Os02g43300 | Trihelix | 0.15 | 2.13 | 0.034304 |
| | LOC_Os03g26210 | bHLH | 0.15 | 2.26 | 0.02494 |
| | LOC_Os03g08960 | HD-ZIP | 0.15 | 2.24 | 0.026369 |
| | LOC_Os01g36460 | MYB | 0.15 | 2.05 | 0.041675 |
| | LOC_Os09g32040 | NAC | 0.15 | 2.08 | 0.038738 |
| | LOC_Os07g48260 | WRKY | 0.15 | 2.14 | 0.033793 |
| | LOC_Os11g03420 | ZF-HD | 0.14 | 2.04 | 0.042528 |
| | LOC_Os01g60640 | WRKY | 0.14 | 2.04 | 0.043101 |
| | LOC_Os02g34970 | NAC | 0.14 | 2.15 | 0.03296 |
| | LOC_Os09g25070 | WRKY | 0.14 | 2.14 | 0.03345 |
| | LOC_Os08g01330 | NAC | 0.14 | 2.13 | 0.034279 |
| | LOC_Os03g47740 | TALE | 0.14 | 2.13 | 0.034374 |

| | | | | | |
|---|---|---|---|---|---|
| | LOC_Os02g43330 | HD-ZIP | 0.14 | 1.99 | 0.047731 |
| | LOC_Os04g46440 | ERF | 0.14 | 2.07 | 0.039757 |
| | LOC_Os04g59380 | C2H2 | 0.14 | 1.98 | 0.04941 |
| | LOC_Os03g48970 | NF-YA | 0.14 | 2.05 | 0.041393 |
| | LOC_Os11g03370 | NAC | 0.13 | 2.01 | 0.045949 |
| | LOC_Os02g36360 | C2H2 | 0.13 | 1.98 | 0.048885 |
| | LOC_Os06g03710 | GRAS | 0.13 | 1.98 | 0.04907 |
| | LOC_Os02g55320 | ARR-B | 0.13 | 1.97 | 0.049928 |
| | LOC_Os04g46400 | ERF | 0.13 | 1.97 | 0.049818 |
| | LOC_Os12g12380 | FAR1 | -0.13 | -2.01 | 0.045798 |
| | LOC_Os03g62660 | FAR1 | -0.13 | -2.00 | 0.046472 |
| | LOC_Os11g09160 | B3 | -0.14 | -2.01 | 0.046196 |
| | LOC_Os04g55560 | AP2 | -0.14 | -2.11 | 0.035764 |
| | LOC_Os05g43380 | CPP | -0.14 | -2.17 | 0.031013 |
| | LOC_Os01g68560 | M-type_MADS | -0.14 | -2.12 | 0.034913 |
| | LOC_Os12g10660 | DBB | -0.15 | -2.20 | 0.028725 |
| | LOC_Os01g17000 | Dof | -0.15 | -2.21 | 0.028279 |
| | LOC_Os06g07010 | FAR1 | -0.15 | -2.19 | 0.029386 |
| | LOC_Os04g59430 | ARF | -0.15 | -2.25 | 0.025745 |
| | LOC_Os02g38470 | B3 | -0.15 | -2.07 | 0.039374 |
| | LOC_Os06g11860 | ERF | -0.15 | -2.10 | 0.036625 |
| | LOC_Os02g42870 | MYB | -0.15 | -2.14 | 0.033778 |
| | LOC_Os02g39520 | FAR1 | -0.15 | -2.21 | 0.02853 |
| | LOC_Os01g70810 | HB-other | -0.15 | -2.23 | 0.026928 |
| | LOC_Os07g13170 | AP2 | -0.15 | -2.17 | 0.031441 |
| | LOC_Os03g58250 | bZIP | -0.15 | -2.28 | 0.02363 |
| | LOC_Os06g49550 | FAR1 | -0.16 | -2.17 | 0.031079 |
| | LOC_Os12g18150 | C2H2 | -0.16 | -2.39 | 0.017498 |
| | LOC_Os02g06910 | ARF | -0.16 | -2.42 | 0.016385 |
| | LOC_Os12g41860 | HD-ZIP | -0.16 | -2.41 | 0.01695 |
| | LOC_Os04g47890 | G2-like | -0.16 | -2.46 | 0.014503 |
| | LOC_Os01g61810 | NF-YB | -0.16 | -2.26 | 0.025113 |
| | LOC_Os01g68160 | C2H2 | -0.16 | -2.37 | 0.018925 |
| | LOC_Os04g45650 | GATA | -0.16 | -2.46 | 0.014716 |
| | LOC_Os02g05510 | GATA | -0.17 | -2.52 | 0.012471 |
| | LOC_Os01g48700 | E2F/DP | -0.17 | -2.42 | 0.016267 |
| | LOC_Os09g13940 | ERF | -0.17 | -2.40 | 0.017349 |
| | LOC_Os11g32100 | bHLH | -0.17 | -2.43 | 0.015921 |
| | LOC_Os11g39000 | bHLH | -0.17 | -2.53 | 0.012274 |
| | LOC_Os01g64560 | bHLH | -0.17 | -2.56 | 0.011104 |
| | LOC_Os03g54160 | MIKC_MADS | -0.17 | -2.40 | 0.017173 |
| | LOC_Os09g29460 | HD-ZIP | -0.17 | -2.43 | 0.016007 |
| | LOC_Os06g04870 | HD-ZIP | -0.17 | -2.58 | 0.010637 |
| | LOC_Os09g24820 | ZF-HD | -0.18 | -2.44 | 0.0155 |
| | LOC_Os06g41770 | bZIP | -0.18 | -2.59 | 0.010342 |
| | LOC_Os05g27930 | ERF | -0.18 | -2.50 | 0.013191 |
| | LOC_Os08g04170 | C3H | -0.18 | -2.73 | 0.006775 |
| | LOC_Os01g06550 | NF-X1 | -0.18 | -2.70 | 0.007379 |
| | LOC_Os09g35760 | HD-ZIP | -0.18 | -2.48 | 0.014222 |
| | LOC_Os05g50080 | C3H | -0.18 | -2.57 | 0.010925 |
| | LOC_Os03g63750 | HSF | -0.18 | -2.72 | 0.007006 |
| | LOC_Os03g20550 | WRKY | -0.18 | -2.74 | 0.006721 |
| | LOC_Os05g49930 | GRAS | -0.18 | -2.59 | 0.010275 |
| | LOC_Os01g09620 | C3H | -0.18 | -2.60 | 0.01004 |
| | LOC_Os08g41950 | MIKC_MADS | -0.18 | -2.56 | 0.011094 |
| | LOC_Os03g42420 | B3 | -0.18 | -2.60 | 0.01003 |
| | LOC_Os03g11370 | B3 | -0.18 | -2.60 | 0.01005 |
| | LOC_Os04g57600 | C3H | -0.19 | -2.83 | 0.00509 |
| | LOC_Os03g56110 | TALE | -0.19 | -2.67 | 0.008322 |
| | LOC_Os12g41230 | CPP | -0.19 | -2.81 | 0.005396 |
| | LOC_Os01g15350 | C3H | -0.19 | -2.89 | 0.004165 |
| | LOC_Os02g42380 | TCP | -0.19 | -2.90 | 0.004065 |
| | LOC_Os03g58830 | bHLH | -0.19 | -2.77 | 0.006073 |
| | LOC_Os01g72490 | SRS | -0.19 | -2.76 | 0.006306 |
| | LOC_Os01g13300 | B3 | -0.19 | -2.87 | 0.004513 |

| | | | | |
|---|---|---|---|---|
| LOC_Os03g55220 | bHLH | -0.19 | -2.88 | 0.004381 |
| LOC_Os02g34630 | MYB_related | -0.20 | -2.92 | 0.00389 |
| LOC_Os12g38940 | C2H2 | -0.20 | -2.97 | 0.003316 |
| LOC_Os03g27390 | bHLH | -0.20 | -3.00 | 0.00301 |
| LOC_Os03g21800 | bZIP | -0.20 | -2.97 | 0.003276 |
| LOC_Os05g41450 | NF-YC | -0.20 | -2.87 | 0.004588 |
| LOC_Os03g54170 | MIKC_MADS | -0.20 | -3.07 | 0.002377 |
| LOC_Os06g49080 | C3H | -0.20 | -2.95 | 0.00358 |
| LOC_Os03g51970 | GRF | -0.20 | -3.04 | 0.002663 |
| LOC_Os08g42440 | CO-like | -0.21 | -3.18 | 0.001684 |
| LOC_Os12g41950 | ARF | -0.21 | -3.08 | 0.002352 |
| LOC_Os06g40960 | C2H2 | -0.21 | -2.98 | 0.003213 |
| LOC_Os03g60120 | ERF | -0.21 | -3.16 | 0.001777 |
| LOC_Os04g40060 | FAR1 | -0.21 | -3.20 | 0.001568 |
| LOC_Os03g05690 | C2H2 | -0.21 | -3.18 | 0.001681 |
| LOC_Os05g38460 | MYB | -0.21 | -3.06 | 0.002533 |
| LOC_Os02g05450 | HB-other | -0.21 | -3.11 | 0.002134 |
| LOC_Os02g33750 | FAR1 | -0.22 | -3.21 | 0.001538 |
| LOC_Os01g42710 | LSD | -0.22 | -3.41 | 0.00076 |
| LOC_Os05g25320 | FAR1 | -0.22 | -3.22 | 0.001484 |
| LOC_Os08g37920 | C2H2 | -0.23 | -3.40 | 0.000809 |
| LOC_Os04g52560 | FAR1 | -0.23 | -3.45 | 0.000668 |
| LOC_Os03g11614 | MIKC_MADS | -0.23 | -3.16 | 0.001835 |
| LOC_Os06g02560 | GRF | -0.23 | -3.46 | 0.00064 |
| LOC_Os05g51830 | C2H2 | -0.23 | -3.47 | 0.000631 |
| LOC_Os02g18660 | GeBP | -0.23 | -3.33 | 0.001019 |
| LOC_Os03g53340 | HSF | -0.23 | -3.60 | 0.000385 |
| LOC_Os04g53540 | HD-ZIP | -0.23 | -3.39 | 0.000852 |
| LOC_Os01g32890 | GeBP | -0.24 | -3.45 | 0.000685 |
| LOC_Os02g47280 | GRF | -0.24 | -3.45 | 0.000685 |
| LOC_Os02g45850 | B3 | -0.24 | -3.51 | 0.000551 |
| LOC_Os04g11830 | TCP | -0.24 | -3.51 | 0.000551 |
| LOC_Os02g06370 | Whirly | -0.24 | -3.53 | 0.000508 |
| LOC_Os06g41384 | C3H | -0.24 | -3.42 | 0.000782 |
| LOC_Os09g01140 | GeBP | -0.24 | -3.52 | 0.00054 |
| LOC_Os06g46410 | ARF | -0.25 | -3.52 | 0.000536 |
| LOC_Os02g19804 | C3H | -0.25 | -3.60 | 0.000404 |
| LOC_Os04g36590 | FAR1 | -0.25 | -3.63 | 0.00036 |
| LOC_Os11g35030 | GRF | -0.25 | -3.65 | 0.000335 |
| LOC_Os03g50110 | GeBP | -0.25 | -3.89 | 0.000133 |
| LOC_Os08g28214 | CPP | -0.25 | -3.63 | 0.000359 |
| LOC_Os02g39540 | FAR1 | -0.25 | -3.93 | 0.000115 |
| LOC_Os06g04010 | BBR-BPC | -0.25 | -3.97 | 9.67E-05 |
| LOC_Os07g43030 | CAMTA | -0.26 | -3.87 | 0.000142 |
| LOC_Os01g63160 | MYB | -0.26 | -3.88 | 0.000137 |
| LOC_Os07g44640 | C2H2 | -0.26 | -3.90 | 0.000127 |
| LOC_Os06g45840 | MYB_related | -0.26 | -3.84 | 0.000165 |
| LOC_Os03g13614 | bZIP | -0.26 | -3.81 | 0.000187 |
| LOC_Os01g14720 | GeBP | -0.26 | -4.03 | 7.64E-05 |
| LOC_Os10g17630 | B3 | -0.26 | -3.96 | 0.000105 |
| LOC_Os03g06860 | FAR1 | -0.26 | -4.05 | 7.11E-05 |
| LOC_Os07g08140 | HSF | -0.27 | -4.13 | 5.15E-05 |
| LOC_Os06g12400 | HB-PHD | -0.28 | -4.10 | 6.1E-05 |
| LOC_Os08g19590 | HD-ZIP | -0.28 | -4.03 | 8.09E-05 |
| LOC_Os02g33610 | Trihelix | -0.28 | -3.96 | 0.000109 |
| LOC_Os12g41920 | MYB_related | -0.28 | -4.36 | 1.95E-05 |
| LOC_Os02g18370 | FAR1 | -0.28 | -4.37 | 1.91E-05 |
| LOC_Os06g16400 | bHLH | -0.28 | -4.38 | 1.88E-05 |
| LOC_Os01g09760 | MYB_related | -0.29 | -4.51 | 1.05E-05 |
| LOC_Os03g12120 | NAC | -0.29 | -4.48 | 1.19E-05 |
| LOC_Os08g37904 | C2H2 | -0.29 | -4.18 | 4.43E-05 |
| LOC_Os06g14190 | NF-X1 | -0.29 | -4.27 | 3.07E-05 |
| LOC_Os04g28090 | MYB | -0.29 | -4.57 | 8.08E-06 |
| LOC_Os02g10840 | FAR1 | -0.29 | -4.62 | 6.44E-06 |
| LOC_Os08g01090 | B3 | -0.30 | -4.60 | 7.04E-06 |

| | | | | | |
|---|---|---|---|---|---|
| | LOC_Os04g02730 | C3H | -0.30 | -4.52 | 1.08E-05 |
| | LOC_Os01g67970 | C2H2 | -0.30 | -4.54 | 9.77E-06 |
| | LOC_Os04g35800 | C3H | -0.31 | -4.59 | 7.78E-06 |
| | LOC_Os05g09630 | HB-other | -0.31 | -4.93 | 1.57E-06 |
| | LOC_Os04g40930 | Trihelix | -0.31 | -4.75 | 3.74E-06 |
| | LOC_Os03g42280 | B3 | -0.32 | -4.71 | 4.63E-06 |
| | LOC_Os03g63810 | WRKY | -0.32 | -4.66 | 5.85E-06 |
| | LOC_Os10g42490 | HD-ZIP | -0.32 | -5.02 | 1.04E-06 |
| | LOC_Os07g37630 | FAR1 | -0.32 | -5.00 | 1.19E-06 |
| | LOC_Os06g46890 | C3H | -0.33 | -4.84 | 2.67E-06 |
| | LOC_Os12g42970 | GATA | -0.34 | -5.25 | 3.65E-07 |
| | LOC_Os03g05480 | C2H2 | -0.34 | -5.44 | 1.43E-07 |
| | LOC_Os08g23470 | B3 | -0.35 | -5.27 | 3.46E-07 |
| | LOC_Os03g50900 | FAR1 | -0.37 | -5.85 | 1.86E-08 |
| | LOC_Os02g33560 | bZIP | -0.39 | -6.35 | 1.18E-09 |
| RY motif | LOC_Os03g17150 | C2H2 | 0.47 | 7.72 | 5.03E-13 |
| | LOC_Os09g10840 | bZIP | 0.46 | 7.14 | 1.8E-11 |
| | LOC_Os06g04090 | NAC | 0.44 | 6.87 | 8.06E-11 |
| | LOC_Os05g51160 | MYB_related | 0.43 | 6.71 | 1.92E-10 |
| | LOC_Os09g29820 | bZIP | 0.42 | 6.55 | 5.12E-10 |
| | LOC_Os12g41680 | NAC | 0.42 | 6.70 | 1.78E-10 |
| | LOC_Os04g43560 | NAC | 0.41 | 6.14 | 4.54E-09 |
| | LOC_Os10g01470 | HD-ZIP | 0.39 | 5.91 | 1.51E-08 |
| | LOC_Os05g48850 | NAC | 0.38 | 5.72 | 4E-08 |
| | LOC_Os01g48130 | NAC | 0.38 | 5.91 | 1.41E-08 |
| | LOC_Os12g37970 | MYB | 0.38 | 6.07 | 5.43E-09 |
| | LOC_Os05g48010 | MYB | 0.37 | 5.68 | 4.59E-08 |
| | LOC_Os02g43970 | ERF | 0.37 | 5.39 | 2.18E-07 |
| | LOC_Os06g46270 | NAC | 0.37 | 5.57 | 8.03E-08 |
| | LOC_Os03g42630 | NAC | 0.36 | 5.51 | 1.1E-07 |
| | LOC_Os01g50720 | MYB | 0.36 | 5.46 | 1.46E-07 |
| | LOC_Os02g56600 | NAC | 0.36 | 5.79 | 2.31E-08 |
| | LOC_Os11g05480 | bZIP | 0.36 | 5.23 | 4.49E-07 |
| | LOC_Os02g36880 | NAC | 0.34 | 5.09 | 8.28E-07 |
| | LOC_Os01g53260 | WRKY | 0.34 | 5.12 | 7.35E-07 |
| | LOC_Os08g02300 | NAC | 0.34 | 5.38 | 1.88E-07 |
| | LOC_Os05g38120 | TALE | 0.34 | 5.06 | 9.66E-07 |
| | LOC_Os05g02420 | MYB_related | 0.34 | 5.18 | 5.24E-07 |
| | LOC_Os02g39140 | bHLH | 0.33 | 4.97 | 1.44E-06 |
| | LOC_Os07g42510 | ERF | 0.33 | 5.05 | 9.7E-07 |
| | LOC_Os06g12230 | TCP | 0.33 | 4.94 | 1.64E-06 |
| | LOC_Os07g48596 | G2-like | 0.33 | 4.75 | 4.14E-06 |
| | LOC_Os03g05500 | LBD | 0.33 | 4.87 | 2.32E-06 |
| | LOC_Os09g31390 | bZIP | 0.33 | 4.86 | 2.37E-06 |
| | LOC_Os02g43300 | Trihelix | 0.33 | 4.89 | 2.12E-06 |
| | LOC_Os05g38140 | bHLH | 0.32 | 4.81 | 2.95E-06 |
| | LOC_Os09g16510 | WRKY | 0.32 | 4.77 | 3.55E-06 |
| | LOC_Os01g09550 | NAC | 0.32 | 5.03 | 1.02E-06 |
| | LOC_Os08g15050 | CO-like | 0.32 | 4.71 | 4.72E-06 |
| | LOC_Os04g50920 | WRKY | 0.31 | 4.68 | 5.25E-06 |
| | LOC_Os04g50770 | MYB | 0.31 | 4.56 | 9.08E-06 |
| | LOC_Os07g22770 | ERF | 0.30 | 4.74 | 3.77E-06 |
| | LOC_Os08g02070 | MIKC_MADS | 0.30 | 4.64 | 6.01E-06 |
| | LOC_Os02g47190 | G2-like | 0.30 | 4.44 | 1.51E-05 |
| | LOC_Os04g38720 | NAC | 0.29 | 4.33 | 2.4E-05 |
| | LOC_Os03g46860 | bHLH | 0.29 | 4.29 | 2.74E-05 |
| | LOC_Os01g53040 | WRKY | 0.29 | 4.52 | 1E-05 |
| | LOC_Os09g38340 | C2H2 | 0.29 | 4.25 | 3.24E-05 |
| | LOC_Os02g02820 | bHLH | 0.29 | 4.48 | 1.2E-05 |
| | LOC_Os11g05614 | NAC | 0.29 | 4.17 | 4.51E-05 |
| | LOC_Os05g42130 | GRAS | 0.28 | 4.16 | 4.77E-05 |
| | LOC_Os03g53020 | bHLH | 0.28 | 4.37 | 1.88E-05 |
| | LOC_Os02g08500 | ARR-B | 0.28 | 4.02 | 8.37E-05 |
| | LOC_Os03g64260 | ERF | 0.28 | 4.06 | 7.1E-05 |
| | LOC_Os01g51140 | bHLH | 0.27 | 3.88 | 0.000143 |

| | | | | | |
|---|---|---|---|---|---|
| | LOC_Os12g43950 | TALE | 0.27 | 4.07 | 6.66E-05 |
| | LOC_Os03g08490 | ERF | 0.27 | 3.88 | 0.000142 |
| | LOC_Os06g35140 | G2-like | 0.27 | 3.88 | 0.000144 |
| | LOC_Os02g34970 | NAC | 0.27 | 4.13 | 5.08E-05 |
| | LOC_Os08g39630 | bHLH | 0.27 | 3.93 | 0.000118 |
| | LOC_Os01g36460 | MYB | 0.26 | 3.78 | 0.00021 |
| | LOC_Os03g42430 | B3 | 0.26 | 4.04 | 7.45E-05 |
| | LOC_Os01g52540 | B3 | 0.26 | 3.81 | 0.000188 |
| | LOC_Os07g22730 | ERF | 0.26 | 3.75 | 0.000237 |
| | LOC_Os01g67480 | bHLH | 0.26 | 3.83 | 0.000167 |
| | LOC_Os03g20900 | G2-like | 0.26 | 4.00 | 8.68E-05 |
| | LOC_Os05g37080 | NAC | 0.26 | 3.90 | 0.00013 |
| | LOC_Os05g02390 | C2H2 | 0.26 | 3.71 | 0.000273 |
| | LOC_Os03g29614 | MYB | 0.26 | 3.57 | 0.000459 |
| | LOC_Os09g28354 | HSF | 0.25 | 3.67 | 0.000317 |
| | LOC_Os01g09990 | bHLH | 0.25 | 3.62 | 0.000374 |
| | LOC_Os03g04310 | bHLH | 0.25 | 3.74 | 0.000236 |
| | LOC_Os01g09640 | MYB_related | 0.25 | 3.59 | 0.00041 |
| | LOC_Os03g22170 | ERF | 0.25 | 3.80 | 0.00019 |
| | LOC_Os04g56990 | G2-like | 0.25 | 3.68 | 0.000301 |
| | LOC_Os01g39330 | bHLH | 0.25 | 3.58 | 0.000424 |
| | LOC_Os07g37920 | NAC | 0.24 | 3.65 | 0.000328 |
| | LOC_Os01g51690 | WRKY | 0.24 | 3.61 | 0.000388 |
| | LOC_Os03g07880 | NF-YA | 0.24 | 3.67 | 0.000309 |
| | LOC_Os07g48550 | NAC | 0.24 | 3.75 | 0.000229 |
| | LOC_Os10g26620 | Dof | 0.24 | 3.76 | 0.000217 |
| | LOC_Os02g13800 | HSF | 0.24 | 3.39 | 0.000857 |
| | LOC_Os02g49986 | MYB | 0.24 | 3.32 | 0.001075 |
| | LOC_Os06g49040 | G2-like | 0.24 | 3.37 | 0.000904 |
| | LOC_Os02g44120 | C2H2 | 0.23 | 3.31 | 0.001123 |
| | LOC_Os06g11970 | M-type_MADS | 0.23 | 3.34 | 0.001008 |
| | LOC_Os02g46780 | MYB | 0.23 | 3.47 | 0.000629 |
| | LOC_Os09g11480 | ERF | 0.23 | 3.33 | 0.001019 |
| | LOC_Os01g53650 | C3H | 0.23 | 3.29 | 0.001192 |
| | LOC_Os05g20930 | C2H2 | 0.23 | 3.24 | 0.001418 |
| | LOC_Os03g44710 | YABBY | 0.23 | 3.50 | 0.00056 |
| | LOC_Os09g19950 | LBD | 0.23 | 3.17 | 0.001773 |
| | LOC_Os01g43550 | WRKY | 0.22 | 3.41 | 0.000775 |
| | LOC_Os01g74590 | MYB | 0.22 | 3.20 | 0.001596 |
| | LOC_Os02g15340 | NAC | 0.22 | 3.34 | 0.000967 |
| | LOC_Os01g74140 | WRKY | 0.22 | 3.24 | 0.001398 |
| | LOC_Os08g26880 | bZIP | 0.22 | 3.30 | 0.001145 |
| | LOC_Os10g25170 | ERF | 0.22 | 3.30 | 0.001118 |
| | LOC_Os09g35910 | HD-ZIP | 0.22 | 3.03 | 0.002744 |
| | LOC_Os04g54474 | bZIP | 0.21 | 3.10 | 0.002246 |
| | LOC_Os12g06080 | B3 | 0.21 | 3.23 | 0.001426 |
| | LOC_Os08g33750 | G2-like | 0.21 | 3.20 | 0.001589 |
| | LOC_Os10g39030 | TALE | 0.21 | 3.17 | 0.001724 |
| | LOC_Os07g39800 | HRT-like | 0.21 | 3.22 | 0.001483 |
| | LOC_Os03g42290 | B3 | 0.21 | 3.14 | 0.001902 |
| | LOC_Os12g40590 | bHLH | 0.21 | 3.01 | 0.002947 |
| | LOC_Os04g53990 | bHLH | 0.20 | 3.13 | 0.001976 |
| | LOC_Os02g52190 | bHLH | 0.20 | 2.83 | 0.005142 |
| | LOC_Os05g03884 | TALE | 0.20 | 2.91 | 0.003978 |
| | LOC_Os03g15660 | ERF | 0.20 | 3.05 | 0.002558 |
| | LOC_Os07g03770 | TALE | 0.20 | 2.84 | 0.004964 |
| | LOC_Os03g32230 | C2H2 | 0.20 | 3.06 | 0.002498 |
| | LOC_Os09g33490 | NAC | 0.20 | 2.86 | 0.004661 |
| | LOC_Os03g31240 | C2H2 | 0.20 | 2.96 | 0.003378 |
| | LOC_Os03g21030 | NAC | 0.20 | 2.85 | 0.004846 |
| | LOC_Os08g36920 | ERF | 0.20 | 2.81 | 0.005389 |
| | LOC_Os01g50110 | MYB | 0.20 | 2.88 | 0.004458 |
| | LOC_Os08g32080 | HD-ZIP | 0.19 | 2.72 | 0.007159 |
| | LOC_Os04g43680 | MYB | 0.19 | 2.98 | 0.003202 |
| | LOC_Os01g18870 | bHLH | 0.19 | 2.94 | 0.003666 |

| | | | | | |
|---|---|---|---|---|---|
| | LOC_Os09g36250 | MYB | 0.19 | 2.73 | 0.006937 |
| | LOC_Os03g08460 | ERF | 0.19 | 2.90 | 0.004074 |
| | LOC_Os08g41030 | ERF | 0.19 | 2.84 | 0.00492 |
| | LOC_Os10g39750 | bHLH | 0.19 | 2.75 | 0.006485 |
| | LOC_Os01g70870 | C2H2 | 0.19 | 2.69 | 0.007824 |
| | LOC_Os10g41230 | HD-ZIP | 0.19 | 2.60 | 0.010187 |
| | LOC_Os01g43650 | WRKY | 0.19 | 2.86 | 0.004691 |
| | LOC_Os05g04820 | MYB | 0.19 | 2.73 | 0.006809 |
| | LOC_Os01g03720 | MYB | 0.19 | 2.81 | 0.005337 |
| | LOC_Os05g45410 | HSF | 0.19 | 2.60 | 0.010202 |
| | LOC_Os01g54210 | GATA | 0.18 | 2.69 | 0.007715 |
| | LOC_Os06g09717 | ERF | 0.18 | 2.59 | 0.010304 |
| | LOC_Os03g37920 | FAR1 | 0.18 | 2.76 | 0.006326 |
| | LOC_Os01g09850 | C2H2 | 0.18 | 2.55 | 0.011695 |
| | LOC_Os04g45690 | DBB | 0.18 | 2.72 | 0.007114 |
| | LOC_Os06g17480 | NF-YB | 0.18 | 2.45 | 0.015112 |
| | LOC_Os02g15350 | Dof | 0.18 | 2.56 | 0.011298 |
| | LOC_Os03g55760 | G2-like | 0.18 | 2.64 | 0.008881 |
| | LOC_Os07g48570 | Dof | 0.18 | 2.70 | 0.007506 |
| | LOC_Os01g70110 | NAC | 0.18 | 2.46 | 0.014618 |
| | LOC_Os05g33810 | SBP | 0.18 | 2.60 | 0.010019 |
| | LOC_Os11g05740 | B3 | 0.17 | 2.62 | 0.009472 |
| | LOC_Os05g37190 | C2H2 | 0.17 | 2.62 | 0.009532 |
| | LOC_Os07g48820 | bZIP | 0.17 | 2.48 | 0.01406 |
| | LOC_Os01g11910 | bHLH | 0.17 | 2.39 | 0.01776 |
| | LOC_Os04g45810 | HD-ZIP | 0.17 | 2.55 | 0.011363 |
| | LOC_Os03g08930 | bHLH | 0.17 | 2.35 | 0.019637 |
| | LOC_Os02g40070 | AP2 | 0.17 | 2.56 | 0.011209 |
| | LOC_Os03g20910 | WOX | 0.17 | 2.53 | 0.012193 |
| | LOC_Os12g39330 | ERF | 0.17 | 2.32 | 0.021572 |
| | LOC_Os01g51610 | B3 | 0.17 | 2.44 | 0.015422 |
| | LOC_Os01g18240 | MYB | 0.17 | 2.36 | 0.019465 |
| | LOC_Os04g23910 | MIKC_MADS | 0.17 | 2.34 | 0.020183 |
| | LOC_Os08g04840 | MYB_related | 0.17 | 2.27 | 0.024596 |
| | LOC_Os08g44830 | C2H2 | 0.16 | 2.34 | 0.020144 |
| | LOC_Os05g34050 | bZIP | 0.16 | 2.35 | 0.019519 |
| | LOC_Os04g41560 | DBB | 0.16 | 2.25 | 0.025629 |
| | LOC_Os08g39980 | MYB_related | 0.16 | 2.44 | 0.015447 |
| | LOC_Os07g48260 | WRKY | 0.16 | 2.36 | 0.019219 |
| | LOC_Os08g42470 | bHLH | 0.16 | 2.42 | 0.016144 |
| | LOC_Os04g47040 | bHLH | 0.16 | 2.41 | 0.016603 |
| | LOC_Os03g01870 | NAC | 0.16 | 2.18 | 0.030281 |
| | LOC_Os01g13740 | G2-like | 0.16 | 2.36 | 0.019375 |
| | LOC_Os02g49880 | CO-like | 0.15 | 2.31 | 0.021609 |
| | LOC_Os10g38834 | NAC | 0.15 | 2.14 | 0.033281 |
| | LOC_Os05g03760 | C3H | 0.15 | 2.17 | 0.03119 |
| | LOC_Os10g23050 | bHLH | 0.15 | 2.25 | 0.025674 |
| | LOC_Os03g17810 | LBD | 0.15 | 2.11 | 0.036286 |
| | LOC_Os01g14030 | LBD | 0.15 | 2.19 | 0.029537 |
| | LOC_Os03g10210 | HD-ZIP | 0.15 | 2.12 | 0.035498 |
| | LOC_Os03g55540 | C2H2 | 0.15 | 2.25 | 0.02546 |
| | LOC_Os01g07930 | C3H | 0.15 | 2.11 | 0.036208 |
| | LOC_Os06g45140 | bZIP | 0.15 | 2.10 | 0.037387 |
| | LOC_Os03g51690 | TALE | 0.15 | 2.11 | 0.036454 |
| | LOC_Os10g42130 | NAC | 0.15 | 2.03 | 0.04334 |
| | LOC_Os10g42850 | WRKY | 0.15 | 2.10 | 0.036876 |
| | LOC_Os02g46560 | bHLH | 0.15 | 2.19 | 0.029637 |
| | LOC_Os07g27330 | NAC | 0.15 | 2.01 | 0.045563 |
| | LOC_Os09g26420 | ERF | 0.15 | 2.10 | 0.037044 |
| | LOC_Os03g45300 | FAR1 | 0.14 | 2.15 | 0.033061 |
| | LOC_Os06g03710 | GRAS | 0.14 | 2.16 | 0.031594 |
| | LOC_Os07g48630 | EIL | 0.14 | 2.14 | 0.033335 |
| | LOC_Os02g12310 | NAC | 0.14 | 2.15 | 0.032403 |
| | LOC_Os08g33660 | MYB | 0.14 | 2.12 | 0.034871 |
| | LOC_Os04g47860 | C2H2 | 0.14 | 2.06 | 0.040339 |

| | | | | |
|---|---|---|---|---|
| LOC_Os05g41760 | ERF | 0.14 | 2.05 | 0.041422 |
| LOC_Os03g08470 | ERF | 0.14 | 2.03 | 0.043338 |
| LOC_Os01g29840 | NAC | 0.13 | 1.99 | 0.047912 |
| LOC_Os08g29660 | WRKY | 0.13 | 2.02 | 0.044671 |
| LOC_Os01g63460 | MYB | 0.13 | 1.98 | 0.048647 |
| LOC_Os04g58000 | B3 | 0.13 | 1.99 | 0.047554 |
| LOC_Os01g68700 | bHLH | -0.13 | -2.01 | 0.04613 |
| LOC_Os08g41940 | SBP | -0.13 | -2.01 | 0.045107 |
| LOC_Os02g47660 | bHLH | -0.13 | -1.97 | 0.049579 |
| LOC_Os02g51670 | ERF | -0.14 | -2.06 | 0.040086 |
| LOC_Os03g60560 | C2H2 | -0.14 | -2.04 | 0.042626 |
| LOC_Os02g34680 | C2H2 | -0.14 | -2.04 | 0.042967 |
| LOC_Os02g39520 | FAR1 | -0.14 | -2.00 | 0.046572 |
| LOC_Os09g28310 | bZIP | -0.14 | -2.07 | 0.039467 |
| LOC_Os01g10504 | MIKC_MADS | -0.14 | -1.98 | 0.04972 |
| LOC_Os06g09420 | B3 | -0.14 | -2.07 | 0.039827 |
| LOC_Os01g63160 | MYB | -0.14 | -2.14 | 0.033345 |
| LOC_Os01g62460 | GRAS | -0.14 | -2.13 | 0.034323 |
| LOC_Os05g43380 | CPP | -0.15 | -2.25 | 0.025349 |
| LOC_Os09g13570 | bZIP | -0.15 | -2.11 | 0.035823 |
| LOC_Os09g29930 | bHLH | -0.15 | -2.06 | 0.040847 |
| LOC_Os05g41780 | ERF | -0.15 | -2.28 | 0.023415 |
| LOC_Os05g11414 | MIKC_MADS | -0.15 | -2.15 | 0.032942 |
| LOC_Os12g07120 | GATA | -0.15 | -2.31 | 0.021579 |
| LOC_Os04g59430 | ARF | -0.15 | -2.33 | 0.020969 |
| LOC_Os02g53150 | GeBP | -0.15 | -2.23 | 0.026748 |
| LOC_Os10g41460 | Trihelix | -0.16 | -2.28 | 0.023804 |
| LOC_Os04g42950 | MYB | -0.16 | -2.23 | 0.026902 |
| LOC_Os03g02900 | B3 | -0.16 | -2.33 | 0.020892 |
| LOC_Os11g11100 | bZIP | -0.16 | -2.23 | 0.026741 |
| LOC_Os07g42400 | FAR1 | -0.16 | -2.40 | 0.01715 |
| LOC_Os04g47890 | G2-like | -0.16 | -2.42 | 0.016362 |
| LOC_Os12g38490 | GRAS | -0.16 | -2.31 | 0.021968 |
| LOC_Os01g50940 | bHLH | -0.16 | -2.21 | 0.028278 |
| LOC_Os06g06750 | MIKC_MADS | -0.16 | -2.24 | 0.026067 |
| LOC_Os02g02424 | C2H2 | -0.16 | -2.29 | 0.02304 |
| LOC_Os08g31580 | ERF | -0.16 | -2.23 | 0.027255 |
| LOC_Os12g13170 | bZIP | -0.16 | -2.45 | 0.014924 |
| LOC_Os01g13460 | bHLH | -0.16 | -2.40 | 0.017287 |
| LOC_Os01g59350 | bZIP | -0.16 | -2.48 | 0.014055 |
| LOC_Os09g01140 | GeBP | -0.16 | -2.32 | 0.021617 |
| LOC_Os02g07780 | SBP | -0.17 | -2.34 | 0.020432 |
| LOC_Os01g14870 | C3H | -0.17 | -2.36 | 0.019035 |
| LOC_Os04g32620 | ERF | -0.17 | -2.52 | 0.012591 |
| LOC_Os06g02230 | B3 | -0.17 | -2.52 | 0.012526 |
| LOC_Os04g41850 | Nin-like | -0.17 | -2.54 | 0.011707 |
| LOC_Os10g41200 | MYB_related | -0.17 | -2.56 | 0.010975 |
| LOC_Os01g48060 | ARF | -0.17 | -2.54 | 0.01181 |
| LOC_Os07g04700 | MYB | -0.17 | -2.55 | 0.011367 |
| LOC_Os04g56850 | ARF | -0.17 | -2.55 | 0.011398 |
| LOC_Os07g08140 | HSF | -0.17 | -2.56 | 0.010991 |
| LOC_Os11g30484 | C2H2 | -0.17 | -2.44 | 0.015496 |
| LOC_Os05g11510 | DBB | -0.17 | -2.46 | 0.01479 |
| LOC_Os09g12750 | G2-like | -0.17 | -2.49 | 0.013517 |
| LOC_Os01g62660 | G2-like | -0.17 | -2.50 | 0.013094 |
| LOC_Os10g41130 | ERF | -0.18 | -2.69 | 0.00776 |
| LOC_Os04g50060 | GRAS | -0.18 | -2.70 | 0.0075 |
| LOC_Os03g08500 | ERF | -0.18 | -2.54 | 0.011834 |
| LOC_Os02g33430 | E2F/DP | -0.18 | -2.73 | 0.00693 |
| LOC_Os05g10670 | C3H | -0.18 | -2.58 | 0.010626 |
| LOC_Os12g42400 | NF-YA | -0.18 | -2.75 | 0.006406 |
| LOC_Os01g61810 | NF-YB | -0.18 | -2.54 | 0.011878 |
| LOC_Os02g32590 | HSF | -0.18 | -2.72 | 0.007146 |
| LOC_Os01g14720 | GeBP | -0.18 | -2.79 | 0.005742 |
| LOC_Os11g32100 | bHLH | -0.18 | -2.65 | 0.008663 |

| | | | | | |
|---|---|---|---|---|---|
| | LOC_Os06g49010 | SBP | -0.18 | -2.58 | 0.010649 |
| | LOC_Os04g46860 | GRAS | -0.19 | -2.83 | 0.00509 |
| | LOC_Os04g58020 | MYB_related | -0.19 | -2.77 | 0.006168 |
| | LOC_Os06g04870 | HD-ZIP | -0.19 | -2.84 | 0.004943 |
| | LOC_Os05g41070 | bZIP | -0.19 | -2.69 | 0.007841 |
| | LOC_Os12g41860 | HD-ZIP | -0.19 | -2.86 | 0.004701 |
| | LOC_Os01g17000 | Dof | -0.19 | -2.87 | 0.004558 |
| | LOC_Os06g49550 | FAR1 | -0.19 | -2.68 | 0.008029 |
| | LOC_Os05g50080 | C3H | -0.19 | -2.77 | 0.00607 |
| | LOC_Os02g42380 | TCP | -0.19 | -2.95 | 0.003532 |
| | LOC_Os02g53670 | MYB_related | -0.19 | -2.98 | 0.003248 |
| | LOC_Os03g54160 | MIKC_MADS | -0.20 | -2.78 | 0.00589 |
| | LOC_Os04g55560 | AP2 | -0.20 | -2.98 | 0.003201 |
| | LOC_Os06g43090 | MYB | -0.20 | -2.85 | 0.00485 |
| | LOC_Os12g41230 | CPP | -0.20 | -3.03 | 0.002781 |
| | LOC_Os03g09280 | GRAS | -0.20 | -3.03 | 0.002704 |
| | LOC_Os03g11370 | B3 | -0.20 | -2.87 | 0.004555 |
| | LOC_Os03g55590 | G2-like | -0.20 | -3.15 | 0.001831 |
| | LOC_Os07g38240 | C2H2 | -0.20 | -2.98 | 0.003214 |
| | LOC_Os01g15460 | C3H | -0.20 | -3.10 | 0.002217 |
| | LOC_Os05g38460 | MYB | -0.20 | -2.96 | 0.003448 |
| | LOC_Os03g31230 | MYB_related | -0.21 | -3.07 | 0.002417 |
| | LOC_Os08g37920 | C2H2 | -0.21 | -3.08 | 0.002332 |
| | LOC_Os11g06010 | bHLH | -0.21 | -2.93 | 0.003797 |
| | LOC_Os08g41950 | MIKC_MADS | -0.21 | -2.93 | 0.00377 |
| | LOC_Os03g63810 | WRKY | -0.21 | -2.96 | 0.003492 |
| | LOC_Os02g49440 | Dof | -0.21 | -3.17 | 0.001721 |
| | LOC_Os11g47900 | GRAS | -0.21 | -2.96 | 0.003515 |
| | LOC_Os07g44200 | GeBP | -0.21 | -3.22 | 0.001475 |
| | LOC_Os12g10660 | DBB | -0.21 | -3.27 | 0.00124 |
| | LOC_Os09g32040 | NAC | -0.21 | -3.09 | 0.002315 |
| | LOC_Os12g41210 | CPP | -0.22 | -3.30 | 0.001138 |
| | LOC_Os06g49080 | C3H | -0.22 | -3.16 | 0.001808 |
| | LOC_Os06g04850 | HD-ZIP | -0.22 | -3.16 | 0.001804 |
| | LOC_Os12g38940 | C2H2 | -0.22 | -3.32 | 0.00106 |
| | LOC_Os12g06640 | Trihelix | -0.22 | -3.41 | 0.000774 |
| | LOC_Os06g11860 | ERF | -0.22 | -3.16 | 0.001847 |
| | LOC_Os03g55990 | HB-other | -0.22 | -3.40 | 0.000803 |
| | LOC_Os03g01890 | HD-ZIP | -0.22 | -3.42 | 0.000732 |
| | LOC_Os06g02560 | GRF | -0.22 | -3.37 | 0.000873 |
| | LOC_Os03g58250 | bZIP | -0.22 | -3.36 | 0.000916 |
| | LOC_Os12g41950 | ARF | -0.22 | -3.33 | 0.00102 |
| | LOC_Os06g35900 | BES1 | -0.22 | -3.22 | 0.00151 |
| | LOC_Os02g29340 | HSF | -0.23 | -3.46 | 0.000648 |
| | LOC_Os08g40900 | ARF | -0.23 | -3.28 | 0.001218 |
| | LOC_Os07g38090 | C3H | -0.23 | -3.51 | 0.000548 |
| | LOC_Os01g09620 | C3H | -0.23 | -3.35 | 0.000981 |
| | LOC_Os10g17630 | B3 | -0.23 | -3.44 | 0.000702 |
| | LOC_Os08g37580 | HD-ZIP | -0.23 | -3.47 | 0.000641 |
| | LOC_Os03g51970 | GRF | -0.23 | -3.49 | 0.00058 |
| | LOC_Os11g09160 | B3 | -0.24 | -3.40 | 0.000804 |
| | LOC_Os08g43160 | TCP | -0.24 | -3.34 | 0.000992 |
| | LOC_Os12g40570 | WRKY | -0.24 | -3.67 | 0.0003 |
| | LOC_Os08g25799 | G2-like | -0.24 | -3.52 | 0.000531 |
| | LOC_Os04g57610 | ARF | -0.24 | -3.64 | 0.000339 |
| | LOC_Os03g55220 | bHLH | -0.24 | -3.69 | 0.000288 |
| | LOC_Os08g36790 | bZIP | -0.24 | -3.53 | 0.000521 |
| | LOC_Os02g45480 | C3H | -0.24 | -3.51 | 0.000558 |
| | LOC_Os01g68160 | C2H2 | -0.24 | -3.61 | 0.000387 |
| | LOC_Os03g42280 | B3 | -0.25 | -3.60 | 0.000408 |
| | LOC_Os05g51830 | C2H2 | -0.25 | -3.76 | 0.00022 |
| | LOC_Os03g29760 | NF-YA | -0.25 | -3.79 | 0.000194 |
| | LOC_Os06g40710 | G2-like | -0.25 | -3.65 | 0.000328 |
| | LOC_Os06g37450 | GATA | -0.25 | -3.55 | 0.000489 |
| | LOC_Os10g41770 | STAT | -0.25 | -3.82 | 0.000175 |

| | | | | |
|---|---|---|---|---|
| LOC_Os08g01090 | B3 | -0.25 | -3.87 | 0.000142 |
| LOC_Os03g58830 | bHLH | -0.25 | -3.71 | 0.000272 |
| LOC_Os02g34590 | FAR1 | -0.26 | -3.96 | 0.000101 |
| LOC_Os09g30400 | WRKY | -0.26 | -3.77 | 0.000215 |
| LOC_Os03g63530 | NF-YC | -0.26 | -3.98 | 9.34E-05 |
| LOC_Os07g39310 | C2H2 | -0.26 | -3.77 | 0.000215 |
| LOC_Os05g41450 | NF-YC | -0.26 | -3.78 | 0.000206 |
| LOC_Os01g65900 | GRAS | -0.26 | -4.04 | 7.49E-05 |
| LOC_Os01g15350 | C3H | -0.26 | -4.10 | 5.74E-05 |
| LOC_Os01g13300 | B3 | -0.26 | -4.01 | 8.4E-05 |
| LOC_Os02g58670 | bZIP | -0.26 | -4.11 | 5.57E-05 |
| LOC_Os02g42870 | MYB | -0.26 | -3.87 | 0.000145 |
| LOC_Os04g52560 | FAR1 | -0.27 | -4.10 | 5.86E-05 |
| LOC_Os01g70270 | ARF | -0.27 | -4.17 | 4.29E-05 |
| LOC_Os02g19804 | C3H | -0.27 | -3.91 | 0.000129 |
| LOC_Os07g43030 | CAMTA | -0.27 | -4.12 | 5.37E-05 |
| LOC_Os01g09760 | MYB_related | -0.27 | -4.23 | 3.43E-05 |
| LOC_Os03g54170 | MIKC_MADS | -0.27 | -4.17 | 4.36E-05 |
| LOC_Os03g02160 | C3H | -0.27 | -4.24 | 3.26E-05 |
| LOC_Os03g62660 | FAR1 | -0.27 | -4.18 | 4.14E-05 |
| LOC_Os07g25710 | G2-like | -0.27 | -4.21 | 3.79E-05 |
| LOC_Os07g39480 | WRKY | -0.28 | -4.33 | 2.27E-05 |
| LOC_Os01g42710 | LSD | -0.28 | -4.38 | 1.83E-05 |
| LOC_Os02g06910 | ARF | -0.28 | -4.42 | 1.52E-05 |
| LOC_Os01g10610 | BES1 | -0.28 | -4.41 | 1.6E-05 |
| LOC_Os02g45850 | B3 | -0.28 | -4.11 | 5.7E-05 |
| LOC_Os11g35030 | GRF | -0.28 | -4.16 | 4.81E-05 |
| LOC_Os02g34630 | MYB_related | -0.29 | -4.36 | 2.01E-05 |
| LOC_Os11g06410 | Trihelix | -0.29 | -4.16 | 4.67E-05 |
| LOC_Os03g58160 | HSF | -0.29 | -4.52 | 9.75E-06 |
| LOC_Os03g21240 | G2-like | -0.29 | -4.49 | 1.12E-05 |
| LOC_Os06g40960 | C2H2 | -0.29 | -4.27 | 3.02E-05 |
| LOC_Os05g03020 | C2H2 | -0.29 | -4.30 | 2.65E-05 |
| LOC_Os03g50110 | GeBP | -0.29 | -4.57 | 8.15E-06 |
| LOC_Os01g32890 | GeBP | -0.29 | -4.38 | 1.88E-05 |
| LOC_Os01g70810 | HB-other | -0.30 | -4.51 | 1.08E-05 |
| LOC_Os02g18370 | FAR1 | -0.30 | -4.64 | 6.05E-06 |
| LOC_Os12g06380 | FAR1 | -0.30 | -4.63 | 6.37E-06 |
| LOC_Os02g33750 | FAR1 | -0.30 | -4.48 | 1.25E-05 |
| LOC_Os09g13940 | ERF | -0.30 | -4.45 | 1.45E-05 |
| LOC_Os03g11614 | MIKC_MADS | -0.30 | -4.29 | 2.89E-05 |
| LOC_Os12g29520 | ARF | -0.30 | -4.74 | 3.74E-06 |
| LOC_Os09g35760 | HD-ZIP | -0.30 | -4.32 | 2.51E-05 |
| LOC_Os08g17400 | WRKY | -0.30 | -4.70 | 4.54E-06 |
| LOC_Os03g53340 | HSF | -0.30 | -4.81 | 2.71E-06 |
| LOC_Os07g13170 | AP2 | -0.30 | -4.49 | 1.22E-05 |
| LOC_Os04g45650 | GATA | -0.31 | -4.84 | 2.42E-06 |
| LOC_Os02g33560 | bZIP | -0.31 | -4.87 | 2.13E-06 |
| LOC_Os04g02730 | C3H | -0.31 | -4.66 | 5.74E-06 |
| LOC_Os10g41260 | MYB_related | -0.31 | -4.74 | 4.02E-06 |
| LOC_Os10g42490 | HD-ZIP | -0.32 | -4.97 | 1.31E-06 |
| LOC_Os07g43420 | MYB | -0.32 | -5.02 | 1.02E-06 |
| LOC_Os08g19590 | HD-ZIP | -0.32 | -4.66 | 5.79E-06 |
| LOC_Os06g46410 | ARF | -0.32 | -4.65 | 6.11E-06 |
| LOC_Os10g22950 | CAMTA | -0.32 | -5.09 | 7.45E-07 |
| LOC_Os06g14190 | NF-X1 | -0.32 | -4.75 | 3.86E-06 |
| LOC_Os02g05510 | GATA | -0.32 | -5.12 | 6.48E-07 |
| LOC_Os08g37904 | C2H2 | -0.33 | -4.85 | 2.55E-06 |
| LOC_Os01g54550 | HSF | -0.34 | -5.32 | 2.57E-07 |
| LOC_Os08g23470 | B3 | -0.34 | -5.15 | 6.06E-07 |
| LOC_Os08g28214 | CPP | -0.34 | -5.07 | 9.38E-07 |
| LOC_Os08g42440 | CO-like | -0.34 | -5.51 | 9.7E-08 |
| LOC_Os03g08370 | FAR1 | -0.35 | -5.44 | 1.42E-07 |
| LOC_Os09g29460 | HD-ZIP | -0.35 | -5.19 | 5.13E-07 |
| LOC_Os03g13790 | MYB_related | -0.35 | -5.53 | 8.87E-08 |

| | | | | | |
|---|---|---|---|---|---|
| | LOC_Os07g35870 | bHLH | -0.35 | -5.62 | 5.67E-08 |
| | LOC_Os12g41920 | MYB_related | -0.35 | -5.61 | 5.82E-08 |
| | LOC_Os02g10840 | FAR1 | -0.35 | -5.71 | 3.59E-08 |
| | LOC_Os01g42970 | C3H | -0.35 | -5.30 | 3.09E-07 |
| | LOC_Os04g11830 | TCP | -0.36 | -5.33 | 2.72E-07 |
| | LOC_Os04g57600 | C3H | -0.36 | -5.69 | 4.06E-08 |
| | LOC_Os03g15010 | FAR1 | -0.36 | -5.71 | 3.51E-08 |
| | LOC_Os12g42970 | GATA | -0.36 | -5.59 | 6.87E-08 |
| | LOC_Os09g24820 | ZF-HD | -0.36 | -5.29 | 3.35E-07 |
| | LOC_Os01g67970 | C2H2 | -0.36 | -5.52 | 1.05E-07 |
| | LOC_Os06g46890 | C3H | -0.36 | -5.50 | 1.19E-07 |
| | LOC_Os06g07010 | FAR1 | -0.37 | -5.79 | 2.53E-08 |
| | LOC_Os08g04170 | C3H | -0.37 | -5.97 | 9.23E-09 |
| | LOC_Os05g09630 | HB-other | -0.37 | -5.98 | 8.53E-09 |
| | LOC_Os03g63750 | HSF | -0.37 | -5.91 | 1.3E-08 |
| | LOC_Os07g44640 | C2H2 | -0.37 | -5.83 | 2.04E-08 |
| | LOC_Os03g06860 | FAR1 | -0.37 | -5.90 | 1.37E-08 |
| | LOC_Os12g18150 | C2H2 | -0.37 | -6.01 | 7.27E-09 |
| | LOC_Os04g35800 | C3H | -0.37 | -5.68 | 4.68E-08 |
| | LOC_Os01g48700 | E2F/DP | -0.38 | -5.86 | 1.83E-08 |
| | LOC_Os02g18660 | GeBP | -0.38 | -5.84 | 2.15E-08 |
| | LOC_Os06g45840 | MYB_related | -0.38 | -5.94 | 1.2E-08 |
| | LOC_Os07g07974 | CPP | -0.39 | -6.02 | 8.12E-09 |
| | LOC_Os05g25320 | FAR1 | -0.40 | -6.15 | 4.16E-09 |
| | LOC_Os07g30774 | CAMTA | -0.40 | -6.28 | 2.01E-09 |
| | LOC_Os01g72490 | SRS | -0.40 | -6.22 | 2.9E-09 |
| | LOC_Os06g41384 | C3H | -0.40 | -6.00 | 1.01E-08 |
| | LOC_Os02g33610 | Trihelix | -0.40 | -6.04 | 8.34E-09 |
| | LOC_Os02g39540 | FAR1 | -0.41 | -6.66 | 2.15E-10 |
| | LOC_Os07g37630 | FAR1 | -0.41 | -6.59 | 3.24E-10 |
| | LOC_Os03g50900 | FAR1 | -0.41 | -6.52 | 5.04E-10 |
| | LOC_Os02g06370 | Whirly | -0.41 | -6.32 | 1.74E-09 |
| | LOC_Os06g16400 | bHLH | -0.41 | -6.64 | 2.52E-10 |
| | LOC_Os04g53540 | HD-ZIP | -0.41 | -6.31 | 1.76E-09 |
| | LOC_Os03g05480 | C2H2 | -0.41 | -6.72 | 1.55E-10 |
| | LOC_Os03g53960 | MYB_related | -0.42 | -6.78 | 1.09E-10 |
| | LOC_Os05g27930 | ERF | -0.42 | -6.37 | 1.41E-09 |
| | LOC_Os01g04020 | ERF | -0.42 | -6.83 | 8.34E-11 |
| | LOC_Os03g13614 | bZIP | -0.42 | -6.59 | 3.84E-10 |
| | LOC_Os06g41770 | bZIP | -0.42 | -6.70 | 2E-10 |
| | LOC_Os01g64560 | bHLH | -0.42 | -6.98 | 3.48E-11 |
| | LOC_Os03g60120 | ERF | -0.44 | -7.22 | 8.08E-12 |
| | LOC_Os01g06550 | NF-X1 | -0.44 | -7.32 | 4.46E-12 |
| | LOC_Os06g04010 | BBR-BPC | -0.46 | -7.82 | 1.95E-13 |
| | LOC_Os06g12400 | HB-PHD | -0.47 | -7.47 | 2.3E-12 |
| | LOC_Os04g28090 | MYB | -0.47 | -8.04 | 5.32E-14 |
| | LOC_Os03g05690 | C2H2 | -0.48 | -8.07 | 4.66E-14 |
| | LOC_Os04g40930 | Trihelix | -0.48 | -7.98 | 1E-13 |
| | LOC_Os04g40060 | FAR1 | -0.51 | -8.82 | 3.53E-16 |
| | LOC_Os02g05450 | HB-other | -0.52 | -8.63 | 1.86E-15 |
| | LOC_Os03g27390 | bHLH | -0.53 | -9.37 | 8.93E-18 |
| | LOC_Os03g12120 | NAC | -0.60 | -11.26 | 1.48E-23 |
